# Perspectives in Myrtaceae evolution from plastomes and nuclear phylogenies

**DOI:** 10.1590/1678-4685-GMB-2021-0191

**Published:** 2022-01-21

**Authors:** Natalia Balbinott, Nureyev Ferreira Rodrigues, Frank Lino Guzman, Andreia Carina Turchetto-Zolet, Rogerio Margis

**Affiliations:** 1 Universidade Federal do Rio Grande do Sul, Departamento de Biofísica, Laboratório de Genomas e Populações de Plantas, Porto Alegre, RS, Brazil.; 2 Universidade Federal do Rio Grande do Sul, Programa de Pós-graduação em Biologia Celular e Molecular, Porto Alegre, RS, Brazil.; 3 Universidade Federal do Rio Grande do Sul, Departamento de Genética, Instituto de Biociências, Programa de Pós-Graduação em Genética e Biologia Molecular, Porto Alegre, RS, Brazil.; 4 Instituto Nacional de Innovación Agraria, Dirección de Recursos Genéticos y Biotecnología, Lima, Perú.

**Keywords:** cpDNA, Eucalypteae, Myrteae, phylogenetics, Syzygieae

## Abstract

Myrtaceae is a large and species-rich family of woody eudicots, with prevalent distribution in the Southern Hemisphere. Classification and taxonomy of species belonging to this family is quite challenging, sometimes with difficulty in species identification and producing phylogenies with low support for species relationships. Most of the current knowledge comes from few molecular markers, such as plastid genes and intergenic regions, which can be difficult to handle and produce conflicting results. Based on plastid protein-coding sequences and nuclear markers, we present a topology for the phylogenetic relationships among Myrtaceae tribes. Our phylogenetic estimate offers a contrasting topology over previous analysis with fewer markers. Plastome phylogeny groups the tribes Syzygieae and Eucalypteae and individual chloroplast genes produce divergent topologies, especially among species within Myrteae tribe, but also in regard to the grouping of Syzygieae and Eucalypteae. Results are consistent and reproducible with both nuclear and organellar datasets. It confronts previous data about the deep nodes of Myrtaceae phylogeny.

## Introduction

Myrtaceae is a large family of pantropical woody eudicots with a Gondwanan origin and distributed predominantly in the Southern Hemisphere (Thornhill et al., 2015). Comprising over 5,650 species, Myrtaceae is considered the eighth largest family of flowering plants and one of the most species-rich families in the Neotropics (Govaerts et al., 2015). Representatives of the family are commonly found in many biodiversity hotspots and are ecologically relevant components of wet forests in South America, Australia, and tropical Asia. Besides, many genera of the family display economic importance by the provision of food resources, essential oils, timber and fiber, such as *Syzygium, Psidium, Eugenia, Eucalyptus* and *Melaleuca* (Stefanello et al., 2011; de Araújo et al., 2019; de Paulo Farias et al., 2020). 

Since its first description, the Myrtaceae family has been ground for a great difficulty of classification and species delimitation, going through many revisions over the past decades. The current classification groups species in two subfamilies (Psiloxyloideae and Myrtoideae) and 17 tribes (Wilson et al., 2005). The prevailing knowledge on Myrtaceae phylogeny comes from the use of a few non-coding molecular markers, such as rRNA internal transcribed spacers (ITS), plastid marker genes and chloroplast intergenic regions, used to resolve relationships from species to family level (Thornhill et al., 2015; Vasconcelos et al., 2017; Mazine et al., 2018; Amorim et al., 2019; Lucas et al., 2019). Many works focused on the relationships among Myrtaceae tribes using different sampling and phylogenetic approaches in an attempt to obtain a good resolution of these relationships (Sytsma et al., 2004; Wilson *et al.*, 2005; Biffin et al., 2010; Maurin et al., 2021).

Most of the sequences used for phylogenetic inference of green plants are from plastid origins, which are believed to experience slower evolutionary rates when compared to nuclear loci (Palmer et al., 1988). Besides, the conservation of sequence organization and content in the chloroplast genome ensures the orthology of the sequences used for reconstruction, one of the main difficulties assessing nuclear regions other than ITS (Álvarez and Wendel, 2003). It is accepted that molecular studies should include multiple markers to evaluate the complete evolutionary history of species, accounting for phylogenetic signals from different genomic sources to produce an accurate representation of species phylogeny (Doyle, 1992).

Despite their ease to obtain through PCR amplification, the effectiveness of few sequences to fully reconstruct phylogenetic relationships at deep levels has been questioned, with plastome phylogenetics emerging as a powerful alternative for reconstructing species relationships (Davis et al., 2014; Ruhfel et al., 2014; Barrett et al., 2016; Wang et al., 2020; Wei and Zhang, 2020). The rise of Next-Generation Sequencing (NGS) made it easy to perform plastome sequencing, increasing the availability of complete chloroplast genomes and changing molecular systematics, population and conservation genetics (Soltis et al., 2013). Since a small set of sequences may represent a limited source of informative molecular characters to resolve difficult relationships, the whole plastome represents an alternative to settle previously contentious and unresolved systematic problems (Ruhfel *et al.*, 2014; Barrett *et al.*, 2016; Bedoya et al., 2019; Wang *et al.*, 2020; Wei and Zhang, 2020).

While maintaining the benefits of using chloroplast DNA (cpDNA) for phylogenetic reconstruction, such as slow mutation rate, haploid inheritance and conservation of gene content and order (Palmer et al., 1988; Davis et al., 2014), the whole plastome harbors a greater source of phylogenetically informative characters, with potential to provide resolution and support for deeper relationships involving rapid diversification in short periods (Barrett et al., 2016; Foster et al., 2018; Nie et al., 2020). The advances in plastome sequencing enabled the use of large datasets and resulted in the clarification of previously unsettled relationships of basal angiosperms, besides providing additional support for previously estimated relationships at deeper levels and resolving rapid radiations difficult to do so with smaller datasets (Jansen et al., 2007; Moore et al., 2007; Moore *et al.*, 2010). 

Among the nuclear markers widely used to infer species relationships, the ITS region is by far the most commonly used (Schmid, 1972; da Cruz et al., 2013; Hussain et al., 2019). The ITS displays many valuable features for phylogenetic reconstruction, such as simplicity, universality, and sequence variation (Álvarez and Wendel, 2003). However, concerted evolution, sequence saturation, and difficulties in performing alignments are characteristics of the ITS region, potentially inducing wrong topologies, especially when trying to reconstruct deeper nodes (Álvarez and Wendel, 2003).

As an alternative to the ITS sequences, single-copy nuclear genes have increasingly been used for phylogenetic analysis (Duarte et al., 2010; Zhang et al., 2012; Luo et al., 2019). Besides being a valuable information for biparental inheritance due to their nuclear origin, protein-coding nuclear markers show lower homoplasy than ITS regions. Nuclear coding genes are a powerful source of phylogenetically informative characters and do not present the issues associated with the use of ITS (Álvarez and Wendel, 2003). Besides, the codon organization facilitates homologous comparisons and reduces the effects of misalignments in the phylogenetic inference. Zhang *et al.* (2012) identified low-copy nuclear genes with inherent qualities of effective markers for reconstructing angiosperm phylogeny, which provided similar results to those previously inferred from organellar genes and uncovered new placements for a range of lineages. In this perspective, nuclear single-copy genes have great potential for angiosperm phylogenetic reconstruction and can provide new insights into the evolutionary history of plant species.

In the current work, we present a new topology for Myrtaceae relationships within tribes and species, reconstructed from the entire set of plastid protein-coding sequences and also supported by a set of four single-copy nuclear markers.

## Methods

### Taxon sampling

Sampling was performed in an attempt to maximize tribal representativeness of the family, despite the limited amount of complete sequenced chloroplast genomes from most Myrtaceae tribes. The complete plastomes of Myrtaceae species deposited in NCBI were retrieved, along with *Punica granatum* (Lythraceae), which was used as an outgroup due to the absence of complete plastomes for other closely related species to Myrtaceae by the time the analysis was performed. For those species, the 18-26S internal transcribed spacers (ITS) were also obtained from NCBI for phylogenetic inference, when available. A complete list of species and vouchers are available in Tables S1 and S2. 

Besides the ITS, for the analysis of the nuclear markers we also used the coding-sequences of *MSH1, MLH1, SMC1, SMC2*, and *MCM5* described by Zhang et al. (2012) as highly conserved single-copy genes suitable for phylogenetic inference. *Eucalyptus grandis* nuclear markers were obtained from the complete genome deposited in Phytozome13 (https://phytozome.jgi.doe.gov/) and used as a query for BLAST searches of other species sequences, conducted against complete genomes available in NCBI and transcriptomes from 1KP database (Table S3). To obtain the sequences of the five nuclear markers of *Eugenia uniflora*, *E. brasiliensis*, *E. selloi*, *E. pyriformis*, *Myrcianthes pungens*, *Plinia trunciflora, Psidium cattleyanum* and *Syzygium cumini,* we used reads from DNAseq and RNAseq libraries sequenced by our group (Rodrigues et al., 2020; unpublished data). Quality check of the raw reads was done with FastQC software (https://www.bioinformatics.babraham.ac.uk/projects/fastqc/) and the remaining adapter sequences, unknown bases, and low-quality ends below 30 were trimmed using Trim Galore! (https://www.bioinformatics.babraham.ac.uk/projects/trim_galore/). The remaining reads of each library were *de novo* assembled using Abyss (Simpson et al., 2009) and Trinity (Grabherr et al., 2011) to obtain the genomic and transcriptomic sequences for each species, respectively. Because the nuclear markers sequences matched several contigs after a BLASTx search (Altschul et al., 1990), we decided to use *E. grandis* coding sequences as references to map the cleaned reads of the libraries using Bowtie (Langmead et al., 2009) and obtained individual fastq files with reads for each locus. We used these files in the SPADES assembler (Bankevich et al., 2012) to obtain the *de novo* assembled genes for each species. In the assembly of each locus, we used the optimal kmer that resulted in the complete sequence in a single contig. In the case of the sequences obtained with genomic data, the coding sequence was predicted using FGENESH (Solovyev et al., 2006), while for the transcriptomic data, it was identified with ORFinder (https://www.ncbi.nlm.nih.gov/orffinder/).

Under the impossibility of obtaining *Melaleuca quinquenervia* nuclear markers from the 1KP database, nuclear genes were assembled *de novo* from transcriptome data deposited in European Nucleotide Archive (https://www.ebi.ac.uk/ena - study accession PRJNA357284) using *E. grandis* coding sequences as references, as described above. Information on accession numbers and vouchers for all the sequences used in this study are available in Table S3.

### Phylogenetic analysis

Our datasets consist of chloroplast protein-coding genes, nuclear marker genes (*MSH1, MLH1, SMC1, SMC2,* and *MCM5*) and ITS. For the former two, phylogenetic analyses were conducted for individual genes and also concatenating genes, allowing each gene to have its own partition. Both nuclear marker genes and chloroplast coding sequences were individually aligned by codon using MUSCLE (Edgar, 2004) default options implemented in MEGA7 (Kumar et al., 2016), while ITS sequences were aligned by nucleotide. All alignments were visually inspected to avoid misaligned nucleotides and were manually corrected if necessary. 

Bayesian phylogenetic inference for all datasets was performed with BEAST 2.6 (Bouckaert et al., 2019), with an uncorrelated lognormal relaxed clock to allow for rate variation among lineages and the Yule model as a prior for branching rates. When using multiple loci for the same analysis, a single relaxed clock was applied for the complete dataset, allowing each locus to have a distinct relative rate. Best-fit nucleotide substitution models were calculated with ModelTest-NG (Darriba *et al.*, 2020) and selected according to AIC. Posterior distributions of the parameters were estimated via Markov chain Monte Carlo (MCMC) sampling, with data from two independent runs combined for each analysis. Details on the nucleotide substitution models, chain length, and sampling frequency for each dataset are available in Supplementary Table S4. The software Tracer 1.7.1 (Rambaut et al., 2018) was used to assess run convergence and certify that the effective sample sizes (ESS) for all the parameters were sufficient (> 200). The trees from individual runs were combined with LogCombiner using a burn-in of 20% and a maximum clade credibility tree was generated with TreeAnnotator. Maximum-likelihood phylogenetic analyses for the nuclear and plastid dataset were performed with RAxML-NG v. 0.9.0, using the best-fit model for each gene and 10,000 bootstrap replicates. Outputs of the phylogenetic analysis were visualized using FigTree 1.4.4 (Rambaut, 2014).

### Test of conflicting topologies of plastid genes

Plastid genes can be a source of incongruence when evaluated individually, generating conflicting topologies that yet may share identical internal nodes. To identify the conflicts and concordances among plastid genes, we used phyparts (Smith et al., 2015) to examine the shared internal edges (bipartitions) across the different topologies. It allows the identification and mapping of concordant, conflicting, and unique bipartitions. After performing this analysis with the 78 plastid genes, we accessed which genes had more parsimony informative sites and subsequently used them for conflict analysis. A parsimony-informative site was defined as a site with at least two types of nucleotides with at least two of them occurring with a minimum frequency of two, as specified by MEGA7 (Kumar et al., 2016). 

### Divergence time estimation

To compare the divergence time estimates to other published datasets, we co-estimated phylogeny and divergence times using BEAST 2.6 (Bouckaert et al., 2019), with chloroplast concatenated protein-coding sequences and nine fossil calibrations, and also using the five nuclear marker genes supermatrix and eight fossil calibrations (Table S5). Fossils used varied according to species present in each dataset. The fossilized birth and death model (Heath et al., 2014) was used as a prior to integrating fossil information into the diversification process of the lineages, along with an uncorrelated relaxed lognormal clock to allow for branch variation. Run convergence assessment and sampled trees combining was performed as previously described in section 2.2.

## Results

### Phylogenetic relationships vary according to the dataset

**Figure 1 -:**
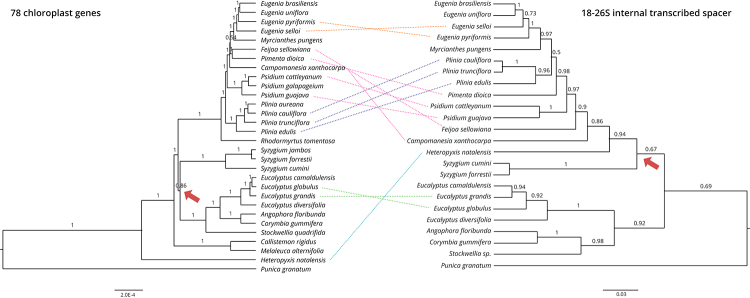
Maximum clade-credibility phylogeny of Myrtaceae using 78 chloroplast protein-coding genes (left) and internal transcribed spacers (right). Posterior probabilities for each node are indicated and branch lengths are scaled according to the number of substitutions per site. Red arrows indicate the node grouping the tribe Syzygieae with either Eucalypteae or Myrteae.

**Figure 2 -:**
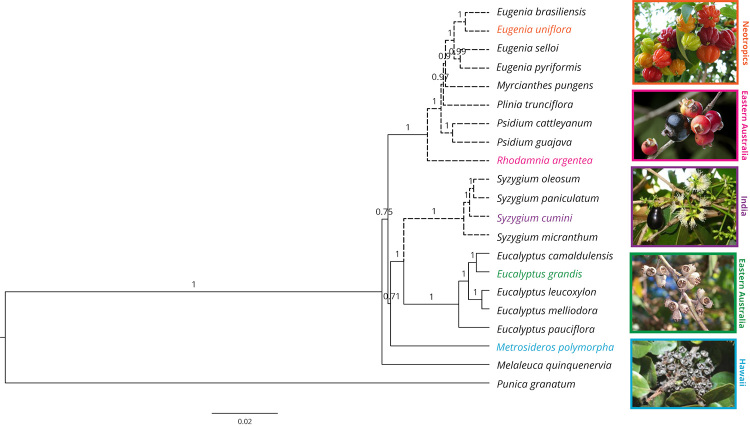
Maximum clade-credibility phylogeny of Myrtaceae using concatenated sequences of five single-copy nuclear markers *MCM5, MLH1, MSH1, SMC1* and *SMC2*. Posterior probabilities for each node are indicated and branch lengths are scaled according to the number of substitutions per site. Dashed lines correspond to species with fleshy fruits in the Myrtaceae family. Species with colored scientific names are those represented in pictures on the right, along with an indication of species occurrence.

The maximum clade credibility trees reconstructed from chloroplast coding sequences, nuclear genes, or ITS sequences produce different topologies, with variation in species, subtribe, and tribe placements. Despite the lack of fully sequenced chloroplast genomes, both Bayesian and Maximum-likelihood phylogenetic analysis with 78 plastid protein-coding sequences yielded a consistent and well-supported phylogeny and successfully recovered the monophyly of the tribes (PP = 1.0, Figure 1). Nuclear marker genes and ITS datasets are also both capable of recovering the monophyly of Myrteae, Syzygieae, and Eucalypteae, with higher support provided by the nuclear marker genes (Figures 1 and 2). Besides the lower support provided by the ITS, the mere use of the ITS for phylogenetic reconstruction results in the grouping of *Heteropyxis natalensis* with Myrteae (PP > 0.94; Figure 1), placing the subfamily Psiloxyloideae within Myrtoideae (Figure 1). The use of nuclear markers individually is also capable of recovering the monophyly of the tribes Myrteae, Syzygieae and Eucalypteae (Figure S1).

The major difference from our data to previously published studies is the positioning of Syzygieae (indicated by arrows in Figure 1). Bayesian phylogenetic inference using either chloroplast protein-coding genes or five nuclear marker genes results in the grouping of Syzygieae and Eucalypteae with high support (PP = 1, Figures 1 and 2). The topologies generated using *MSH1, SMC1,* and *SMC2* individually also show strong support for this relationship (PP > 0.95), while *MLH1* and *MCM5* are also capable of recovering this relationship but with less significative support (PP = 0.66 and 0.62, respectively) (Figure S1). Contrastingly, ITS groups Syzygieae with Myrteae + Heteropyxideae with low support (PP = 0.67; Figure 1).

All datasets are successful in recovering the subtribes within Myrteae and Eucalypteae in accordance with current knowledge, with variations in the relationships among subtribes and species. In the tribe Eucalypteae, the chloroplast dataset positions *Stockwellia quadrifida* as an external branch to the other *Eucalypteae* species, while the ITS recovers the grouping of *Stockwellia* sp. with *Angophora* + *Corymbia* (Figure 1). 

The relationships within Myrteae recovered by plastid and nuclear datasets highlight the monophyly of the subtribes Eugeniinae and Pliniinae. However, subtribe Pimentinae appears to be paraphyletic. Plastid data recover a closer relationship between Eugeniinae and Pimentinae, while ITS recovers a closer relationship between Eugeniinae and Pliniinae (Figure 1). Combined nuclear markers, however, place *Plinia trunciflora* closer to Eugeniinae than representants of the subtribe Pimentinae.

Discordances among interspecific grouping also can be seen when comparing topologies produced by distinct datasets. The plastid sequences result in the grouping of *Feijoa sellowiana* and *Pimenta dioica*, with *Campomanesia xanthocarpa* as an external branch, placing species from the genera *Psidium* even more externally. The ITS topology displays a fairly different species positioning within the subtribe Pimentinae, with *C. xanthocarpa* external to all species of the subtribe, followed by *A. sellowiana* and *Psidium* species positioned more internally along with *Pimenta dioica* (Figure 1). 

The combined use of the protein-coding nuclear markers allowed for the basal positioning of *Melaleuca quinquenervia* with high support (PP = 1), as well as the positioning of *Metrosideros polymorpha* as an outer branch of the clade including Syzygieae and Eucalypteae with low support (0.71) (Figure 2). All genes could recover the monophyly of the tribes included in the analysis, with the exception of *MSH1,* which resulted in the exclusion of *Rhodamnia argentea* of the Myrteae clade with low support (PP = 0.47 - Figure S1). 

Phylogenies reconstructed by a maximum-likelihood approach using plastid coding sequences and five nuclear markers recovered the same topology as the Bayesian inferences of the respective datasets, with high support for the monophyly of the tribes (BS = 100, Supplementary Figures S2 and S3) and the Syzygieae + Eucalypteae relationship (BS of 99 and 74 for the nuclear and plastid data, respectively; Figures S2 and S3).

### Individual plastid coding genes produce different topologies

The phylogenies generated from each plastid protein-coding sequence showed a high level of variability. Many plastid genes are poor in phylogenetic information to reconstruct a reliable species phylogeny, particularly those that are short and highly conserved, such as components of the photosystem complex *psbM* and *psbT* (Figure S4). 

To further access conflicting plastid gene topologies, we performed a bipartition analysis to determine their level of concordance with the topology inferred from the complete dataset and their predominance. An analysis of the 78 protein-coding sequences revealed a high level of incongruence among plastid genes, more frequently in the interspecific relationships of the Myrteae tribe. Thirty-five genes show an internal node shared by Eucalypteae and Syzygieae, while 43 were discordant for this bipartition (Figure S5). Although this analysis is informative, it masks the many possibilities of species grouping within a bipartition. Also, it includes topologies recovered from sequences with few informative sites, which can be a source of noise in the analysis.

To reduce the potential impact of non-phylogenetic signals in the analysis and generate a more consistent result, we selected sequences with a minimum of parsimony-informative sites to perform a conflict analysis. Thirty-five parsimony-informative sites were defined as a cut-off for gene selection according to their ability to recover the monophyly of the tribes. A set of 20 out of 78 chloroplast genes satisfied these criteria (Table 1, Table S6).

**Table 1 -:** Twenty plastid genes used for concordance analysis due to their phylogenetic effectiveness, ranked according to the tribe grouping, they reconstruct and the posterior probability of the branch.

Tribe clustering	Gene	PP	Shorter sequence (nt)	Longer sequence (nt)	Parsim-info sites	Alignment size
(Syzygieae, Eucalypteae)	*ycf1*	1	5661	5808	539	3144
(Syzygieae, Eucalypteae)	*psaA*	0.99	2253	2253	38	2253
(Syzygieae, Eucalypteae)	*rpoC1*	0.86	2070	2070	36	2040
(Syzygieae, Eucalypteae)	*ndhD*	0.68	1512	1512	69	1503
(Syzygieae, Eucalypteae)	*rpoB*	0.66	3222	3222	79	3213
(Syzygieae, Eucalypteae)	*petA*	0.49	963	963	36	963
(Syzygieae, Eucalypteae)	*rps3*	0.49	657	657	42	651
(Syzygieae, Eucalypteae)	*ycf2*	0.49	6921	6969	79	6825
(Syzygieae, Eucalypteae)	*rbcL*	0.46	1459	1480	61	1428
(Syzygieae, Eucalypteae)	*ccsA*	0.43	960	975	51	975
(Syzygieae, Eucalypteae)	*accD*	0.41	1461	1479	67	1494
(Syzygieae, Eucalypteae)	*psbB*	0.39	1527	1527	38	1527
(Myrteae, Syzygieae)	*ndhF*	0.97	2292	2304	150	2199
(Myrteae, Syzygieae)	*psaB*	0.66	2205	2205	47	2205
(Myrteae, Syzygieae)	*rpoC2*	0.65	4220	4229	162	4116
(Myrteae, Syzygieae)	*matK*	0.41	1557	1572	106	1479
((Eucalypteae, Myrteae) Syzygieae)	*atpB*	0.96	1494	1497	42	1497
Syzygieae with outgroup	*rpl22*	n/a	282	513	58	282
Syzygieae with outgroup with Myrteae	*rps12*	n/a	390	402	71	351
Syzygieae within Eucalypteae (Syzygieae + *E. globosus*)	*ndhA*	n/a	1104	1107	46	1062

When reconstructing Myrtaceae phylogeny using the 20 coding sequences with more parsimony-informative characters, the same topology as the complete dataset with 78 genes is recovered, with a slight reduction on the posterior probability of the branch grouping *A. sellowiana + P. dioica* and the subtribe Eugeniinae (Figure S6).

**Figure 3 -:**
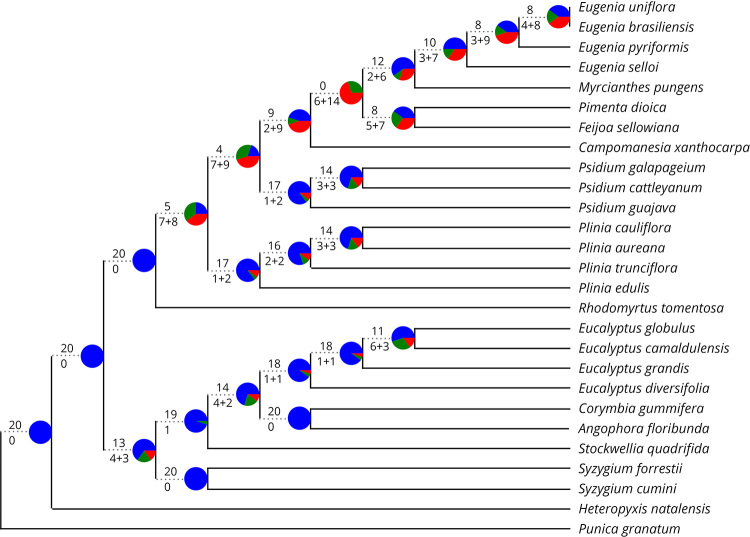
Bipartition analysis of Myrtaceae phylogeny using the topologies generated individually by the 20 chloroplast protein-coding sequences against the topology of the. Pie charts and the numbers in the nodes represent the concordant (blue), discordant (red) and discordant prevalent alternative (green) bipartitions. Numbers above the branch correspond to the concordant bipartitions, and numbers under the branch represent the number of genes supporting a discordant prevalent bipartition + other discordant bipartitions. Information on number of tree topology and alignment data are present in Table 1 and Figure S2.

Thirteen out of the 20 genes used for the bipartition analysis support the grouping of Eucalypteae and Syzygieae (Figure 3). Those genes encompasses different chloroplast functions: fatty acid metabolism (*accD*)*,* energy metabolism (*petA*), oxireduction (*ndhD*), photosystem components (*psaA and psbB*), polymerases (*rpoB and rpoC1*), ribosomal structure (*rps3*), protein transport and assembly (*ccsA, ycf1, ycf2*) and RuBisCo large chain (*rbcL*). The discordant genes that support a closer relationship between Myrteae and Syzygieae also cover some of these same functions, such as *psaB, rpoC2,* and those widely used for phylogenetic reconstruction *matK* and *ndhF*. The use of *atpB* produces a uniquely different topology, grouping Eucalypteae, and Myrteae, with Syzygieae placed in an external branch (Figure S4).

### Divergence time estimation

**Figure 4 -:**
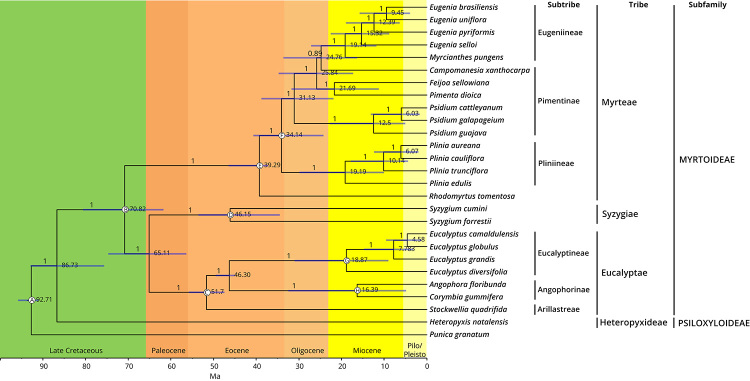
Chronogram of Myrtaceae species based on a supermatrix of 78 plastid genes. Posterior probabilities for each node are indicated and nodal ages represent the median posterior estimates. Branch lengths are scaled according to time and blue bars represent the 95% highest posterior density interval of age estimates. Subtribal, tribal and subfamilial classifications of Myrtaceae species are indicated on the right. Calibrated nodes are labelled from A-H corresponding to fossil calibration detailed in Table S5.

**Figure 5 -:**
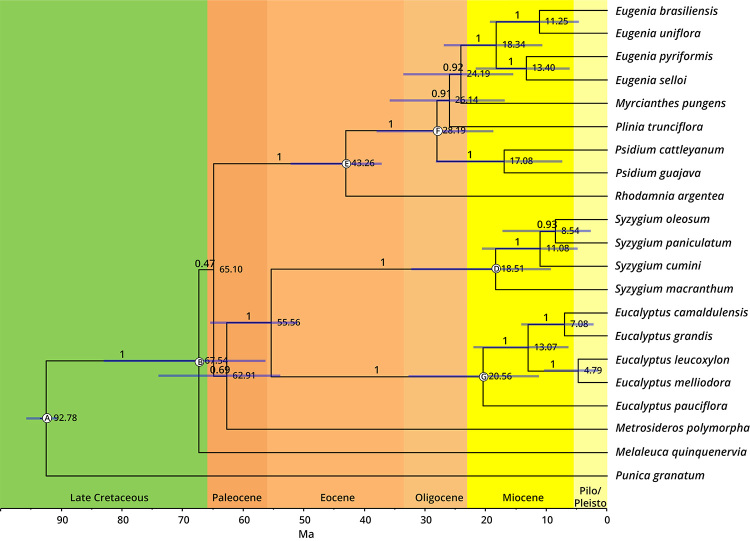
Chronogram of Myrtaceae species based on the five nuclear marker genes supermatrix. Posterior probabilities for each node are indicated and nodal ages represent the median posterior estimates. Branch lengths are scaled according to time and blue bars represent the 95% highest posterior density interval of age estimates. Calibrated nodes are labelled from A, B, D-G corresponding to fossil calibration detailed in Table S5.

Two chronograms were reconstructed based on chloroplast protein-coding sequences and five nuclear markers using nine and eight crown fossil calibrations, respectively (Figures 4 and 5, Table S5). Most of the node age estimates obtained are consistent between the two datasets (Table 2). The family emergence was estimated on the Late Cretaceous (91 - 95.83 Ma), mostly due to the calibration used on the basal node. The divergence of the two subfamilies Psiloxyloideae and Myrtoideae was also estimated during the Late Cretaceous (75.66 - 92.93 Ma), consistent with previous works (Thornhill et al., 2015). Another divergence event within the subfamily Myrtoideae was placed in the Late Cretaceous, involving the split of the tribe Myrteae and the clade with the tribes Syzygieae and Eucalypteae (62.02 - 80.28 Ma). The crown ages for the three tribes of Myrtoideae present in the analysis were estimated during the Eocene, contrasting the estimate for the tribe Syzygieae in Thornhill *et al.* (2015).

**Table 2 -:** Estimates for species divergence times and substitution rates of the Bayesian analysis. Calibrated nodes are indicated by an asterisk (*).

	Plastid dataset					Nuclear dataset				
	Crown age (Ma)			Substitution rate		Crown age (Ma)			Substitution rate	
Group	PP	Mean	95% HPD	Mean	95% HPD	PP	Mean	95% HPD	Mean	95% HPD
Myrtales*	n/a	92.7058	91 - 95.83	1	n/a	n/a	92.7817	91 - 96.10	7.09E-04	4.90E-04 - 1.83E-03
Myrtoideae crown*	1	70.82	62.02 - 80.28	3.92E-04	1.70E+04 - 7.84E-04	1	67.5396	56.49 - 83.25	9.71E-04	6.50E-05 - 2.65E-03
Eucalypteae crown*	1	51.7	47.45 - 55.84	3.04E-04	7.13E-05 - 5.94E-04	n/a	n/a	n/a	n/a	n/a
Myrteae crown*	1	39.29	37.2 - 46.5	1.89E-04	3.23E-05 - 3.52E-04	1	43.26	37.20 - 52.31	5.75E-04	6.70E-05 - 1.25E-03
Syzygieae crown*	1	46.15	34.5 - 53.56	1.96E-04	1.47E-05 - 4.71E-04	1	18.51	9.17 - 32.31	4.34E-04	1.30E-04 - 7.37E-04
South American Myrteae crown*	1	34.14	24.21 - 40.65	8.68E-05	1.46E-05 - 2.23E-04	1	28.19	18.74 - 38.12	5.28E-04	6.50E-05 - 1.35E-03
*Eucalyptus* crown*	1	18.87	9.01 - 31.01	1.08E-04	1.81E-05 - 2.25E-04	1	20.56	11.23 - 32.86	6.08E-04	7.20E-05 - 1.29E-03
Angophora/Corymbia crown*	1	16.39	4.93 - 32.55	8.69E-05	3.19E-05 - 1.65E-04	n/a	n/a	n/a	n/a	n/a
Pliniineae	1	19.19	10.06 - 29.87	1.25E-04	4.00E-05 - 2.77E-04	n/a	n/a	n/a	n/a	n/a
Eugeniineae	1	19.14	11.86 - 27.16	1.62E-04	3.40E-05 - 3.74E-04	0.92	24.19	15.45 - 33.68	4.58E-04	5.60E-05 - 1.16E-03
*Psidium* group	1	12.5	5.07 - 22.71	1.14E-04	4.89E-05 - 2.22E-04	1	17.08	7.32 - 28.3	4.94E-04	1.19E-04 - 1.11E-03
Eucalypteae and Syzygieae divergence	1	65.11	56.42 - 74.69	1.75E-04	3.04E-05 - 4.08E-04	1	55.56	51.20 - 65.40	6.41E-04	9.77E-05 - 1.44E-03
*Melaleuca* divergence from other spp*	n/a	n/a	n/a	n/a	n/a	1	67.54	56.5 - 83.26	4.89E-04	3.34E-04 - 6.46E-04
*Metrosideros* divergence from Syzygieae and Eucalypteae*	n/a	n/a	n/a	n/a	n/a	0.69	62.91	54.03 - 74.18	4.19E-04	8.60E-05 - 7.88E-04

The divergence of the tribes Syzygieae and Eucalypteae, a relationship described with great support and by extensive analyses in the present work, was predicted to the end of the Cretaceous and beginning of Paleocene (56.42 - 74.69 Ma for the chloroplast dataset and 51.20 - 65.40 Ma for the nuclear dataset). The Syzygieae crown node age estimate was contrasting between both datasets, with an older estimate for the chloroplast dataset (34.5 - 53.56 Ma) in comparison with the nuclear marker genes (9.17 - 32.31 Ma). 

Another conflicting result between the two datasets places Syzygieae crown either in the Eocene or in the Miocene. The chloroplast dataset gives an older estimate for the node age (34.5 - 53.56 Ma), while the nuclear marker genes estimate for the Syzygieae crown (9.17 - 32.31 Ma) coincides with Thornhill et al. (2015) estimates. In addition, the divergence of Angophora/Corymbia crown is placed in the Miocene-Oligocene (4.93 - 32.55 Ma) by the chloroplast dataset, while previous estimates place this node in the Eocene.

Within the tribe Myrteae, divergence time estimations for the Australasian species, Pliniineae, Eugeniineae and *Psidium* species were consistent among datasets and with previous estimates for these groups (Vasconcelos et al., 2017). 

## Discussion

We present the first report on Myrtaceae relationships inferred from the complete set of 78 protein-coding sequences from the chloroplast genome using a Bayesian framework and supported by Maximum-likelihood. Unfortunately, sampling was limited by the simultaneous availability of full plastome and nuclear sequences. Although our analyses do not cover all the tribes belonging to Myrtaceae, the substantial amount of informative characters used may counteract the unavailability of plastid and nuclear data for many representatives of the family. More importantly, the nature of molecular sequences and their informativeness have a direct effect on their ability to resolve phylogenetic problems and produce more accurate phylogenies (Rosenberg and Kumar, 2003). In that sense, plastome phylogenomic analyses are powerful approaches to resolve unclear relationships using a set of genes to help increase the phylogenetic signal and potentially overwhelm conflicting signals. The present work is an opportunity to evaluate the use of plastome-scale datasets to reconstruct Myrtaceae evolutionary history.

The result of our phylogenomic analysis with the plastome is consistent, displays high branch support with posterior probability values of one for the majority of the branches, and is highly concordant with the topology inferred from the five single-copy nuclear marker genes. However, inconsistencies among tribes and species grouping were identified when comparing our data to the phylogeny inferred solely from ITS and previously published data on Myrtaceae phylogeny with few markers.

With the expansion of studies assessing the relationships within Myrtaceae tribes or genera, many ITS and plastid markers sequences (e.g. *ndhF*, *matK*, *rbcL*, tRNA intergenic regions) became available, and subsequent works focusing on the whole family phylogeny used molecular data from the same genetic sources (Biffin et al., 2010; Murillo-A. et al., 2012; Thornhill et al., 2015, 2019; Schuster et al., 2018; Giaretta et al., 2019). With that approach, the flesh-fruited tribes Syzygieae and Myrteae were proposed to form a clade along with Backhousieae, Kanieae, Metrosidereae and Tristanieae, tribes including species with dry capsular fruits. This clade, referred to as BKMMST clade, indicates that the emergence of the fleshy fruit in the tribes Syzygieae and Myrteae occurred independently (Biffin *et al.*, 2010). 

The topologies provided by *matK* and *ndhF* individually (Figure S4) recover the grouping of Syzygieae and Myrteae, in agreement with the results presented by Biffin et al. (2010). However, plastome coding sequences and nuclear marker gene topologies are discordant for that grouping and recover a closer relationship between the tribes Syzygieae and Eucalypteae (Figure 1 and 2), contrasting with the widely used plastid markers. Recent work using the Angiosperms353 probe kit had similar results, showing that fleshy-fruit tribes may be more distantly related than previously believed (Maurin et al., 2021).

Although *matK* and *ndhF* individually support the grouping of Syzygieae and Myrteae, the concatenation of these regions with *rpl16* and six plastid intergenic regions (*psbA-trnH*, *trnL-trnF, trnQ-rps16, rpl32-trnL)* show a well-supported relationship between Eucalypteae and Syzygieae (Vasconcelos et al., 2017). Previous works based on limited sampling, focused mostly in the description of Myrtaceae and Lythraceae chloroplast genomes, also present phylogenies that support a closer relationship between Syzygieae and Eucalypteae (Eguiluz et al., 2017a,b; Gu et al., 2019; Liu et al., 2019; Liu *et al.*, 2020; Machado et al., 2020), reinforcing our results with analyses based on protein-coding genes and whole chloroplast genomes. Yet, when *S. jambos* is included in our analysis, along with two species from the tribe Melaleuceae (Figure S8), the grouping of Syzygieae and Eucalypteae is recovered.

Despite the lack of representants for the BKMMST clade, both plastid and nuclear topologies are also concordant with multiple emergence events of fleshy fruits within Myrtaceae. The basal positioning of *Melaleuca quinquenervia* in the phylogeny supports the idea of the emergence of fleshy fruits from a dry ancestral fruit form (Figure 2).

Although the majority of plant phylogenetic studies still rely on few markers for reconstruction, whole plastid genomes and all shared protein-coding genes have been useful and often provided greater phylogenetic resolution when compared with multilocus analysis of few marker genes, especially when investigating deep phylogenetic relationships, but also at family and genera level (Davis et al., 2014; Ruhfel et al., 2014; Barrett et al., 2016; Ran et al., 2018; Zong et al., 2019; Wang et al., 2020; Wei and Zhang, 2020). The growing use of plastomes for phylogenetic inference is potentially linked to a combination of the increasing availability of complete sequenced chloroplast genomes and an abundance of informative molecular characters obtained from the plastome, in comparison with a small set of genes from multi-gene studies.

When assessing phylogenies inferred from individual plastid sequences, a high level of conflict in the recovered topologies is observed, with many genes lacking phylogenetically informative sites to resolve species relationships and with significant inconsistencies among topologies (Figures S4 and S3). Due to the slow evolving rate nature of the plastid genome, many of the incongruences may be a result of the rapid radiation of species in a short period. Still, it cannot be discarded that the source of the discordance may be conflicting phylogenetic signals, such as distinct nucleotide substitutions and evolutionary rates among genes. Most individual plastid gene trees can recover the monophyly of the tribe Myrteae but cannot resolve interspecific relationships of the group (Figure S5). Due to the rapid diversification process believed to have occurred in this group (Biffin et al., 2010), it is reasonable that sequences with slow evolutionary rates, such as the plastid protein-coding sequences, might not be informative enough to resolve species phylogeny individually. In this case, non-coding sequences are a better source of phylogenetically informative characters to resolve low-rank relationships (Murillo-A et al., 2012; Alwadani et al., 2019). 

Previous works have extensively assessed the incongruences of plastid protein-coding inferred phylogenies, identifying the variation of genetic signal and how it can be used to expand the understanding of species relationships (Gonçalves et al., 2019; Walker et al., 2019). Recently, *rpoC2*, *ycf1* and *ycf2* were identified as possible substitutes for the current widely used *matK*, *ndhF* and *rbcL* plastid markers (Walker *et al.*, 2019), mostly due to the greater phylogenetic information provided by longer sequences. Although in our analysis *rpoC2* phylogeny has a topology consistent with *matK* and *ndhF*, the use of either *ycf1* or *ycf2* for the phylogenetic inference recovered a closer relationship between the tribes Syzygieae and Eucalypteae (Figure S4). When all protein-coding sequences from the plastid genome are combined for the analysis, the same relationship among tribes is recovered with higher branch support, either in a Bayesian framework (Figure 1) or in a maximum-likelihood estimation (Figure S2), pointing to the notion that some genes may share different evolutionary histories. 

Restricting the data to sequences with the strong phylogenetic signal can considerably reduce the level of conflict and assist in drawing conclusions about the phylogenetic hypothesis of Myrtaceae. When analysing the topologies recovered individually by the 20 coding sequences with more than 36 parsimony-informative sites, most genes produce a topology concordant with the obtained from the complete plastome dataset (Figure 3; Table 1). 

Although displaying a great number of parsimony-informative characters, *rpl22, rps12* and *ndhA* were unable to recover the monophyly of the tribes or correctly place the outgroup. A trans-splicing present in the first exon of *rps12* was identified as the major source of phylogenetic information in the alignment; that in combination with a short sequence could bias the phylogenetic result. Surprisingly, *rps12* and *rps22*, along with *rps32*, show the greater percentages of parsimony-informative sites per alignment (Table S1), indicating that an excess of such characters may negatively impact the capacity of the sequences in individually recovering a reliable topology. Also, Myrteae species share a deletion in nucleotide 439 of *rpl22* that changes the reading frame, resulting in an early stop codon and distinct amino acids, suggesting a specific evolutionary history for this gene in this clade which is not shared by other Myrtaceae species. Further analysis is necessary to clarify the impact this deletion may represent to the function of the ribosomal large subunit of these species.

When combining the same 20 coding sequences in a multilocus approach, the grouping of Eucalypteae and Syzygieae is still recovered, with a slight reduction of the posterior probability in Myrteae internal branches and no alteration in support of deeper branches (Figure S6), meaning that eliminating sequences with less phylogenetic information has little impact in the resulting reconstruction. Although the number of parsimony-informative sites is not a direct measure of the phylogenetic utility of the sequences, it can be used as a metric to approximate it and our results suggest that the sequences holding the most parsimony-informative characters are the major sources of phylogenetically informative characters of the complete plastome, reinforcing the results obtained with the analysis of the 78 plastid coding sequences.

Chloroplast sequences have been long used for phylogenetic inference, especially due to their slow mutation rate in comparison with nuclear sequences, their relatively small size, conserved order in the genome, and the haploid inheritance of the chloroplast (Palmer et al., 1988). 

Our results reinforce that the ITS alone cannot be a reliable source of phylogenetically informative characters for the reconstruction of Myrtaceae evolutionary history, especially when evaluating deeper nodes. Although most of the phylogenetic inferences of Myrtaceae combine the ITS with other marker genes, we argue that the use of ITS might negatively impact the reconstruction of deeper relationships due to the lack of informative sites for this level of analysis. When performing reconstruction of the same species relationships using ITS, the resulting phylogeny had lower posterior probabilities for most of the branches and displayed inconsistencies, such as the grouping of *H. natalensis*, a member of the subtribe Heteropyxideae (subfamily Psiloxyloideae), within the subfamily Myrtoideae. 

Thus, the positioning of *Heteropyxis* along with Myrteae is inaccurate and clearly a methodological artifact, mostly due to the high variability of the ITS and the limited amount of informative characters present in the sequence for reconstructing a deep node. Therefore, the inclusion of the ITS in phylogenetic reconstructions may be a source of noise in the analysis, masked by the inclusion of other regions that are more informative. Along with the high variability of the sequence, the lack of closely related species to *H. natalensis* could be a factor influencing its erroneous grouping, once the occurrence of multiple substitutions in the same position over time could reduce the informativeness of the sequence for deeper nodes.

In the specific case of Myrtaceae, the final topology of the phylogeny inferred from ITS combined with *ndhF* and *matK* (Biffin et al., 2010) resembles those obtained from *ndhF* and *matK* individually (Figure S1). This could be an indicator that these two sequences are greatly responsible for the resulting topology, accounting for most of the phylogenetic information supporting it. Therefore, we argue that the inclusion of ITS data in the phylogenetic analysis focusing on resolving the deep nodes of Myrtaceae might serve as a greater source of non-phylogenetically informative characters for these relationships rather than informative.

As alternatives to the ITS sequences, single-copy nuclear genes account for biparental inheritance and provide a vast amount of phylogenetically informative characters for species evolution reconstruction. We used five nuclear markers described by Zhang et al. (2012) as effective for the reconstruction of angiosperm relationships at both above- and below-order levels. These genes are longer and phylogenetically more informative than ITS and include both rapid and slowly evolving genes, accounting for a range of taxonomy hierarchies. Besides, due to the conservation of the function of the genes, it is less likely that any adaptive or environmentally driven selective pressure is acting on them (Zhang *et al.*, 2012). 

A closer relationship between Syzygieae and Eucalypteae is recovered when combining the five nuclear genes *MCM5*, *MLH1, MSH1, SMC1,* and *SMC2*, concordant with the phylogeny reconstructed from the plastome dataset. The results of Zhang et al. (2012) agree with phylogenetic reconstruction performed previously with 83 plastid genes, with almost 80% of concordance. The high compatibility with results obtained from plastid data and the above-mentioned nuclear markers demonstrate their adequacy for phylogenetic reconstruction. A combination of the five nuclear markers above-mentioned with other 54 low-copy nuclear genes provided robust resolution for deep branches in angiosperm phylogeny, highlighting the suitability of nuclear coding sequences to resolve deep phylogenetic relationships (Zeng et al., 2014).

When evaluating tree topologies for individual nuclear genes, all five genes are concordant with Eucalypteae and Syzygieae grouping (Figures S1 and S7). For *MSH1*, *SMC1* and *SMC2*, the posterior probabilities of the branches supporting this relationship are higher than 0.95, while *MLH1* and *MCM5* display lower posterior probabilities but still recover the same clade, emphasizing the results obtained with plastid sequences. Most of the species positioning in the individual gene phylogeny are consistent with the one obtained from the combined nuclear genes, with fluctuations in the positioning of *M. polymorpha* and *M. quinquenervia*.

Zhang et al. (2012) identified *SMC1* as the best-performing gene out of the five nuclear markers used in the study. In our analysis, *SMC1* recovered the most similar topology to the combined nuclear genes, and therefore the plastome topology, with alterations in low-rank relationships among Syzygieae and Myrteae species and decrease in branch support (Figure S1). Unfortunately, as for the chloroplast genomes, there is not enough full genomic and transcriptomic information on Myrtaceae species available to perform a phylogenetic reconstruction with larger sampling.

Most of the divergence time estimates produced by our data are in accordance with previously described crown ages for the family Myrtaceae (Thornhill et al., 2015; Vasconcelos et al., 2017) with contrasting estimates produced for the Syzygieae and Angophora/Corymbia crown between datasets. Distinct estimates for these nodes may be a result of rate variation among lineages and among loci. Also, the number of taxa and loci used for the analyses, along with distinct taxon sampling for the two datasets may impact the precision of the obtained node age estimates, explaining the differences between plastome and nuclear marker genes estimates, as well as differences from our results to previously published data (Soares and Schrago, 2015; O’Reilly and Donoghue, 2020). The higher support of the plastome estimates in comparison with the nuclear marker genes presumes that node ages resulting from the plastome dataset are more accurate. Besides, the inclusion of more loci reduces the uncertainty of the estimates (Zhu et al., 2015), emphasizing the credibility of the obtained ages. It is important to note that the disparity in age estimates for some nodes may be explained by the lack of species representing the referred tribes. In that sense, the analysis may provide proper age estimates for the divergence of the analysed species, which may differ from the estimated age of the tribe. 

Our results offer insights into the evolution of Myrtaceae, drawn from a phylogenomic analysis based on plastid and nuclear coding sequences, indicating a closer relationship between Syzygieae and Eucalypteae. We used the complete set of coding sequences from Myrtaceae plastomes to consistently infer the evolutionary history of the family. Various levels of conflict were identified among individual plastid genes topologies, ITS, and nuclear marker genes, demonstrating that different datasets display distinct capacity of relationship resolution. The discordances observed might be produced by conflicting phylogenetic signals given by each analysed gene, either as a result of independent evolutionary histories or systematic error. Our results reinforce the need to use both organellar and nuclear sequences for phylogenetic reconstruction, highlighting the limitations and influences of the ITS in the reconstruction of Myrtaceae phylogeny. Our work also emphasizes the lack of both plastid and nuclear data available for most of the tribes within Myrtaceae, with major genomic and transcriptomic data available for Eucalypteae, Myrteae, and Syzygieae, stressing the need for more studies in this area. The inclusion of more species in the analysis with the sequencing of more plastomes and nuclear genes would expand and provide a stronger basis for the understanding of the evolutionary history of the family.

## Supplementary material

The following online material is available for this article:

Table S1 - Information on the species plastome accessions used for the phylogenetic inference.

Table S2 - Information on the species ITS accessions used for the phylogenetic inference.

Table S3 - Identification for the nuclear sequences used for the phylogenetic analysis.

Table S4 - Information of BEAST runs.

Table S5 - Details of fossils data used for time divergence estimation.

Table S6 - Information on the variation level of each plastid gene.

Figure S1 - Individual maximum clade-credibility phylogenies of Myrtaceae inferred from the five single-copy nuclear markers.

Figure S2 - Maximum-likelihood tree inferred from the 78 plastid protein-coding sequences.

Figure S3 - Maximum-likelihood tree inferred from the five nuclear protein-coding markers.

Figure S4 - Individual maximum clade-credibility phylogenies of Myrtaceae inferred from the 78 plastid protein-coding sequences.

Figure S5 - Bipartition analysis of Myrtaceae phylogeny using the five single-copy nuclear markers *MCM5, MLH1, MSH1, SMC1* and *SMC2*.

Figure S6 - Bayesian phylogenetic estimation of phylogeny using the 20 coding sequences with more parsimony-informative characters.

Figure S7 - Bipartition analysis of Myrtaceae phylogeny using the five single-copy nuclear markers *MCM5, MLH1, MSH1, SMC1* and *SMC2*.

Figure S8 - Bayesian phylogenetic estimation using 78 chloroplast protein-coding genes, including the species *Syzygium ambos*, *Callistemon rigidus* and *Melaleuca alternifolia*.
