# Supplementary material for: Perspectives in Myrtaceae evolution from plastomes and nuclear phylogenies
Source: Genet Mol Biol. 2022 Jan 21;45(1):e20210191. doi: 10.1590/1678-4685-GMB-2021-0191 (PMC8796035; doi:10.1590/1678-4685-GMB-2021-0191)
Supplement: Table S1 - [file 1415-4757-GMB-45-1-e20210191-s1.pdf]

Genetics and Molecular Biology
https://doi.org/10.1590/1678-4685-GMB-2021-0191

# Supplementary Material to “Perspectives in Myrtaceae evolution from plastomes and nuclear phylogenies”

**Table S1** - Information on the species plastome accessions used for the phylogenetic inference.

| Tribe | Species | Plastome accession | Voucher no. plastome | Source |
|---|---|---|---|---|
| Eucalypteae | *Angophora floribunda* (Sm.) Sweet | NC_022412.1 | 108351 | NCBI |
| Eucalypteae | *Corymbia gummifera* (Gaertn.) K.D.Hill & L.A.S.Johnson | NC_022407.1 | 108355 | NCBI |
| Eucalypteae | *Eucalyptus camaldulensis* Dehnh. | NC_022398.1 | 108435 | NCBI |
| Eucalypteae | *Eucalyptus diversifolia* Bonpl. | NC_022383.1 | 108362 | NCBI |
| Eucalypteae | *Eucalyptus globulus* Labill. | NC_008115.1 | HO528199 | NCBI |
| Eucalypteae | *Eucalyptus grandis* W.Hill ex Maiden | MG925369.1 | - | NCBI |
| Eucalypteae | *Stockwellia quadrifida* D..Carr, S.G.M.Carr & B.Hyland | NC_022414.1 | 108349 | NCBI |
| Heteropyxideae | *Heteropyxis natalensis* Harv. | MK726014.1 | K-MFF s.n. | NCBI |
| Melaleuceae | *Callistemon rigidus* R. Br. | MN794317 | - | NCBI |
| Melaleuceae | *Melaleuca alternifólia* | MN310606) | 617001 | NCBI |
| Myrteae | *Feijoa sellowiana* (O. Berg) O. Berg | KX289887.1 | 01-FLOR0059178 | NCBI |
| Myrteae | *Campomanesia xanthocarpa* (Mart.) O. Berg | KY392760.1 | - | NCBI |
| Myrteae | *Eugenia brasiliensis* Lam. | MN095407 | ICN 202312 | NCBI |
| Myrteae | *Eugenia pyriformis* Cambess. | MN095410 | ICN 202311 | NCBI |
| Myrteae | *Eugenia selloi* B.D.Jacks | MN095411 | ICN 202310 | NCBI |
| Myrteae | *Eugenia uniflora* O.Berg | NC_027744.1 | ICN 193277 | NCBI |
| Myrteae | *Myrcianthes pungens* (O.Berg) D.Legrand | MN095409 | - | NCBI |
| Myrteae | *Pimenta dioica* (L.) Merr. | NC_034684.1 | - | NCBI |
| Myrteae | *Plinia aureana* (Mattos) Mattos | NC_039557.1 | - | NCBI |
| Myrteae | *Plinia cauliflora* (Mart.) Kausel | NC_039395.1 | - | NCBI |
| Myrteae | *Plinia edulis* (Vell.) Sobral | MN095408 | ICN 202315 | NCBI |
| Myrteae | *Plinia trunciflora* (O.Berg) Kausel | NC_034801.1 | - | NCBI |
| Myrteae | *Psidium cattleyanum* Sabine | MN095413 | ICN 202314 | NCBI |
| Myrteae | *Psidium galapageium* Hook.f. | MH491846.1 | 3053515 | NCBI |
| Myrteae | *Psidium guajava* L. | NC_033355.1 | PDBK 2014-0244 | NCBI |
| Syzygieae | *Syzygium cumini* (L.) Skeels | GQ870669.3 | KUH81317 | NCBI |
| Syzygieae | *Syzygium forrestii* Merr. & L.M.Perry | NC_044106.1 | RL0700 | NCBI |
| Syzygieae | *Syzygium jambos* | MT731620 | SCBG-CF-2061 | NCBI |
| - | *Punica granatum* L. | NC_035240.1 | C. Lee 0004 | NCBI |
