# Supplementary material for: Perspectives in Myrtaceae evolution from plastomes and nuclear phylogenies
Source: Genet Mol Biol. 2022 Jan 21;45(1):e20210191. doi: 10.1590/1678-4685-GMB-2021-0191 (PMC8796035; doi:10.1590/1678-4685-GMB-2021-0191)
Supplement: Table S2 - [file 1415-4757-GMB-45-1-e20210191-s2.pdf]

Genetics and Molecular Biology
https://doi.org/10.1590/1678-4685-GMB-2021-0191

Supplementary Material to "Perspectives in Myrtaceae evolution from plastomes and nuclear phylogenies"

Table S2 - Information on the species ITS accessions used for the phylogenetic inference.

Tribe	Species	ITS acession	Source
Eucalypteae	*Angophora floribunda* (Sm.) Sweet	AF190358	NCBI
Eucalypteae	*Corymbia gummifera* (Gaertn.) K.D.Hill & L.A.S.Johnson	AF390463	NCBI
Eucalypteae	*Eucalyptus camaldulensis* Dehnh.	AF190363	NCBI
Eucalypteae	*Eucalyptus diversifolia* Bonpl.	AF058483	NCBI
Eucalypteae	*Eucalyptus globulus* Labill.	HM596049	NCBI
Eucalypteae	*Eucalyptus grandis* W.Hill ex Maiden	AF390472	NCBI
Eucalypteae	*Stockwellia quadrifida* D..Carr, S.G.M.Carr & B.Hyland	AF390445	NCBI
Heteropyxideae	*Heteropyxis natalensis* Harv.	KM064798	NCBI
Myrteae	*Feijoa sellowiana* (O. Berg) O. Berg	KM064915	NCBI
Myrteae	*Campomanesia xanthocarpa* (Mart.) O. Berg	MG708055	NCBI
Myrteae	*Eugenia brasiliensis* Lam.	SRR11746815	NCBI
Myrteae	*Eugenia pyriformis* Cambess.	SRR11745229	NCBI
Myrteae	*Eugenia selloi* B.D.Jacks	SRR11744877	NCBI
Myrteae	*Eugenia uniflora* O.Berg	KM064994.1	NCBI
Myrteae	*Myrcianthes pungens* (O.Berg) D.Legrand	SRR11782077	NCBI
Myrteae	*Pimenta dioica* (L.) Merr.	KM064833	NCBI
Myrteae	*Plinia cauliflora* (Mart.) Kausel	AM234093 (syn. *Myrciaria cauliflora*)	NCBI
Myrteae	*Plinia edulis* (Vell.) Sobral	SRR11786912	NCBI
Myrteae	*Plinia trunciflora* (O.Berg) Kausel	MG708062 (syn. *P. peruviana)*	NCBI
Myrteae	*Psidium cattleyanum* Sabine	SRR11782543	NCBI
Myrteae	*Psidium guajava* L.	AY487283	NCBI
Syzygieae	*Syzygium cumini* (L.) Skeels	KF186456	NCBI
Syzygieae	*Syzygium forrestii* Merr. & L.M.Perry	KR532624	NCBI
-	*Punica granatum* L.	JQ740191	NCBI
