# Supplementary material for: Perspectives in Myrtaceae evolution from plastomes and nuclear phylogenies
Source: Genet Mol Biol. 2022 Jan 21;45(1):e20210191. doi: 10.1590/1678-4685-GMB-2021-0191 (PMC8796035; doi:10.1590/1678-4685-GMB-2021-0191)
Supplement: Table S3 - [file 1415-4757-GMB-45-1-e20210191-s3.pdf]

Genetics and Molecular Biology
https://doi.org/10.1590/1678-4685-GMB-2021-0191

Supplementary Material to “Perspectives in Myrtaceae evolution from plastomes and nuclear phylogenies”

Table S3 - Identification for the nuclear sequences used for the phylogenetic analysis.

Species	Source	Accession	Gene/scaffold accession				
			**MLH1**	**MSH1**	**MCM5**	**SMC1**	**SMC2**
Eucalyptus camaldulensis Dehnh.	NCBI	BADO01000000	BADO01105522.1	BADO01116498.1	BADO01012700.1	BADO01028969.1	BADO01118393.1
Eucalyptus grandis W.Hill ex Maiden	Phytozome	BRASUZ1 v2.0	Eucgr.F02988	Eucgr.H02905	Eucgr.C00405	Eucgr.B01550	Eucgr.A02895
Eucalyptus leucoxylon F.Muell.	1KP	AYMT	AYMT_scaffold_2011512	AYMT_scaffold_2115626	AYMT_scaffold_2007539	AYMT_scaffold_2007947	AYMT_scaffold_2008759
Eucalyptus melliodora A.Cunn. ex Schauer	NCBI	SISH01000000	SISH01000028.1	SISH01000018.1	SISH01000004.1	SISH01000023.1	SISH01000001.1
Eucalyptus pauciflora Siever ex Spreng.	NCBI	VMYD01000000	VMYD01000394.1	VMYD01000040.1	VMYD01000248.1	VMYD01000359.1	VMYD01000256.1
Eugenia brasiliensis Lam.	NCBI	SRR11746815	-	-	-	-	-
Eugenia selloi B.D.Jacks	NCBI	SRR11744877	-	-	-	-	-
Eugenia pyriformis Cambess.	NCBI	SRR11745229	-	-	-	-	-
Eugenia uniflora O.Berg	NCBI	SRR11802646	-	-	-	-	-
Melaleuca quinquenervia (Cav.)S.T.Blake	ENA	PRJNA357284	-	-	-	-	-
Metrosideros polymorpha var. glaberrima (H.Lév.) H.St.John	NCBI	BCNH01000000	BCNH01000091.1	BCNH01000116.1	BCNH01000108.1	BCNH01000109.1	BCNH01000109.1
Myrcianthes pungens (O.Berg) D.Legrand	NCBI	SRR11782077	-	-	-	-	-
Plinia trunciflora (O.Berg) Kausel	NCBI	SRR11815053	-	-	-	-	-
Psidium cattleyanum Sabine	NCBI	SRR11782543	-	-	-	-	-
Psidium guajava L.	NCBI	NTGF01000000	NTGF01000463.1	NTGF01001994.1	NTGF01000078.1	NTGF01002833.1	NTGF01000540.1
Punica granatum L.	NCBI	GCF_007655135.1	XM_031533887.1	XM_031543090.1	XM_031522663.1	XM_031547542.1	XM_031536730.1
Rhodamnia argenteae Benth.	NCBI	GCF_900635035.1	NW_022060796.1	NW_022072000.1	NW_022060926.1	NW_022065603.1	NW_022060838.1

Species	Source	Accession	Gene/scaffold accession				
			MLH1	MSH1	MCM5	SMC1	SMC2
Syzygium cumini (L.) Skeels	NCBI	SRR11747669	-	-	-	-	-
Syzygium micranthum Thwaites	1KP	NEBM	NEBM_scaffold_2018778	NEBM_scaffold_2019471	NEBM_scaffold_2019595	NEBM_scaffold_2005812	NEBM_scaffold_2014640
Syzygium oleosum (F.Muell.) B.Hyland	NCBI	GCF_900635055.1	NW_022076680.1	NW_022076852.1	NW_022076608.1	NW_022076609.1	NW_022078691.1
Syzygium paniculatum Gaertn.	1KP	FGDU	FGDU_scaffold_2012784	FGDU_scaffold_2004874	FGDU_scaffold_2012009	FGDU_scaffold_2061890	FGDU_scaffold_2049802
