# Supplementary material for: Perspectives in Myrtaceae evolution from plastomes and nuclear phylogenies
Source: Genet Mol Biol. 2022 Jan 21;45(1):e20210191. doi: 10.1590/1678-4685-GMB-2021-0191 (PMC8796035; doi:10.1590/1678-4685-GMB-2021-0191)
Supplement: Table S4 - [file 1415-4757-GMB-45-1-e20210191-s4.pdf]

Genetics and Molecular Biology
https://doi.org/10.1590/1678-4685-GMB-2021-0191

Supplementary Material to “Perspectives in Myrtaceae evolution from plastomes and nuclear phylogenies”

Table S4 - Information of BEAST runs.

Dataset	Substitution model	Tree model	Chain length	Sampling	Alignment size
Plastid 78 genes	TVM+I+G4	Yule	200,000,000	5,000	69,354
		FBD	300,000,000	5,000	
Internal transcribed spacer (ITS)	TIM2+I+G4	Yule	200,000,000	5,000	694
Plastid 20 genes	TVM+I+G4	Yule	300,000,000	5,000	42,759
Five nuclear genes	TIM3+I+G4	Yule	200,000,000	5,000	14,799
		FBD	100,000,000	5,000	
MSH1	TIM3+G4	Yule	100,000,000	5,000	3,180
MLH1	HKY+I+G4	Yule	100,000,000	5,000	2,223
SMC1	TIM3+I+G	Yule	100,000,000	5,000	3,660
SMC2	TIM3+I+G	Yule	100,000,000	5,000	3,528
MCM5	TrN+I+G4	Yule	100,000,000	5,000	2,208
accD	GTR+I+G4	Yule	30,000,000	5,000	1494
atpA	TVM+I	Yule	30,000,000	5,000	1524
atpB	GTR+I+G4	Yule	30,000,000	5,000	1497
atpE	TIM3	Yule	30,000,000	5,000	402
atpF	GTR+I	Yule	30,000,000	5,000	570
atpH	HKY+I	Yule	30,000,000	5,000	246
atpI	TPM3uf+I	Yule	30,000,000	5,000	750
ccsA	TVM+G4	Yule	30,000,000	5,000	975
cemA	TPM1uf+I	Yule	30,000,000	5,000	693
clpP	HKY+I	Yule	30,000,000	5,000	591
matK	GTR+G4	Yule	30,000,000	5,000	1572
ndhA	TVM+I+G4	Yule	30,000,000	5,000	1107
ndhB	TPM1uf+I	Yule	30,000,000	5,000	1533
ndhC	TPM3uf	Yule	30,000,000	5,000	363
ndhD	TVM+I+G4	Yule	30,000,000	5,000	1512
ndhE	TVM+I	Yule	30,000,000	5,000	306
ndhF	GTR+I+G4	Yule	30,000,000	5,000	2304
ndhG	TVM+I	Yule	30,000,000	5,000	531
ndhH	GTR+G4	Yule	30,000,000	5,000	1182
ndhI	TIM2+G4	Yule	30,000,000	5,000	522
ndhJ	TVM+G4	Yule	30,000,000	5,000	477
ndhK	TVM+I	Yule	30,000,000	5,000	861

Dataset	Substitution model	Tree model	Chain length	Sampling	Alignment size
petA	TVM+G4	Yule	30,000,000	5,000	963
petB	HKY+I	Yule	30,000,000	5,000	654
petD	TPM2uf+I	Yule	30,000,000	5,000	483
petG	TrN	Yule	30,000,000	5,000	114
petL	F81	Yule	30,000,000	5,000	96
petN	F81	Yule	30,000,000	5,000	96
psaA	GTR+I	Yule	30,000,000	5,000	2253
psaB	TPM1uf+I	Yule	30,000,000	5,000	2205
psaC	TPM1uf+I	Yule	30,000,000	5,000	246
psaI	TIM1+G4	Yule	30,000,000	5,000	114
psaJ	TPM2uf	Yule	30,000,000	5,000	135
psbA	TIM1+I	Yule	30,000,000	5,000	1062
psbB	TPM1uf+I+G4	Yule	30,000,000	5,000	1527
psbC	TVM+I+G4	Yule	30,000,000	5,000	1422
psbD	TPM1uf+I	Yule	30,000,000	5,000	1062
psbE	TIM2	Yule	30,000,000	5,000	252
psbF	TPM3	Yule	30,000,000	5,000	120
psbH	HKY+I	Yule	30,000,000	5,000	222
psbI	TPM2uf	Yule	30,000,000	5,000	111
psbJ	TPM1uf+I	Yule	30,000,000	5,000	123
psbK	TPM3uf	Yule	30,000,000	5,000	186
psbL	HKY	Yule	30,000,000	5,000	117
psbM	TPM2uf	Yule	30,000,000	5,000	105
psbN	K80	Yule	30,000,000	5,000	132
psbT	F81	Yule	30,000,000	5,000	117
psbZ	TPM1uf	Yule	30,000,000	5,000	189
rbcL	TVM+I+G4	Yule	30,000,000	5,000	1480
rpl14	TPM3uf+G4	Yule	30,000,000	5,000	369
rpl16	TPM3uf+G4	Yule	30,000,000	5,000	423
rpl2	TPM1uf+I	Yule	30,000,000	5,000	828
rpl20	TPM1uf+I	Yule	30,000,000	5,000	369
rpl22	TPM1uf+G4	Yule	30,000,000	5,000	513
rpl23	TPM3uf+I	Yule	30,000,000	5,000	282
rpl32	TPM3uf+I	Yule	30,000,000	5,000	174
rpl33	TPM3uf	Yule	30,000,000	5,000	207
rpl36	TPM1uf+G4	Yule	30,000,000	5,000	114
rpoA	TVM+I+G4	Yule	30,000,000	5,000	1014
rpoB	TVM+I+G4	Yule	30,000,000	5,000	3222
rpoC1	GTR+G4	Yule	30,000,000	5,000	2070

Dataset	Substitution model	Tree model	Chain length	Sampling	Alignment size
rpoC2	GTR+I+G4	Yule	30,000,000	5,000	4229
rps11	TVM+I	Yule	30,000,000	5,000	417
rps12	TPM1uf	Yule	30,000,000	5,000	402
rps14	TPM2uf+I	Yule	30,000,000	5,000	303
rps15	TVM+I	Yule	30,000,000	5,000	276
rps16	TPM3uf+G4	Yule	30,000,000	5,000	267
rps18	TPM1uf	Yule	30,000,000	5,000	306
rps19	TVM+G4	Yule	30,000,000	5,000	279
rps2	GTR+G4	Yule	30,000,000	5,000	711
rps3	TVM+I+G4	Yule	30,000,000	5,000	657
rps4	TPM1uf	Yule	30,000,000	5,000	606
rps7	HKY	Yule	30,000,000	5,000	468
rps8	TPM3uf+I	Yule	30,000,000	5,000	405
ycf1	TVM+I+G4	Yule	30,000,000	5,000	5808
ycf2	GTR+I	Yule	30,000,000	5,000	6969
ycf3	TPM1uf+I	Yule	30,000,000	5,000	513
ycf4	TVM+I	Yule	30,000,000	5,000	555
