# Supplementary material for: Perspectives in Myrtaceae evolution from plastomes and nuclear phylogenies
Source: Genet Mol Biol. 2022 Jan 21;45(1):e20210191. doi: 10.1590/1678-4685-GMB-2021-0191 (PMC8796035; doi:10.1590/1678-4685-GMB-2021-0191)
Supplement: Table S5 - [file 1415-4757-GMB-45-1-e20210191-s5.pdf]

Genetics and Molecular Biology
https://doi.org/10.1590/1678-4685-GMB-2021-0191

Supplementary Material to "Perspectives in Myrtaceae evolution from plastomes and nuclear phylogenies"

Table S5 - Details of fossils data used for time divergence estimation.

Fossil	Calibration node	Age	Fossil location	Reference	Prior distribution
Myrtales crown age estimate	Root node calibration (A)	91 Ma	n/a	Wang et al., 2009	Normal Mean - 91 st.dev. - 2.5
Myrtaceidites mesonesu Cookson & Pike	Myrtoideaeeae crown (B)	Paleocene	New Zealand	Couper, 1960	Exponential Mean - 1.03 Offset - 61.7
Eucalyptus fruits	Eucalypteae crown (C)	Early Eocene	Patagonia, South America	Gandolfo et al., 2011	Exponential Mean - 1.844 Offset - 51.2
Myrtaceidites eugeniioides Cookson & Pike	Syzygieae crown (D)	Early Eocene	Princetown, Victoria	Harris, 1965	Exponential Mean - 1.952 Offset - 48.6
Paleomyrtinaea princetonensis Stockey & Maxwell	Myrteae crown (E)	Early Eocene	United States of America	Pigg et al., 1993	Exponential Mean - 4.066 Offset - 50.0
Myrtaceidites verrucosus	South American Myrteae crown (F)	Middle Eocene	Panama and Argentina	Graham, 1985; Romero & Zamaloa, 1985	Exponential Mean - 3.09 Offset - 37.2
Myrtaceidites eucalyptoides Cookson & Pike	*Eucalyptus* crown (G)	Late Eocene	Lake Eyre and Otway Basins, South Australia	Alley et al., 1996	Exponential Mean - 0.895 Offset - 33.9
Myrtaceidites tenuis Harris	Angophora + Corymbia crown (H)	Early Eocene	Bass Strait, Tasmania	Thornhill & Macphail, 2012	Exponential Mean - 2.17 Offset - 45.0
Myrtaceidites verrucosus Stover & Partridge	Australasian Myrteae crown (*Rhodomyrtus tomentosa* and *Rhodamnia argentea*)	Late Eocene	Hatfield, New South Wales	Thornhill & Macphail, 2012	Exponential Mean - 0.895 Offset - 33.9
Metrosideros leaves and fruits	Metrosideros crown (*Metrosideros polymorpha*)	Miocene	New Zealand	Pole et al., 2008	Exponential Mean - 2.982 Offset - 20.0

Alley NF, Krieg GW and Callen RA (1996) Early Tertiary Eyre formation, lower Nelly Creek, southern Lake Eyre Basin, Australia: Palynological dating of macrofloras and silcrete, and palaeoclimatic interpretations. Australian Journal of Earth Sciences 43:71–84.

Couper RA (1960) New Zealand Mesozoic and Cainozoic Plant Microfossils. New Zealand Department of Scientific and Industrial Research.

Nixon KC, González CC, Wilf P, Cúneo NR and Johnson KR (2011) Oldest Known Eucalyptus Macrofossils Are from South America. PLoS ONE 6:e21084.

Graham A (1985) Studies in Neotropical Paleobotany IV. The Eocene communities of Panama. Annals of the Missouri Botanical Garden 72:504–534.

Harris WK (1965) Basal Tertiary microfloras from the Princetown area, Victoria, Australia. Palaeontographica B 115:75–106.

Pigg KB, Stockey RA and Maxwell SL (1993) Paleomyrtinaea, a new genus of premineralized Myrtaceous fruits and seeds from the Eocene of British-Columbia and Paleocene of North-Dakota. Canadian Journal of Botany 71:1–9.

Pole M, Dawson J and Denton T (2008) Fossil Myrtaceae from the Early Miocene of southern New Zealand. Australian Journal of Botany 56:67–81.

Thornhill AH and Macphail MK (2012) Fossil myrtaceous pollen as evidence for the evolutionary history of the Myrtaceae: A review of fossil Myrtaceidites species. Review of Palaeobotany and Palynology 176–177:1–23.

Wang H, Moore MJ, Soltis PS, Bell CD, Brockington SF, Alexandre R, Davis CC, Latvis M, Manchester SR and Soltis DE (2009) Rosid radiation and the rapid rise of angiosperm-dominated forests. Proceedings of the National Academy of Sciences of the United States of America 106:3853–3858.
