# Supplementary material for: Perspectives in Myrtaceae evolution from plastomes and nuclear phylogenies
Source: Genet Mol Biol. 2022 Jan 21;45(1):e20210191. doi: 10.1590/1678-4685-GMB-2021-0191 (PMC8796035; doi:10.1590/1678-4685-GMB-2021-0191)
Supplement: Table S6 - [file 1415-4757-GMB-45-1-e20210191-s6.pdf]

Genetics and Molecular Biology
https://doi.org/10.1590/1678-4685-GMB-2021-0191

Supplementary Material to “Perspectives in Myrtaceae evolution from plastomes and nuclear phylogenies”

Table S6 - Information on the variation level of each plastid gene.

Gene	Shorter sequence	Longer sequence	Alignment size	Length variation	Parsim-info sites	Parsim-info sites/total	Variable sites	Variable sites/total	Conserved sites	Conserved sites/total	Singleton	0-fold	2-fold	4-fold	Rate mean
accD	1461	1479	1494	18	67	0.045	189	0.127	1290	0.863	122	953	319	137	1.0954
atpA	1518	1524	1524	6	35	0.023	129	0.085	1395	0.915	94	974	295	236	1.177
atpB	1494	1497	1497	3	42	0.028	115	0.077	1382	0.923	73	954	265	251	1.0454
atpE	399	402	402	3	4	0.010	32	0.080	370	0.920	28	254	86	53	1.0666
atpF	555	570	570	15	20	0.035	55	0.096	515	0.904	35	351	133	60	1.0987
atpH	246	246	246	0	4	0.016	18	0.073	228	0.927	14	156	38	50	1.0112
atpI	744	750	750	6	22	0.029	54	0.072	696	0.928	32	485	145	110	1.0403
ccsA	960	975	975	15	51	0.052	144	0.148	816	0.837	93	620	189	98	1.0561
cemA	684	693	693	9	23	0.033	70	0.101	620	0.895	47	445	150	67	1.0475
clpP	588	591	591	3	14	0.024	44	0.074	547	0.926	30	386	106	85	1.1731
matK	1479	1557	1572	78	106	0.067	322	0.205	1205	0.767	215	961	328	140	1.0264
ndhA	1062	1104	1107	42	46	0.042	126	0.114	969	0.875	80	692	219	149	1.0407
ndhB	1533	1533	1533	0	10	0.007	22	0.014	1511	0.986	12	989	301	231	1.0125
ndhC	363	363	363	0	3	0.008	35	0.096	328	0.904	32	237	67	43	1.2129
ndhD	1503	1512	1512	9	69	0.046	204	0.135	1299	0.859	135	951	293	186	1.0521
ndhE	306	306	306	0	11	0.036	30	0.098	276	0.902	19	194	67	39	1.0617
ndhF	2199	2292	2304	93	150	0.065	410	0.178	1867	0.810	255	1442	454	250	1.023
ndhG	531	531	531	0	21	0.040	61	0.115	470	0.885	40	338	100	68	1.1712
ndhH	1182	1182	1182	0	35	0.030	112	0.095	1070	0.905	77	761	257	139	1.0208
ndhI	486	522	522	36	25	0.048	57	0.109	450	0.862	31	336	110	61	1.0821

Gene	Shorter sequence	Longer sequence	Alignment size	Length variation	Parsim-info sites	Parsim-info sites/total	Variable sites	Variable sites/total	Conserved sites	Conserved sites/total	Singleton	0-fold	2-fold	4-fold	Rate mean
ndhJ	477	477	477	0	11	0.023	34	0.071	443	0.929	23	308	110	59	1.1115
ndhK	678	861	861	183	18	0.021	68	0.079	790	0.918	50	559	169	117	1.0297
petA	963	963	963	0	36	0.037	96	0.100	867	0.900	60	621	182	139	1.0627
petB	648	654	654	6	13	0.020	44	0.067	604	0.924	31	433	111	107	1.0349
petD	480	483	483	3	13	0.027	31	0.064	452	0.936	18	308	92	78	1.0111
petG	114	114	114	0	1	0.009	3	0.026	111	0.974	2	70	28	15	1.001
petL	96	96	96	0	1	0.010	7	0.073	89	0.927	6	60	17	17	1.0269
petN	90	96	96	6	1	0.010	6	0.063	90	0.938	5	66	12	16	1.0074
psaA	2253	2253	2253	0	38	0.017	124	0.055	2129	0.945	86	1466	425	343	1.01
psaB	2205	2205	2205	0	47	0.021	134	0.061	2071	0.939	87	1432	441	311	1.0769
psaC	246	246	246	0	19	0.077	227	0.923	10	0.041	10	161	49	35	1.0097
psaI	105	114	114	9	8	0.070	14	0.123	100	0.877	6	75	17	14	1.1189
psaJ	135	135	135	0	3	0.022	7	0.052	128	0.948	4	83	26	23	1.0015
psbA	1062	1062	1062	0	17	0.016	68	0.064	994	0.936	51	701	187	162	1.0209
psbB	1527	1527	1527	0	38	0.025	116	0.076	1411	0.924	78	999	272	243	1.1691
psbC	1386	1422	1422	36	26	0.018	86	0.060	1336	0.940	60	916	254	238	1.1106
psbD	1062	1062	1062	0	16	0.015	45	0.042	1017	0.958	29	693	192	170	1.1494
psbE	252	252	252	0	2	0.008	11	0.044	241	0.956	9	164	53	35	1.0812
psbF	120	120	120	0	1	0.008	5	0.042	115	0.958	4	78	20	21	1.0139
psbH	222	222	222	0	5	0.023	16	0.072	206	0.928	11	142	40	37	1.1245
psbI	111	111	111	0	2	0.018	8	0.072	103	0.928	6	74	16	20	1.0991
psbJ	123	123	123	0	6	0.049	8	0.065	115	0.935	2	79	18	21	0.9983
psbK	180	186	186	6	5	0.027	22	0.118	164	0.882	17	122	37	23	1.016
psbL	117	117	117	0	3	0.026	6	0.051	111	0.949	3	75	26	14	1.0004
psbM	105	105	105	0	0	0.000	6	0.057	99	0.943	6	70	18	16	1.0458

Gene	Shorter sequence	Longer sequence	Alignment size	Length variation	Parsim-info sites	Parsim-info sites/total	Variable sites	Variable sites/total	Conserved sites	Conserved sites/total	Singleton	0-fold	2-fold	4-fold	Rate mean
psbN	132	132	132	0	1	0.008	3	0.023	129	0.977	2	85	25	21	1.0025
psbT	108	117	117	9	0	0.000	5	0.043	103	0.880	5	76	24	16	1.0094
psbZ	189	189	189	0	2	0.011	11	0.058	178	0.942	9	122	31	31	1.0077
rbcL	1428	1459	1480	31	61	0.041	133	0.090	1326	0.896	72	951	264	226	0.9403
rpl14	369	369	369	0	11	0.030	30	0.081	339	0.919	19	232	79	51	1.0876
rpl16	408	417	423	9	15	0.035	44	0.104	364	0.861	29	275	65	64	1.0214
rpl2	351	369	828	18	11	0.013	37	0.045	791	0.955	26	526	169	123	1.0605
rpl20	396	489	369	93	17	0.046	41	0.111	238	0.645	24	218	92	38	1.0198
rpl22	282	282	513	0	58	0.113	116	0.226	385	0.750	56	322	108	46	0.9896
rpl23	825	828	282	3	4	0.014	8	0.028	274	0.972	4	182	56	38	1.0019
rpl32	153	174	174	21	27	0.155	42	0.241	132	0.759	15	110	26	23	1.0277
rpl33	201	207	207	6	5	0.024	31	0.150	176	0.850	22	128	44	21	1.1888
rpl36	114	114	114	0	5	0.044	15	0.132	99	0.868	10	69	29	11	1.0035
rpoA	1014	1014	1014	0	2	0.002	117	0.115	897	0.885	85	633	238	100	1.0189
rpoB	3213	3222	3222	9	79	0.025	260	0.081	2962	0.919	181	2018	700	412	1.022
rpoC1	2040	2070	2070	30	36	0.017	153	0.074	1917	0.926	117	1301	458	253	1.1318
rpoC2	4116	4220	4229	104	162	0.038	528	0.125	3680	0.870	366	2634	886	490	1.1036
rps11	417	417	417	0	13	0.031	38	0.091	379	0.909	25	264	67	75	1.0161
rps12	351	390	402	39	71	0.177	76	0.189	326	0.811	5	245	67	60	1.4579
rps14	303	303	303	0	6	0.020	29	0.096	274	0.904	23	187	69	35	1.0161
rps15	255	276	276	21	21	0.076	39	0.141	237	0.859	18	165	65	30	1.0138
rps16	234	267	267	33	17	0.064	58	0.217	194	0.727	41	1665	47	33	1.1365
rps18	306	306	306	0	2	0.007	23	0.075	283	0.925	21	182	81	31	1.0732
rps19	279	279	279	0	9	0.032	37	0.133	242	0.867	28	180	56	34	1.1134
rps2	711	711	711	0	16	0.023	59	0.083	652	0.917	43	449	154	90	1.1185

Gene	Shorter sequence	Longer sequence	Alignment size	Length variation	Parsim-info sites	Parsim-info sites/total	Variable sites	Variable sites/total	Conserved sites	Conserved sites/total	Singleton	0-fold	2-fold	4-fold	Rate mean
rps3	651	657	657	6	42	0.064	96	0.146	555	0.845	54	412	140	70	1.0436
rps4	606	606	606	0	15	0.025	61	0.101	545	0.899	46	380	128	75	1.0802
rps7	468	468	468	0	0	0.000	5	0.011	463	0.989	5	301	91	76	0.9997
rps8	405	405	405	0	16	0.040	51	0.126	354	0.874	35	246	93	48	1.1463
ycf1	3144	5661	5808	2517	539	0.093	1325	0.228	4354	0.750	785	3574	1247	374	1.0181
ycf2	6825	6921	6969	96	79	0.011	277	0.040	6629	0.951	197	4420	1617	790	1.0732
ycf3	507	513	513	6	15	0.029	25	0.049	482	0.940	10	331	117	61	1.042
ycf4	555	555	555	0	28	0.050	64	0.115	491	0.885	36	358	105	68	1.0502
