# Supplementary material for: Perspectives in Myrtaceae evolution from plastomes and nuclear phylogenies
Source: Genet Mol Biol. 2022 Jan 21;45(1):e20210191. doi: 10.1590/1678-4685-GMB-2021-0191 (PMC8796035; doi:10.1590/1678-4685-GMB-2021-0191)
Supplement: Figure S3 - [file 1415-4757-GMB-45-1-e20210191-s9.pdf]

Genetics and Molecular Biology
https://doi.org/10.1590/1678-4685-GMB-2021-0191

Supplementary Material to "Perspectives in Myrtaceae evolution from plastomes and nuclear phylogenies"

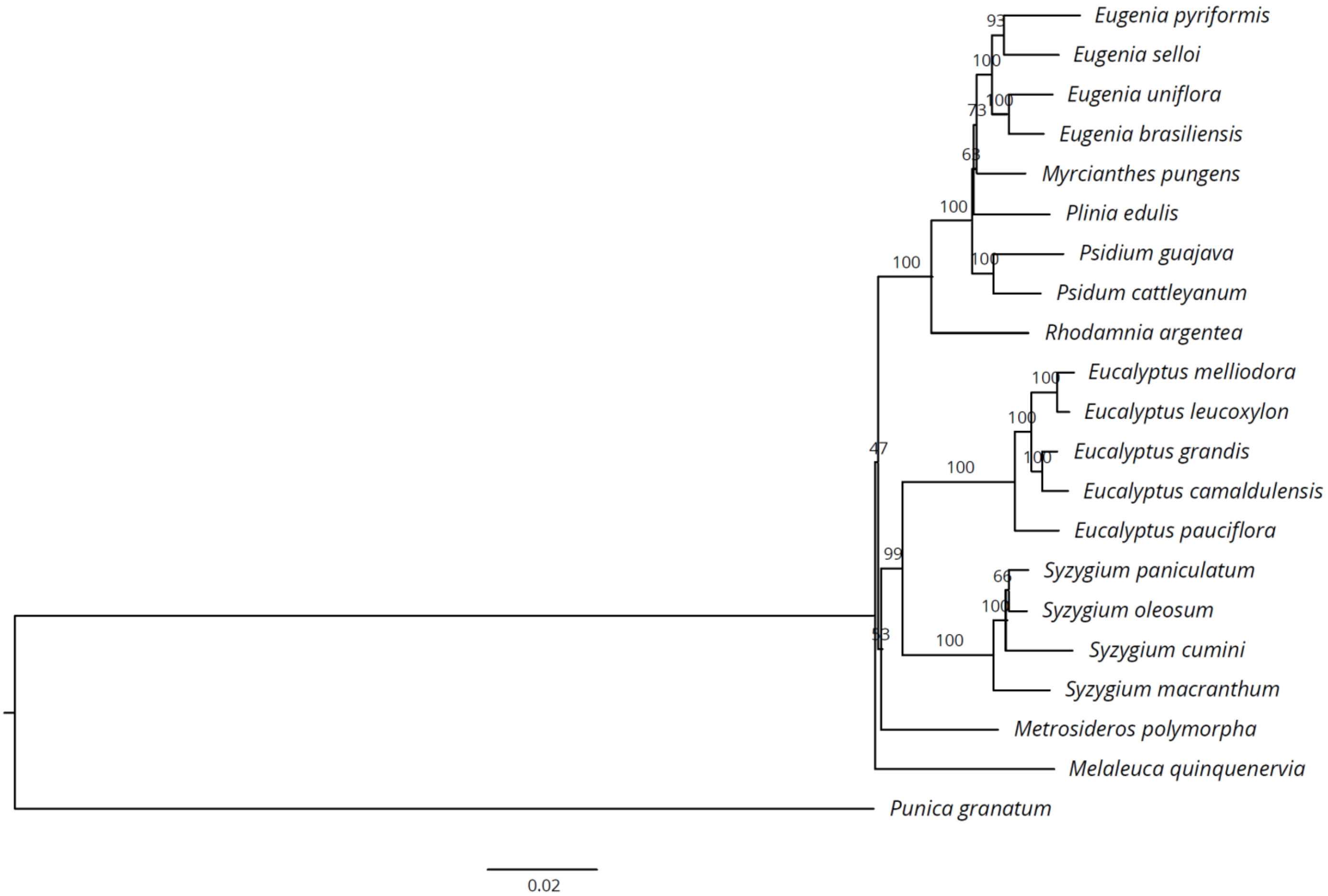

Figure S3 - Maximum-likelihood tree inferred from the five nuclear protein coding markers. Bootstrap values for each node are indicated and branch lengths are scaled according to number of substitutions per site.
