# Supplementary material for: Perspectives in Myrtaceae evolution from plastomes and nuclear phylogenies
Source: Genet Mol Biol. 2022 Jan 21;45(1):e20210191. doi: 10.1590/1678-4685-GMB-2021-0191 (PMC8796035; doi:10.1590/1678-4685-GMB-2021-0191)
Supplement: Figure S4 - [file 1415-4757-GMB-45-1-e20210191-s10.pdf]

Genetics and Molecular Biology
https://doi.org/10.1590/1678-4685-GMB-2021-0191

**Supplementary Material to "Perspectives in Myrtaceae evolution from plastomes and nuclear phylogenies"**

# *accD*

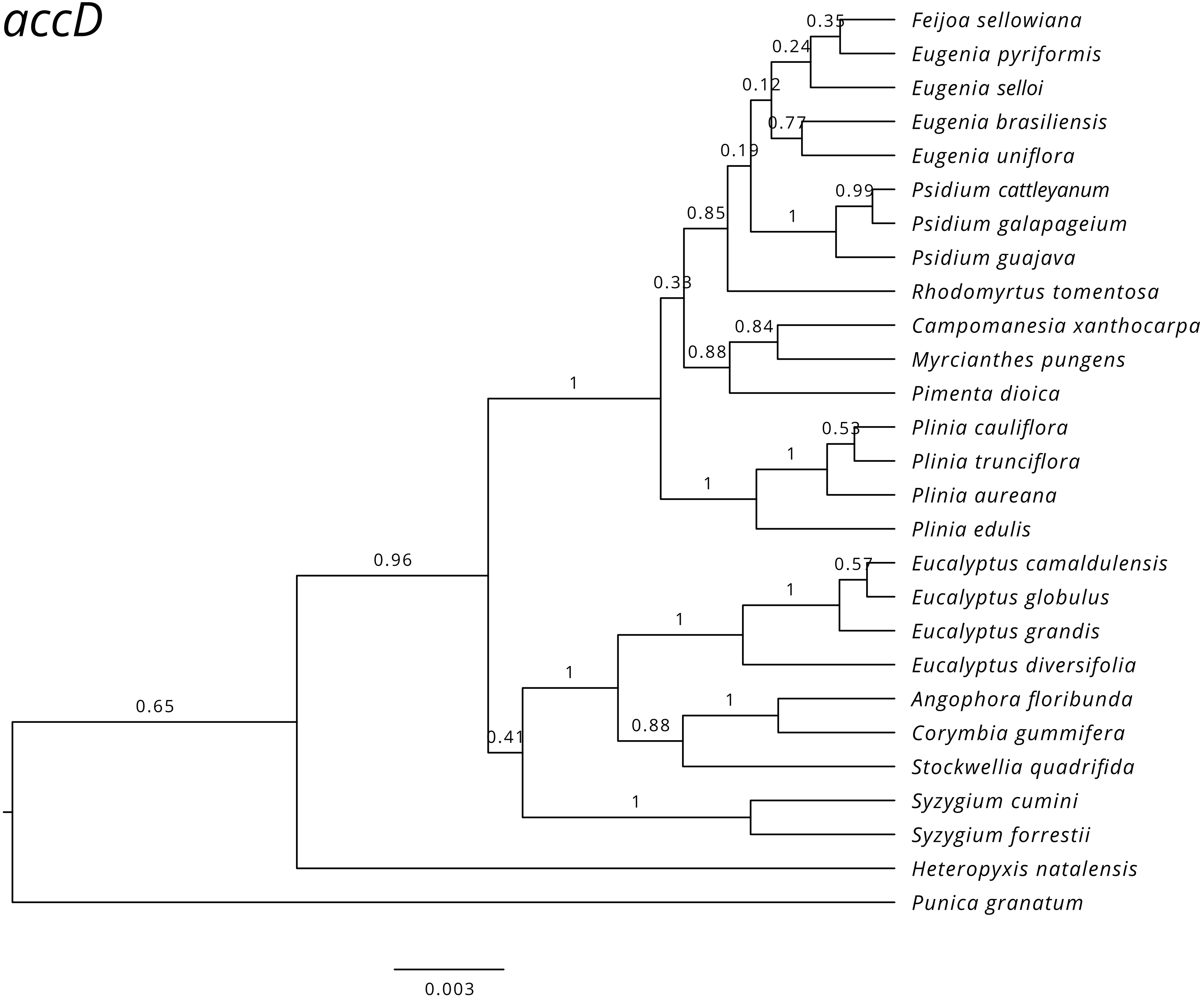

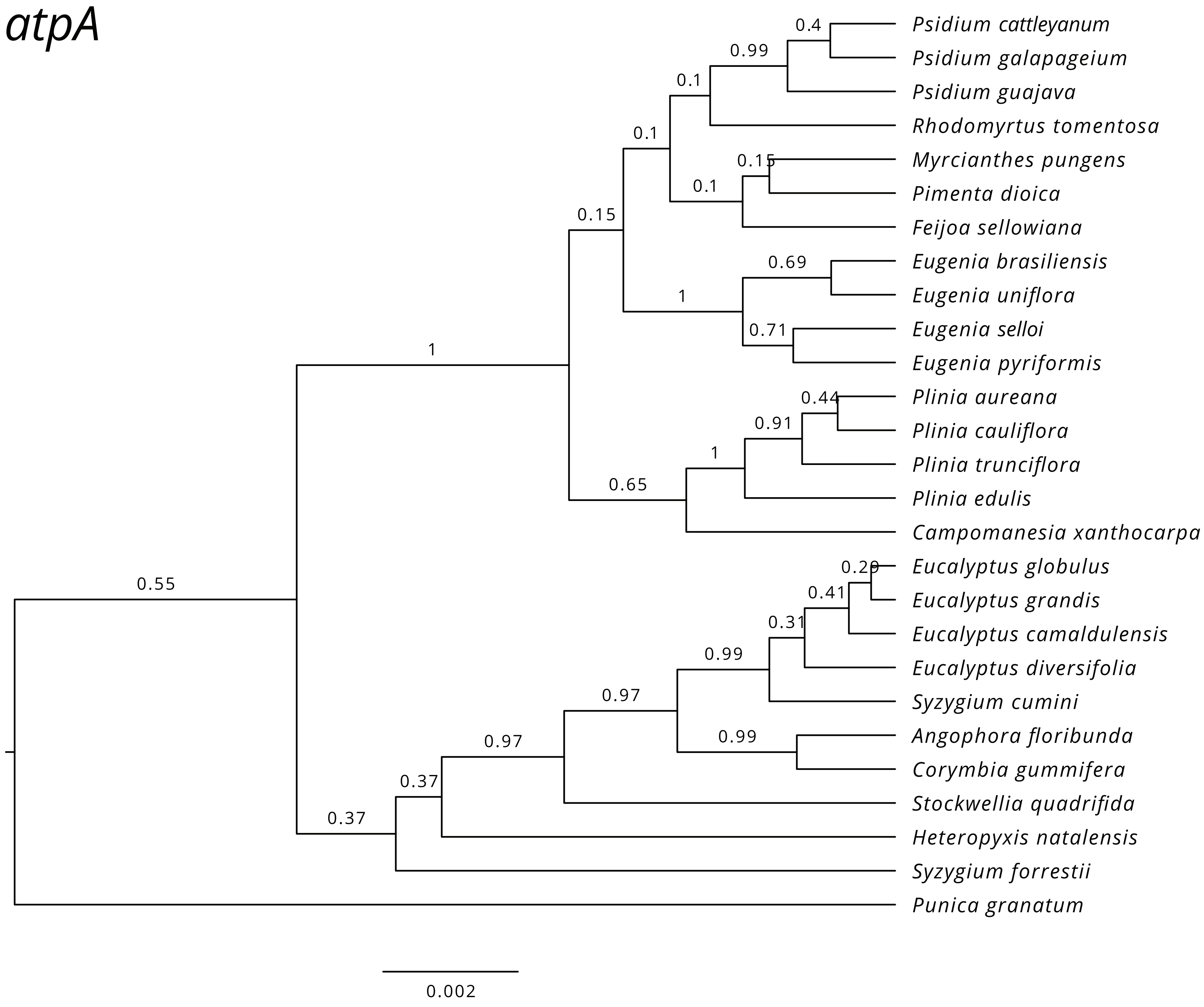
atpA
0.4
0.99
0.1
0.1
0.15
0.1
0.15
0.69
1
0.71
1
0.44
0.91
1
0.65
0.55
0.29
0.41
0.31
0.99
0.97
0.97
0.99
0.37
0.37
Psidium cattleyanum
Psidium galapageium
Psidium guajava
Rhodomyrtus tomentosa
Myrcianthes pungens
Pimenta dioica
Feijoa sellowiana
Eugenia brasiliensis
Eugenia uniflora
Eugenia selloi
Eugenia pyriformis
Plinia aureana
Plinia cauliflora
Plinia trunciflora
Plinia edulis
Campomanesia xanthocarpa
Eucalyptus globulus
Eucalyptus grandis
Eucalyptus camaldulensis
Eucalyptus diversifolia
Syzygium cumini
Angophora floribunda
Corymbia gummifera
Stockwellia quadrifida
Heteropyxis natalensis
Syzygium forrestii
Punica granatum
0.002

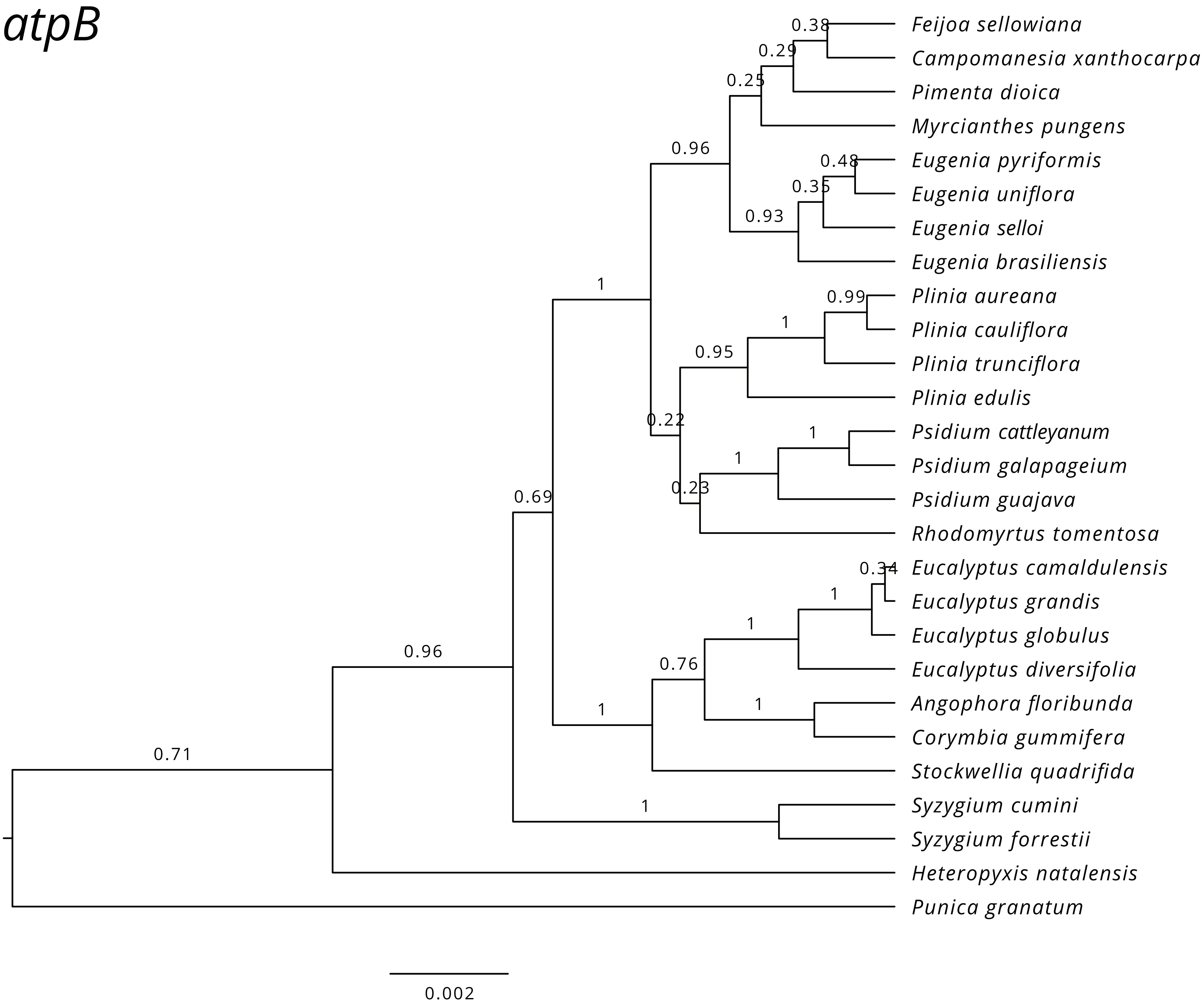
atpB
Feijoa sellowiana
Campomanesia xanthocarpa
Pimenta dioica
Myrcianthes pungens
Eugenia pyriformis
Eugenia uniflora
Eugenia selloi
Eugenia brasiliensis
Plinia aureana
Plinia cauliflora
Plinia trunciflora
Plinia edulis
Psidium cattleyanum
Psidium galapageium
Psidium guajava
Rhodomyrtus tomentosa
Eucalyptus camaldulensis
Eucalyptus grandis
Eucalyptus globulus
Eucalyptus diversifolia
Angophora floribunda
Corymbia gummifera
Stockwellia quadrifida
Syzygium cumini
Syzygium forrestii
Heteropyxis natalensis
Punica granatum
0.38
0.29
0.25
0.96
0.48
0.35
0.93
1
0.99
1
0.95
0.22
1
1
0.23
0.69
0.34
1
1
0.76
1
1
0.96
1
0.71
0.002

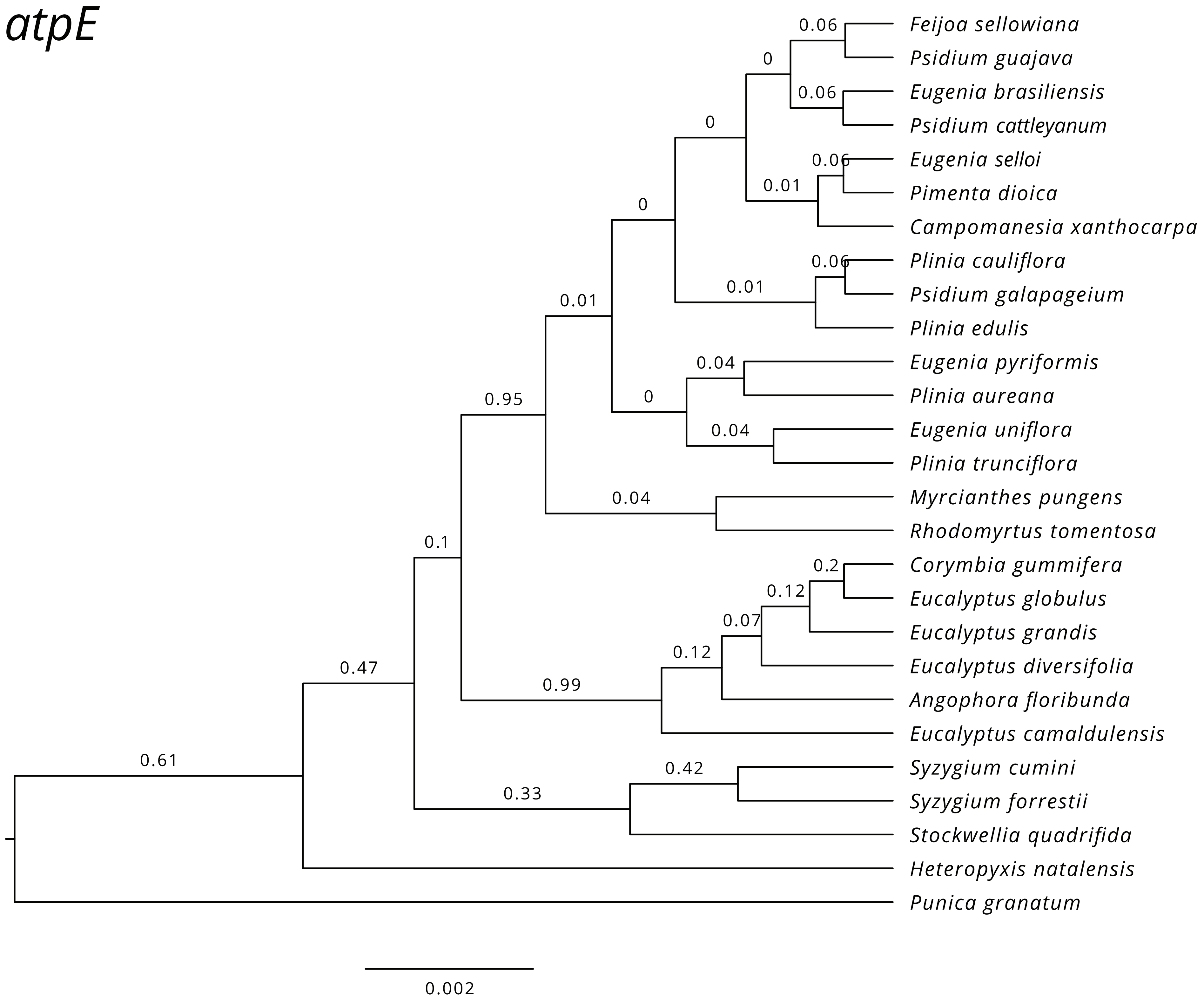

atpE
0.06
0
0.06
0
0.06
0.01
0
0.06
0.01
0.01
0.04
0
0.04
0.95
0.04
0.1
0.2
0.12
0.07
0.12
0.47
0.99
0.61
0.42
0.33
Feijoa sellowiana
Psidium guajava
Eugenia brasiliensis
Psidium cattleyanum
Eugenia selloi
Pimenta dioica
Campomanesia xanthocarpa
Plinia cauliflora
Psidium galapageium
Plinia edulis
Eugenia pyriformis
Plinia aureana
Eugenia uniflora
Plinia trunciflora
Myrcianthes pungens
Rhodomyrtus tomentosa
Corymbia gummifera
Eucalyptus globulus
Eucalyptus grandis
Eucalyptus diversifolia
Angophora floribunda
Eucalyptus camaldulensis
Syzygium cumini
Syzygium forrestii
Stockwellia quadrifida
Heteropyxis natalensis
Punica granatum
0.002

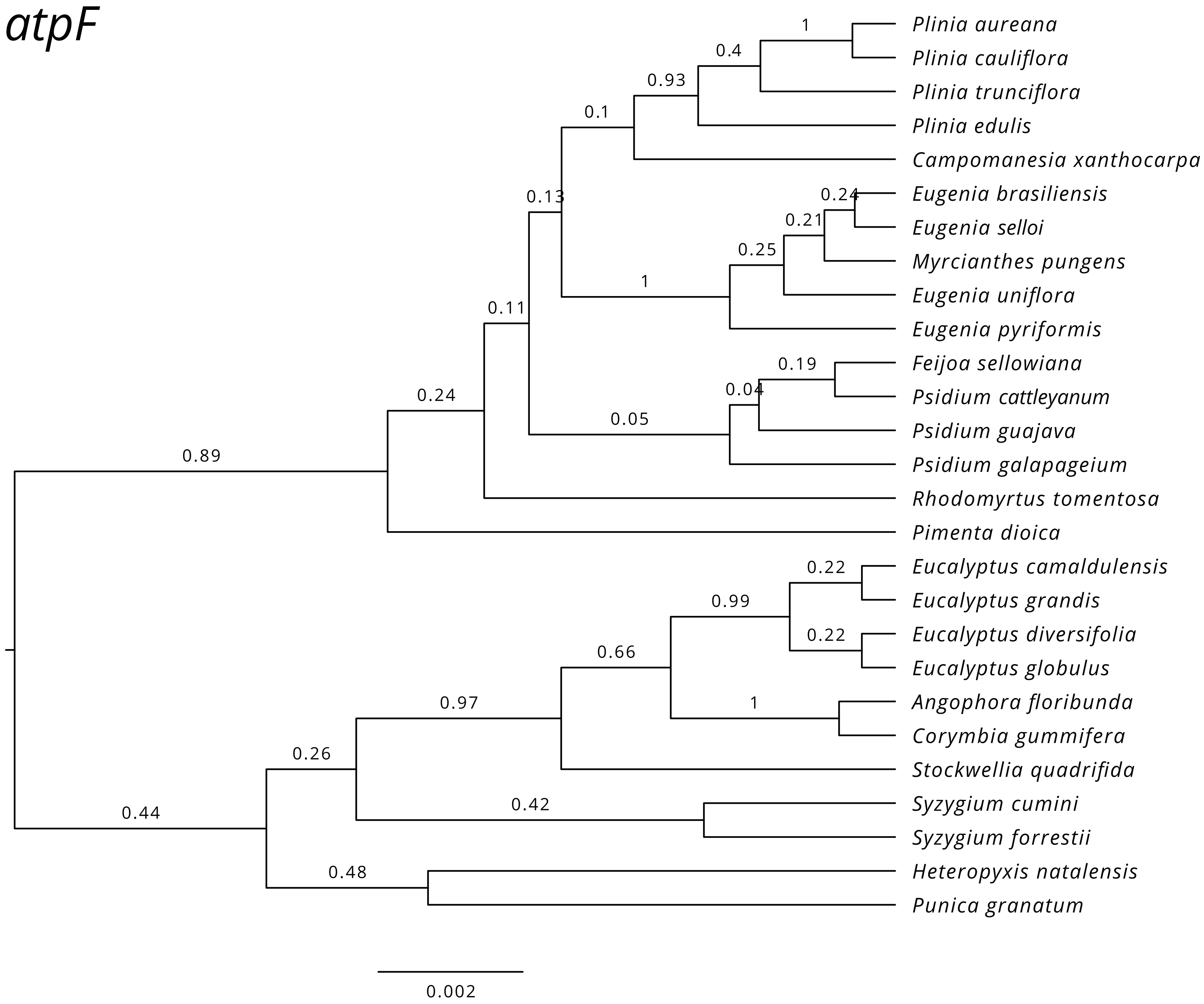
atpF
Plinia aureana
Plinia cauliflora
Plinia trunciflora
Plinia edulis
Campomanesia xanthocarpa
Eugenia brasiliensis
Eugenia selloi
Myrcianthes pungens
Eugenia uniflora
Eugenia pyriformis
Feijoa sellowiana
Psidium cattleyanum
Psidium guajava
Psidium galapageium
Rhodomyrtus tomentosa
Pimenta dioica
Eucalyptus camaldulensis
Eucalyptus grandis
Eucalyptus diversifolia
Eucalyptus globulus
Angophora floribunda
Corymbia gummifera
Stockwellia quadrifida
Syzygium cumini
Syzygium forrestii
Heteropyxis natalensis
Punica granatum
1
0.4
0.93
0.1
0.24
0.21
0.25
0.13
1
0.11
0.19
0.04
0.05
0.24
0.89
0.22
0.99
0.22
0.66
1
0.97
0.26
0.42
0.44
0.48
0.002

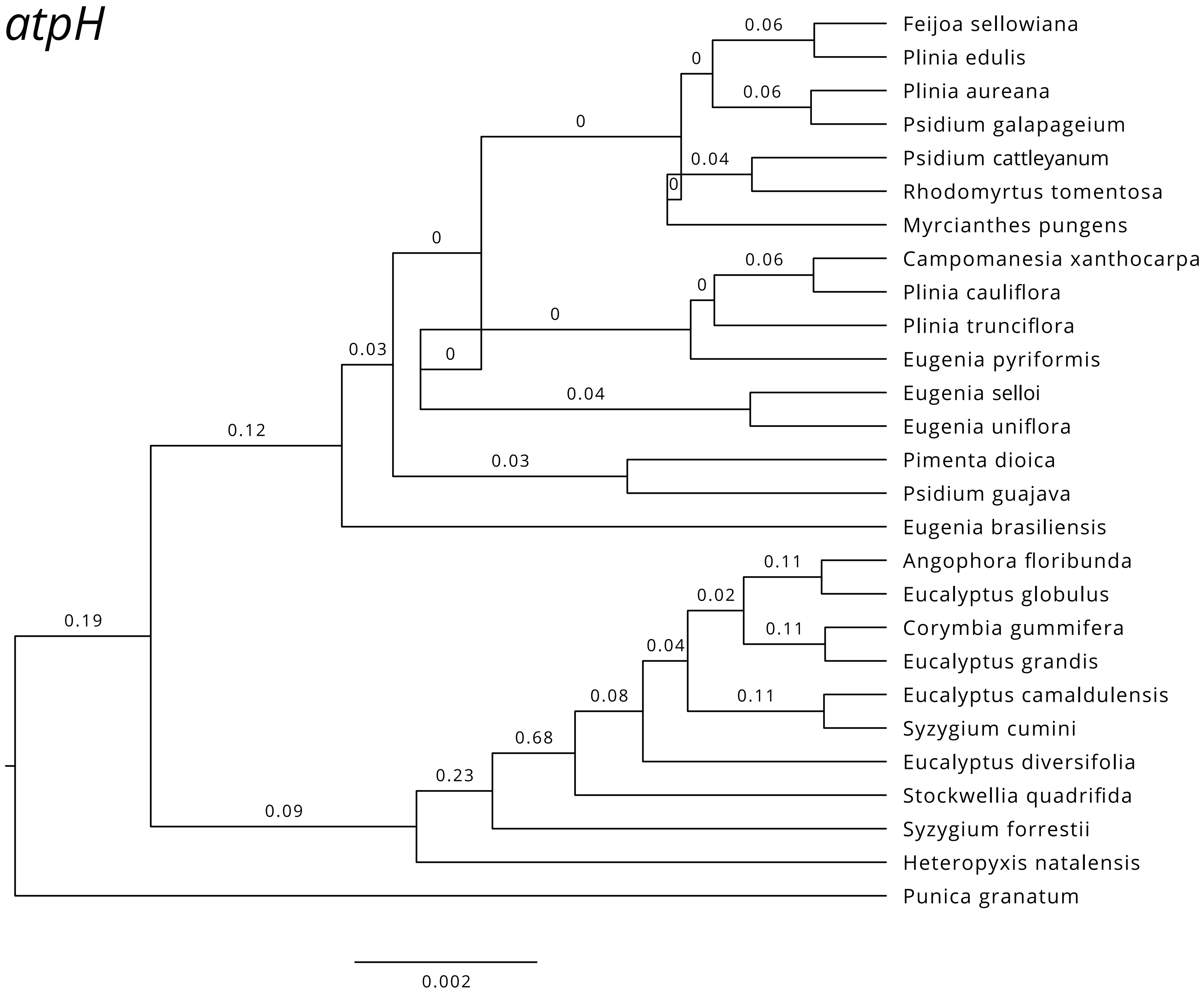
atpH
Feijoa sellowiana
Plinia edulis
Plinia aureana
Psidium galapageium
Psidium cattleyanum
Rhodomyrtus tomentosa
Myrcianthes pungens
Campomanesia xanthocarpa
Plinia cauliflora
Plinia trunciflora
Eugenia pyriformis
Eugenia selloi
Eugenia uniflora
Pimenta dioica
Psidium guajava
Eugenia brasiliensis
Angophora floribunda
Eucalyptus globulus
Corymbia gummifera
Eucalyptus grandis
Eucalyptus camaldulensis
Syzygium cumini
Eucalyptus diversifolia
Stockwellia quadrifida
Syzygium forrestii
Heteropyxis natalensis
Punica granatum
0.06
0
0.06
0
0.04
0
0
0.06
0
0
0.03
0
0.04
0.12
0.03
0.11
0.02
0.11
0.04
0.08
0.11
0.68
0.23
0.19
0.09
0.002

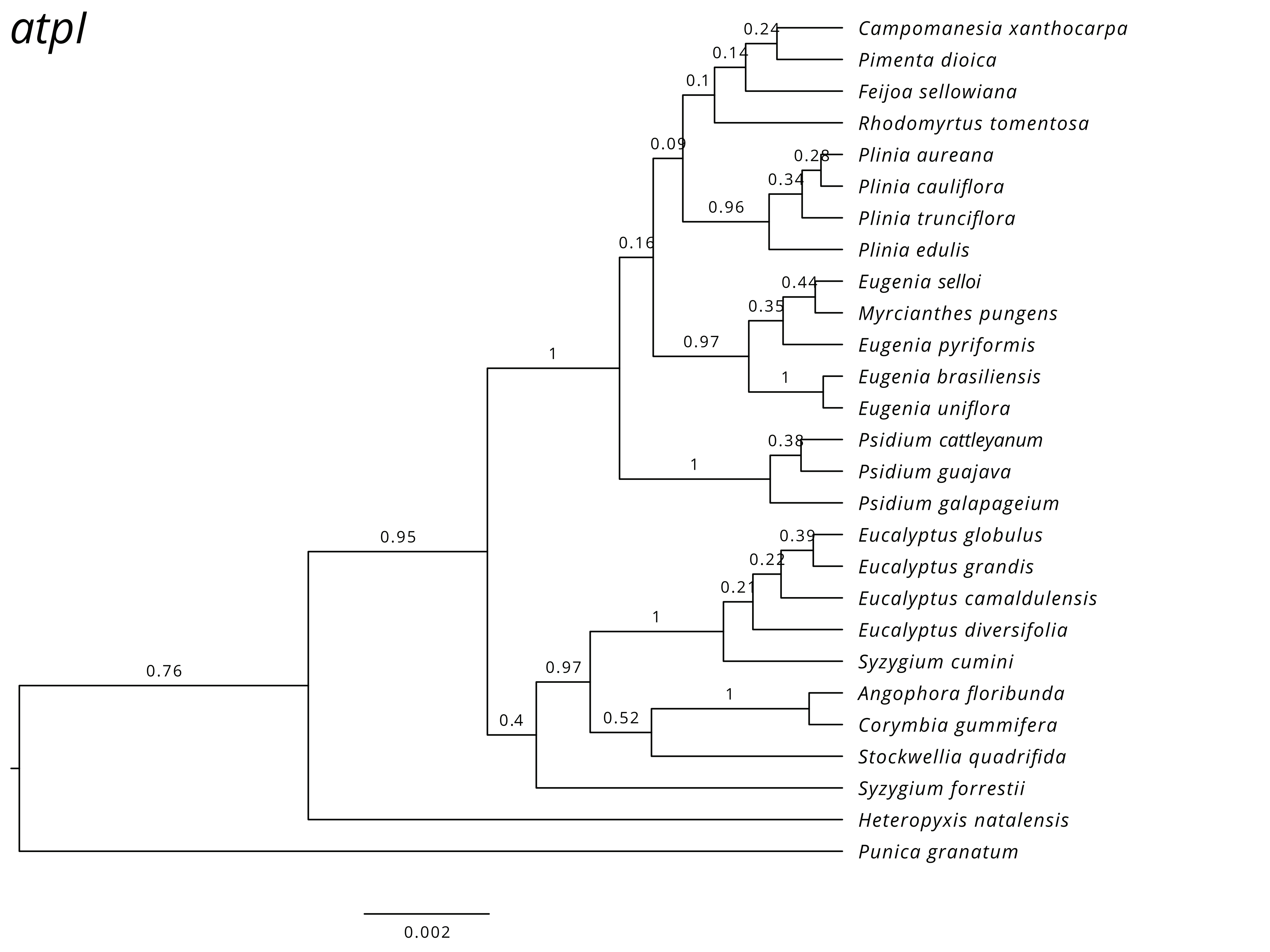
atpI
Campomanesia xanthocarpa
Pimenta dioica
Feijoa sellowiana
Rhodomyrtus tomentosa
Plinia aureana
Plinia cauliflora
Plinia trunciflora
Plinia edulis
Eugenia selloi
Myrcianthes pungens
Eugenia pyriformis
Eugenia brasiliensis
Eugenia uniflora
Psidium cattleyanum
Psidium guajava
Psidium galapageium
Eucalyptus globulus
Eucalyptus grandis
Eucalyptus camaldulensis
Eucalyptus diversifolia
Syzygium cumini
Angophora floribunda
Corymbia gummifera
Stockwellia quadrifida
Syzygium forrestii
Heteropyxis natalensis
Punica granatum
0.24
0.14
0.1
0.09
0.28
0.34
0.96
0.16
0.44
0.35
0.97
1
1
1
0.38
0.95
0.39
0.22
0.21
1
0.97
1
0.52
0.4
0.76
0.002

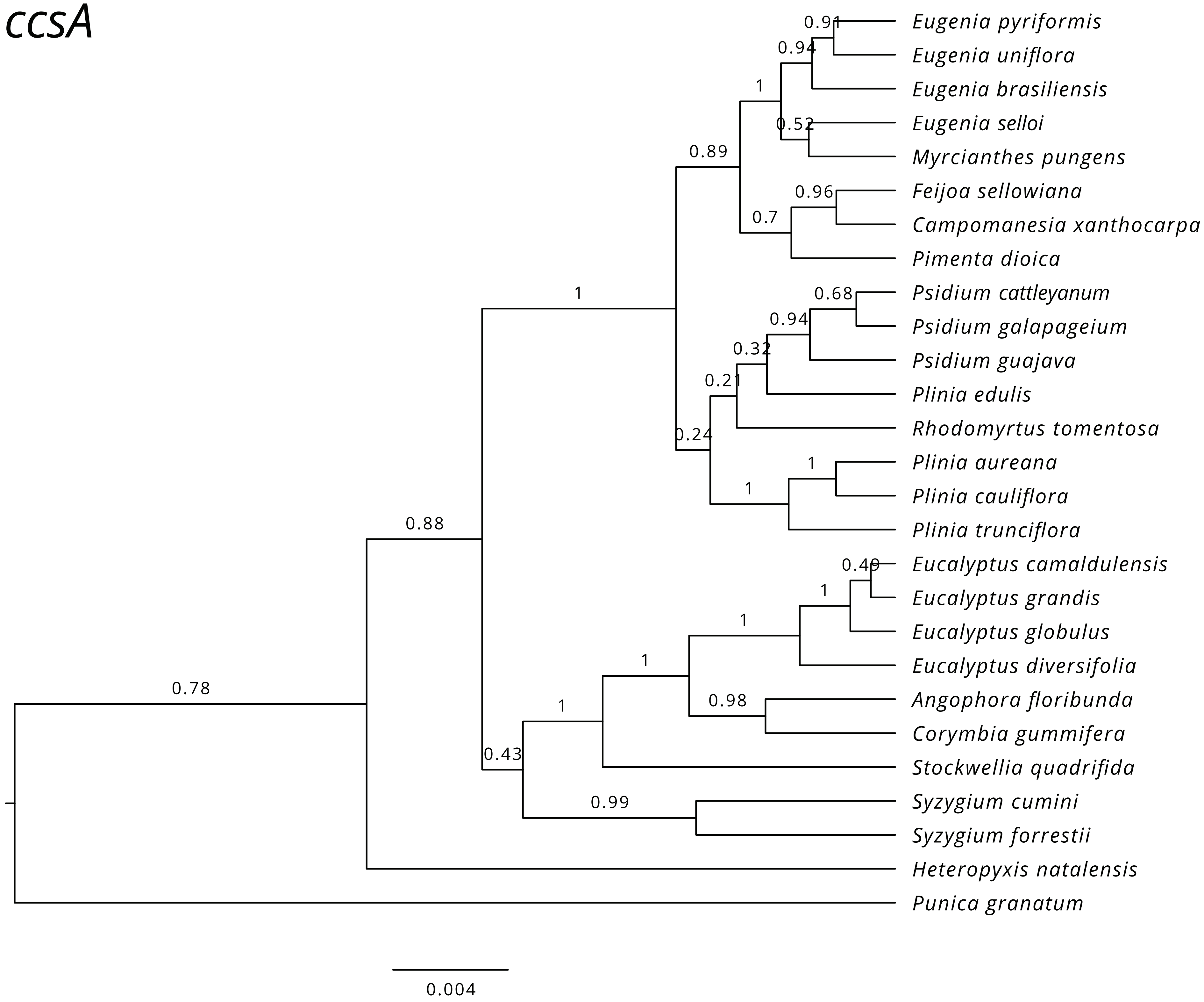
ccsA
Eugenia pyriformis
Eugenia uniflora
Eugenia brasiliensis
Eugenia selloi
Myrcianthes pungens
Feijoa sellowiana
Campomanesia xanthocarpa
Pimenta dioica
Psidium cattleyanum
Psidium galapageium
Psidium guajava
Plinia edulis
Rhodomyrtus tomentosa
Plinia aureana
Plinia cauliflora
Plinia trunciflora
Eucalyptus camaldulensis
Eucalyptus grandis
Eucalyptus globulus
Eucalyptus diversifolia
Angophora floribunda
Corymbia gummifera
Stockwellia quadrifida
Syzygium cumini
Syzygium forrestii
Heteropyxis natalensis
Punica granatum
0.91
0.94
1
0.52
0.89
0.96
0.7
1
0.68
0.94
0.32
0.21
0.24
1
1
0.88
0.49
1
1
1
0.98
1
0.43
0.99
0.78
0.004

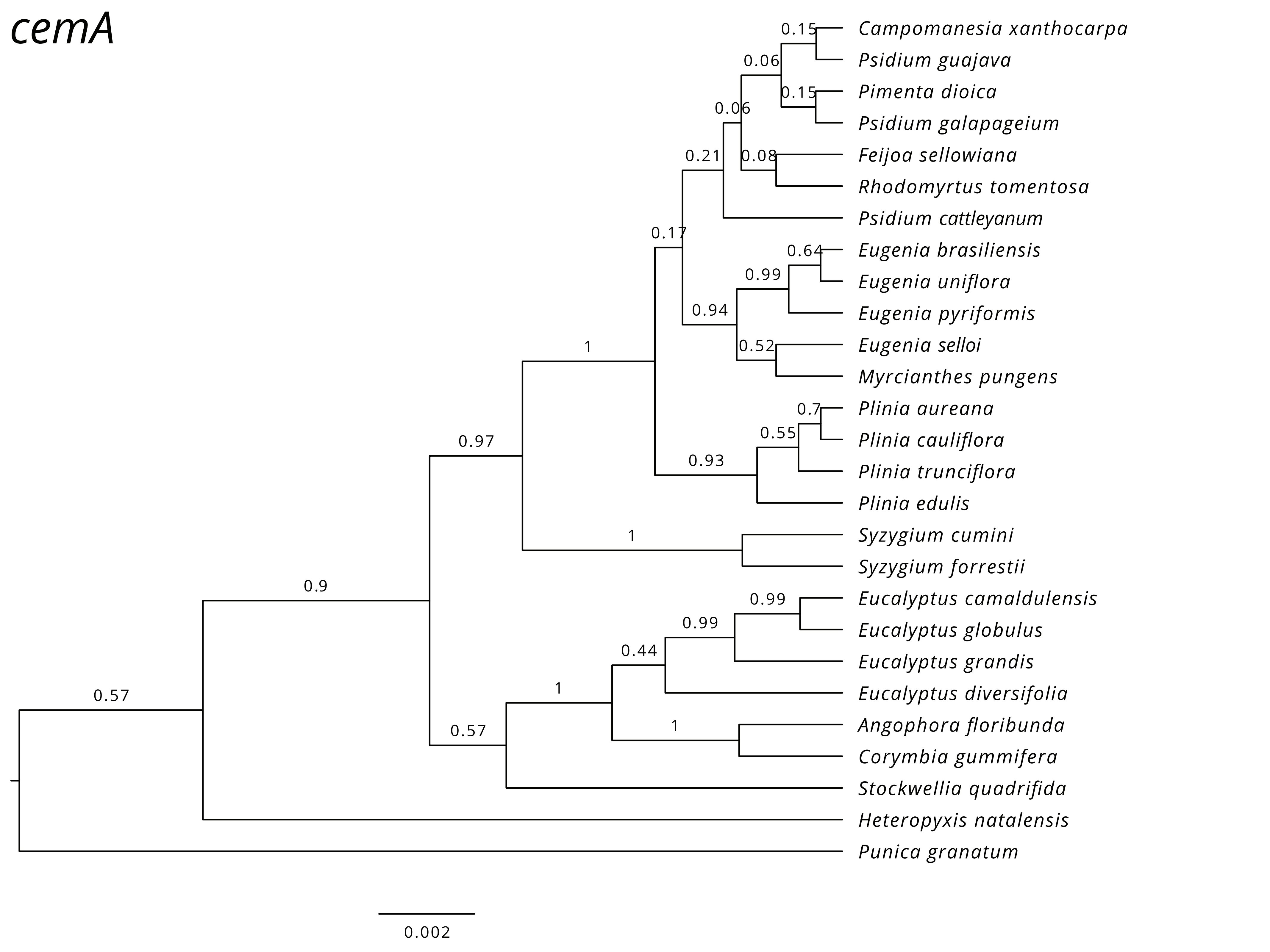
cemA
Campomanesia xanthocarpa
Psidium guajava
Pimenta dioica
Psidium galapageium
Feijoa sellowiana
Rhodomyrtus tomentosa
Psidium cattleyanum
Eugenia brasiliensis
Eugenia uniflora
Eugenia pyriformis
Eugenia selloi
Myrcianthes pungens
Plinia aureana
Plinia cauliflora
Plinia trunciflora
Plinia edulis
Syzygium cumini
Syzygium forrestii
Eucalyptus camaldulensis
Eucalyptus globulus
Eucalyptus grandis
Eucalyptus diversifolia
Angophora floribunda
Corymbia gummifera
Stockwellia quadrifida
Heteropyxis natalensis
Punica granatum
0.15
0.06
0.15
0.06
0.21
0.08
0.17
0.64
0.99
0.94
0.52
1
0.7
0.55
0.97
0.93
1
0.9
0.99
0.99
0.44
1
0.57
1
0.57
0.002

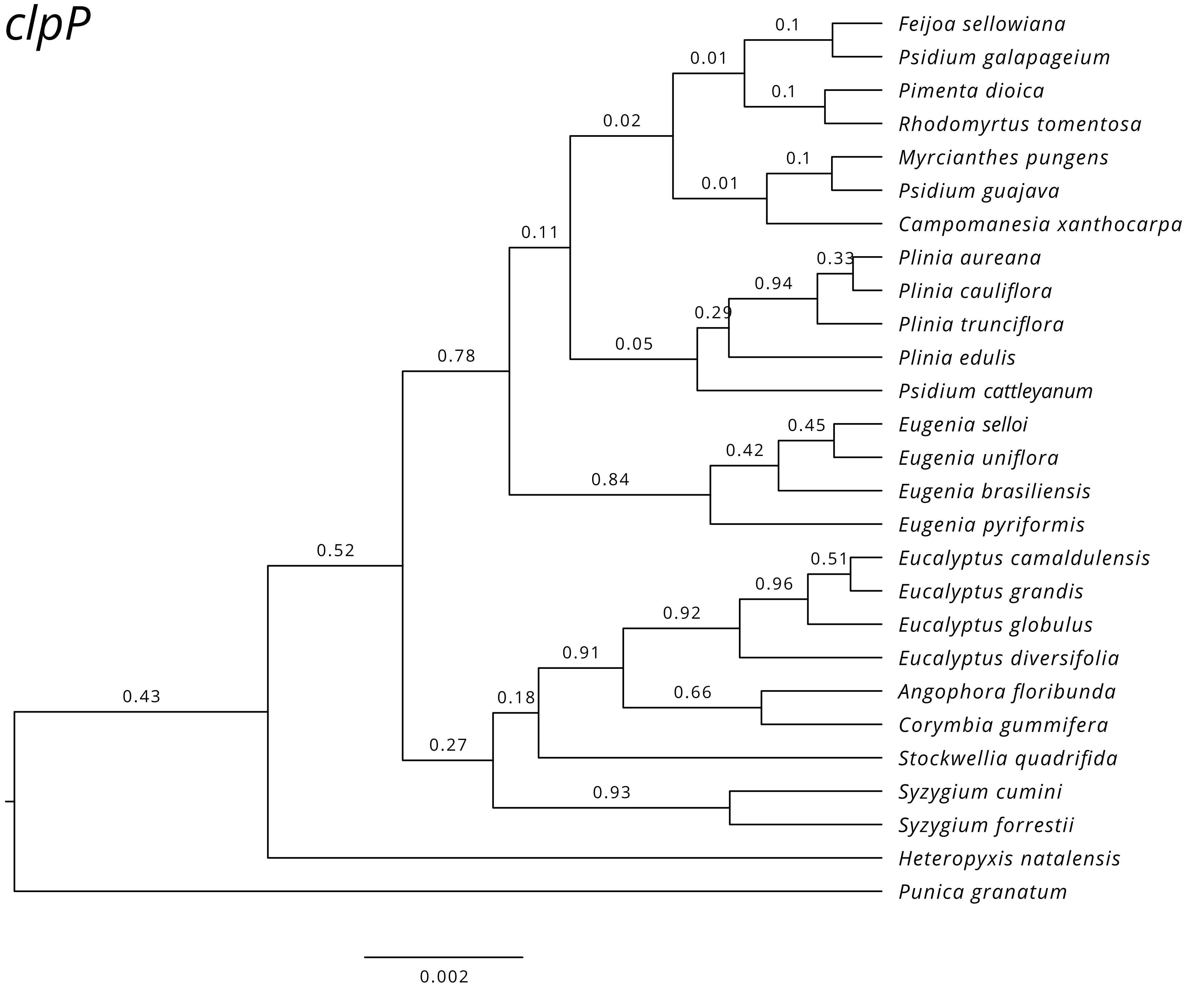
clpP
0.1
0.01
0.1
0.02
0.1
0.01
0.11
0.33
0.94
0.29
0.05
0.78
0.45
0.42
0.84
0.52
0.51
0.96
0.92
0.91
0.66
0.18
0.43
0.27
0.93
Feijoa sellowiana
Psidium galapageium
Pimenta dioica
Rhodomyrtus tomentosa
Myrcianthes pungens
Psidium guajava
Campomanesia xanthocarpa
Plinia aureana
Plinia cauliflora
Plinia trunciflora
Plinia edulis
Psidium cattleyanum
Eugenia selloi
Eugenia uniflora
Eugenia brasiliensis
Eugenia pyriformis
Eucalyptus camaldulensis
Eucalyptus grandis
Eucalyptus globulus
Eucalyptus diversifolia
Angophora floribunda
Corymbia gummifera
Stockwellia quadrifida
Syzygium cumini
Syzygium forrestii
Heteropyxis natalensis
Punica granatum
0.002

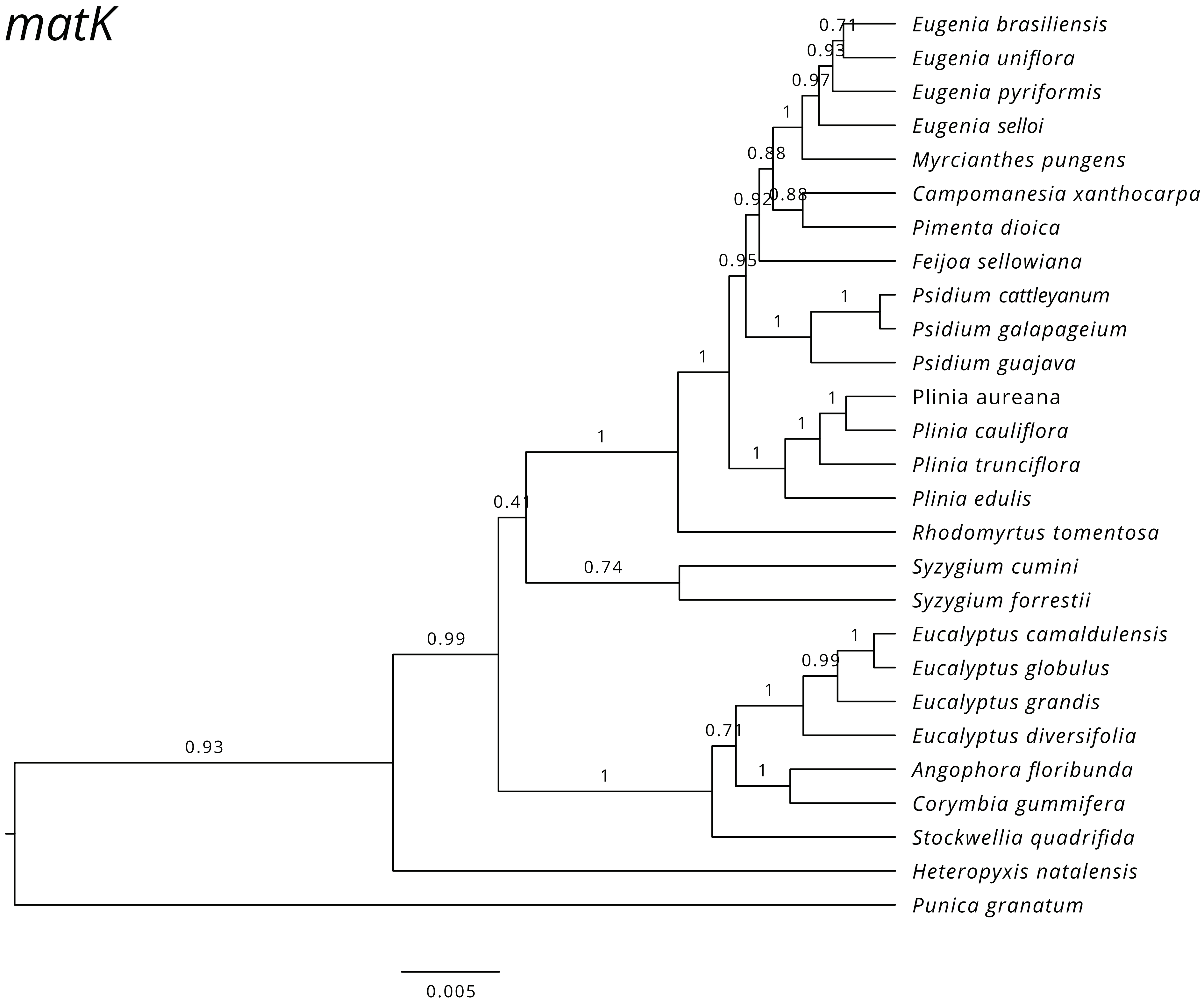
matK
Eugenia brasiliensis
Eugenia uniflora
Eugenia pyriformis
Eugenia selloi
Myrcianthes pungens
Campomanesia xanthocarpa
Pimenta dioica
Feijoa sellowiana
Psidium cattleyanum
Psidium galapageium
Psidium guajava
Plinia aureana
Plinia cauliflora
Plinia trunciflora
Plinia edulis
Rhodomyrtus tomentosa
Syzygium cumini
Syzygium forrestii
Eucalyptus camaldulensis
Eucalyptus globulus
Eucalyptus grandis
Eucalyptus diversifolia
Angophora floribunda
Corymbia gummifera
Stockwellia quadrifida
Heteropyxis natalensis
Punica granatum
0.71
0.93
0.97
1
0.88
0.92
0.88
0.95
1
1
1
1
1
1
1
0.41
0.74
0.99
1
0.99
1
0.71
1
1
0.93
0.005

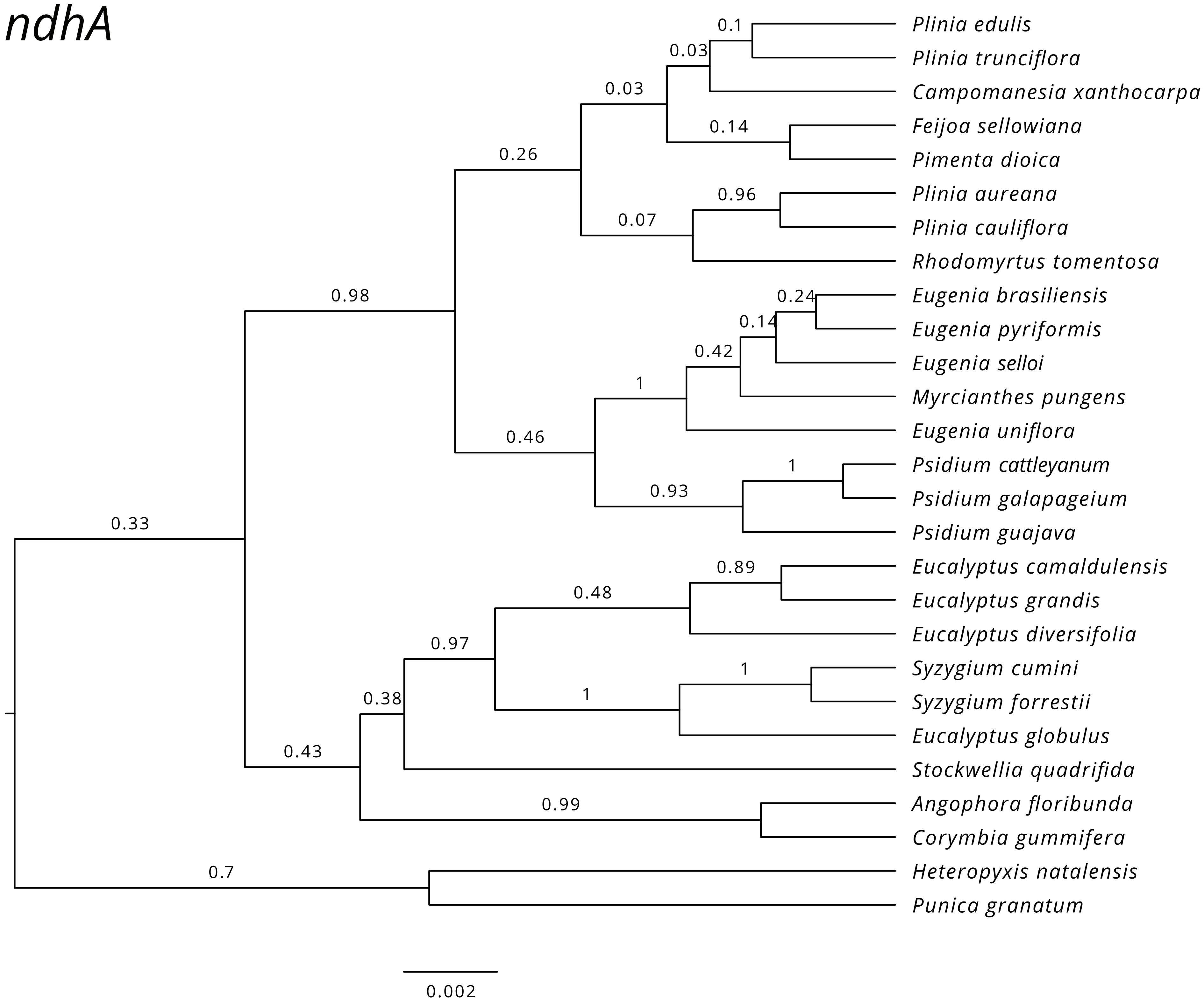
ndhA
0.1
0.03
0.03
0.14
0.26
0.96
0.07
0.98
0.24
0.14
0.42
1
0.46
1
0.93
0.33
0.89
0.48
0.97
1
1
0.38
0.43
0.99
0.7
0.002
Plinia edulis
Plinia trunciflora
Campomanesia xanthocarpa
Feijoa sellowiana
Pimenta dioica
Plinia aureana
Plinia cauliflora
Rhodomyrtus tomentosa
Eugenia brasiliensis
Eugenia pyriformis
Eugenia selloi
Myrcianthes pungens
Eugenia uniflora
Psidium cattleyanum
Psidium galapageium
Psidium guajava
Eucalyptus camaldulensis
Eucalyptus grandis
Eucalyptus diversifolia
Syzygium cumini
Syzygium forrestii
Eucalyptus globulus
Stockwellia quadrifida
Angophora floribunda
Corymbia gummifera
Heteropyxis natalensis
Punica granatum

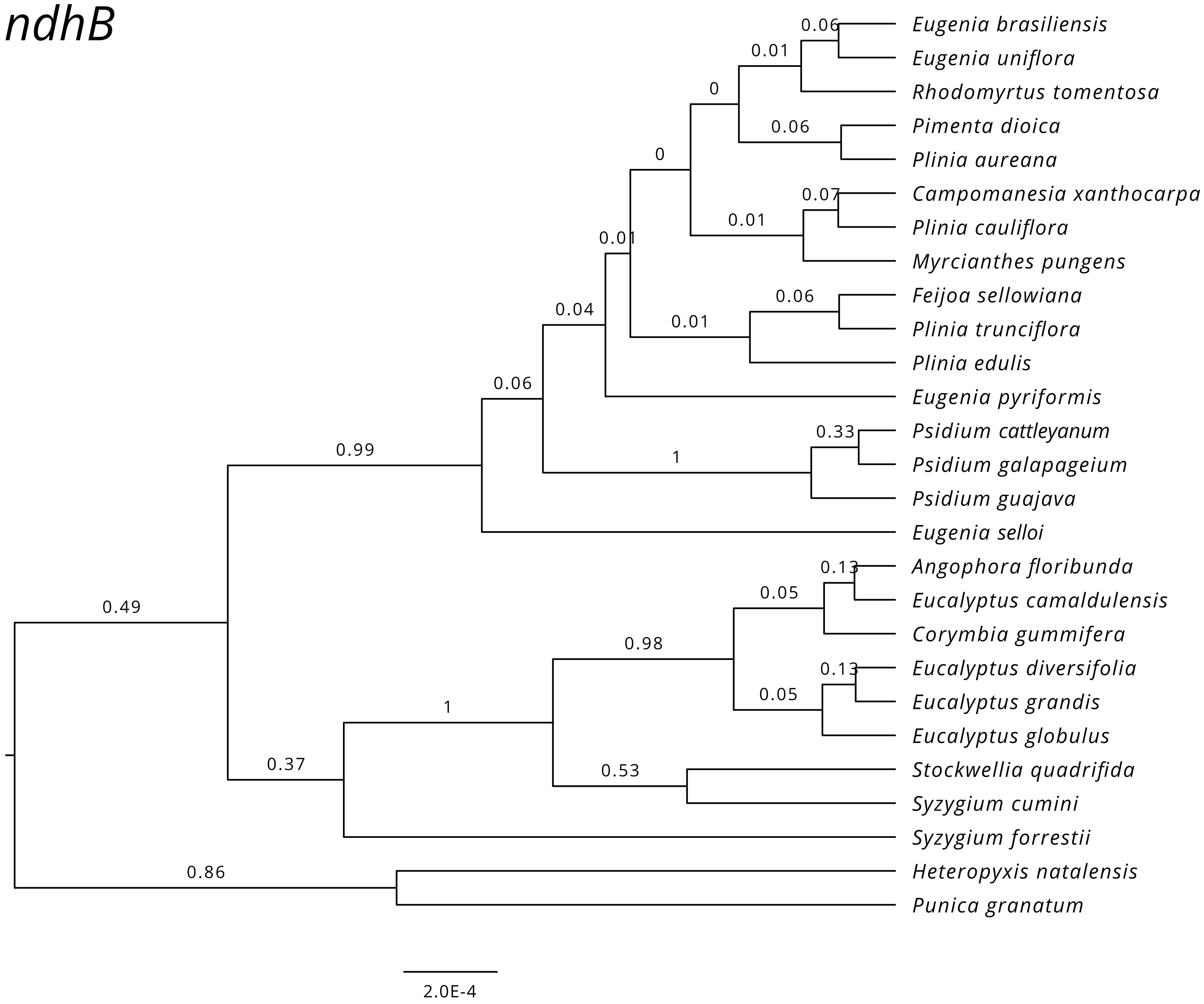
ndhB
0.06
0.01
0
0.06
0
0.07
0.01
0.01
0.04
0.06
0.01
0.06
0.33
0.99
1
0.13
0.05
0.49
0.98
0.13
0.05
1
0.37
0.53
0.86
Eugenia brasiliensis
Eugenia uniflora
Rhodomyrtus tomentosa
Pimenta dioica
Plinia aureana
Campomanesia xanthocarpa
Plinia cauliflora
Myrcianthes pungens
Feijoa sellowiana
Plinia trunciflora
Plinia edulis
Eugenia pyriformis
Psidium cattleyanum
Psidium galapageium
Psidium guajava
Eugenia selloi
Angophora floribunda
Eucalyptus camaldulensis
Corymbia gummifera
Eucalyptus diversifolia
Eucalyptus grandis
Eucalyptus globulus
Stockwellia quadrifida
Syzygium cumini
Syzygium forrestii
Heteropyxis natalensis
Punica granatum
2.0E-4

# *ndhC*

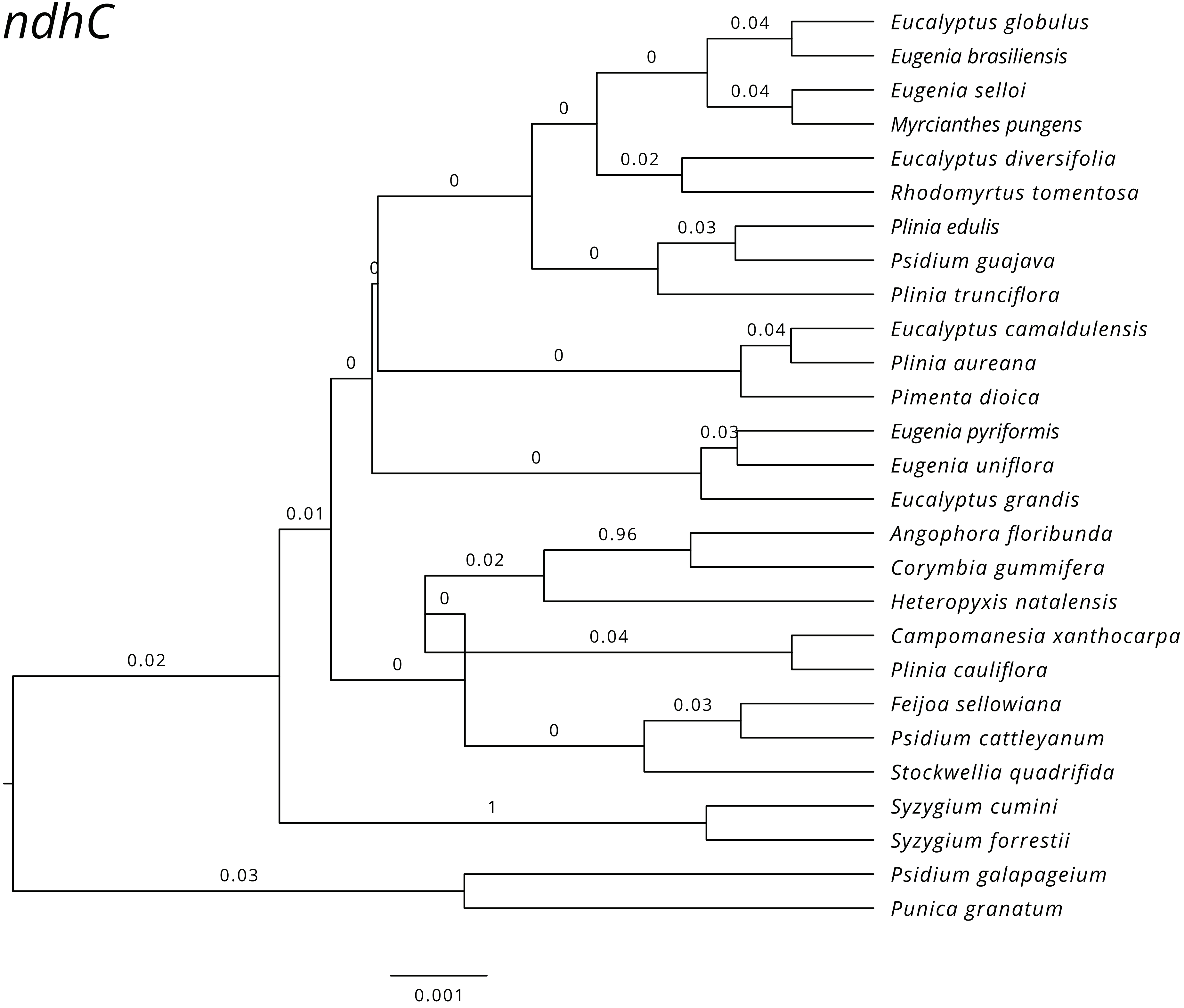
Eucalyptus globulus
Eugenia brasiliensis
Eugenia selloi
Myrcianthes pungens
Eucalyptus diversifolia
Rhodomyrtus tomentosa
Plinia edulis
Psidium guajava
Plinia trunciflora
Eucalyptus camaldulensis
Plinia aureana
Pimenta dioica
Eugenia pyriformis
Eugenia uniflora
Eucalyptus grandis
Angophora floribunda
Corymbia gummifera
Heteropyxis natalensis
Campomanesia xanthocarpa
Plinia cauliflora
Feijoa sellowiana
Psidium cattleyanum
Stockwellia quadrifida
Syzygium cumini
Syzygium forrestii
Psidium galapageium
Punica granatum
0.04
0
0.04
0
0.02
0
0.03
0
0
0.04
0
0
0.03
0
0.01
0.96
0.02
0
0.04
0.02
0
0.03
0
1
0.03
0.001

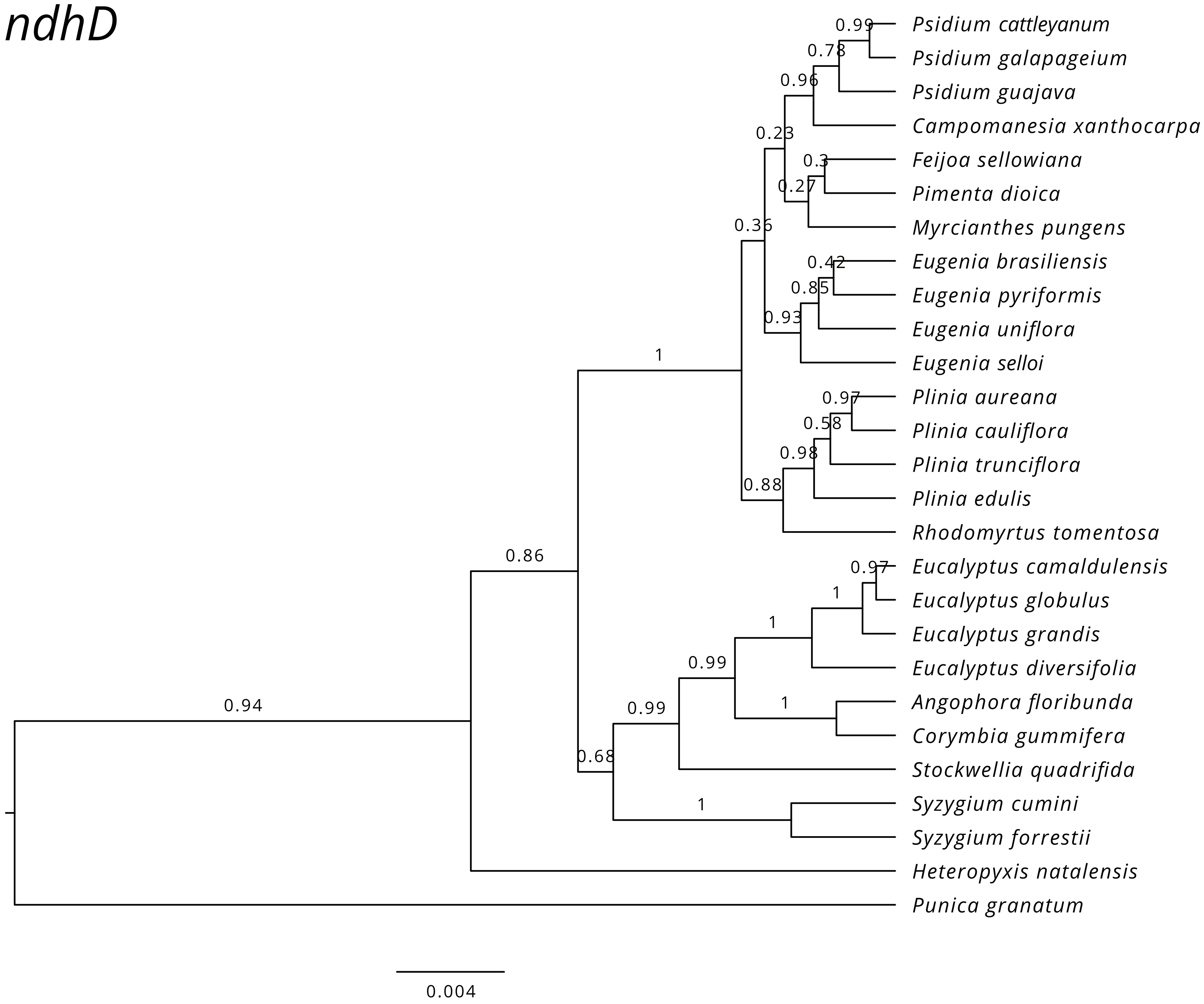
ndhD
0.99
Psidium cattleyanum
0.78
Psidium galapageium
0.96
Psidium guajava
Campomanesia xanthocarpa
0.23
Feijoa sellowiana
0.3
Pimenta dioica
0.27
Myrcianthes pungens
0.36
Eugenia brasiliensis
0.42
Eugenia pyriformis
0.85
Eugenia uniflora
0.93
Eugenia selloi
1
Plinia aureana
0.97
Plinia cauliflora
0.58
Plinia trunciflora
0.98
Plinia edulis
0.88
Rhodomyrtus tomentosa
0.86
Eucalyptus camaldulensis
0.97
Eucalyptus globulus
1
Eucalyptus grandis
1
Eucalyptus diversifolia
0.99
Angophora floribunda
1
Corymbia gummifera
0.99
Stockwellia quadrifida
0.68
Syzygium cumini
1
Syzygium forrestii
0.94
Heteropyxis natalensis
Punica granatum
0.004

# *ndhE*

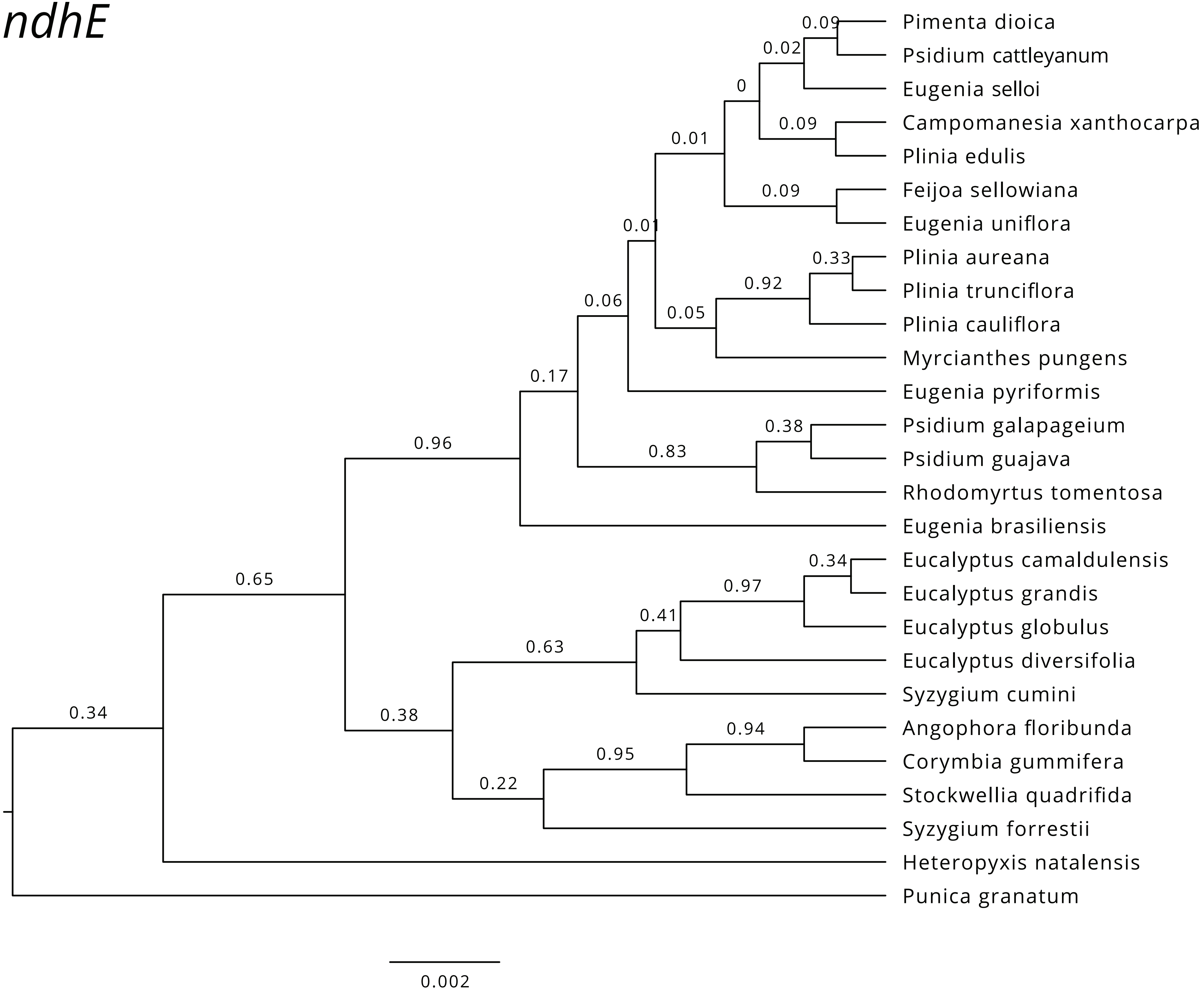

Pimenta dioica
Psidium cattleyanum
Eugenia selloi
Campomanesia xanthocarpa
Plinia edulis
Feijoa sellowiana
Eugenia uniflora
Plinia aureana
Plinia trunciflora
Plinia cauliflora
Myrcianthes pungens
Eugenia pyriformis
Psidium galapageium
Psidium guajava
Rhodomyrtus tomentosa
Eugenia brasiliensis
Eucalyptus camaldulensis
Eucalyptus grandis
Eucalyptus globulus
Eucalyptus diversifolia
Syzygium cumini
Angophora floribunda
Corymbia gummifera
Stockwellia quadrifida
Syzygium forrestii
Heteropyxis natalensis
Punica granatum
0.09
0.02
0
0.09
0.01
0.09
0.01
0.33
0.92
0.05
0.06
0.17
0.38
0.83
0.96
0.34
0.97
0.41
0.65
0.63
0.38
0.94
0.95
0.22
0.34
0.002

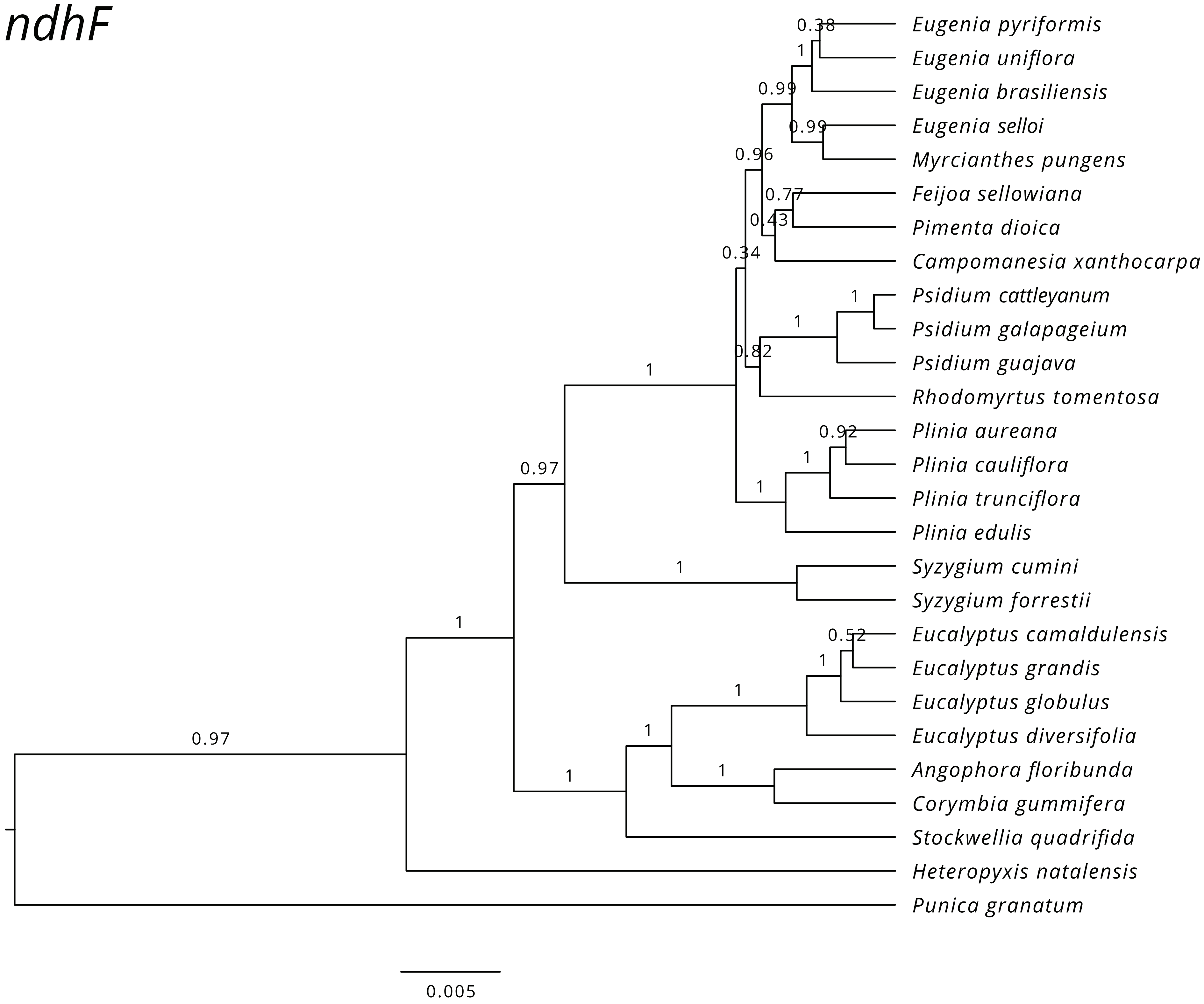
ndhF
Eugenia pyriformis
Eugenia uniflora
Eugenia brasiliensis
Eugenia selloi
Myrcianthes pungens
Feijoa sellowiana
Pimenta dioica
Campomanesia xanthocarpa
Psidium cattleyanum
Psidium galapageium
Psidium guajava
Rhodomyrtus tomentosa
Plinia aureana
Plinia cauliflora
Plinia trunciflora
Plinia edulis
Syzygium cumini
Syzygium forrestii
Eucalyptus camaldulensis
Eucalyptus grandis
Eucalyptus globulus
Eucalyptus diversifolia
Angophora floribunda
Corymbia gummifera
Stockwellia quadrifida
Heteropyxis natalensis
Punica granatum
0.38
1
0.99
0.99
0.96
0.77
0.43
0.34
1
1
0.82
1
0.92
1
1
0.97
1
1
0.52
1
1
1
1
1
0.97
0.005

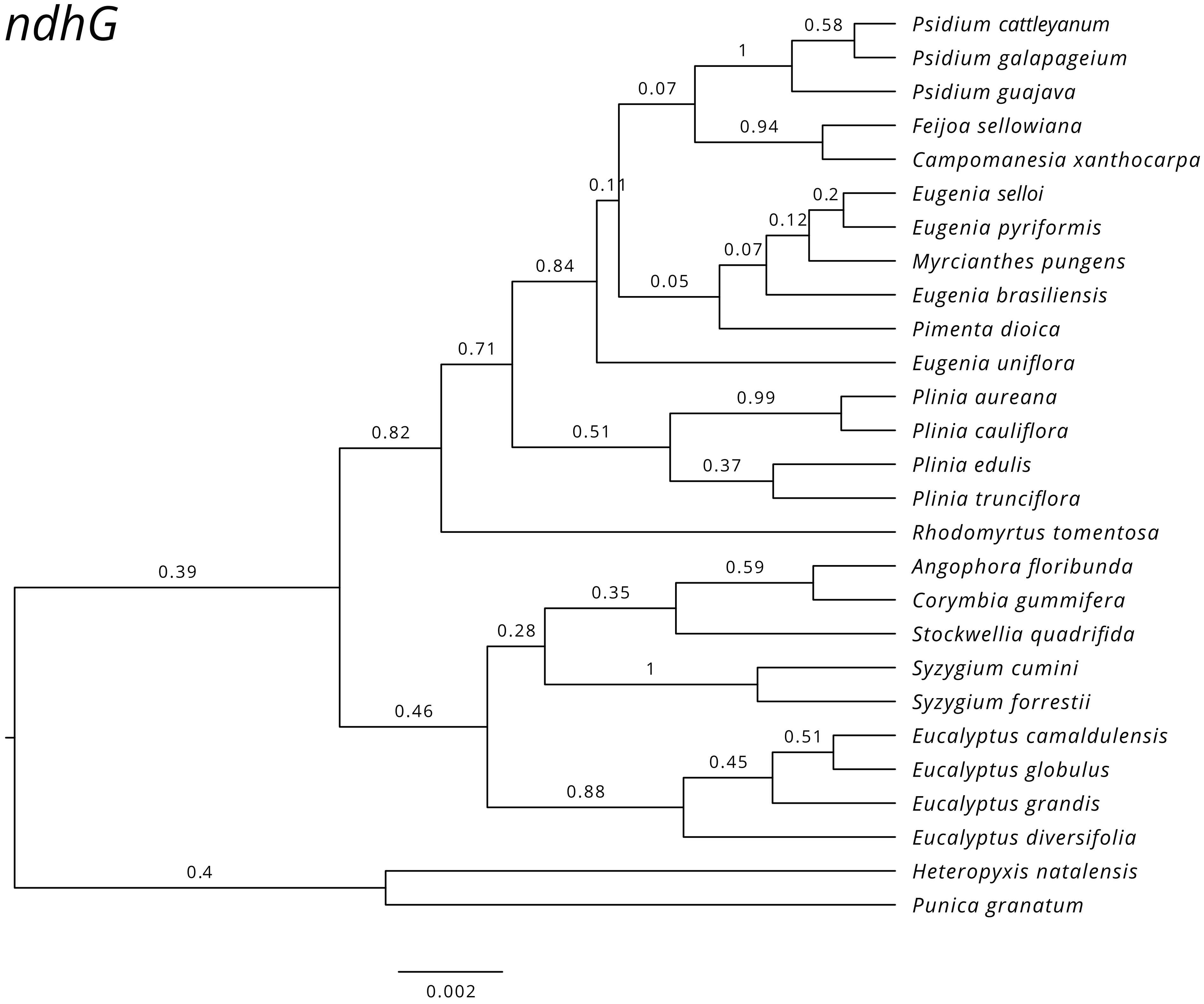
ndhG
0.58
1
0.07
0.94
0.11
0.2
0.12
0.07
0.84
0.05
0.71
0.99
0.82
0.51
0.37
0.39
0.59
0.35
0.28
1
0.46
0.51
0.45
0.88
0.4
Psidium cattleyanum
Psidium galapageium
Psidium guajava
Feijoa sellowiana
Campomanesia xanthocarpa
Eugenia selloi
Eugenia pyriformis
Myrcianthes pungens
Eugenia brasiliensis
Pimenta dioica
Eugenia uniflora
Plinia aureana
Plinia cauliflora
Plinia edulis
Plinia trunciflora
Rhodomyrtus tomentosa
Angophora floribunda
Corymbia gummifera
Stockwellia quadrifida
Syzygium cumini
Syzygium forrestii
Eucalyptus camaldulensis
Eucalyptus globulus
Eucalyptus grandis
Eucalyptus diversifolia
Heteropyxis natalensis
Punica granatum
0.002

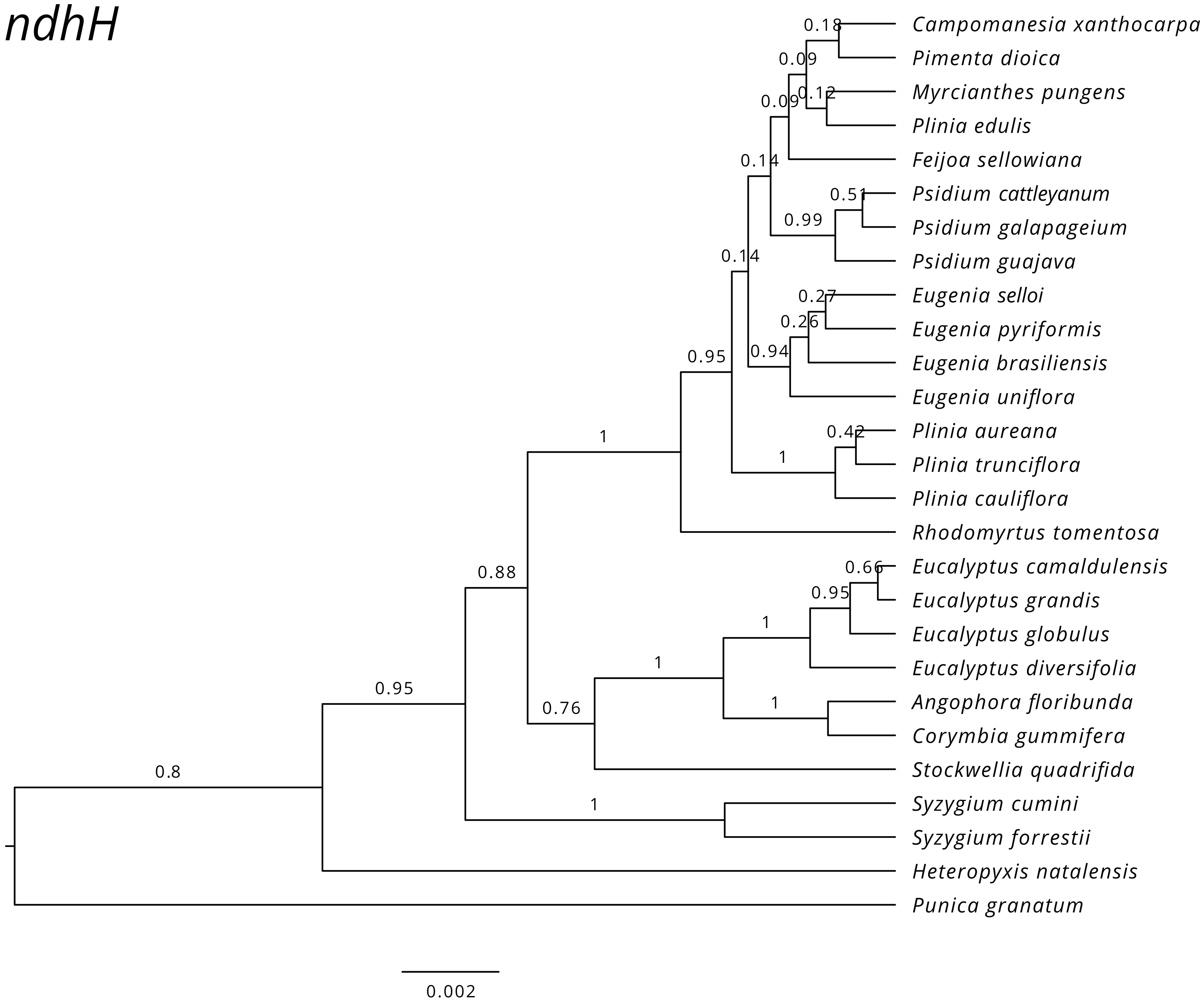
ndhH
0.18
0.09
0.12
0.09
0.14
0.51
0.99
0.14
0.27
0.26
0.94
0.95
0.42
1
1
0.66
0.95
0.88
1
1
1
0.95
0.76
0.8
1
Campomanesia xanthocarpa
Pimenta dioica
Myrcianthes pungens
Plinia edulis
Feijoa sellowiana
Psidium cattleyanum
Psidium galapageium
Psidium guajava
Eugenia selloi
Eugenia pyriformis
Eugenia brasiliensis
Eugenia uniflora
Plinia aureana
Plinia trunciflora
Plinia cauliflora
Rhodomyrtus tomentosa
Eucalyptus camaldulensis
Eucalyptus grandis
Eucalyptus globulus
Eucalyptus diversifolia
Angophora floribunda
Corymbia gummifera
Stockwellia quadrifida
Syzygium cumini
Syzygium forrestii
Heteropyxis natalensis
Punica granatum
0.002

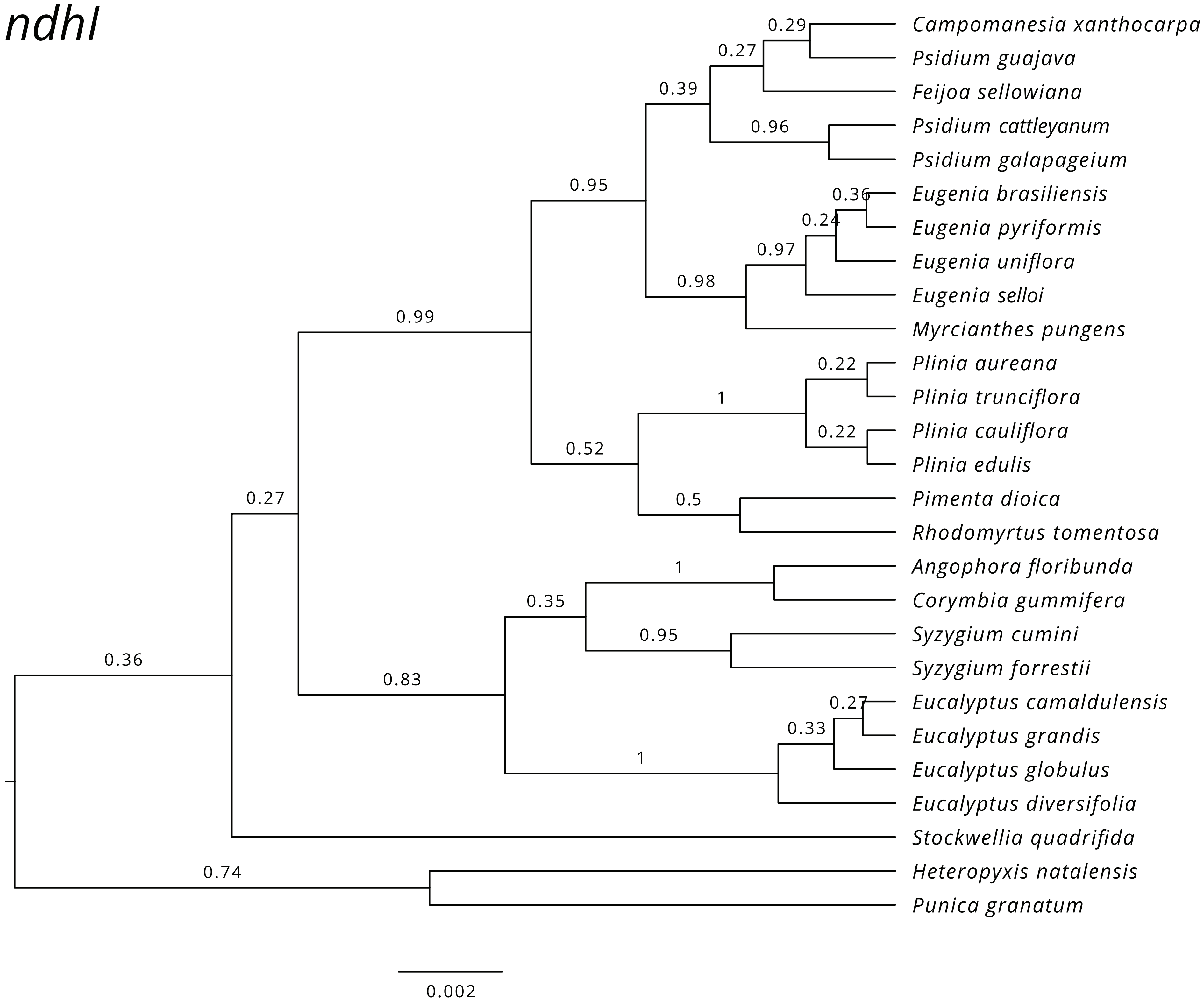
ndhI
Campomanesia xanthocarpa
Psidium guajava
Feijoa sellowiana
Psidium cattleyanum
Psidium galapageium
Eugenia brasiliensis
Eugenia pyriformis
Eugenia uniflora
Eugenia selloi
Myrcianthes pungens
Plinia aureana
Plinia trunciflora
Plinia cauliflora
Plinia edulis
Pimenta dioica
Rhodomyrtus tomentosa
Angophora floribunda
Corymbia gummifera
Syzygium cumini
Syzygium forrestii
Eucalyptus camaldulensis
Eucalyptus grandis
Eucalyptus globulus
Eucalyptus diversifolia
Stockwellia quadrifida
Heteropyxis natalensis
Punica granatum
0.29
0.27
0.39
0.96
0.95
0.36
0.24
0.97
0.98
0.99
0.22
1
0.22
0.52
0.5
0.27
1
0.35
0.95
0.36
0.83
0.27
0.33
1
0.74
0.002

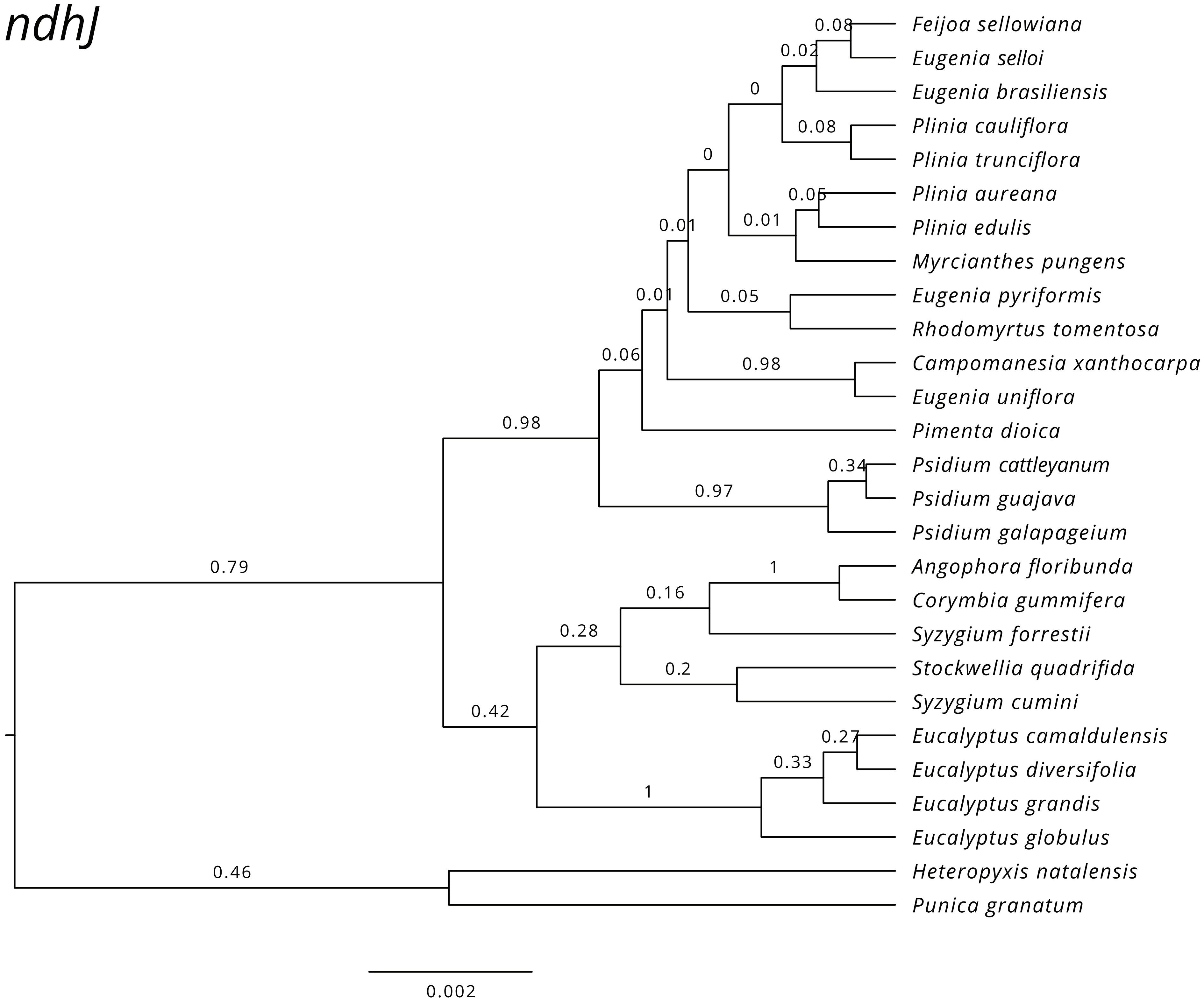
ndhJ
0.08
0.02
0
0.08
0
0.05
0.01
0.01
0.01
0.05
0.98
0.06
0.98
0.34
0.97
0.79
1
0.16
0.28
0.2
0.42
0.27
0.33
1
0.46
Feijoa sellowiana
Eugenia selloi
Eugenia brasiliensis
Plinia cauliflora
Plinia trunciflora
Plinia aureana
Plinia edulis
Myrcianthes pungens
Eugenia pyriformis
Rhodomyrtus tomentosa
Campomanesia xanthocarpa
Eugenia uniflora
Pimenta dioica
Psidium cattleyanum
Psidium guajava
Psidium galapageium
Angophora floribunda
Corymbia gummifera
Syzygium forrestii
Stockwellia quadrifida
Syzygium cumini
Eucalyptus camaldulensis
Eucalyptus diversifolia
Eucalyptus grandis
Eucalyptus globulus
Heteropyxis natalensis
Punica granatum
0.002

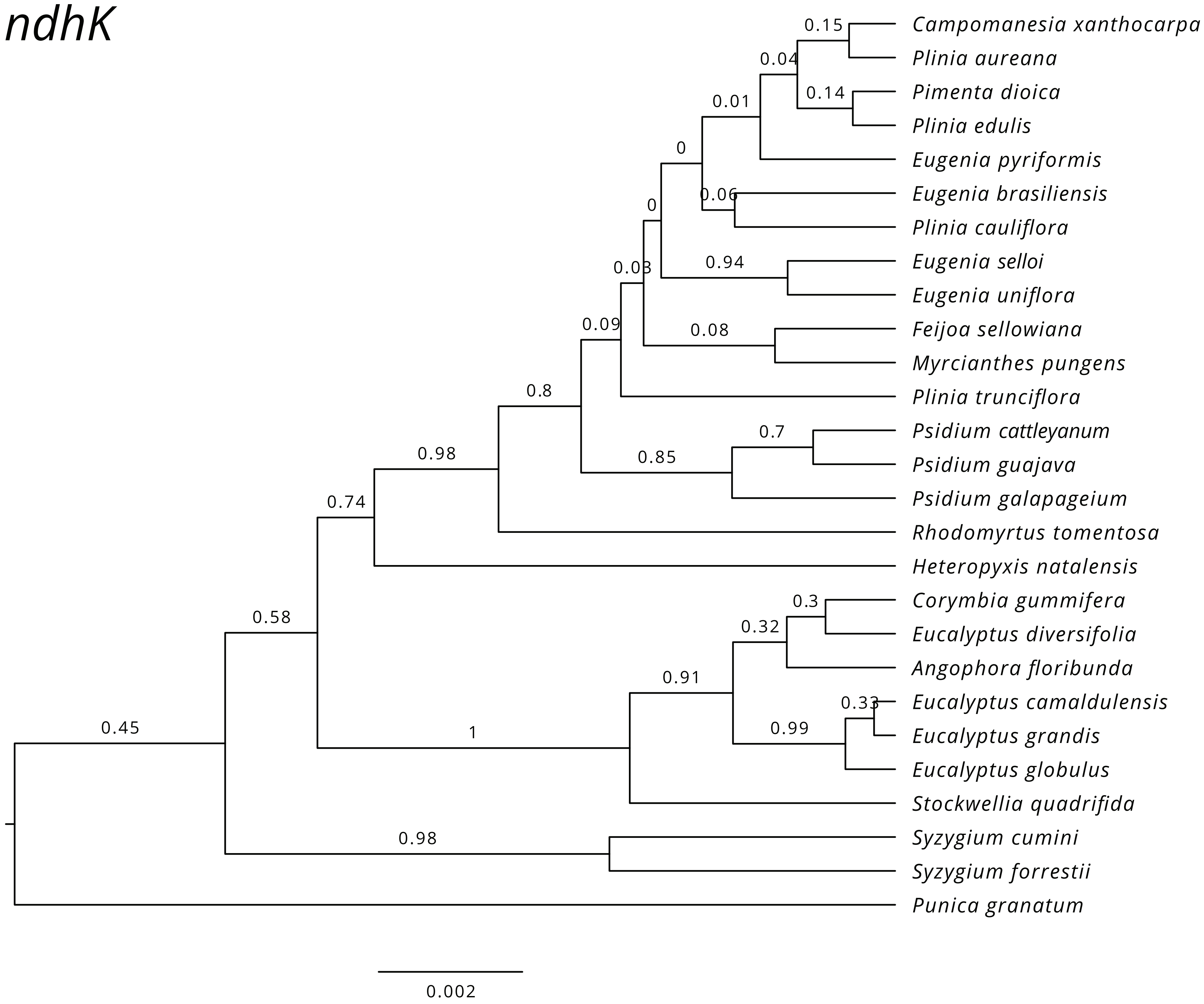
ndhK
0.15
Campomanesia xanthocarpa
Plinia aureana
0.04
0.14
Pimenta dioica
0.01
Plinia edulis
0
Eugenia pyriformis
0.06
Eugenia brasiliensis
0
Plinia cauliflora
0.94
Eugenia selloi
0.03
Eugenia uniflora
0.09
0.08
Feijoa sellowiana
Myrcianthes pungens
0.8
Plinia trunciflora
0.7
Psidium cattleyanum
0.98
0.85
Psidium guajava
Psidium galapageium
0.74
Rhodomyrtus tomentosa
Heteropyxis natalensis
0.3
Corymbia gummifera
0.58
0.32
Eucalyptus diversifolia
Angophora floribunda
0.91
0.33
Eucalyptus camaldulensis
0.45
1
0.99
Eucalyptus grandis
Eucalyptus globulus
Stockwellia quadrifida
0.98
Syzygium cumini
Syzygium forrestii
Punica granatum
0.002

*petA*

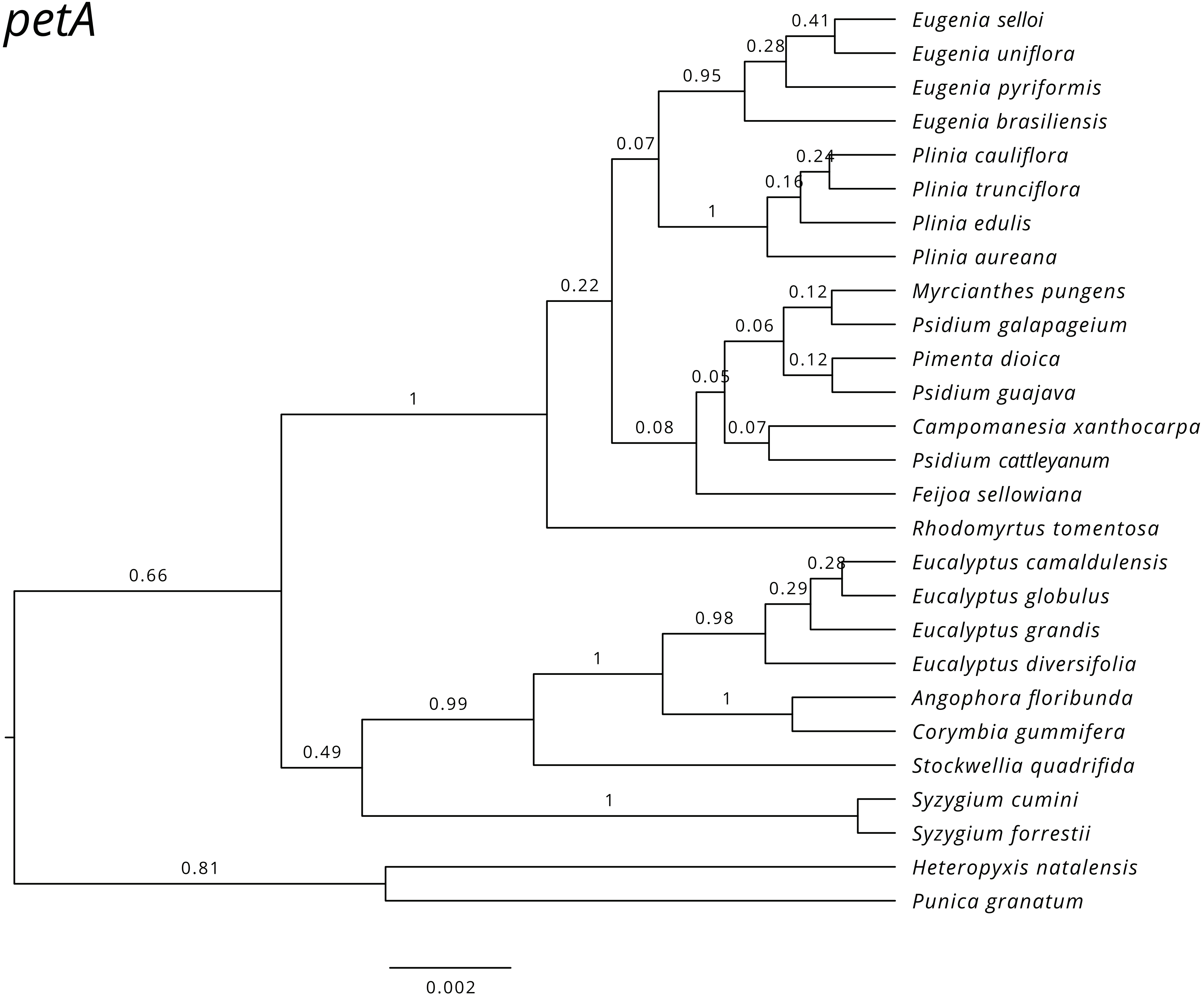

0.41
0.28
0.95
0.07
0.24
0.16
1
0.22
0.12
0.06
0.12
0.05
1
0.07
0.08
0.66
0.28
0.29
0.98
1
0.99
1
0.49
1
0.81
Eugenia selloi
Eugenia uniflora
Eugenia pyriformis
Eugenia brasiliensis
Plinia cauliflora
Plinia trunciflora
Plinia edulis
Plinia aureana
Myrcianthes pungens
Psidium galapageium
Pimenta dioica
Psidium guajava
Campomanesia xanthocarpa
Psidium cattleyanum
Feijoa sellowiana
Rhodomyrtus tomentosa
Eucalyptus camaldulensis
Eucalyptus globulus
Eucalyptus grandis
Eucalyptus diversifolia
Angophora floribunda
Corymbia gummifera
Stockwellia quadrifida
Syzygium cumini
Syzygium forrestii
Heteropyxis natalensis
Punica granatum
0.002

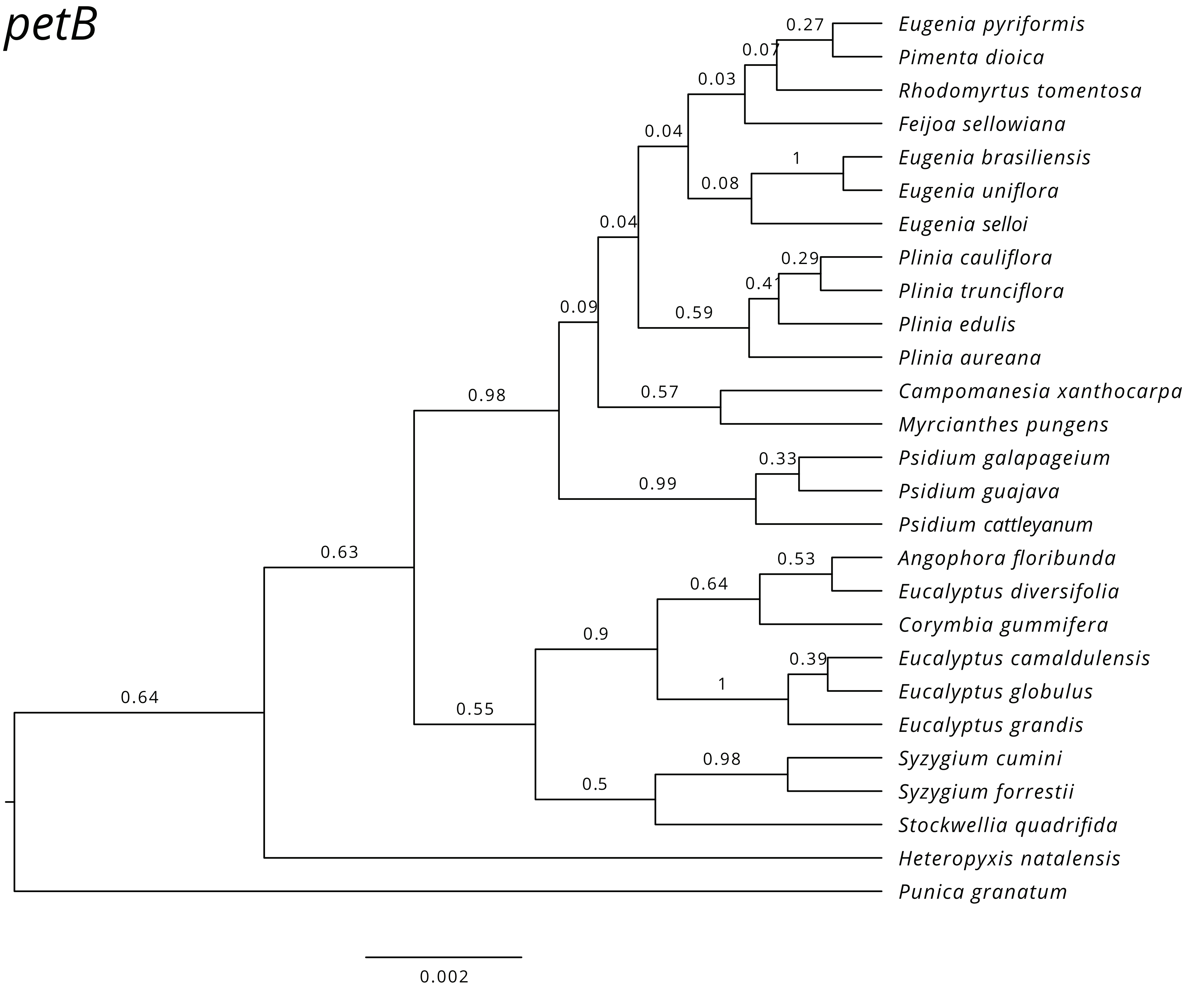

petB
0.27
0.07
0.03
0.04
1
0.08
0.04
0.29
0.41
0.09
0.59
0.98
0.57
0.33
0.99
0.63
0.53
0.64
0.9
0.39
1
0.64
0.55
0.98
0.5
Eugenia pyriformis
Pimenta dioica
Rhodomyrtus tomentosa
Feijoa sellowiana
Eugenia brasiliensis
Eugenia uniflora
Eugenia selloi
Plinia cauliflora
Plinia trunciflora
Plinia edulis
Plinia aureana
Campomanesia xanthocarpa
Myrcianthes pungens
Psidium galapageium
Psidium guajava
Psidium cattleyanum
Angophora floribunda
Eucalyptus diversifolia
Corymbia gummifera
Eucalyptus camaldulensis
Eucalyptus globulus
Eucalyptus grandis
Syzygium cumini
Syzygium forrestii
Stockwellia quadrifida
Heteropyxis natalensis
Punica granatum
0.002

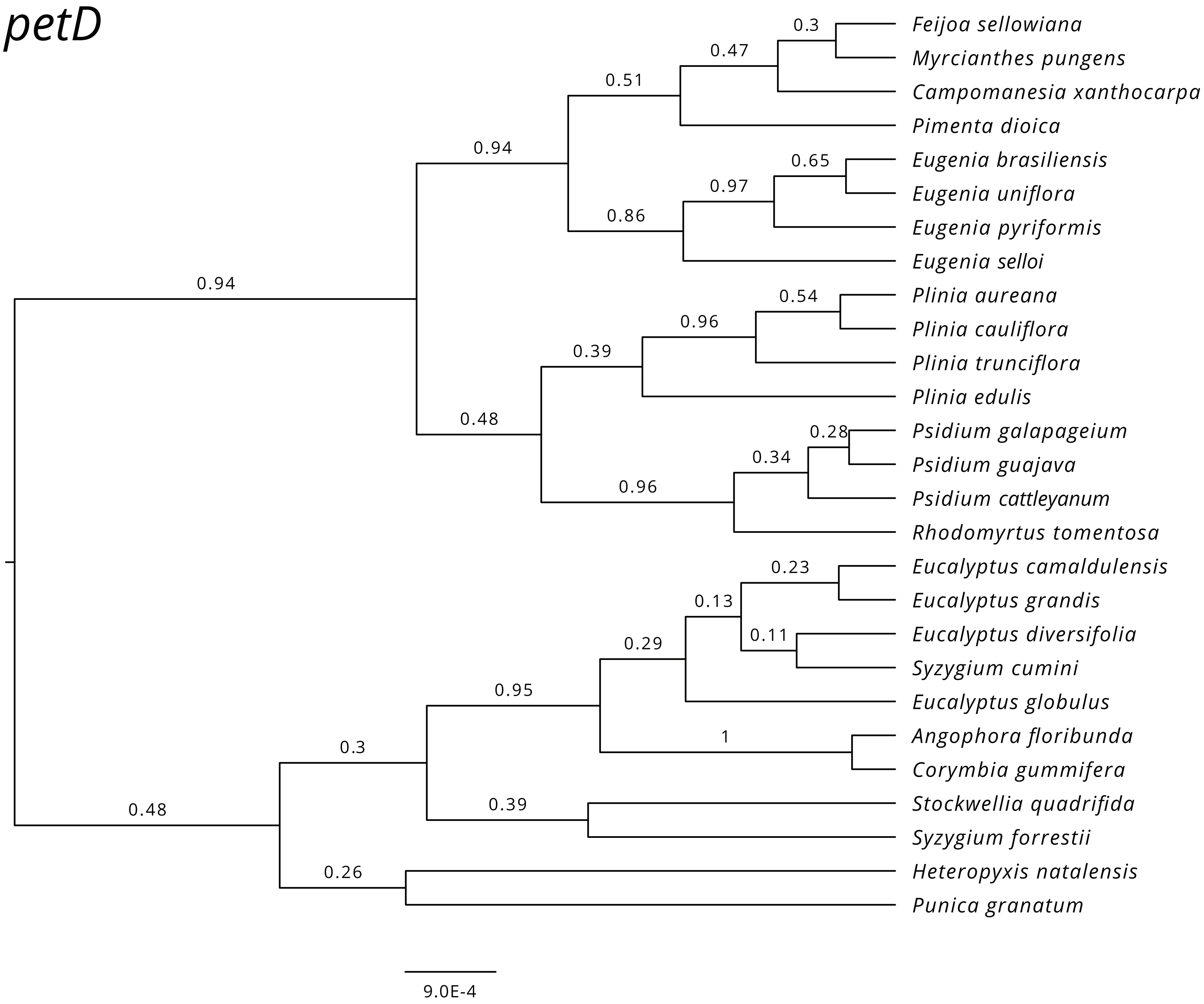
petD
0.3
0.47
0.51
0.94
0.65
0.97
0.86
0.94
0.54
0.96
0.39
0.48
0.28
0.34
0.96
0.23
0.13
0.11
0.29
0.95
1
0.3
0.39
0.48
0.26
Feijoa sellowiana
Myrcianthes pungens
Campomanesia xanthocarpa
Pimenta dioica
Eugenia brasiliensis
Eugenia uniflora
Eugenia pyriformis
Eugenia selloi
Plinia aureana
Plinia cauliflora
Plinia trunciflora
Plinia edulis
Psidium galapageium
Psidium guajava
Psidium cattleyanum
Rhodomyrtus tomentosa
Eucalyptus camaldulensis
Eucalyptus grandis
Eucalyptus diversifolia
Syzygium cumini
Eucalyptus globulus
Angophora floribunda
Corymbia gummifera
Stockwellia quadrifida
Syzygium forrestii
Heteropyxis natalensis
Punica granatum
9.0E-4

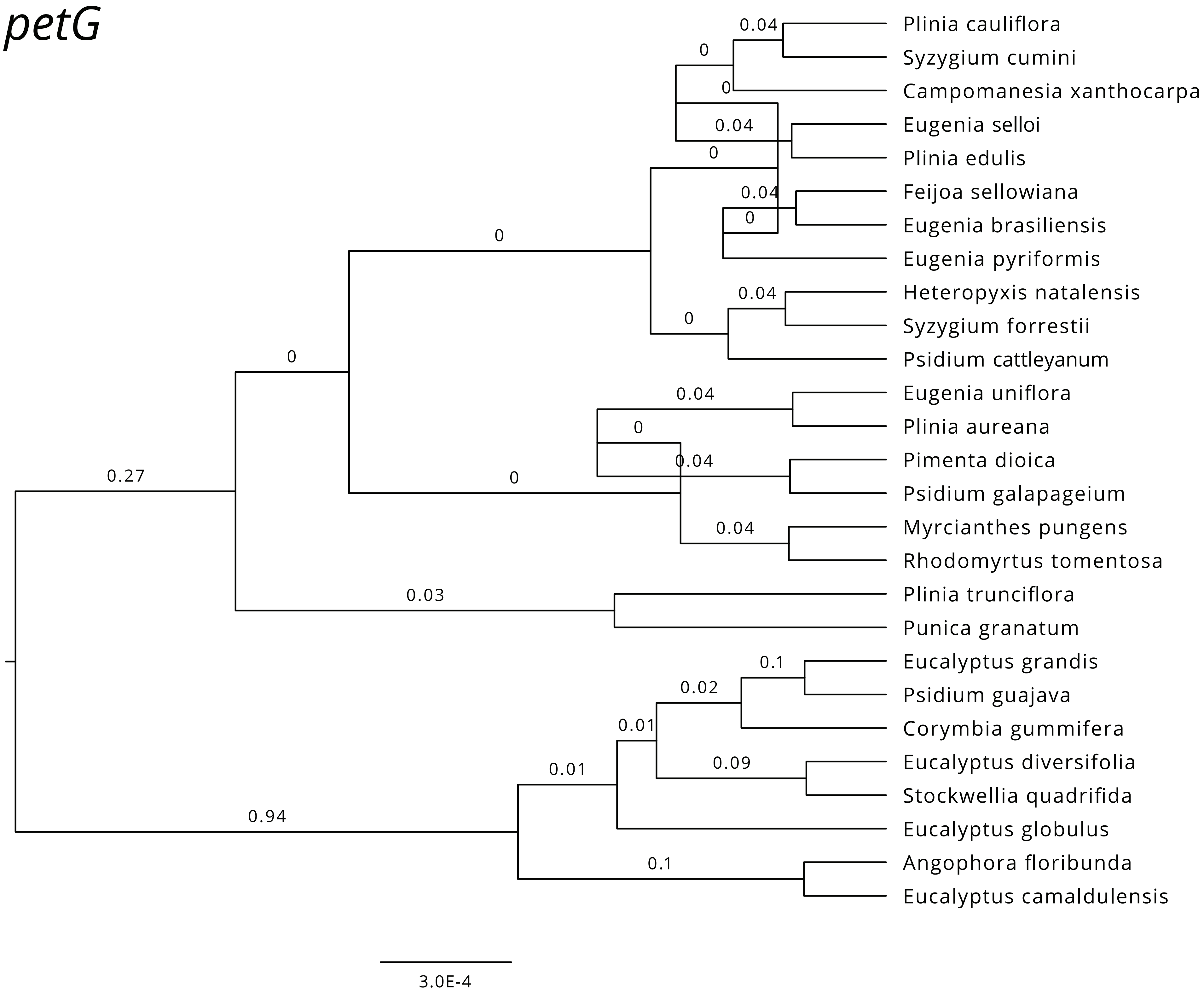
petG
Plinia cauliflora
Syzygium cumini
Campomanesia xanthocarpa
Eugenia selloi
Plinia edulis
Feijoa sellowiana
Eugenia brasiliensis
Eugenia pyriformis
Heteropyxis natalensis
Syzygium forrestii
Psidium cattleyanum
Eugenia uniflora
Plinia aureana
Pimenta dioica
Psidium galapageium
Myrcianthes pungens
Rhodomyrtus tomentosa
Plinia trunciflora
Punica granatum
Eucalyptus grandis
Psidium guajava
Corymbia gummifera
Eucalyptus diversifolia
Stockwellia quadrifida
Eucalyptus globulus
Angophora floribunda
Eucalyptus camaldulensis
0.04
0
0
0.04
0
0.04
0
0
0.04
0
0
0.04
0
0.04
0
0.04
0.27
0.03
0.1
0.02
0.01
0.09
0.01
0.94
0.1
3.0E-4

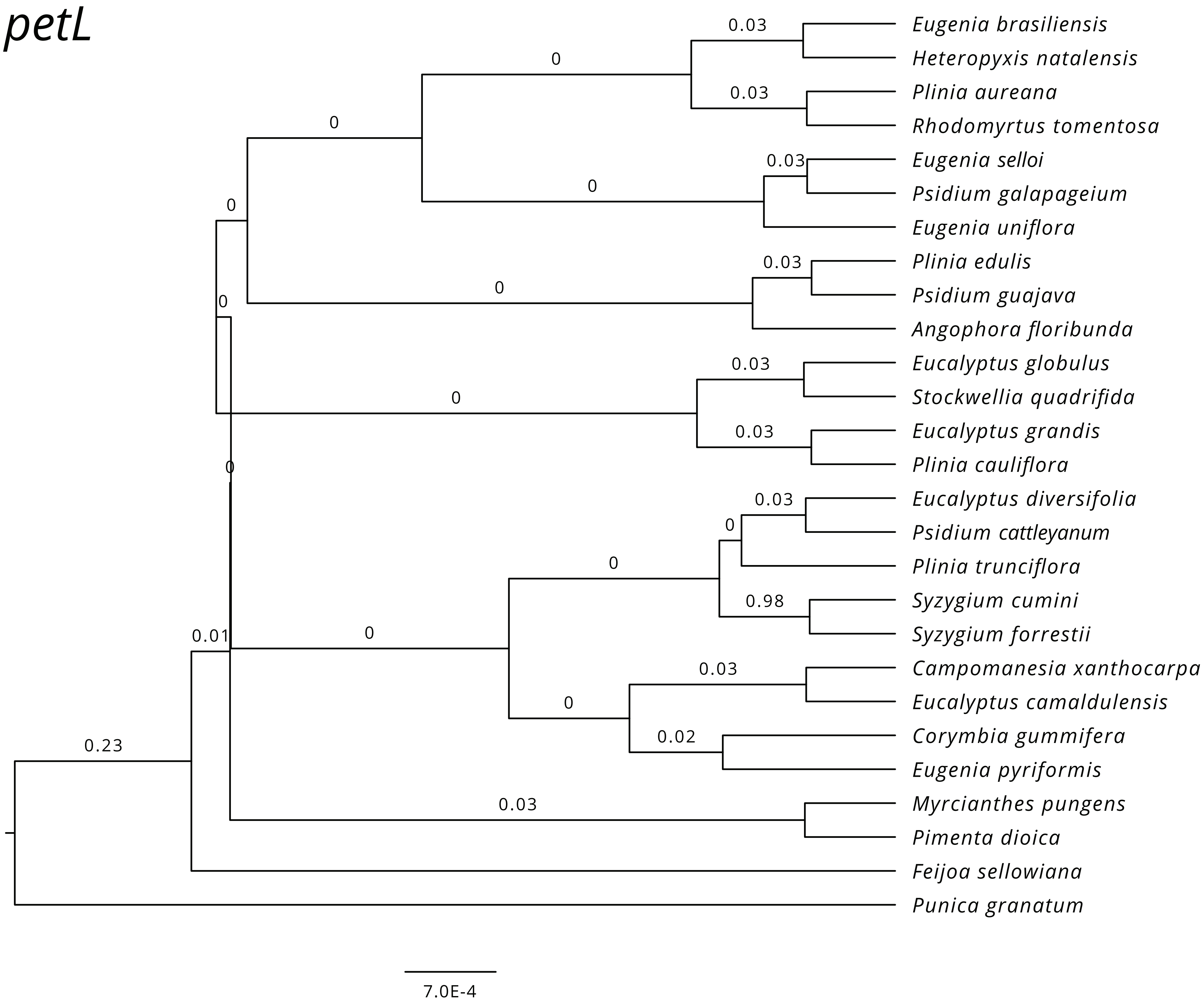
petL
0.03
0
0.03
0
0.03
0
0
0
0.03
0
0
0.03
0
0.03
0
0.03
0
0
0.98
0.01
0
0.03
0
0.02
0.23
0.03
Eugenia brasiliensis
Heteropyxis natalensis
Plinia aureana
Rhodomyrtus tomentosa
Eugenia selloi
Psidium galapageium
Eugenia uniflora
Plinia edulis
Psidium guajava
Angophora floribunda
Eucalyptus globulus
Stockwellia quadrifida
Eucalyptus grandis
Plinia cauliflora
Eucalyptus diversifolia
Psidium cattleyanum
Plinia trunciflora
Syzygium cumini
Syzygium forrestii
Campomanesia xanthocarpa
Eucalyptus camaldulensis
Corymbia gummifera
Eugenia pyriformis
Myrcianthes pungens
Pimenta dioica
Feijoa sellowiana
Punica granatum
7.0E-4

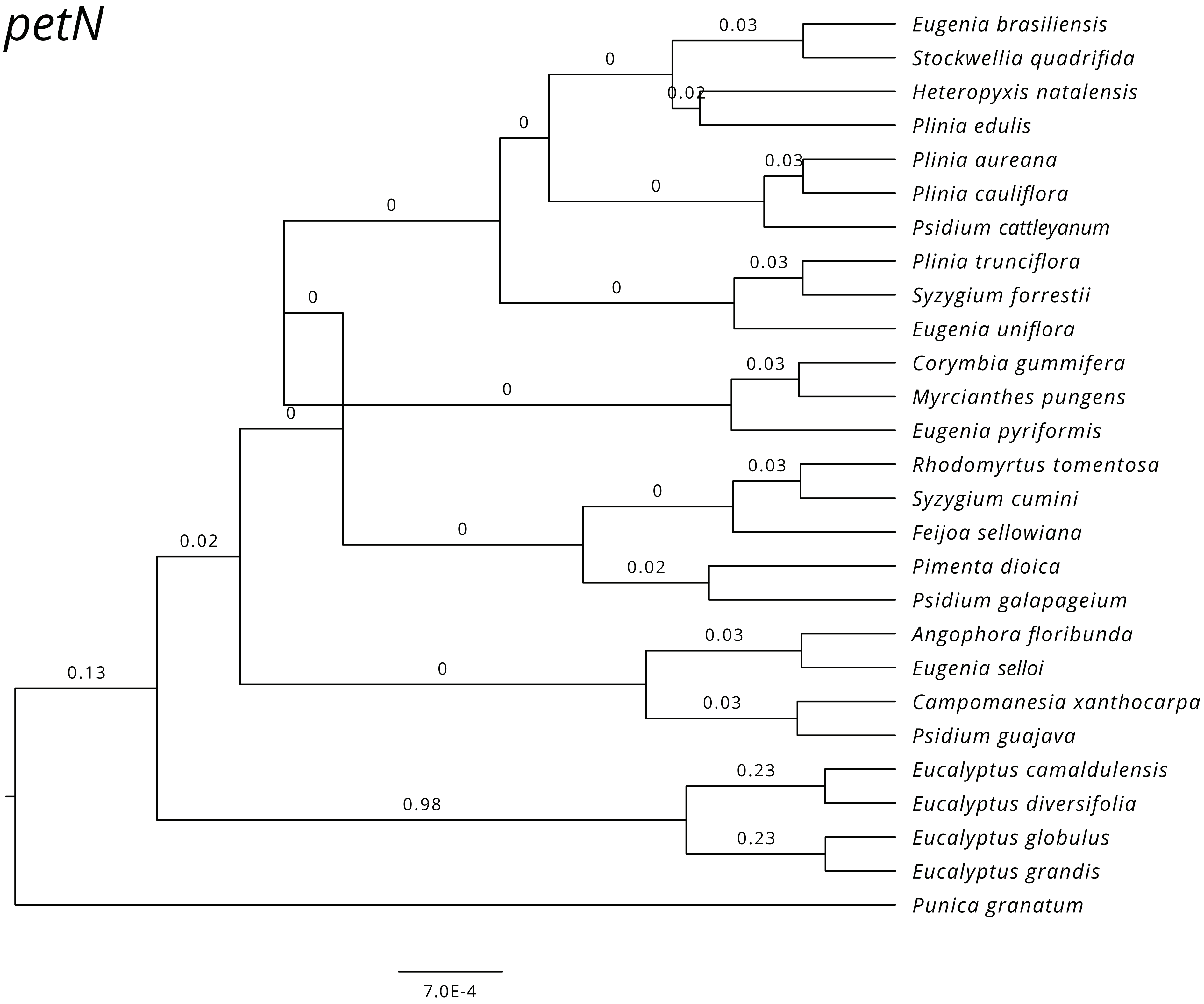
petN
0.03
0
0.02
0
0.03
0
0
0.03
0
0
0.03
0
0
0.03
0
0
0.02
0.02
0
0.03
0.03
0.13
0.23
0.98
0.23
Eugenia brasiliensis
Stockwellia quadrifida
Heteropyxis natalensis
Plinia edulis
Plinia aureana
Plinia cauliflora
Psidium cattleyanum
Plinia trunciflora
Syzygium forrestii
Eugenia uniflora
Corymbia gummifera
Myrcianthes pungens
Eugenia pyriformis
Rhodomyrtus tomentosa
Syzygium cumini
Feijoa sellowiana
Pimenta dioica
Psidium galapageium
Angophora floribunda
Eugenia selloi
Campomanesia xanthocarpa
Psidium guajava
Eucalyptus camaldulensis
Eucalyptus diversifolia
Eucalyptus globulus
Eucalyptus grandis
Punica granatum
7.0E-4

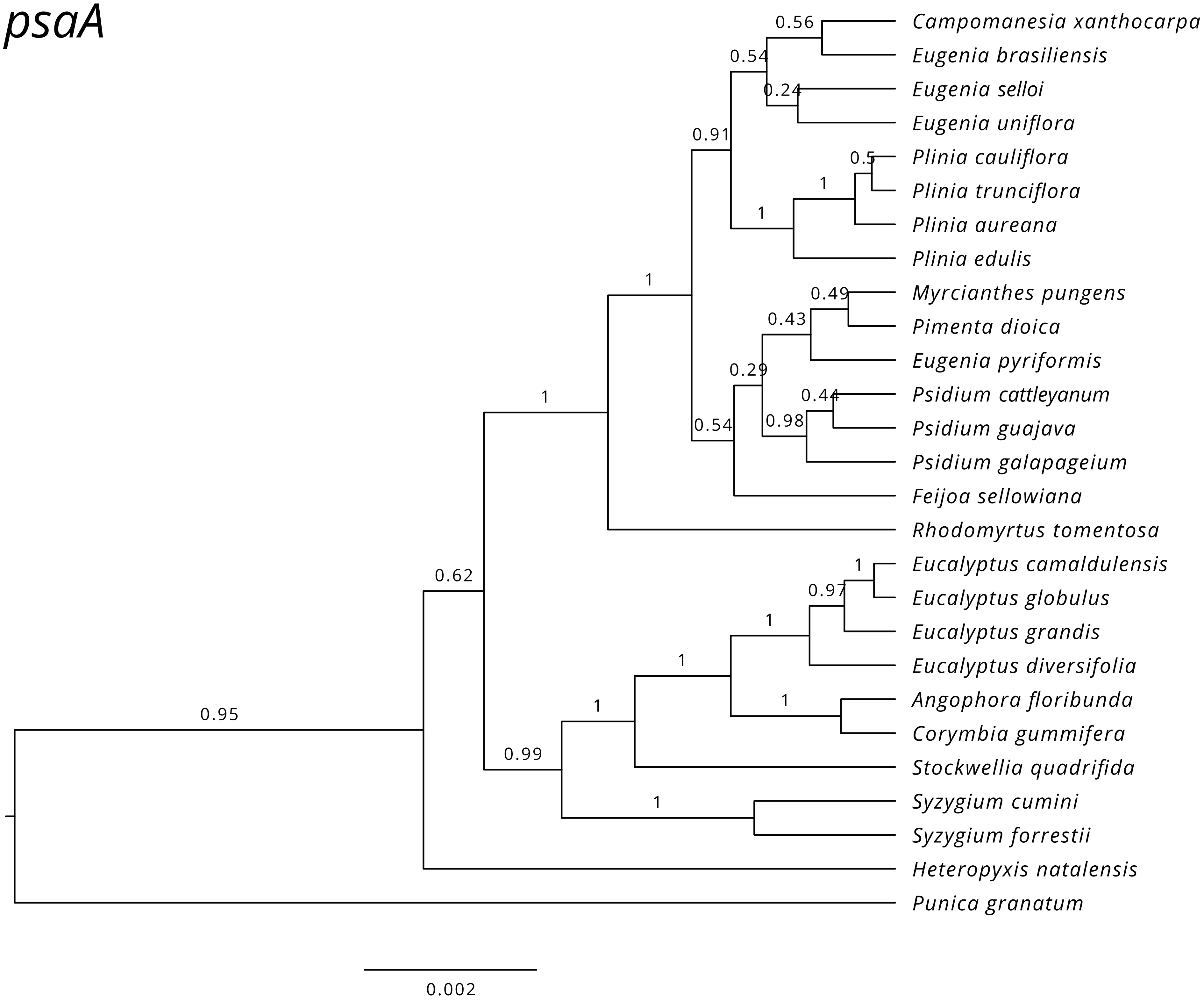
psaA
0.56
0.54
0.24
0.91
0.5
1
1
1
0.49
0.43
0.29
0.44
0.98
0.54
1
0.62
1
0.97
1
1
1
1
0.95
0.99
1
Campomanesia xanthocarpa
Eugenia brasiliensis
Eugenia selloi
Eugenia uniflora
Plinia cauliflora
Plinia trunciflora
Plinia aureana
Plinia edulis
Myrcianthes pungens
Pimenta dioica
Eugenia pyriformis
Psidium cattleyanum
Psidium guajava
Psidium galapageium
Feijoa sellowiana
Rhodomyrtus tomentosa
Eucalyptus camaldulensis
Eucalyptus globulus
Eucalyptus grandis
Eucalyptus diversifolia
Angophora floribunda
Corymbia gummifera
Stockwellia quadrifida
Syzygium cumini
Syzygium forrestii
Heteropyxis natalensis
Punica granatum
0.002

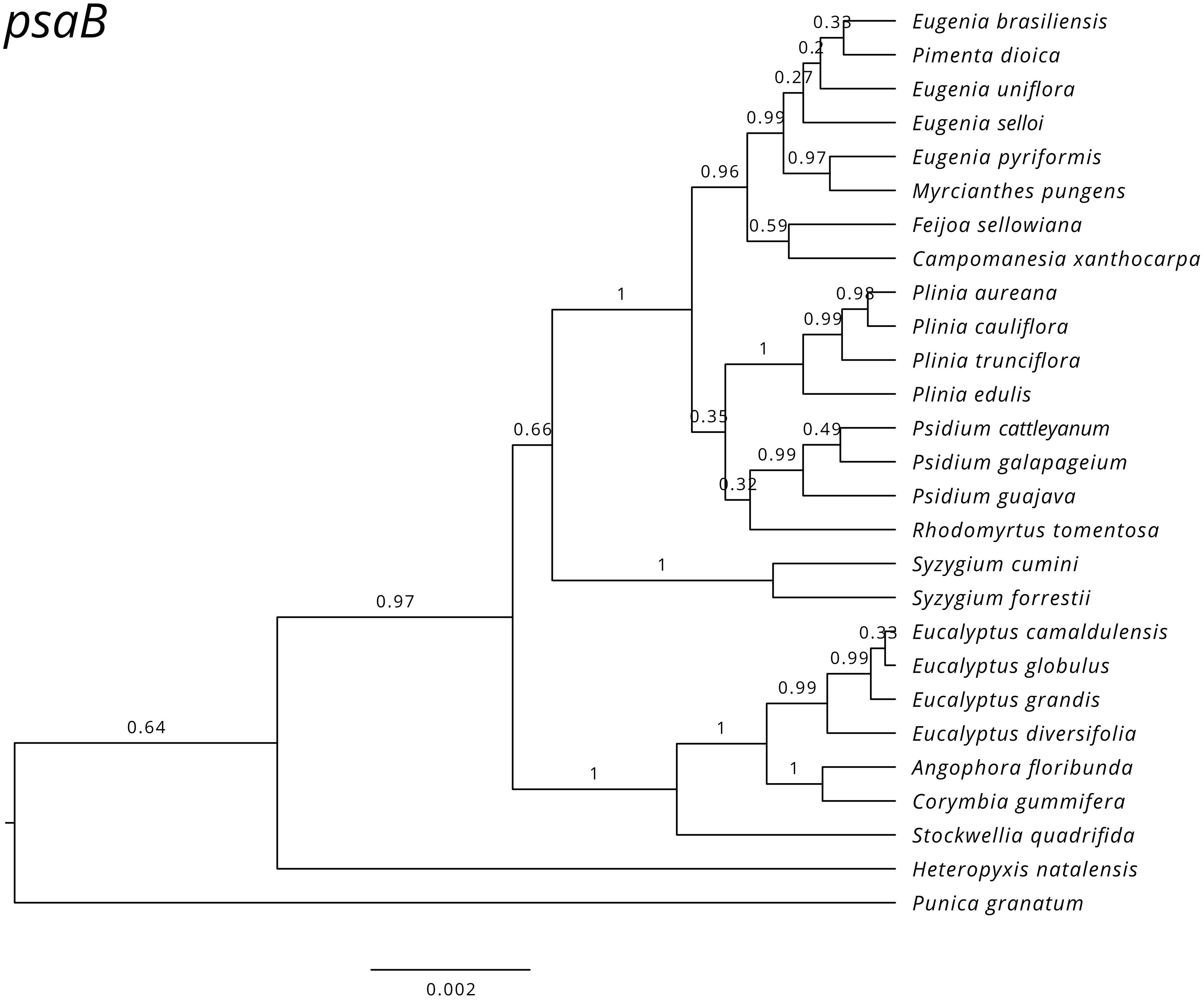

psaB
Eugenia brasiliensis
Pimenta dioica
Eugenia uniflora
Eugenia selloi
Eugenia pyriformis
Myrcianthes pungens
Feijoa sellowiana
Campomanesia xanthocarpa
Plinia aureana
Plinia cauliflora
Plinia trunciflora
Plinia edulis
Psidium cattleyanum
Psidium galapageium
Psidium guajava
Rhodomyrtus tomentosa
Syzygium cumini
Syzygium forrestii
Eucalyptus camaldulensis
Eucalyptus globulus
Eucalyptus grandis
Eucalyptus diversifolia
Angophora floribunda
Corymbia gummifera
Stockwellia quadrifida
Heteropyxis natalensis
Punica granatum
0.33
0.2
0.27
0.99
0.97
0.96
0.59
1
0.98
0.99
1
0.35
0.49
0.99
0.32
0.66
1
0.97
0.33
0.99
0.99
1
1
1
0.64
0.002

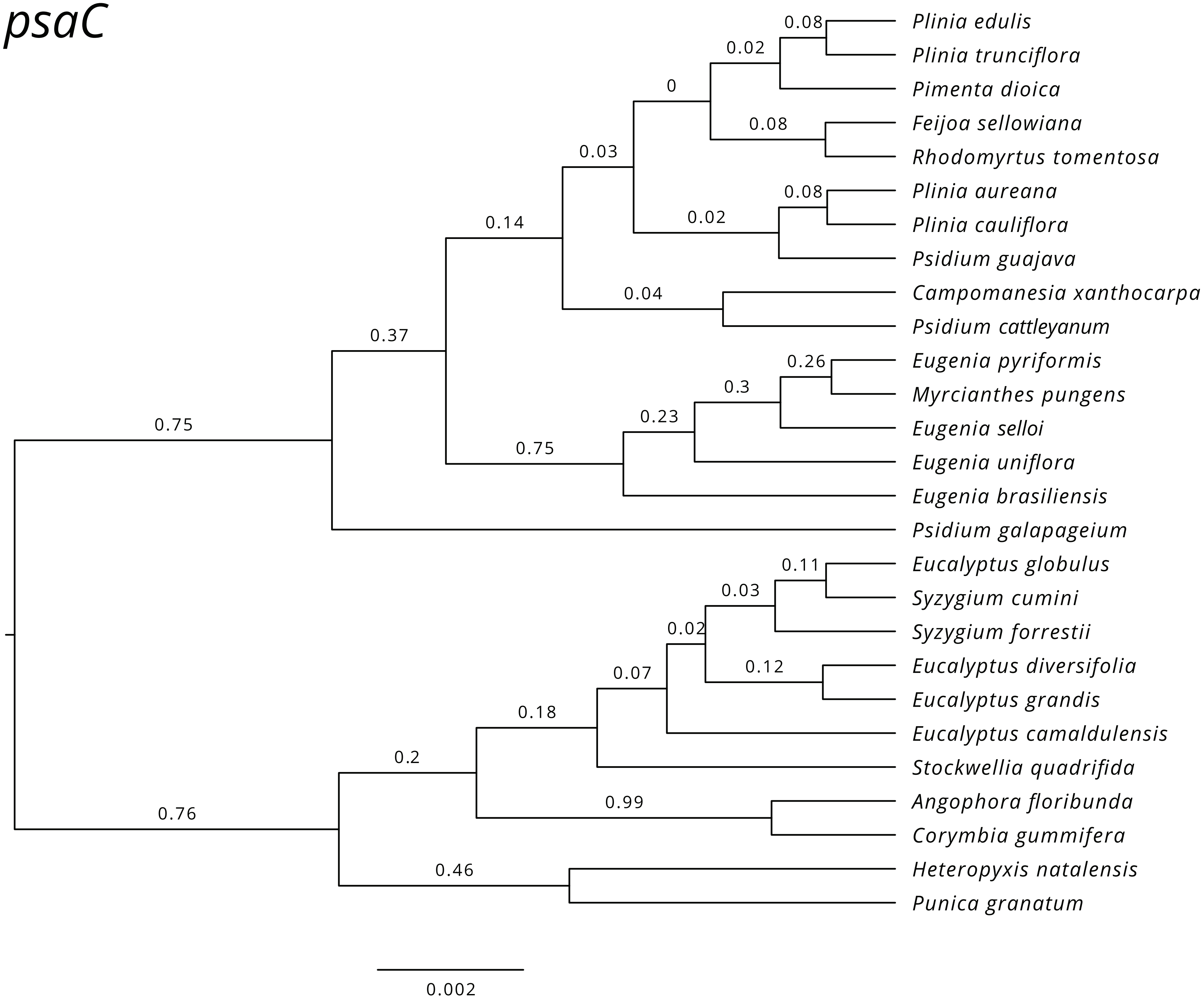
psaC
Plinia edulis
Plinia trunciflora
Pimenta dioica
Feijoa sellowiana
Rhodomyrtus tomentosa
Plinia aureana
Plinia cauliflora
Psidium guajava
Campomanesia xanthocarpa
Psidium cattleyanum
Eugenia pyriformis
Myrcianthes pungens
Eugenia selloi
Eugenia uniflora
Eugenia brasiliensis
Psidium galapageium
Eucalyptus globulus
Syzygium cumini
Syzygium forrestii
Eucalyptus diversifolia
Eucalyptus grandis
Eucalyptus camaldulensis
Stockwellia quadrifida
Angophora floribunda
Corymbia gummifera
Heteropyxis natalensis
Punica granatum
0.08
0.02
0
0.08
0.03
0.08
0.02
0.14
0.04
0.37
0.26
0.3
0.23
0.75
0.75
0.11
0.03
0.02
0.12
0.07
0.18
0.2
0.99
0.76
0.46
0.002

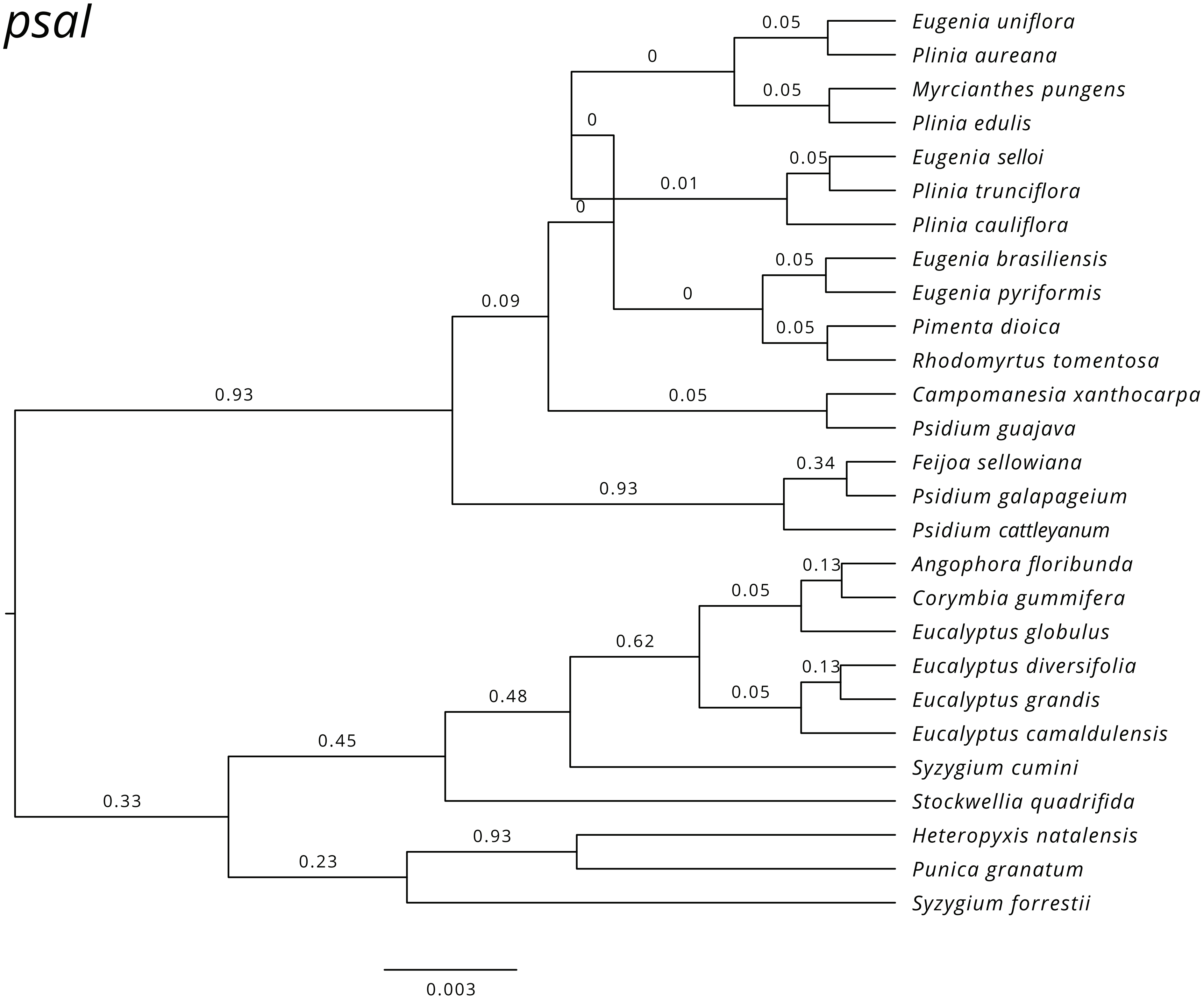
psal
0.05
0
0.05
0
0.05
0.01
0
0.05
0
0.09
0.05
0.93
0.05
0.34
0.93
0.13
0.05
0.62
0.13
0.48
0.05
0.45
0.33
0.93
0.23
Eugenia uniflora
Plinia aureana
Myrcianthes pungens
Plinia edulis
Eugenia selloi
Plinia trunciflora
Plinia cauliflora
Eugenia brasiliensis
Eugenia pyriformis
Pimenta dioica
Rhodomyrtus tomentosa
Campomanesia xanthocarpa
Psidium guajava
Feijoa sellowiana
Psidium galapageium
Psidium cattleyanum
Angophora floribunda
Corymbia gummifera
Eucalyptus globulus
Eucalyptus diversifolia
Eucalyptus grandis
Eucalyptus camaldulensis
Syzygium cumini
Stockwellia quadrifida
Heteropyxis natalensis
Punica granatum
Syzygium forrestii
0.003

# psaJ

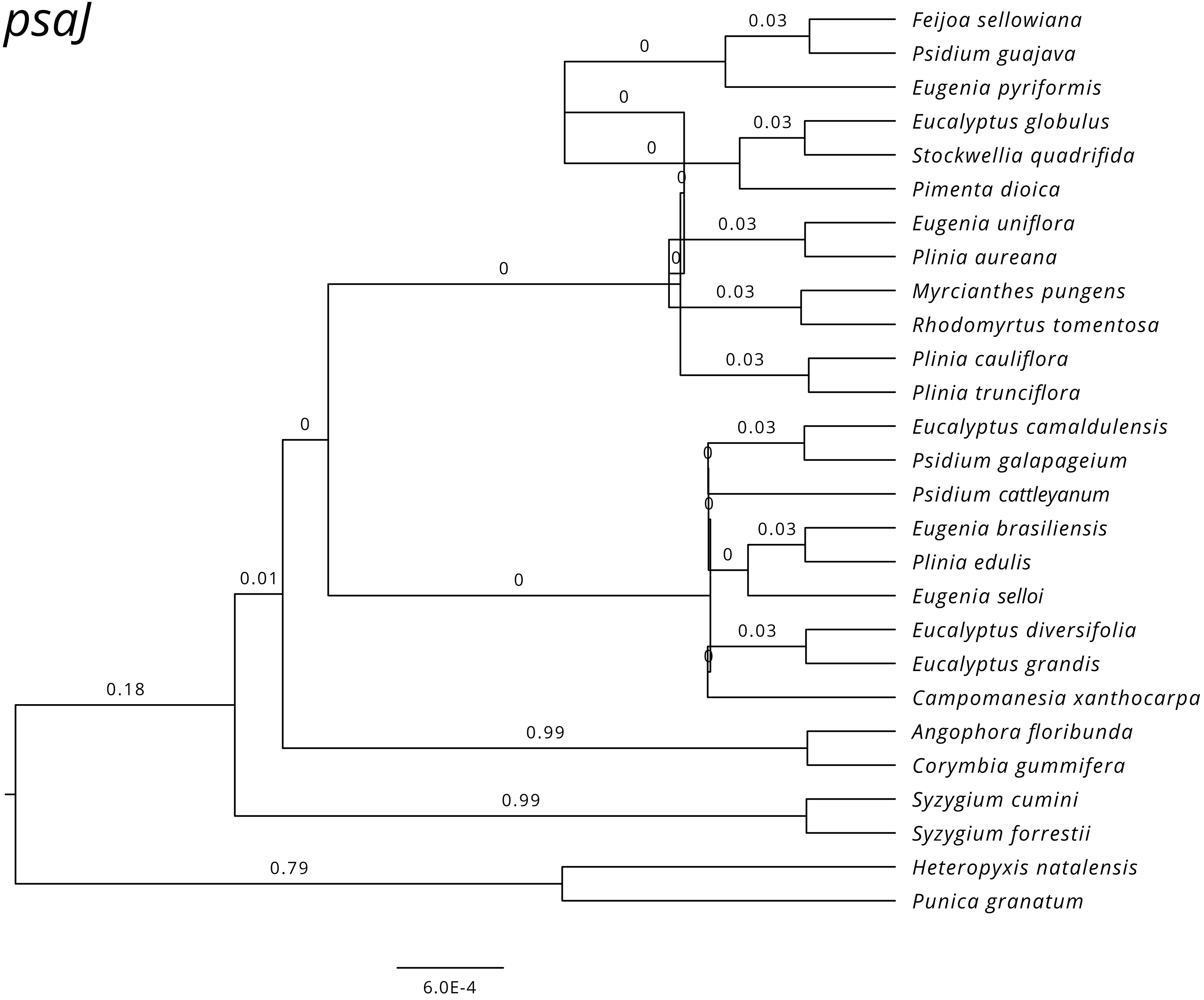

Feijoa sellowiana
Psidium guajava
Eugenia pyriformis
Eucalyptus globulus
Stockwellia quadrifida
Pimenta dioica
Eugenia uniflora
Plinia aureana
Myrcianthes pungens
Rhodomyrtus tomentosa
Plinia cauliflora
Plinia trunciflora
Eucalyptus camaldulensis
Psidium galapageium
Psidium cattleyanum
Eugenia brasiliensis
Plinia edulis
Eugenia selloi
Eucalyptus diversifolia
Eucalyptus grandis
Campomanesia xanthocarpa
Angophora floribunda
Corymbia gummifera
Syzygium cumini
Syzygium forrestii
Heteropyxis natalensis
Punica granatum
0.03
0
0.18
0.01
0.99
0.79
6.0E-4

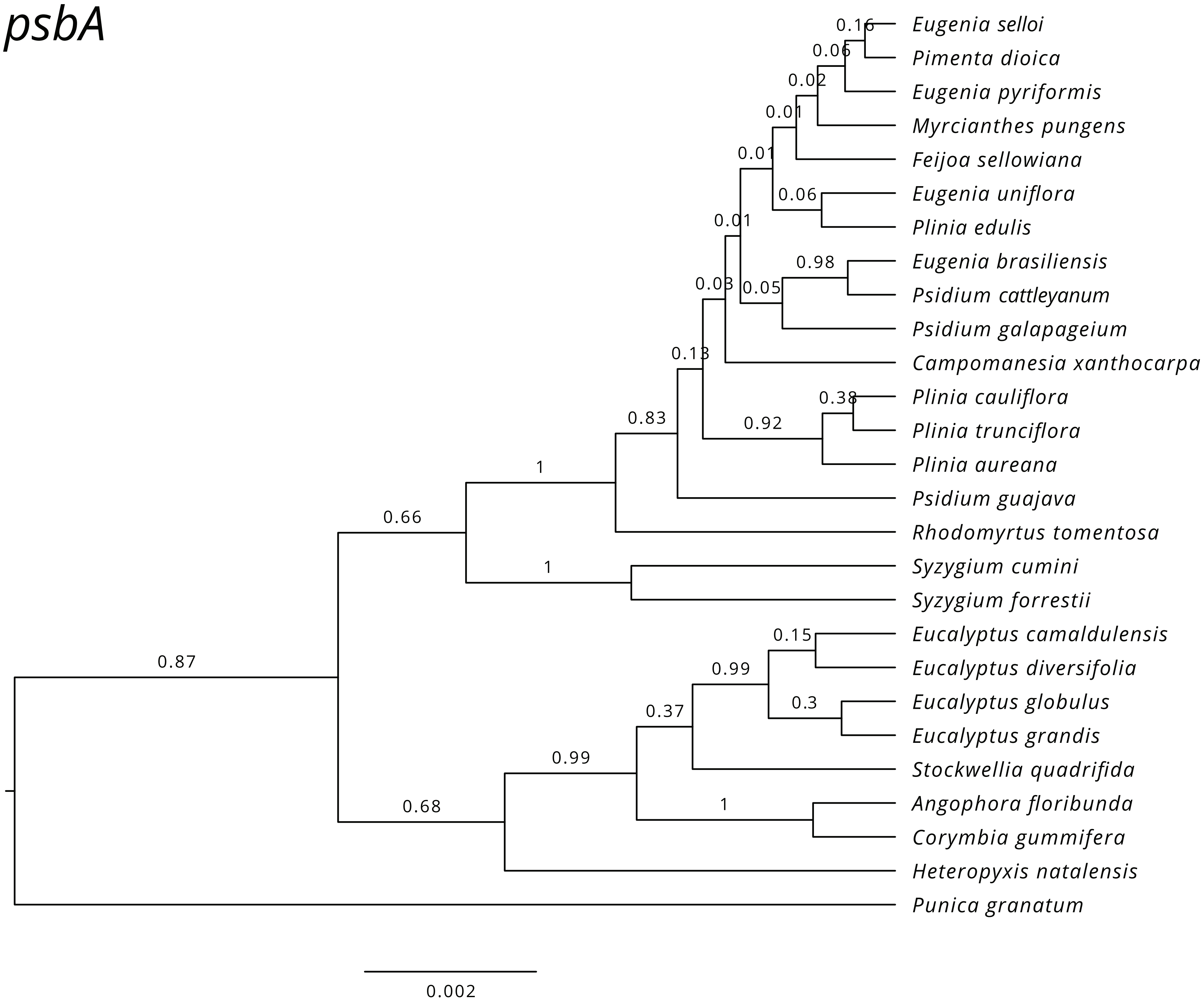
psbA
Eugenia selloi
Pimenta dioica
Eugenia pyriformis
Myrcianthes pungens
Feijoa sellowiana
Eugenia uniflora
Plinia edulis
Eugenia brasiliensis
Psidium cattleyanum
Psidium galapageium
Campomanesia xanthocarpa
Plinia cauliflora
Plinia trunciflora
Plinia aureana
Psidium guajava
Rhodomyrtus tomentosa
Syzygium cumini
Syzygium forrestii
Eucalyptus camaldulensis
Eucalyptus diversifolia
Eucalyptus globulus
Eucalyptus grandis
Stockwellia quadrifida
Angophora floribunda
Corymbia gummifera
Heteropyxis natalensis
Punica granatum
0.16
0.06
0.02
0.01
0.01
0.06
0.01
0.98
0.03
0.05
0.13
0.38
0.83
0.92
1
0.66
1
0.15
0.87
0.99
0.3
0.37
0.99
0.68
1
0.002

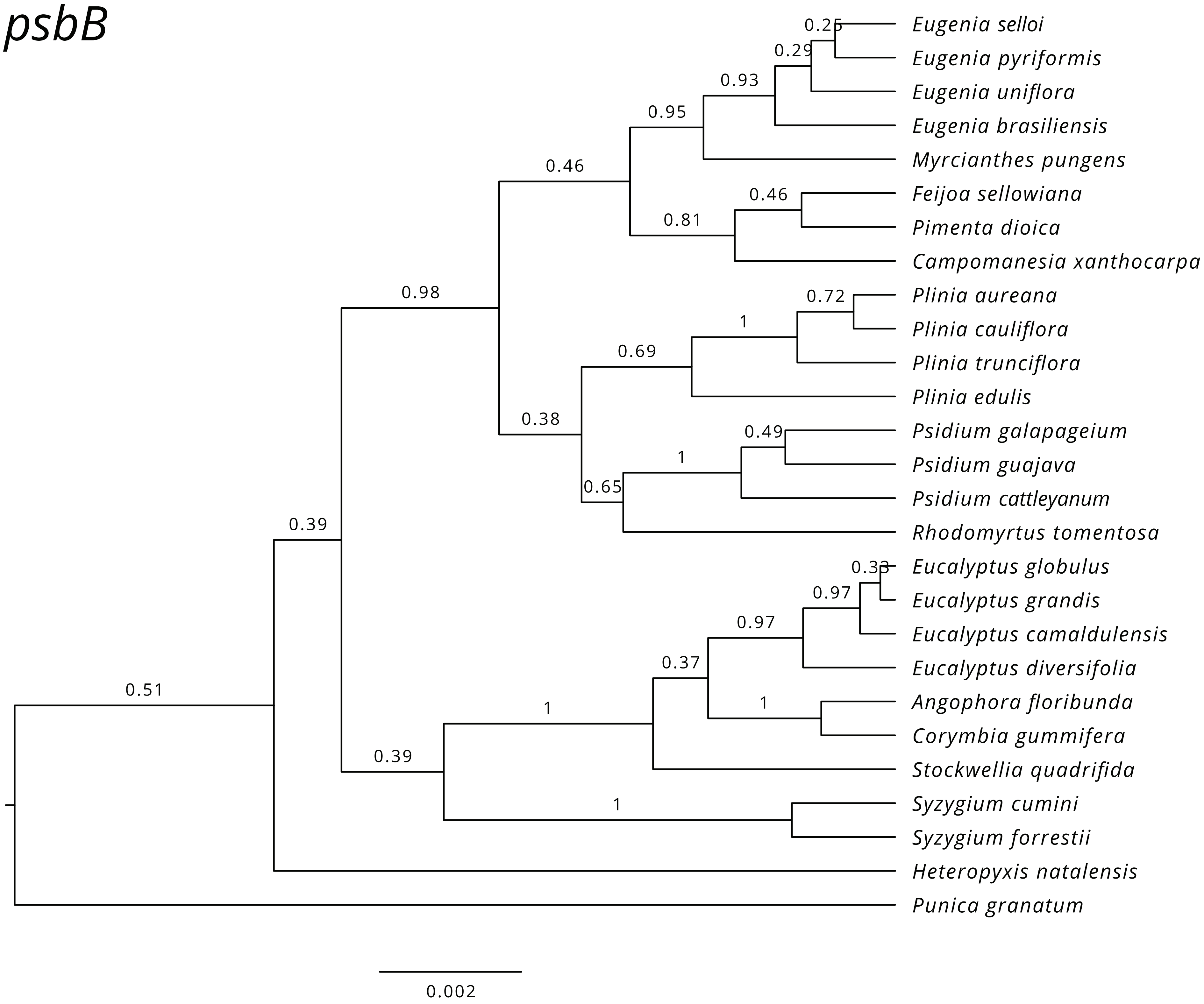
psbB
0.25
0.29
0.93
0.95
0.46
0.46
0.81
0.98
0.72
1
0.69
0.38
0.49
1
0.65
0.39
0.33
0.97
0.97
0.37
1
1
0.51
0.39
1
Eugenia selloi
Eugenia pyriformis
Eugenia uniflora
Eugenia brasiliensis
Myrcianthes pungens
Feijoa sellowiana
Pimenta dioica
Campomanesia xanthocarpa
Plinia aureana
Plinia cauliflora
Plinia trunciflora
Plinia edulis
Psidium galapageium
Psidium guajava
Psidium cattleyanum
Rhodomyrtus tomentosa
Eucalyptus globulus
Eucalyptus grandis
Eucalyptus camaldulensis
Eucalyptus diversifolia
Angophora floribunda
Corymbia gummifera
Stockwellia quadrifida
Syzygium cumini
Syzygium forrestii
Heteropyxis natalensis
Punica granatum
0.002

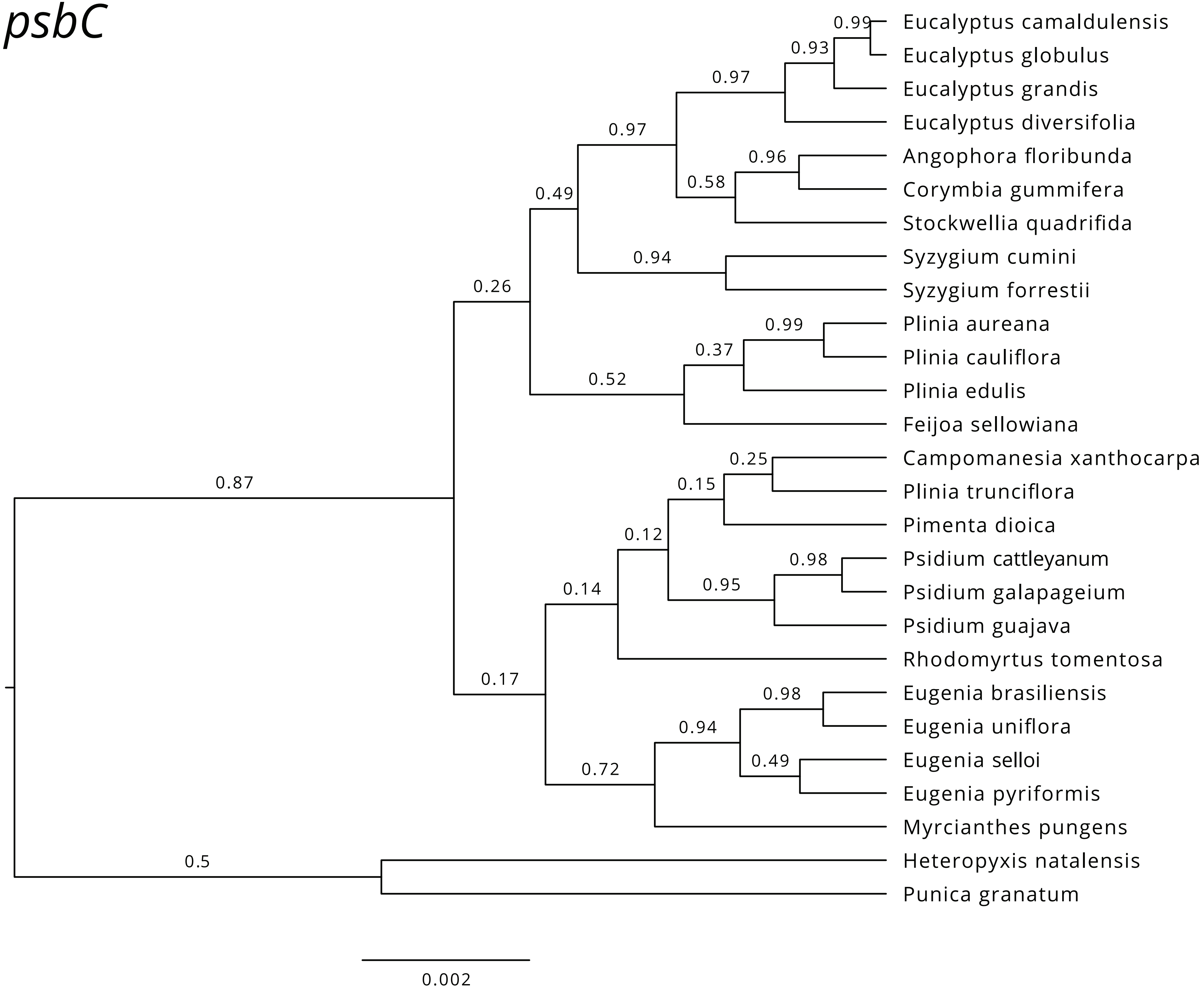
psbC
Eucalyptus camaldulensis
Eucalyptus globulus
Eucalyptus grandis
Eucalyptus diversifolia
Angophora floribunda
Corymbia gummifera
Stockwellia quadrifida
Syzygium cumini
Syzygium forrestii
Plinia aureana
Plinia cauliflora
Plinia edulis
Feijoa sellowiana
Campomanesia xanthocarpa
Plinia trunciflora
Pimenta dioica
Psidium cattleyanum
Psidium galapageium
Psidium guajava
Rhodomyrtus tomentosa
Eugenia brasiliensis
Eugenia uniflora
Eugenia selloi
Eugenia pyriformis
Myrcianthes pungens
Heteropyxis natalensis
Punica granatum
0.99
0.93
0.97
0.97
0.96
0.58
0.49
0.94
0.26
0.99
0.37
0.52
0.87
0.25
0.15
0.12
0.98
0.95
0.14
0.17
0.98
0.94
0.49
0.72
0.5
0.002

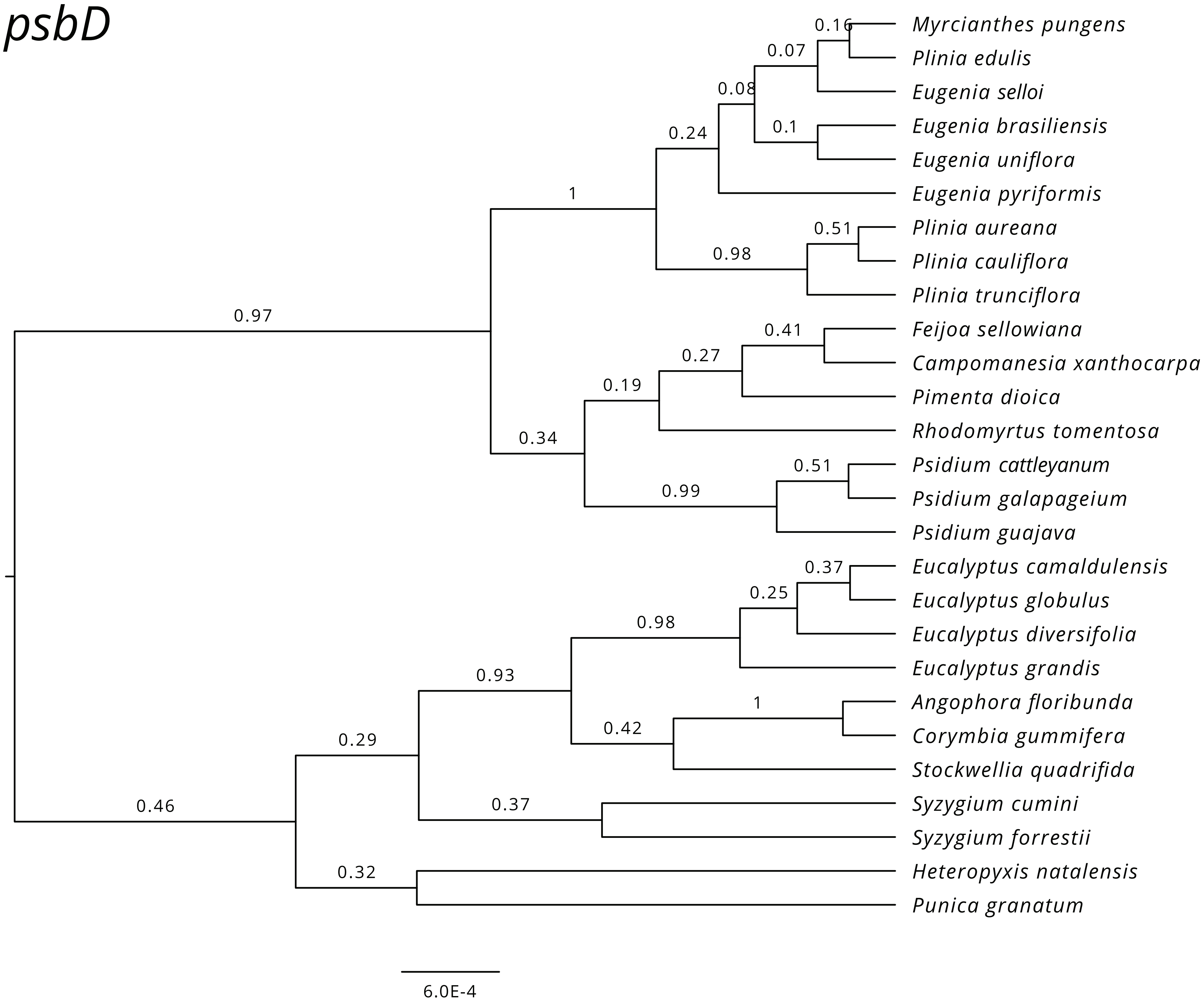
psbD
0.16
Myrcianthes pungens
0.07
Plinia edulis
0.08
Eugenia selloi
0.24
0.1
Eugenia brasiliensis
Eugenia uniflora
1
Eugenia pyriformis
0.51
Plinia aureana
0.98
Plinia cauliflora
Plinia trunciflora
0.97
0.41
Feijoa sellowiana
0.27
Campomanesia xanthocarpa
0.19
Pimenta dioica
0.34
Rhodomyrtus tomentosa
0.51
Psidium cattleyanum
0.99
Psidium galapageium
Psidium guajava
0.37
Eucalyptus camaldulensis
0.25
Eucalyptus globulus
0.98
Eucalyptus diversifolia
Eucalyptus grandis
0.93
1
Angophora floribunda
0.42
Corymbia gummifera
0.29
Stockwellia quadrifida
0.46
0.37
Syzygium cumini
Syzygium forrestii
0.32
Heteropyxis natalensis
Punica granatum
6.0E-4

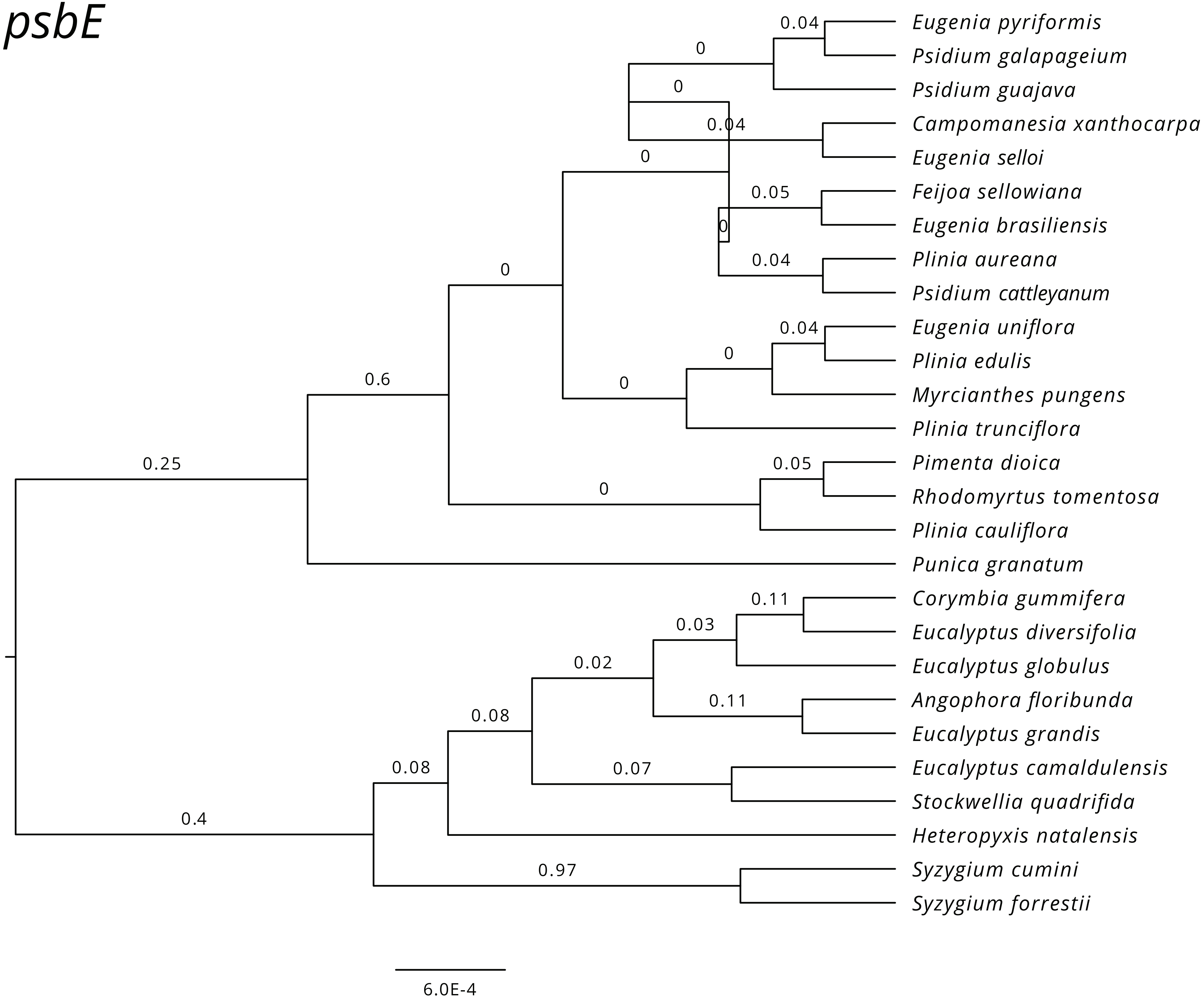
psbE
0.04
0
0
0.04
0
0.05
0
0.04
0
0.04
0
0
0.6
0.05
0
0.25
0.11
0.03
0.02
0.11
0.08
0.07
0.08
0.4
0.97
6.0E-4
Eugenia pyriformis
Psidium galapageium
Psidium guajava
Campomanesia xanthocarpa
Eugenia selloi
Feijoa sellowiana
Eugenia brasiliensis
Plinia aureana
Psidium cattleyanum
Eugenia uniflora
Plinia edulis
Myrcianthes pungens
Plinia trunciflora
Pimenta dioica
Rhodomyrtus tomentosa
Plinia cauliflora
Punica granatum
Corymbia gummifera
Eucalyptus diversifolia
Eucalyptus globulus
Angophora floribunda
Eucalyptus grandis
Eucalyptus camaldulensis
Stockwellia quadrifida
Heteropyxis natalensis
Syzygium cumini
Syzygium forrestii

# *psbF*

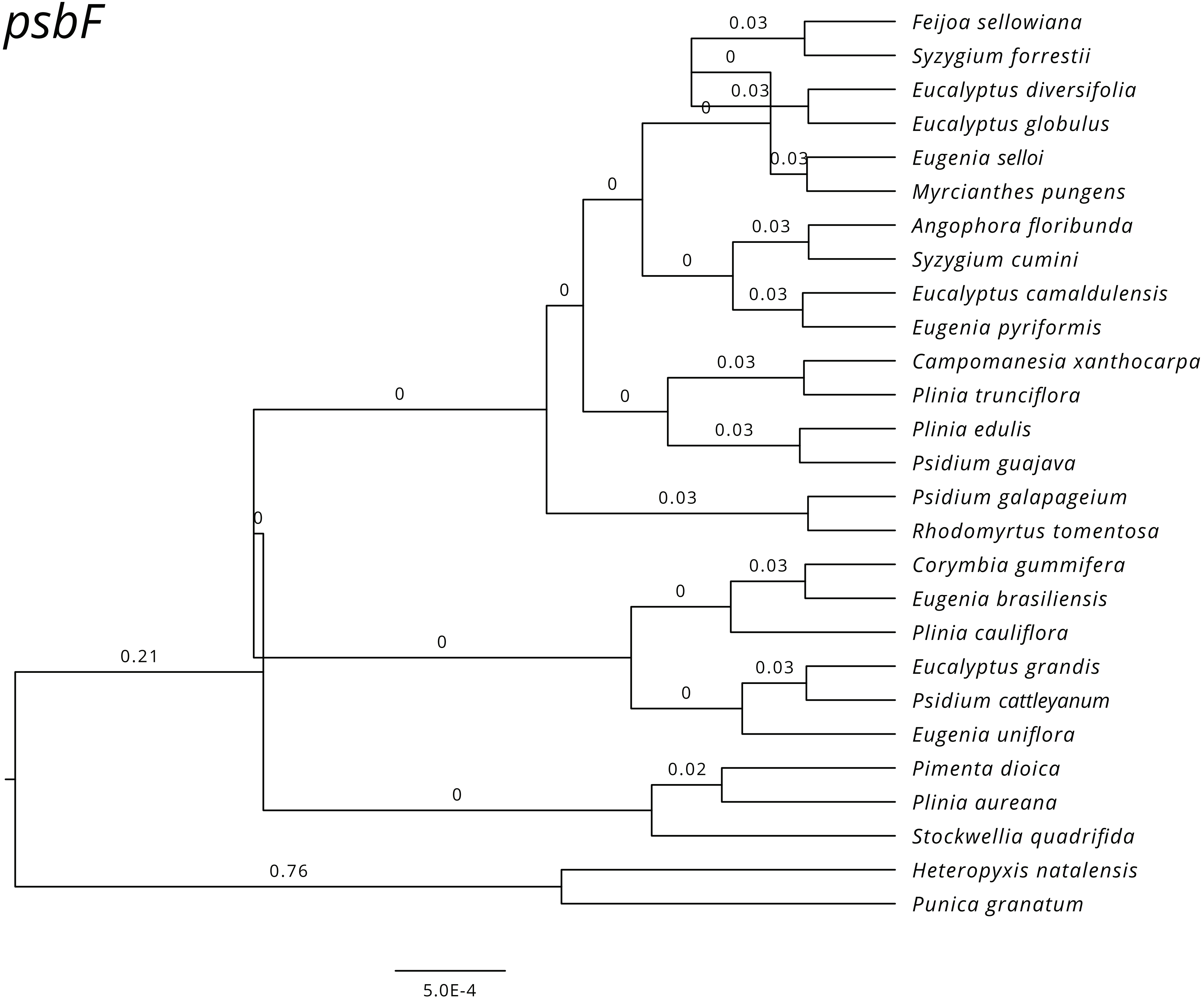

0.03
Feijoa sellowiana
Syzygium forrestii
0
0.03
Eucalyptus diversifolia
0
Eucalyptus globulus
0.03
Eugenia selloi
Myrcianthes pungens
0
0.03
Angophora floribunda
Syzygium cumini
0
0.03
Eucalyptus camaldulensis
0
Eugenia pyriformis
0.03
Campomanesia xanthocarpa
0
Plinia trunciflora
0
0.03
Plinia edulis
Psidium guajava
0.03
Psidium galapageium
0
Rhodomyrtus tomentosa
0.03
Corymbia gummifera
0
Eugenia brasiliensis
0
Plinia cauliflora
0.03
Eucalyptus grandis
0
Psidium cattleyanum
0.21
Eugenia uniflora
0.02
Pimenta dioica
0
Plinia aureana
Stockwellia quadrifida
0.76
Heteropyxis natalensis
Punica granatum
5.0E-4

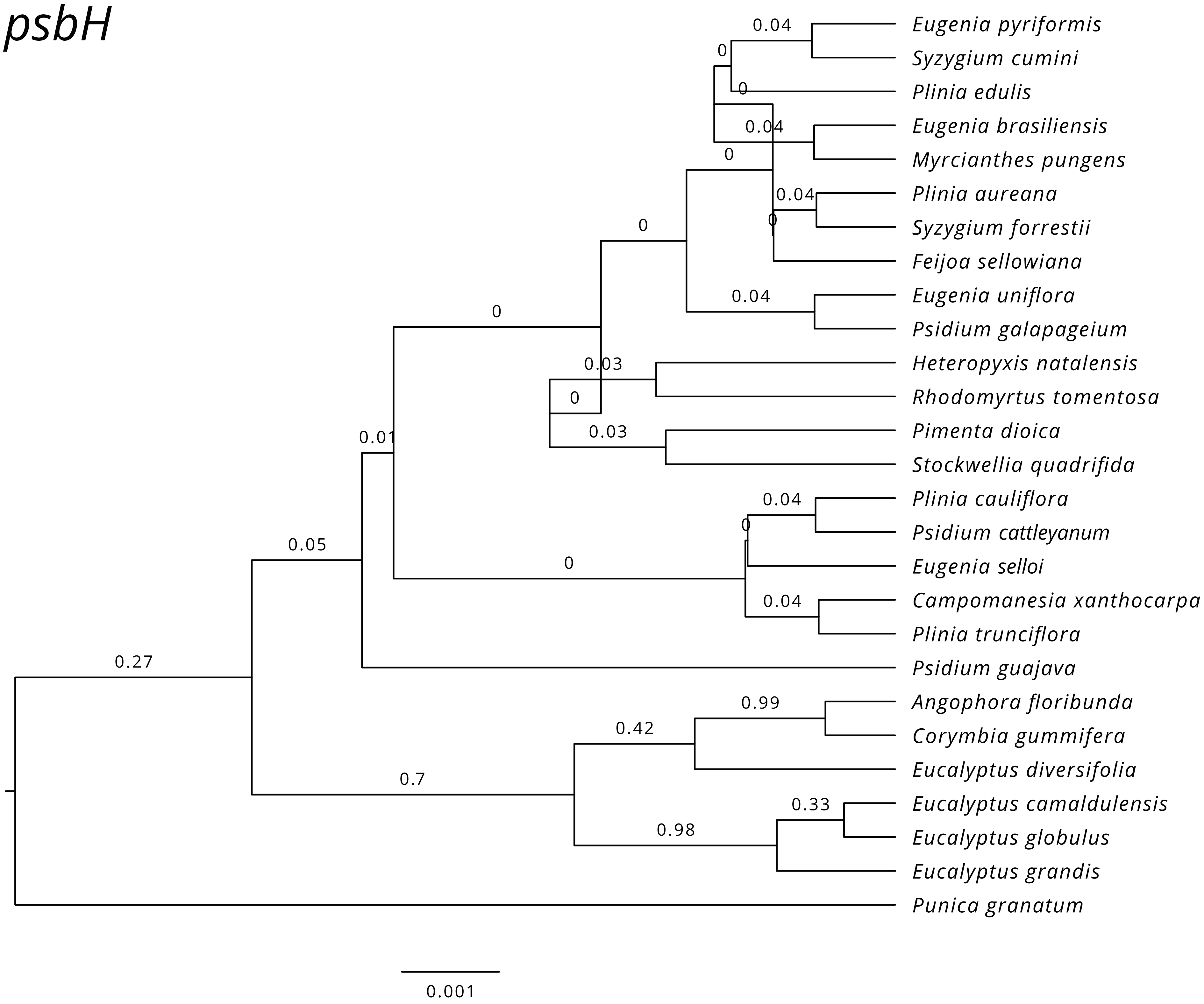
psbH
0.04
0
0
0.04
0
0.04
0
0
0.04
0
0.03
0
0.03
0.01
0.04
0
0
0.04
0.05
0.27
0.99
0.42
0.7
0.33
0.98
0.001
Eugenia pyriformis
Syzygium cumini
Plinia edulis
Eugenia brasiliensis
Myrcianthes pungens
Plinia aureana
Syzygium forrestii
Feijoa sellowiana
Eugenia uniflora
Psidium galapageium
Heteropyxis natalensis
Rhodomyrtus tomentosa
Pimenta dioica
Stockwellia quadrifida
Plinia cauliflora
Psidium cattleyanum
Eugenia selloi
Campomanesia xanthocarpa
Plinia trunciflora
Psidium guajava
Angophora floribunda
Corymbia gummifera
Eucalyptus diversifolia
Eucalyptus camaldulensis
Eucalyptus globulus
Eucalyptus grandis
Punica granatum

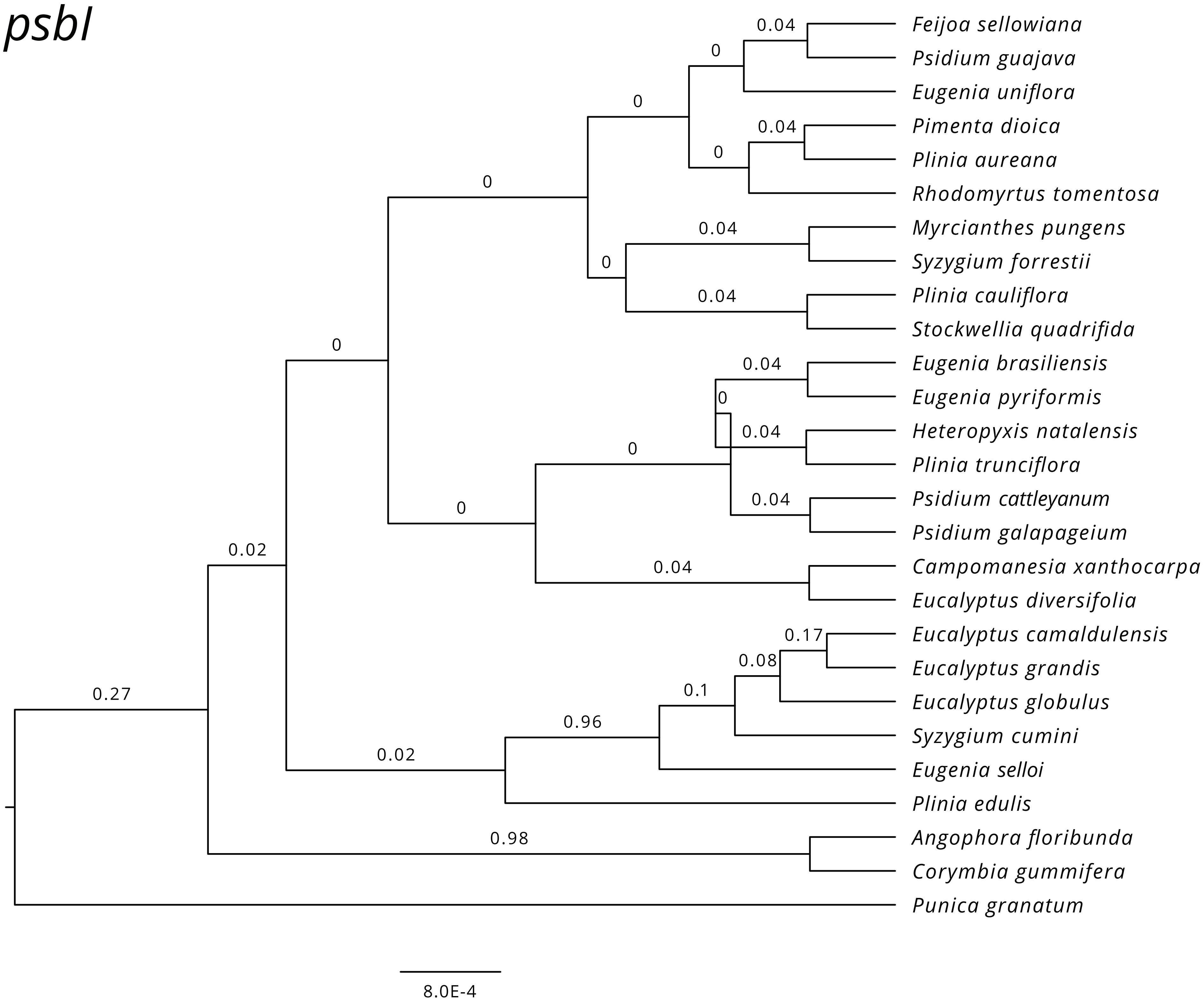
psbI
Feijoa sellowiana
Psidium guajava
Eugenia uniflora
Pimenta dioica
Plinia aureana
Rhodomyrtus tomentosa
Myrcianthes pungens
Syzygium forrestii
Plinia cauliflora
Stockwellia quadrifida
Eugenia brasiliensis
Eugenia pyriformis
Heteropyxis natalensis
Plinia trunciflora
Psidium cattleyanum
Psidium galapageium
Campomanesia xanthocarpa
Eucalyptus diversifolia
Eucalyptus camaldulensis
Eucalyptus grandis
Eucalyptus globulus
Syzygium cumini
Eugenia selloi
Plinia edulis
Angophora floribunda
Corymbia gummifera
Punica granatum
0.04
0
0
0.04
0
0
0.04
0
0.04
0
0.04
0
0.04
0
0.04
0
0.04
0.02
0.17
0.08
0.1
0.96
0.27
0.02
0.98
8.0E-4

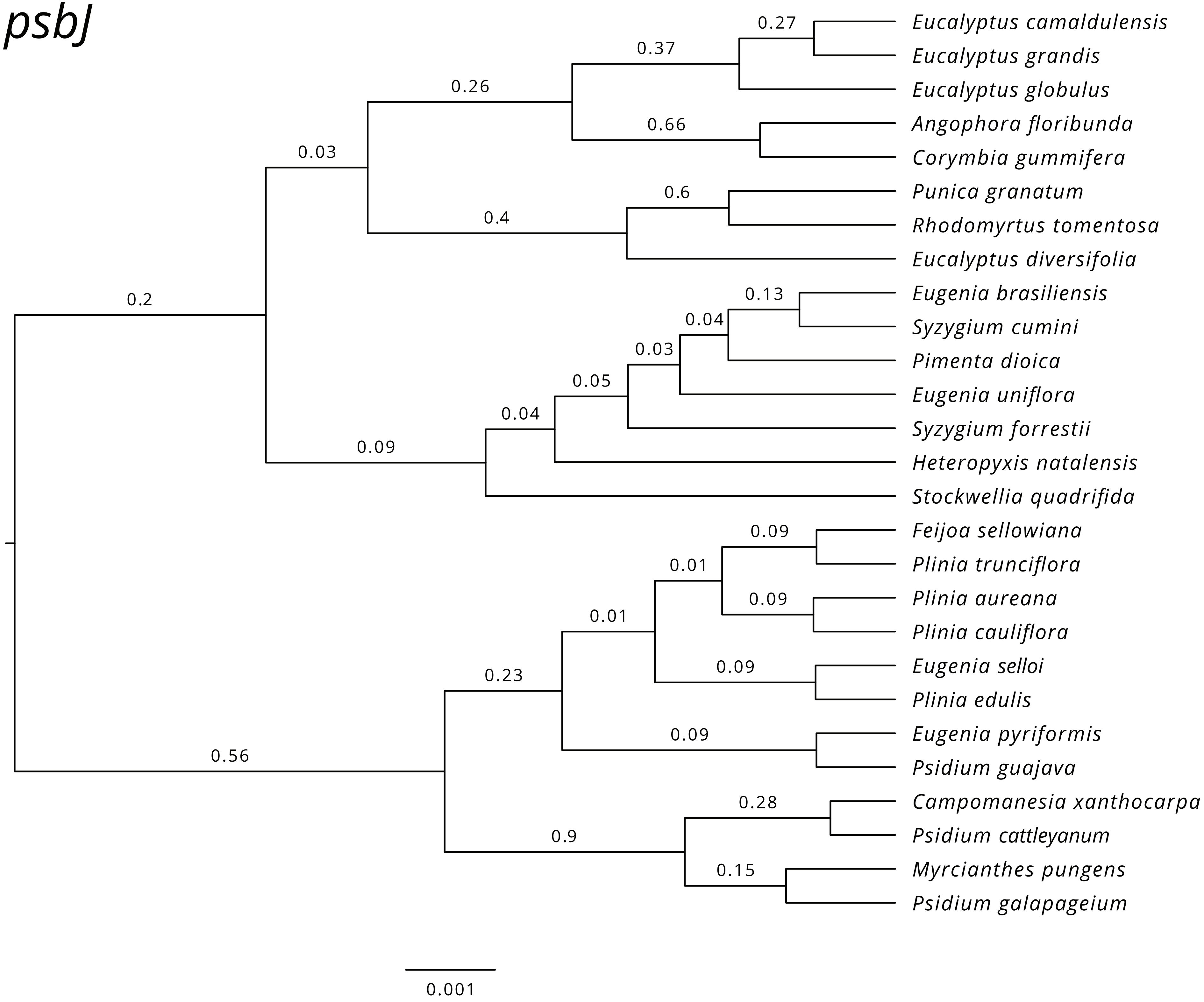
psbJ
0.27
0.37
0.26
0.66
0.03
0.6
0.4
0.2
0.13
0.04
0.03
0.05
0.04
0.09
0.09
0.01
0.09
0.01
0.09
0.23
0.09
0.56
0.28
0.9
0.15
0.001
Eucalyptus camaldulensis
Eucalyptus grandis
Eucalyptus globulus
Angophora floribunda
Corymbia gummifera
Punica granatum
Rhodomyrtus tomentosa
Eucalyptus diversifolia
Eugenia brasiliensis
Syzygium cumini
Pimenta dioica
Eugenia uniflora
Syzygium forrestii
Heteropyxis natalensis
Stockwellia quadrifida
Feijoa sellowiana
Plinia trunciflora
Plinia aureana
Plinia cauliflora
Eugenia selloi
Plinia edulis
Eugenia pyriformis
Psidium guajava
Campomanesia xanthocarpa
Psidium cattleyanum
Myrcianthes pungens
Psidium galapageium

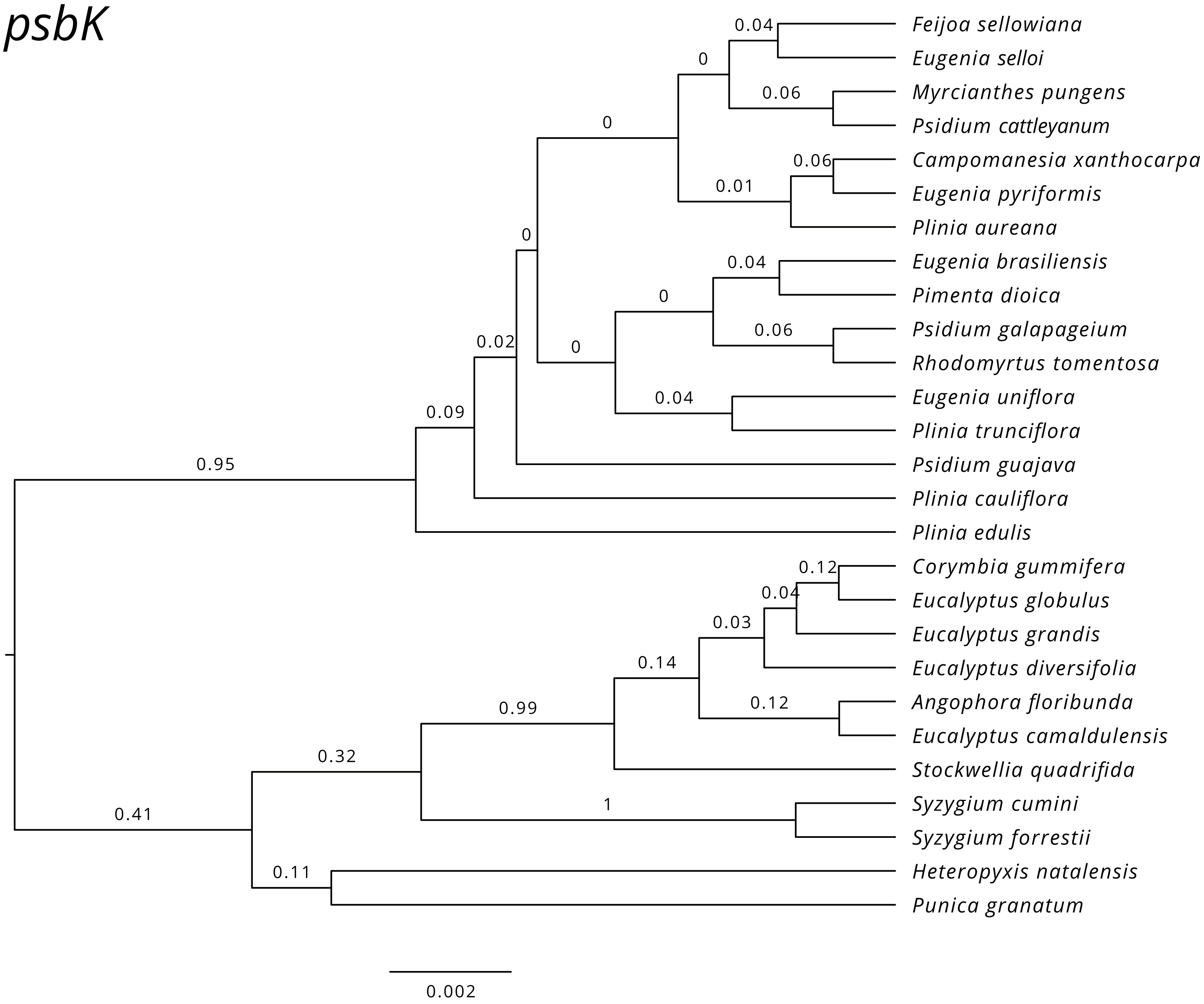
psbK
Feijoa sellowiana
Eugenia selloi
Myrcianthes pungens
Psidium cattleyanum
Campomanesia xanthocarpa
Eugenia pyriformis
Plinia aureana
Eugenia brasiliensis
Pimenta dioica
Psidium galapageium
Rhodomyrtus tomentosa
Eugenia uniflora
Plinia trunciflora
Psidium guajava
Plinia cauliflora
Plinia edulis
Corymbia gummifera
Eucalyptus globulus
Eucalyptus grandis
Eucalyptus diversifolia
Angophora floribunda
Eucalyptus camaldulensis
Stockwellia quadrifida
Syzygium cumini
Syzygium forrestii
Heteropyxis natalensis
Punica granatum
0.04
0
0.06
0
0.06
0.01
0
0.04
0
0.06
0.02
0
0.04
0.09
0.95
0.12
0.04
0.03
0.14
0.12
0.99
0.32
1
0.41
0.11
0.002

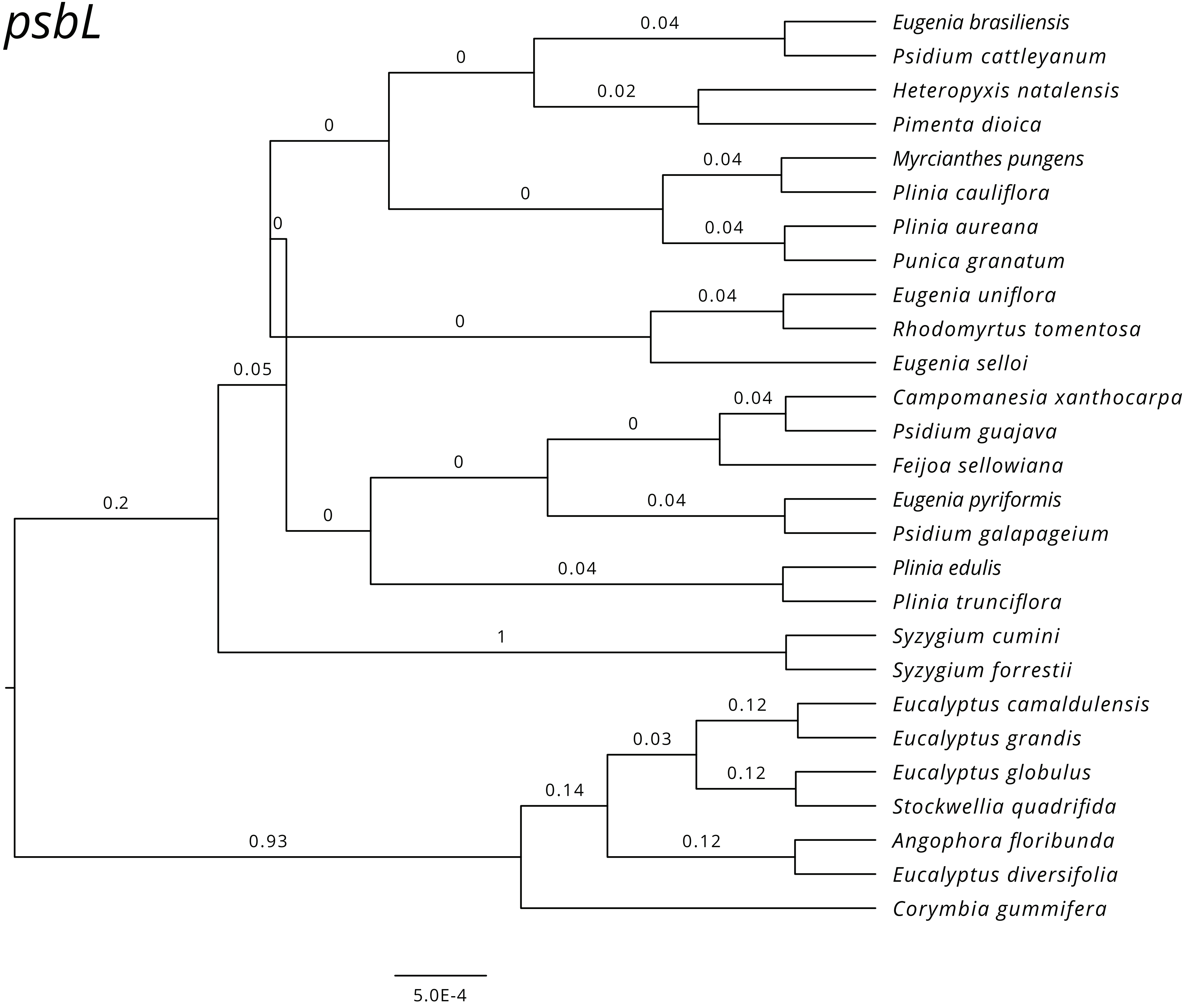
psbL
Eugenia brasiliensis
Psidium cattleyanum
Heteropyxis natalensis
Pimenta dioica
Myrcianthes pungens
Plinia cauliflora
Plinia aureana
Punica granatum
Eugenia uniflora
Rhodomyrtus tomentosa
Eugenia selloi
Campomanesia xanthocarpa
Psidium guajava
Feijoa sellowiana
Eugenia pyriformis
Psidium galapageium
Plinia edulis
Plinia trunciflora
Syzygium cumini
Syzygium forrestii
Eucalyptus camaldulensis
Eucalyptus grandis
Eucalyptus globulus
Stockwellia quadrifida
Angophora floribunda
Eucalyptus diversifolia
Corymbia gummifera
0.04
0
0.02
0
0.04
0
0.04
0
0.04
0
0.05
0.04
0
0
0.04
0
0.04
0.2
1
0.12
0.03
0.12
0.14
0.12
0.93
5.0E-4

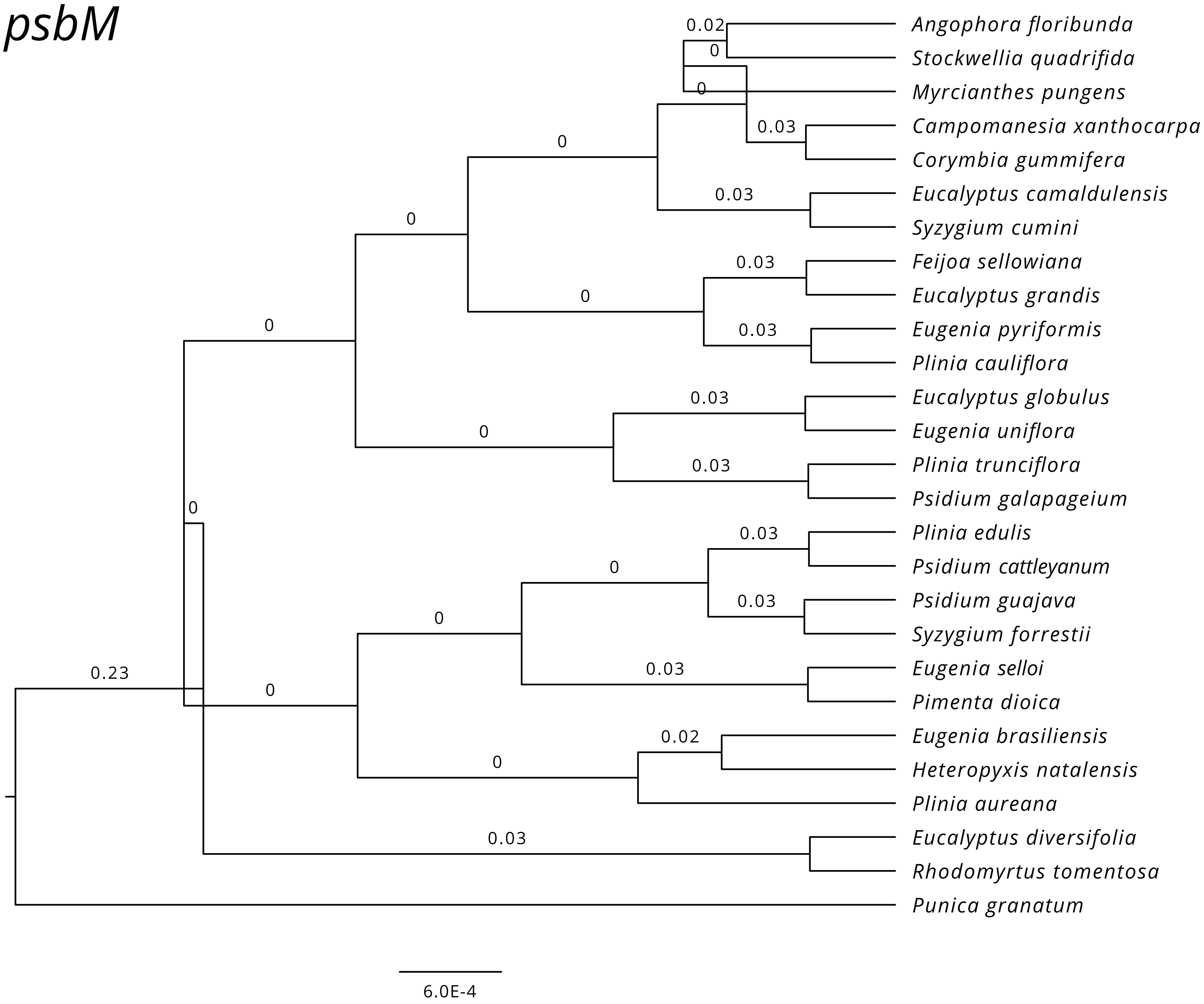
psbM
Angophora floribunda
Stockwellia quadrifida
Myrcianthes pungens
Campomanesia xanthocarpa
Corymbia gummifera
Eucalyptus camaldulensis
Syzygium cumini
Feijoa sellowiana
Eucalyptus grandis
Eugenia pyriformis
Plinia cauliflora
Eucalyptus globulus
Eugenia uniflora
Plinia trunciflora
Psidium galapageium
Plinia edulis
Psidium cattleyanum
Psidium guajava
Syzygium forrestii
Eugenia selloi
Pimenta dioica
Eugenia brasiliensis
Heteropyxis natalensis
Plinia aureana
Eucalyptus diversifolia
Rhodomyrtus tomentosa
Punica granatum
0.02
0
0
0.03
0
0.03
0
0.03
0
0.03
0
0.03
0
0.03
0
0.03
0
0.03
0
0.03
0
0.02
0
0.03
0.23
6.0E-4

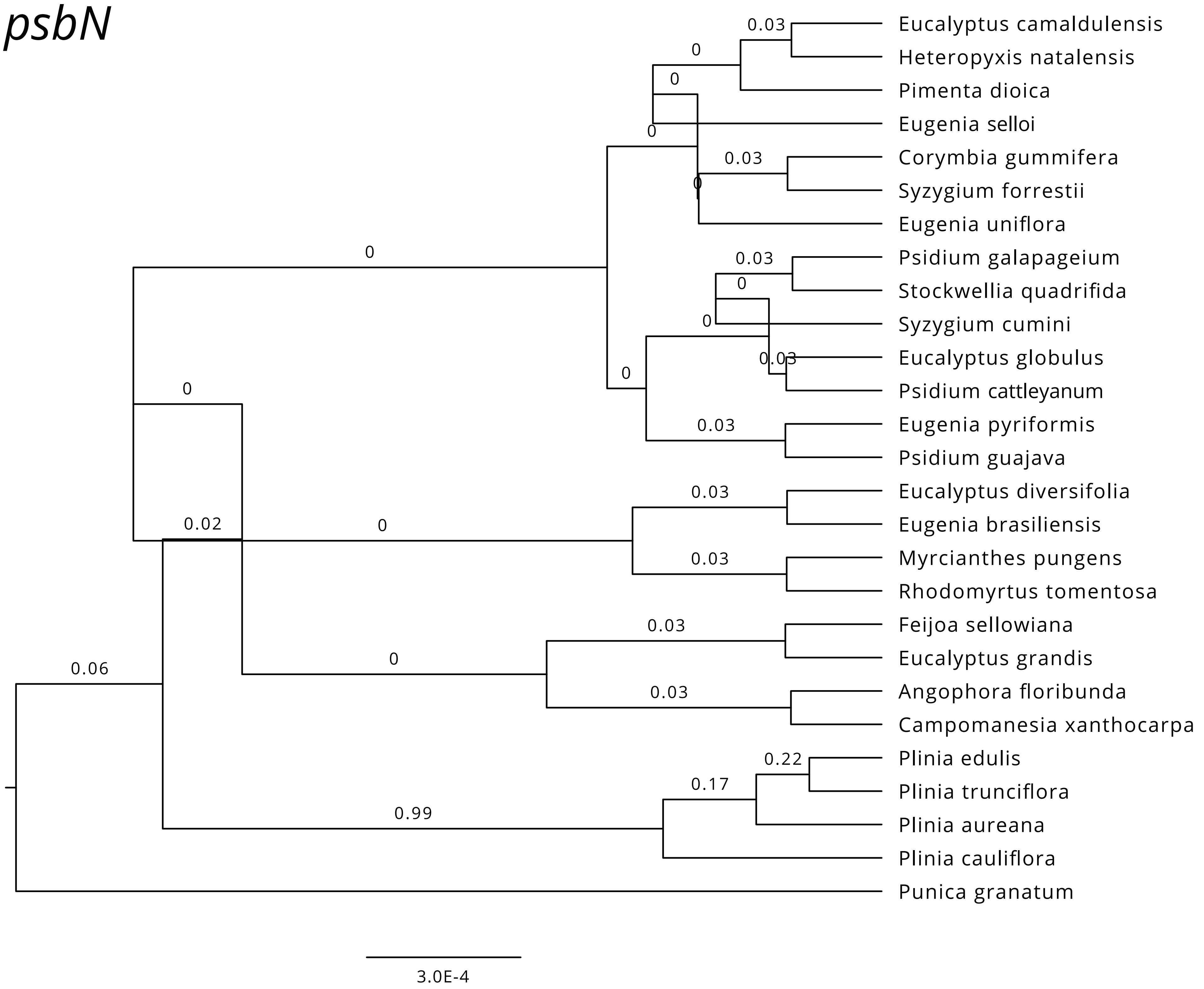
psbN
Eucalyptus camaldulensis
Heteropyxis natalensis
Pimenta dioica
Eugenia selloi
Corymbia gummifera
Syzygium forrestii
Eugenia uniflora
Psidium galapageium
Stockwellia quadrifida
Syzygium cumini
Eucalyptus globulus
Psidium cattleyanum
Eugenia pyriformis
Psidium guajava
Eucalyptus diversifolia
Eugenia brasiliensis
Myrcianthes pungens
Rhodomyrtus tomentosa
Feijoa sellowiana
Eucalyptus grandis
Angophora floribunda
Campomanesia xanthocarpa
Plinia edulis
Plinia trunciflora
Plinia aureana
Plinia cauliflora
Punica granatum
0.03
0
0
0
0.03
0
0
0.03
0
0
0.03
0
0.03
0
0.02
0
0.03
0.03
0
0.03
0.03
0.06
0.22
0.17
0.99
3.0E-4

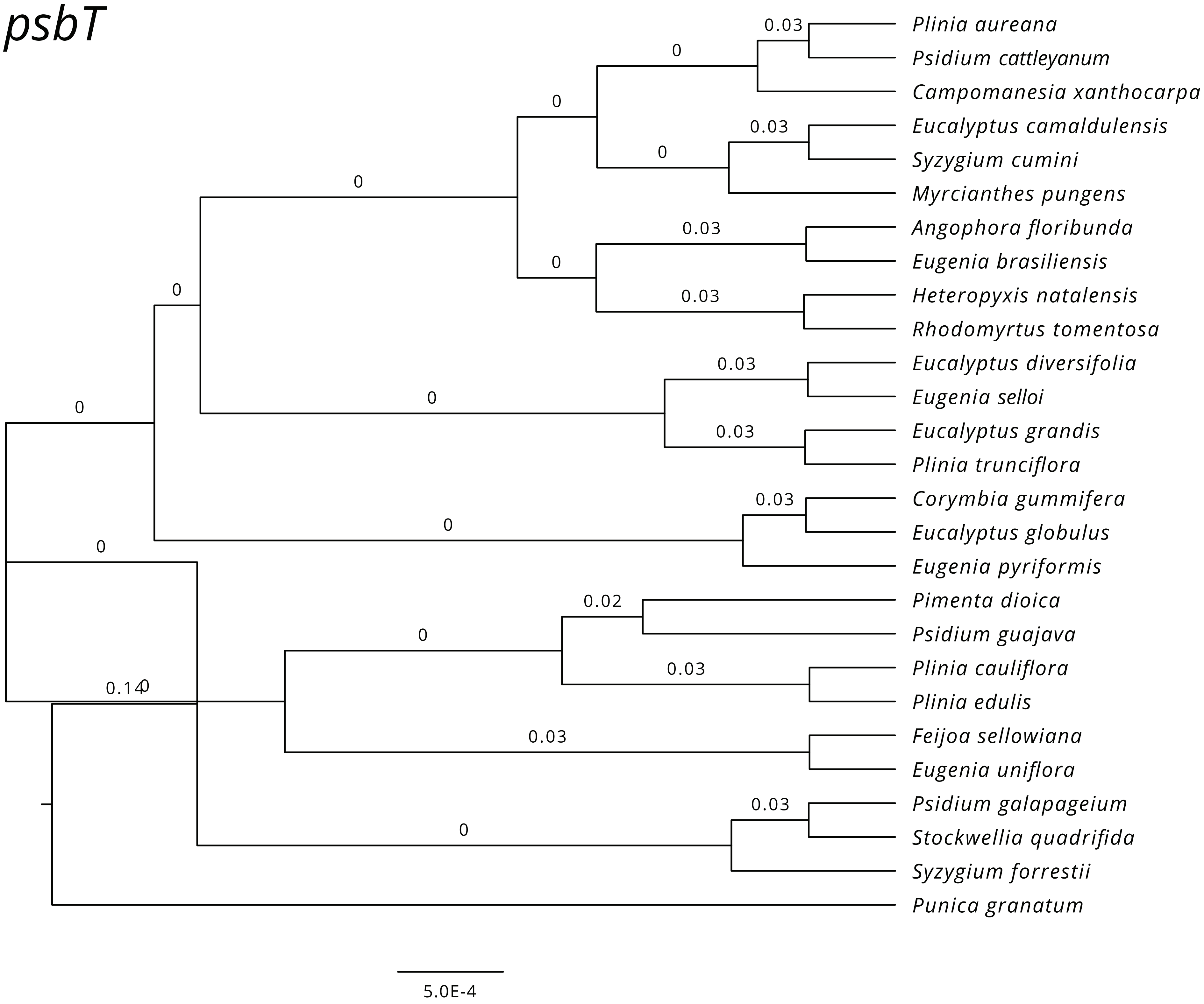
psbT
0.03
0
0
0.03
0
0
0.03
0
0.03
0
0.03
0
0.03
0
0.03
0
0
0.02
0
0.03
0.14
0
0.03
0.03
0
Plinia aureana
Psidium cattleyanum
Campomanesia xanthocarpa
Eucalyptus camaldulensis
Syzygium cumini
Myrcianthes pungens
Angophora floribunda
Eugenia brasiliensis
Heteropyxis natalensis
Rhodomyrtus tomentosa
Eucalyptus diversifolia
Eugenia selloi
Eucalyptus grandis
Plinia trunciflora
Corymbia gummifera
Eucalyptus globulus
Eugenia pyriformis
Pimenta dioica
Psidium guajava
Plinia cauliflora
Plinia edulis
Feijoa sellowiana
Eugenia uniflora
Psidium galapageium
Stockwellia quadrifida
Syzygium forrestii
Punica granatum
5.0E-4

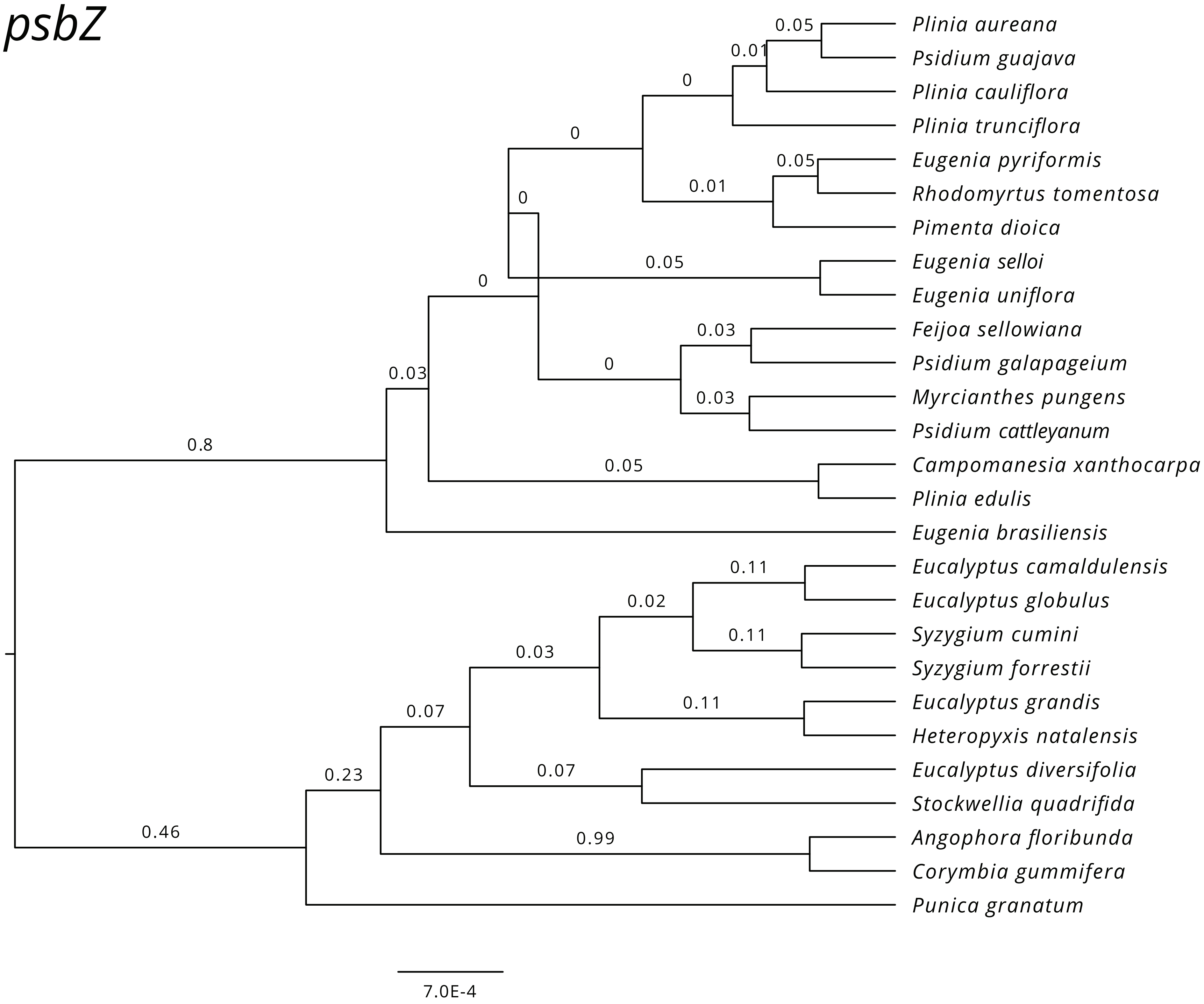
psbZ
Plinia aureana
Psidium guajava
Plinia cauliflora
Plinia trunciflora
Eugenia pyriformis
Rhodomyrtus tomentosa
Pimenta dioica
Eugenia selloi
Eugenia uniflora
Feijoa sellowiana
Psidium galapageium
Myrcianthes pungens
Psidium cattleyanum
Campomanesia xanthocarpa
Plinia edulis
Eugenia brasiliensis
Eucalyptus camaldulensis
Eucalyptus globulus
Syzygium cumini
Syzygium forrestii
Eucalyptus grandis
Heteropyxis natalensis
Eucalyptus diversifolia
Stockwellia quadrifida
Angophora floribunda
Corymbia gummifera
Punica granatum
0.05
0.01
0
0
0.05
0.01
0
0.05
0
0.03
0
0.03
0.03
0.8
0.05
0.11
0.02
0.11
0.03
0.11
0.07
0.07
0.23
0.46
0.99
7.0E-4

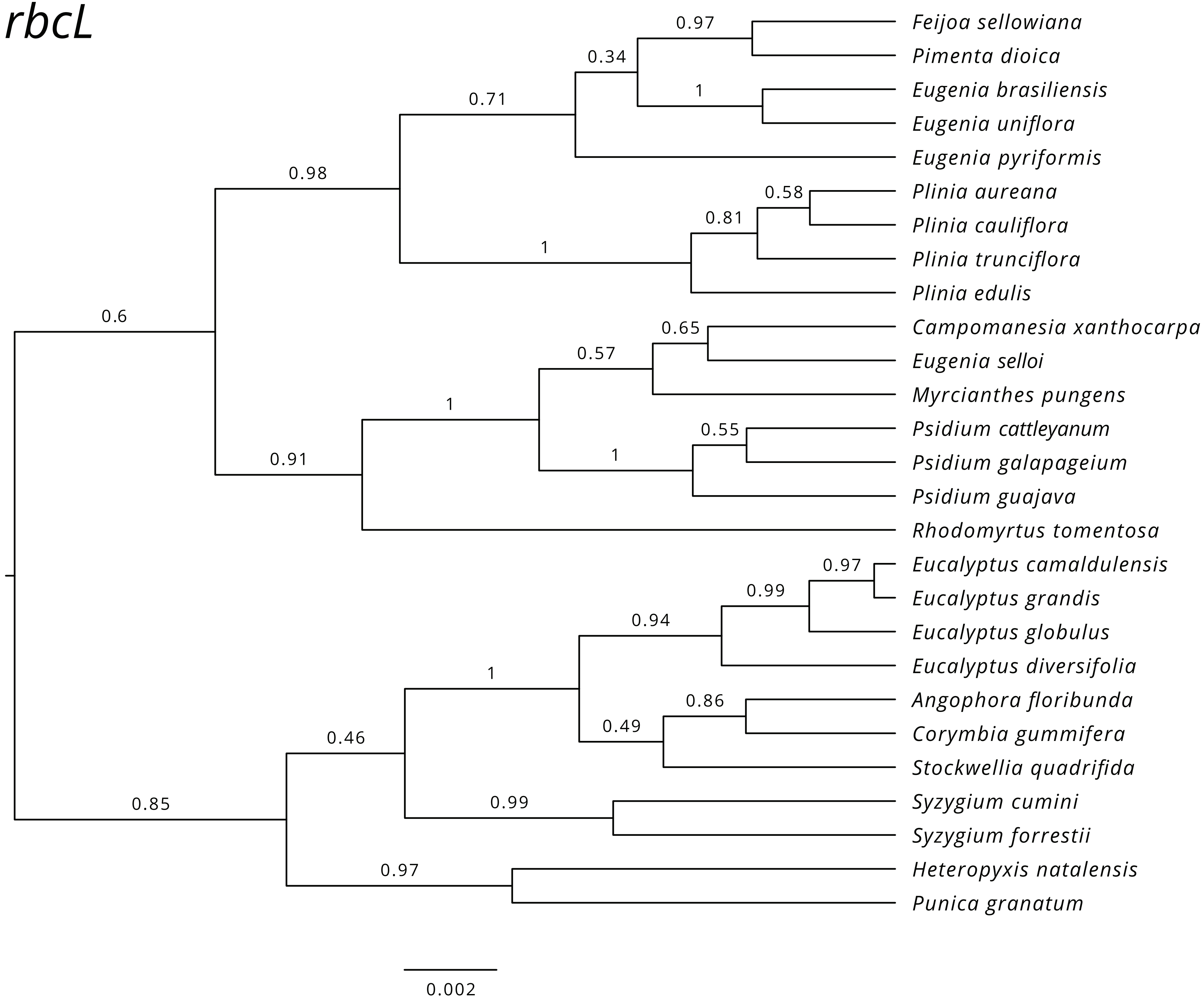
rbcL
0.97
0.34
1
0.71
0.98
0.58
0.81
1
0.6
0.65
0.57
1
0.55
1
0.91
0.97
0.99
0.94
1
0.86
0.49
0.46
0.99
0.85
0.97
Feijoa sellowiana
Pimenta dioica
Eugenia brasiliensis
Eugenia uniflora
Eugenia pyriformis
Plinia aureana
Plinia cauliflora
Plinia trunciflora
Plinia edulis
Campomanesia xanthocarpa
Eugenia selloi
Myrcianthes pungens
Psidium cattleyanum
Psidium galapageium
Psidium guajava
Rhodomyrtus tomentosa
Eucalyptus camaldulensis
Eucalyptus grandis
Eucalyptus globulus
Eucalyptus diversifolia
Angophora floribunda
Corymbia gummifera
Stockwellia quadrifida
Syzygium cumini
Syzygium forrestii
Heteropyxis natalensis
Punica granatum
0.002

# *rpl2*

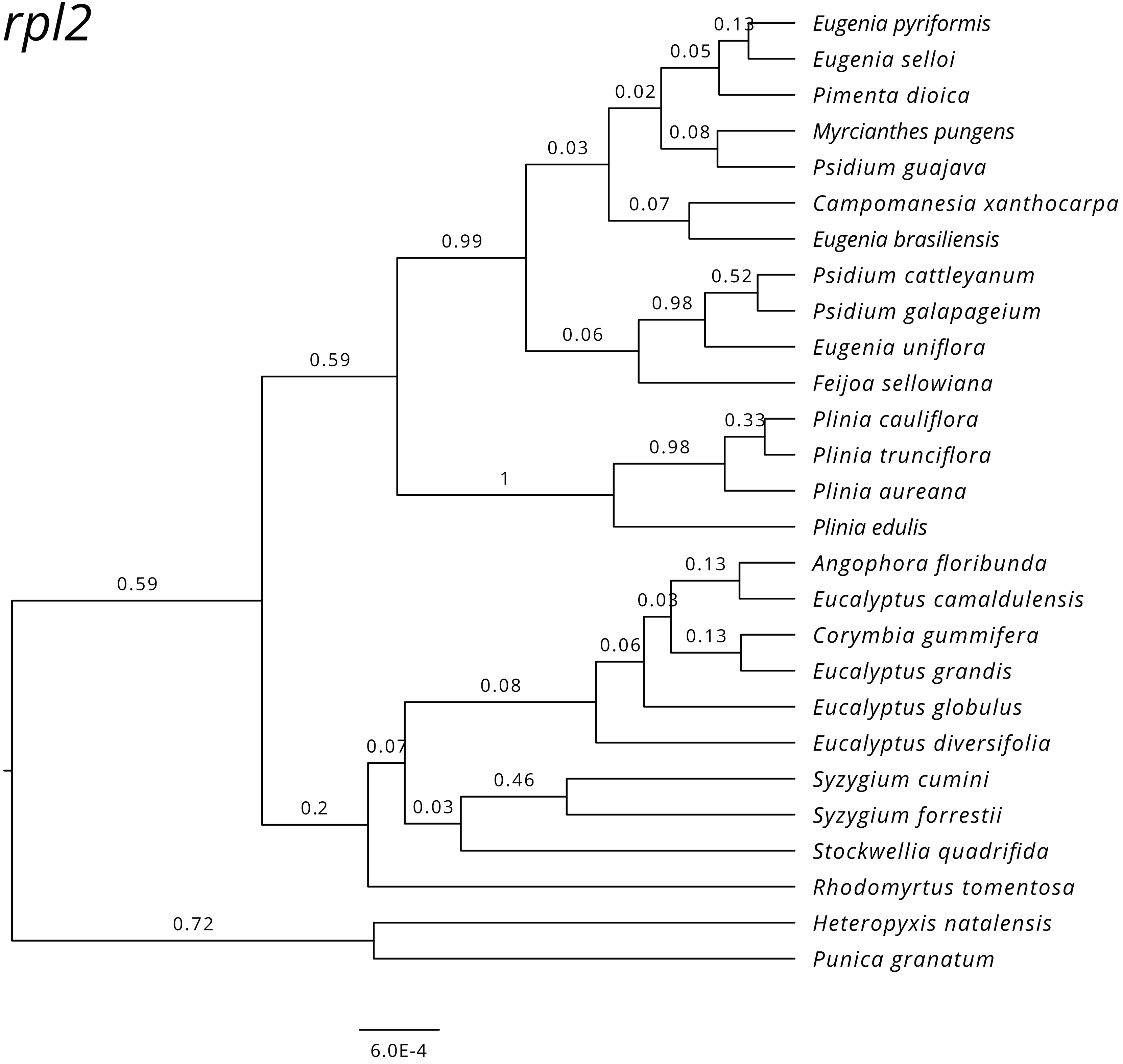

Eugenia pyriformis
Eugenia selloi
Pimenta dioica
Myrcianthes pungens
Psidium guajava
Campomanesia xanthocarpa
Eugenia brasiliensis
Psidium cattleyanum
Psidium galapageium
Eugenia uniflora
Feijoa sellowiana
Plinia cauliflora
Plinia trunciflora
Plinia aureana
Plinia edulis
Angophora floribunda
Eucalyptus camaldulensis
Corymbia gummifera
Eucalyptus grandis
Eucalyptus globulus
Eucalyptus diversifolia
Syzygium cumini
Syzygium forrestii
Stockwellia quadrifida
Rhodomyrtus tomentosa
Heteropyxis natalensis
Punica granatum
0.13
0.05
0.02
0.08
0.03
0.07
0.99
0.52
0.98
0.06
0.59
0.33
0.98
1
0.13
0.03
0.13
0.06
0.08
0.07
0.46
0.03
0.2
0.59
0.72
6.0E-4

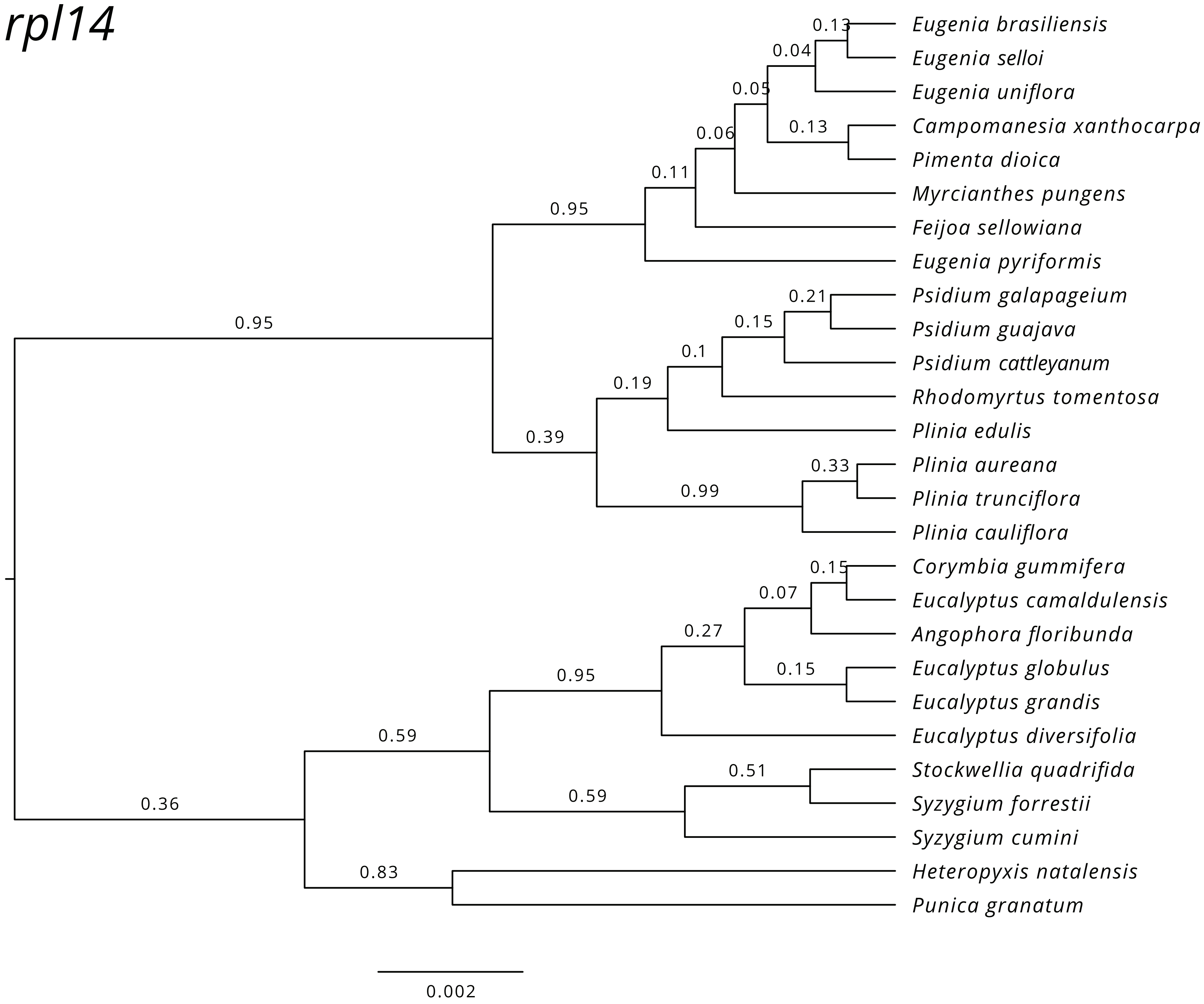

rpl14
0.13
0.04
0.05
0.13
0.06
0.11
0.95
0.95
0.21
0.15
0.1
0.19
0.39
0.33
0.99
0.15
0.07
0.27
0.15
0.95
0.59
0.51
0.59
0.36
0.83
Eugenia brasiliensis
Eugenia selloi
Eugenia uniflora
Campomanesia xanthocarpa
Pimenta dioica
Myrcianthes pungens
Feijoa sellowiana
Eugenia pyriformis
Psidium galapageium
Psidium guajava
Psidium cattleyanum
Rhodomyrtus tomentosa
Plinia edulis
Plinia aureana
Plinia trunciflora
Plinia cauliflora
Corymbia gummifera
Eucalyptus camaldulensis
Angophora floribunda
Eucalyptus globulus
Eucalyptus grandis
Eucalyptus diversifolia
Stockwellia quadrifida
Syzygium forrestii
Syzygium cumini
Heteropyxis natalensis
Punica granatum
0.002

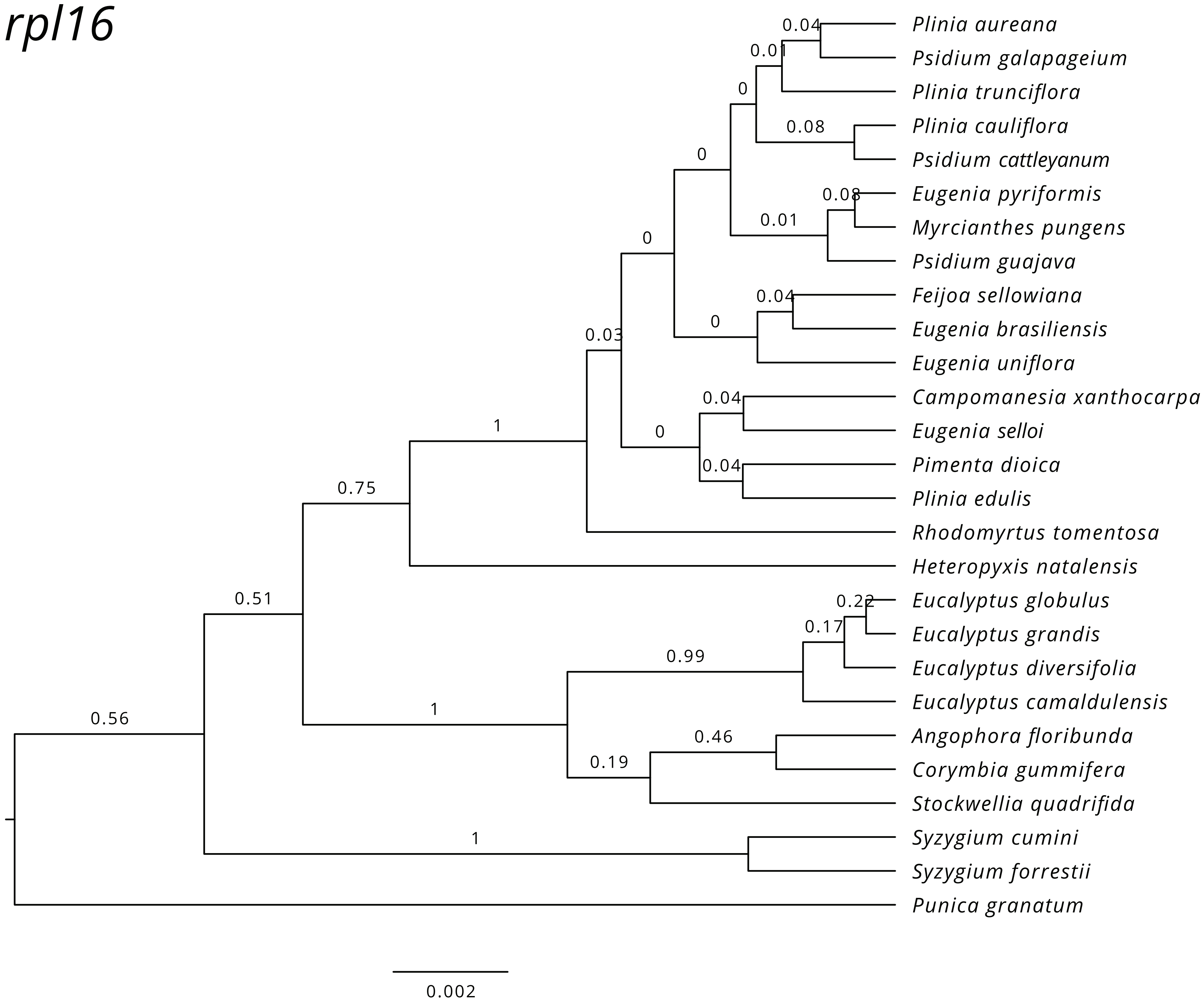

rpl16
Plinia aureana
Psidium galapageium
Plinia trunciflora
Plinia cauliflora
Psidium cattleyanum
Eugenia pyriformis
Myrcianthes pungens
Psidium guajava
Feijoa sellowiana
Eugenia brasiliensis
Eugenia uniflora
Campomanesia xanthocarpa
Eugenia selloi
Pimenta dioica
Plinia edulis
Rhodomyrtus tomentosa
Heteropyxis natalensis
Eucalyptus globulus
Eucalyptus grandis
Eucalyptus diversifolia
Eucalyptus camaldulensis
Angophora floribunda
Corymbia gummifera
Stockwellia quadrifida
Syzygium cumini
Syzygium forrestii
Punica granatum
0.04
0.01
0
0.08
0
0.08
0.01
0
0.04
0
0.03
0.04
0
0.04
1
0.75
0.51
0.22
0.17
0.99
1
0.46
0.19
0.56
1
0.002

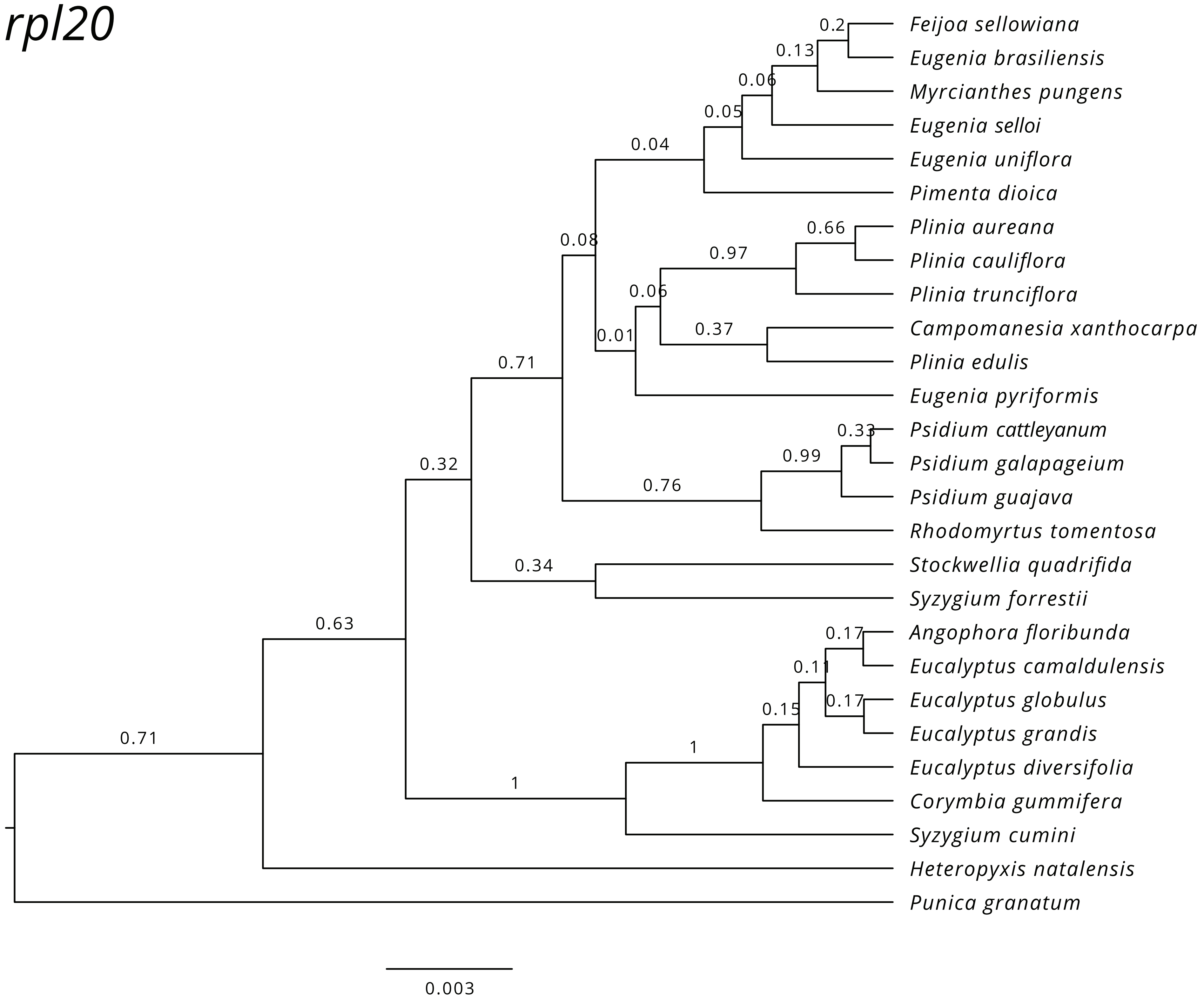
rpl20
Feijoa sellowiana
Eugenia brasiliensis
Myrcianthes pungens
Eugenia selloi
Eugenia uniflora
Pimenta dioica
Plinia aureana
Plinia cauliflora
Plinia trunciflora
Campomanesia xanthocarpa
Plinia edulis
Eugenia pyriformis
Psidium cattleyanum
Psidium galapageium
Psidium guajava
Rhodomyrtus tomentosa
Stockwellia quadrifida
Syzygium forrestii
Angophora floribunda
Eucalyptus camaldulensis
Eucalyptus globulus
Eucalyptus grandis
Eucalyptus diversifolia
Corymbia gummifera
Syzygium cumini
Heteropyxis natalensis
Punica granatum
0.2
0.13
0.06
0.05
0.04
0.66
0.97
0.08
0.06
0.37
0.01
0.71
0.33
0.99
0.32
0.76
0.34
0.63
0.17
0.11
0.17
0.15
0.71
1
1
0.003

# *rpl22*

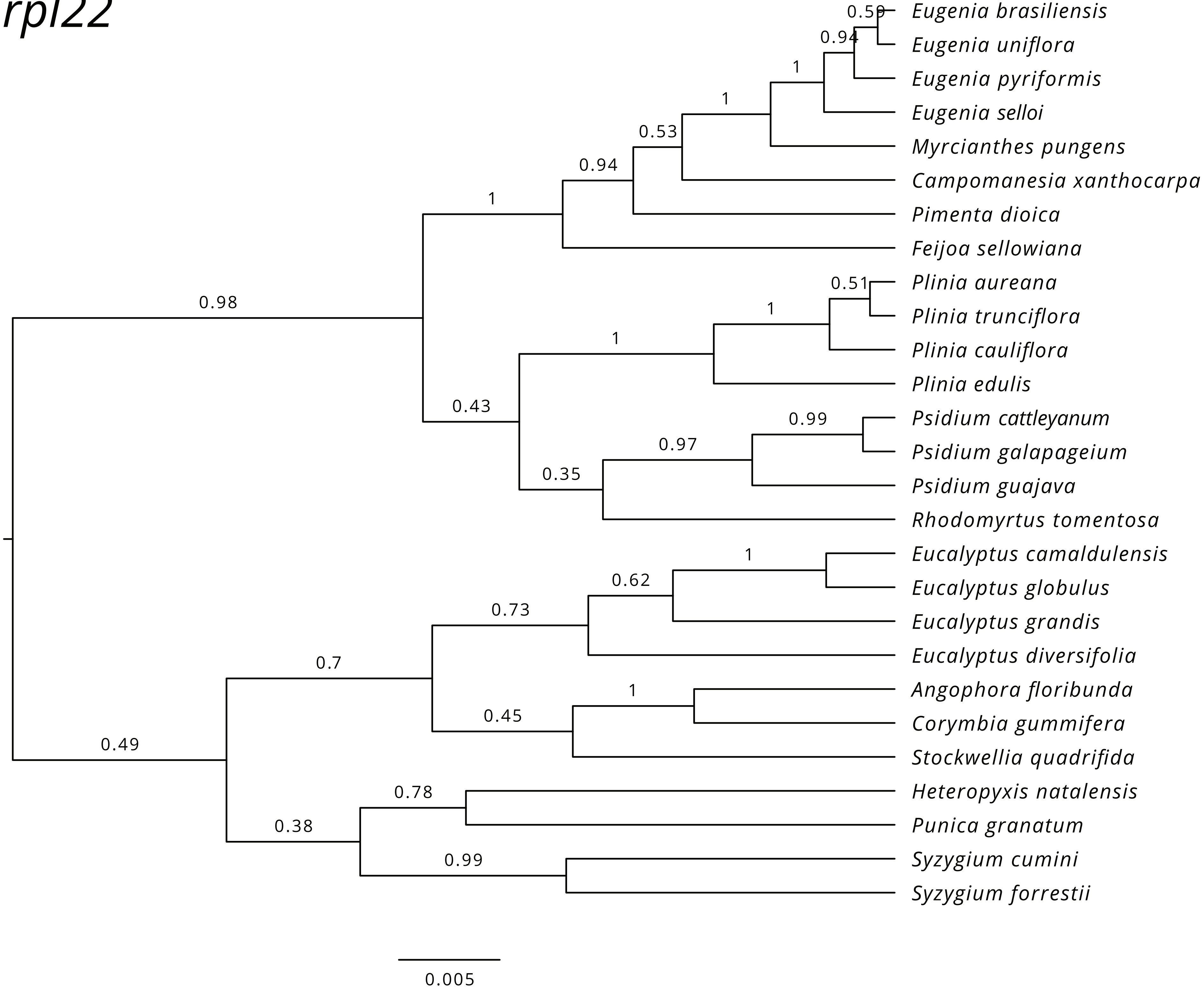
0.50
0.94
1
1
0.53
0.94
1
0.98
0.51
1
1
0.43
0.99
0.97
0.35
1
0.62
0.73
0.7
1
0.45
0.49
0.78
0.38
0.99
*Eugenia brasiliensis*
*Eugenia uniflora*
*Eugenia pyriformis*
*Eugenia selloi*
*Myrcianthes pungens*
*Campomanesia xanthocarpa*
*Pimenta dioica*
*Feijoa sellowiana*
*Plinia aureana*
*Plinia trunciflora*
*Plinia cauliflora*
*Plinia edulis*
*Psidium cattleyanum*
*Psidium galapageium*
*Psidium guajava*
*Rhodomyrtus tomentosa*
*Eucalyptus camaldulensis*
*Eucalyptus globulus*
*Eucalyptus grandis*
*Eucalyptus diversifolia*
*Angophora floribunda*
*Corymbia gummifera*
*Stockwellia quadrifida*
*Heteropyxis natalensis*
*Punica granatum*
*Syzygium cumini*
*Syzygium forrestii*
0.005

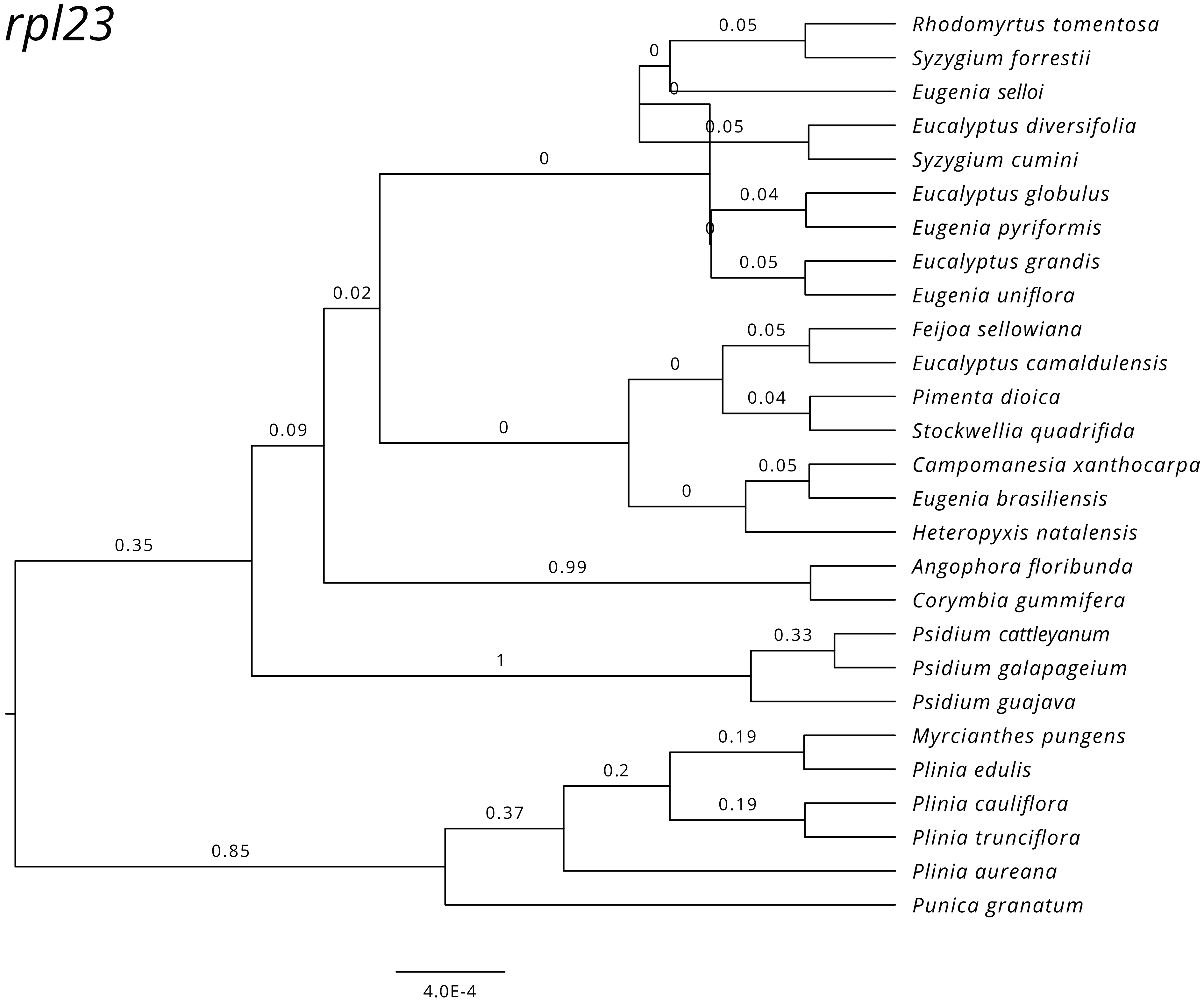
rpl23
Rhodomyrtus tomentosa
Syzygium forrestii
Eugenia selloi
Eucalyptus diversifolia
Syzygium cumini
Eucalyptus globulus
Eugenia pyriformis
Eucalyptus grandis
Eugenia uniflora
Feijoa sellowiana
Eucalyptus camaldulensis
Pimenta dioica
Stockwellia quadrifida
Campomanesia xanthocarpa
Eugenia brasiliensis
Heteropyxis natalensis
Angophora floribunda
Corymbia gummifera
Psidium cattleyanum
Psidium galapageium
Psidium guajava
Myrcianthes pungens
Plinia edulis
Plinia cauliflora
Plinia trunciflora
Plinia aureana
Punica granatum
0.05
0
0
0.05
0
0.04
0
0.05
0.02
0.05
0
0.04
0.09
0
0.05
0
0.35
0.99
0.33
1
0.19
0.2
0.37
0.19
0.85
4.0E-4

# *rpl32*

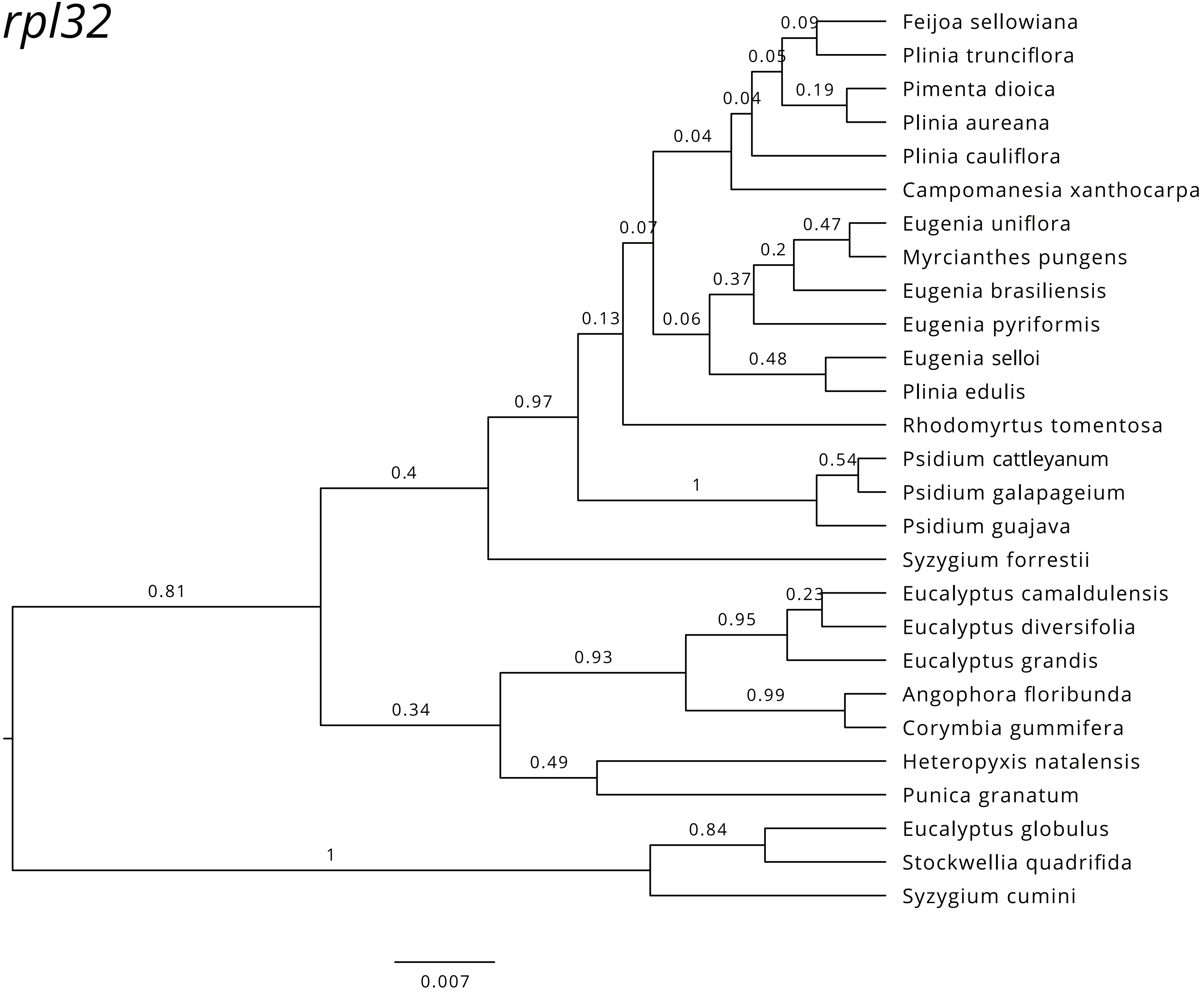

Feijoa sellowiana
Plinia trunciflora
Pimenta dioica
Plinia aureana
Plinia cauliflora
Campomanesia xanthocarpa
Eugenia uniflora
Myrcianthes pungens
Eugenia brasiliensis
Eugenia pyriformis
Eugenia selloi
Plinia edulis
Rhodomyrtus tomentosa
Psidium cattleyanum
Psidium galapageium
Psidium guajava
Syzygium forrestii
Eucalyptus camaldulensis
Eucalyptus diversifolia
Eucalyptus grandis
Angophora floribunda
Corymbia gummifera
Heteropyxis natalensis
Punica granatum
Eucalyptus globulus
Stockwellia quadrifida
Syzygium cumini
0.09
0.05
0.19
0.04
0.04
0.47
0.07
0.2
0.37
0.13
0.06
0.48
0.97
0.54
0.4
1
0.81
0.23
0.95
0.93
0.99
0.34
0.49
0.84
1
0.007

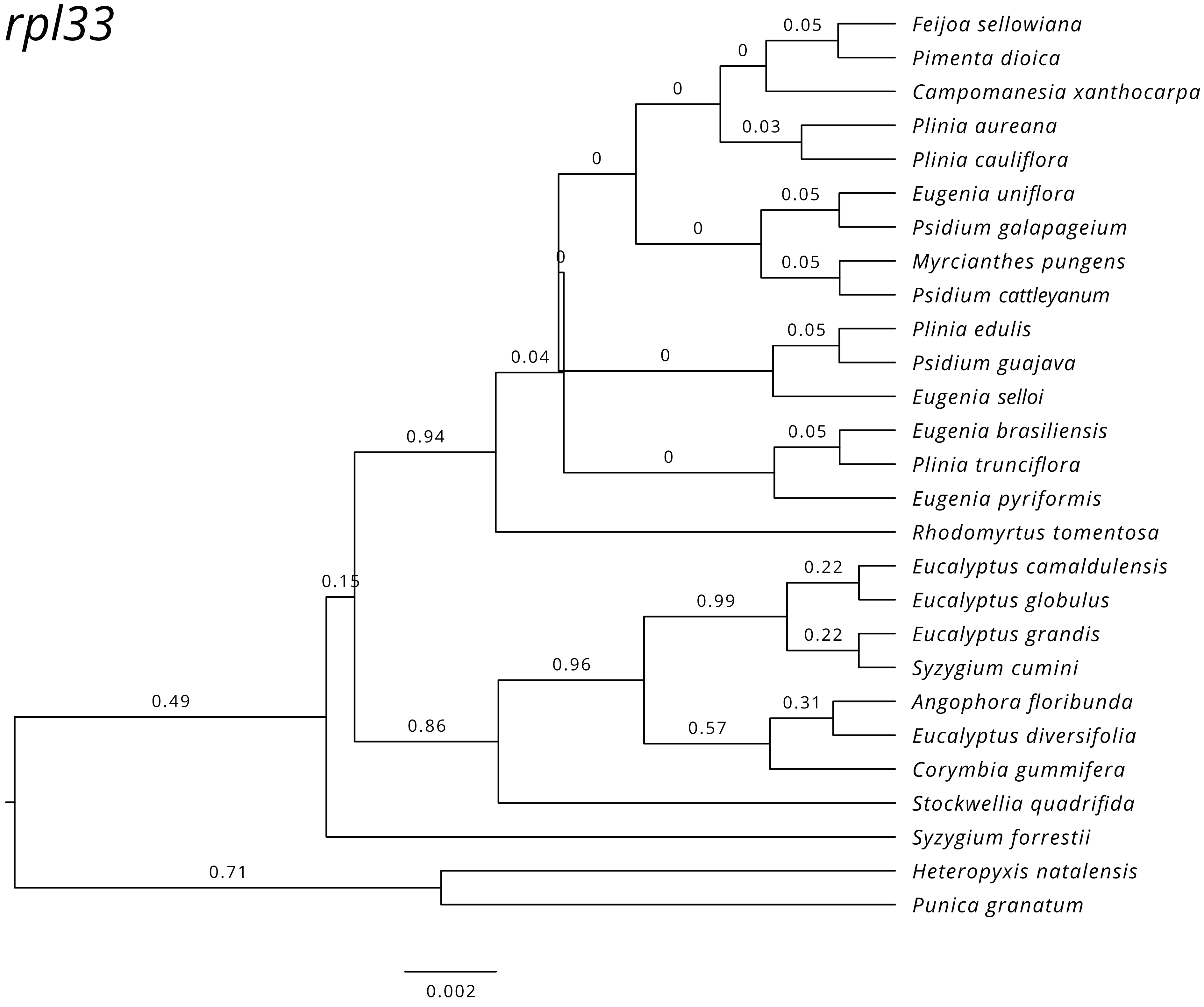
rpl33
0.05
0
0
0.03
0
0.05
0
0.05
0
0.05
0.04
0
0.94
0.05
0
0.15
0.22
0.99
0.22
0.96
0.31
0.49
0.86
0.57
0.71
Feijoa sellowiana
Pimenta dioica
Campomanesia xanthocarpa
Plinia aureana
Plinia cauliflora
Eugenia uniflora
Psidium galapageium
Myrcianthes pungens
Psidium cattleyanum
Plinia edulis
Psidium guajava
Eugenia selloi
Eugenia brasiliensis
Plinia trunciflora
Eugenia pyriformis
Rhodomyrtus tomentosa
Eucalyptus camaldulensis
Eucalyptus globulus
Eucalyptus grandis
Syzygium cumini
Angophora floribunda
Eucalyptus diversifolia
Corymbia gummifera
Stockwellia quadrifida
Syzygium forrestii
Heteropyxis natalensis
Punica granatum
0.002

# rpl36

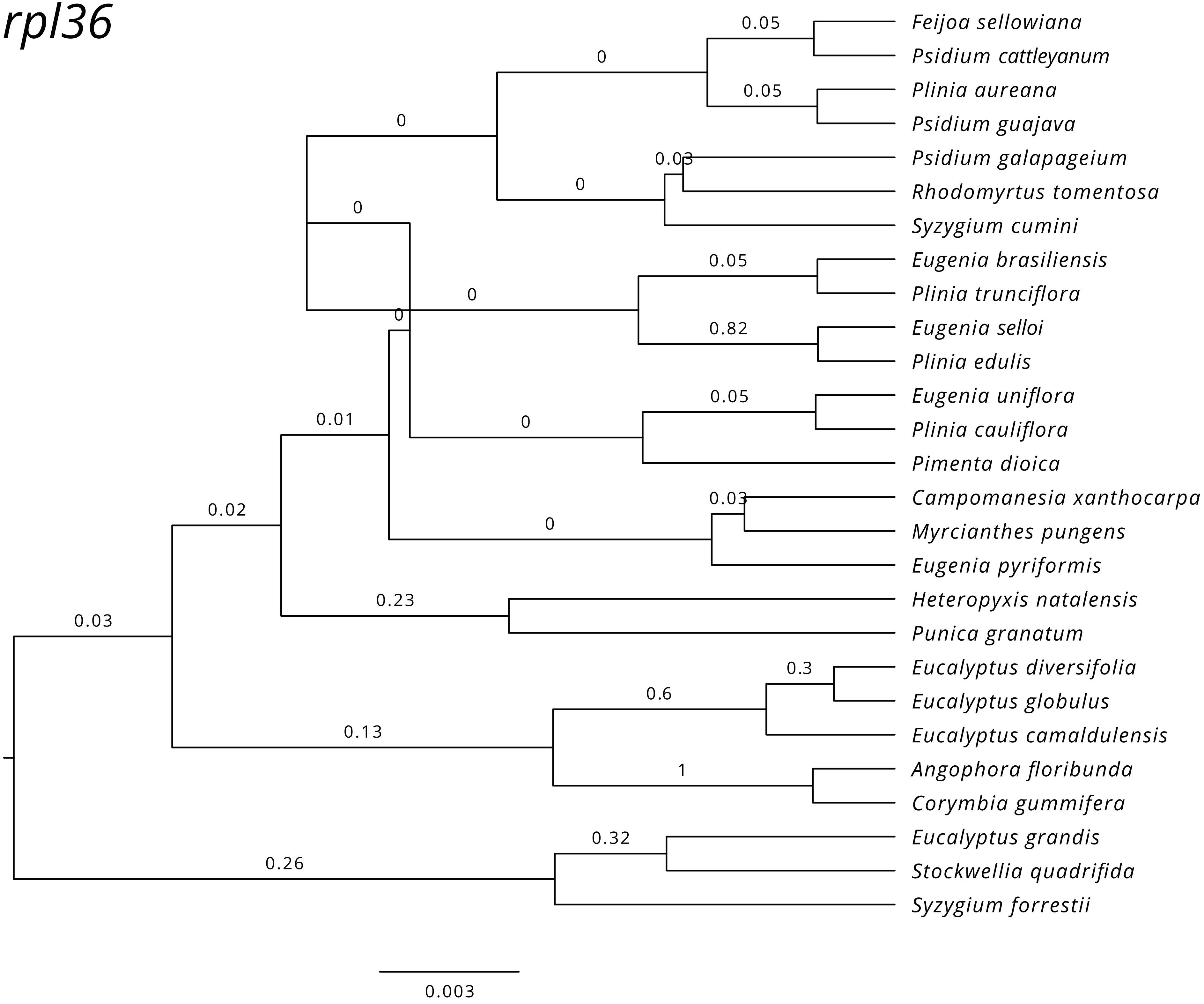
0.05
Feijoa sellowiana
Psidium cattleyanum
0
0.05
Plinia aureana
Psidium guajava
0
0.03
Psidium galapageium
0
Rhodomyrtus tomentosa
Syzygium cumini
0
0.05
Eugenia brasiliensis
Plinia trunciflora
0
0
0.82
Eugenia selloi
Plinia edulis
0.05
Eugenia uniflora
0.01
0
Plinia cauliflora
Pimenta dioica
0.03
Campomanesia xanthocarpa
0.02
0
Myrcianthes pungens
Eugenia pyriformis
0.23
Heteropyxis natalensis
0.03
Punica granatum
0.3
Eucalyptus diversifolia
0.6
Eucalyptus globulus
0.13
Eucalyptus camaldulensis
1
Angophora floribunda
Corymbia gummifera
0.32
Eucalyptus grandis
0.26
Stockwellia quadrifida
Syzygium forrestii
0.003

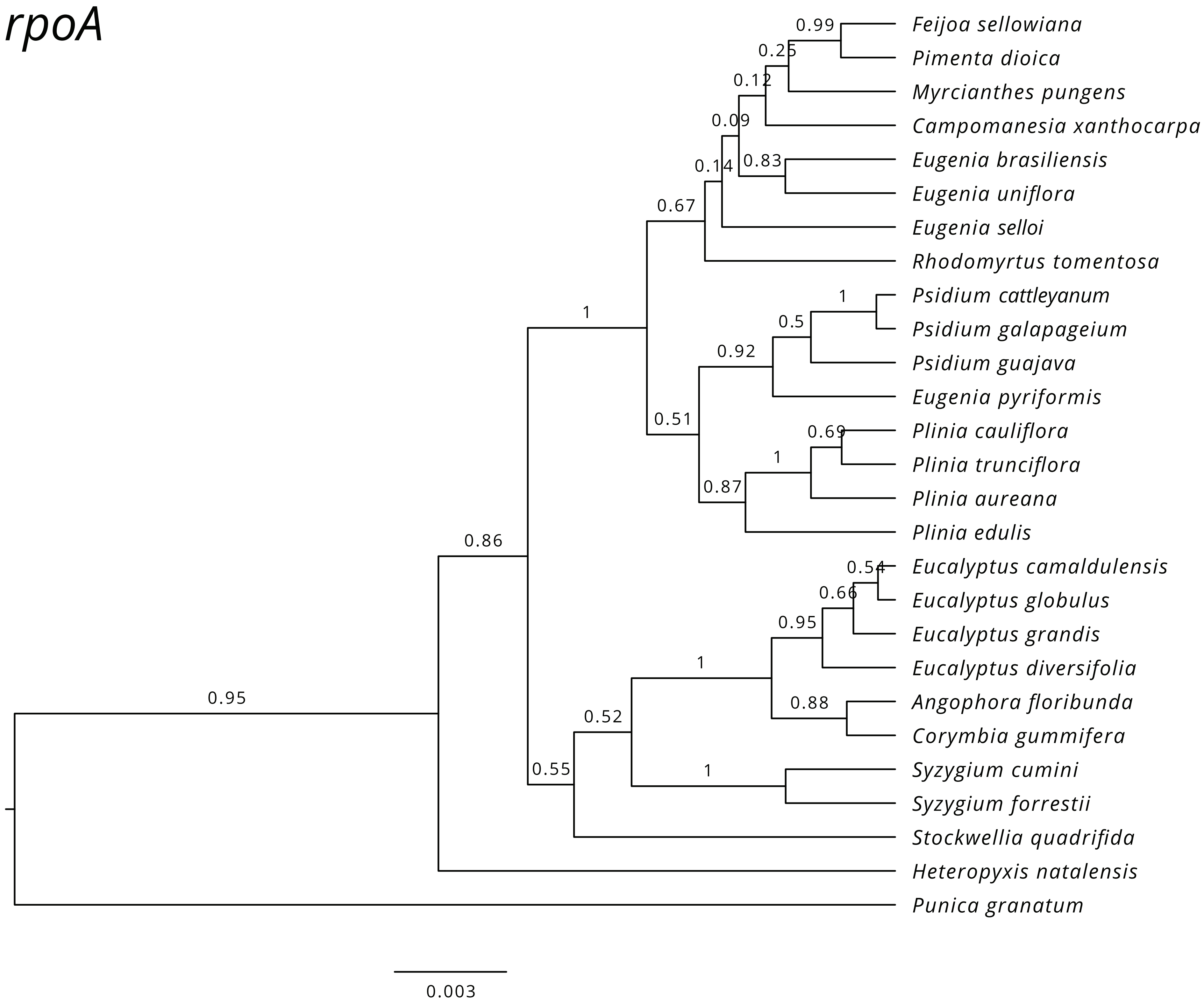
rpoA
0.99
0.25
0.12
0.09
0.83
0.14
0.67
1
1
0.5
0.92
0.51
0.69
1
0.87
0.86
0.54
0.66
0.95
1
0.88
0.52
0.55
1
0.95
Feijoa sellowiana
Pimenta dioica
Myrcianthes pungens
Campomanesia xanthocarpa
Eugenia brasiliensis
Eugenia uniflora
Eugenia selloi
Rhodomyrtus tomentosa
Psidium cattleyanum
Psidium galapageium
Psidium guajava
Eugenia pyriformis
Plinia cauliflora
Plinia trunciflora
Plinia aureana
Plinia edulis
Eucalyptus camaldulensis
Eucalyptus globulus
Eucalyptus grandis
Eucalyptus diversifolia
Angophora floribunda
Corymbia gummifera
Syzygium cumini
Syzygium forrestii
Stockwellia quadrifida
Heteropyxis natalensis
Punica granatum
0.003

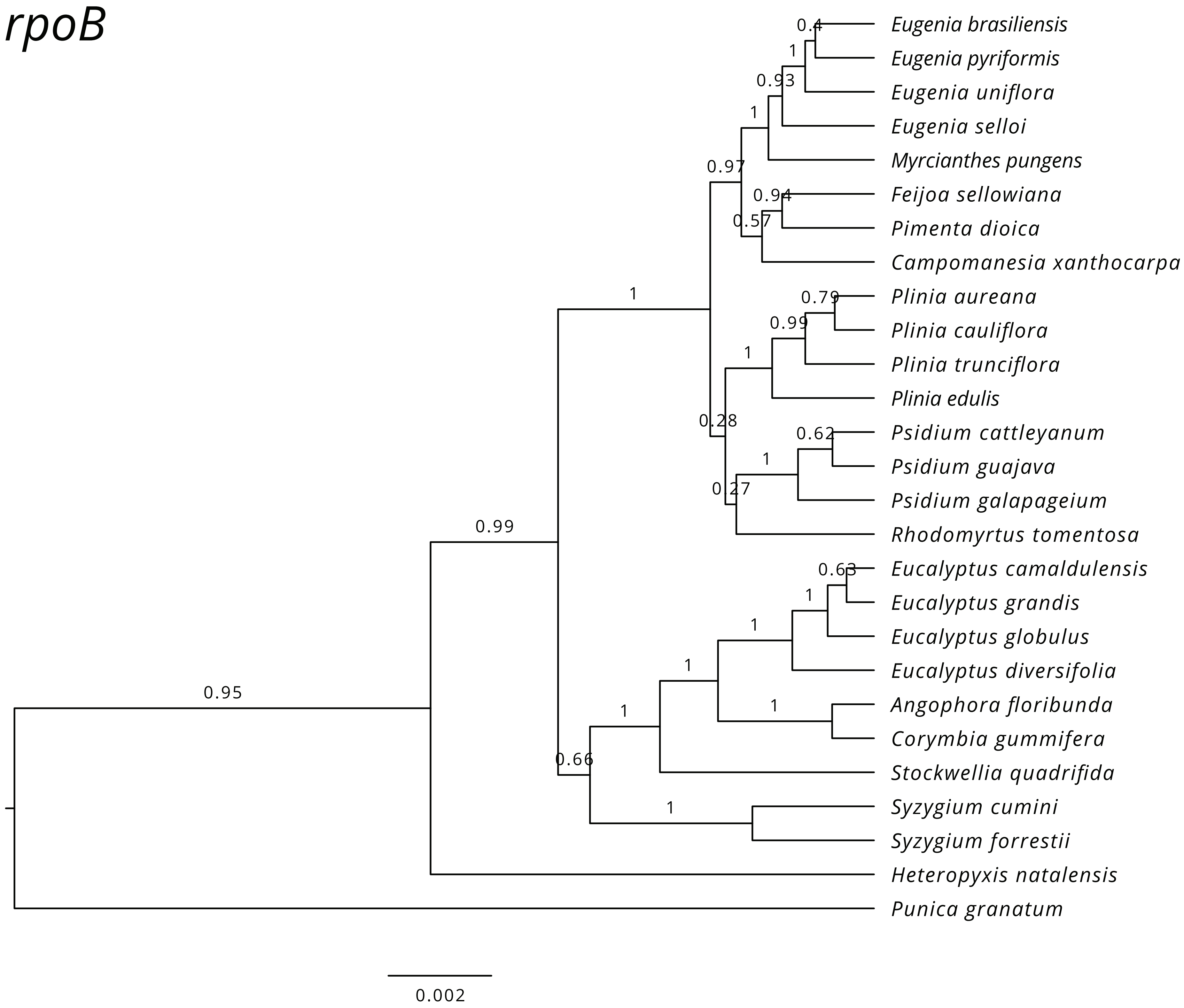
rpoB
Eugenia brasiliensis
Eugenia pyriformis
Eugenia uniflora
Eugenia selloi
Myrcianthes pungens
Feijoa sellowiana
Pimenta dioica
Campomanesia xanthocarpa
Plinia aureana
Plinia cauliflora
Plinia trunciflora
Plinia edulis
Psidium cattleyanum
Psidium guajava
Psidium galapageium
Rhodomyrtus tomentosa
Eucalyptus camaldulensis
Eucalyptus grandis
Eucalyptus globulus
Eucalyptus diversifolia
Angophora floribunda
Corymbia gummifera
Stockwellia quadrifida
Syzygium cumini
Syzygium forrestii
Heteropyxis natalensis
Punica granatum
0.4
1
0.93
1
0.97
0.94
0.57
1
0.79
0.99
1
0.28
0.62
1
0.27
0.99
0.63
1
1
1
1
0.66
1
0.95
0.002

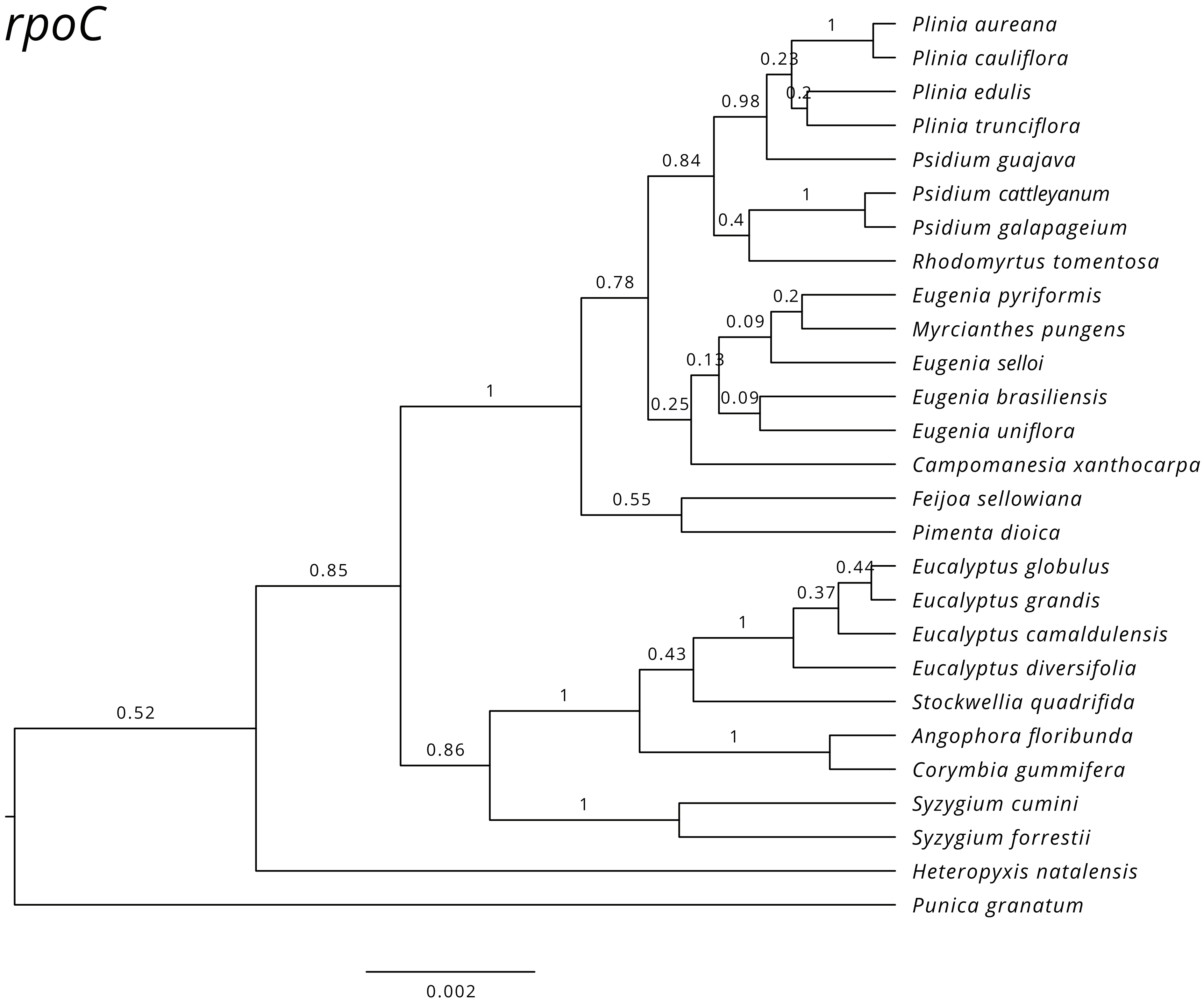
rpoC
Plinia aureana
Plinia cauliflora
Plinia edulis
Plinia trunciflora
Psidium guajava
Psidium cattleyanum
Psidium galapageium
Rhodomyrtus tomentosa
Eugenia pyriformis
Myrcianthes pungens
Eugenia selloi
Eugenia brasiliensis
Eugenia uniflora
Campomanesia xanthocarpa
Feijoa sellowiana
Pimenta dioica
Eucalyptus globulus
Eucalyptus grandis
Eucalyptus camaldulensis
Eucalyptus diversifolia
Stockwellia quadrifida
Angophora floribunda
Corymbia gummifera
Syzygium cumini
Syzygium forrestii
Heteropyxis natalensis
Punica granatum
1
0.23
0.2
0.98
0.84
1
0.4
0.78
0.2
0.09
0.13
0.09
0.25
1
0.55
0.44
0.37
1
0.43
1
1
0.85
0.86
1
0.52
0.002

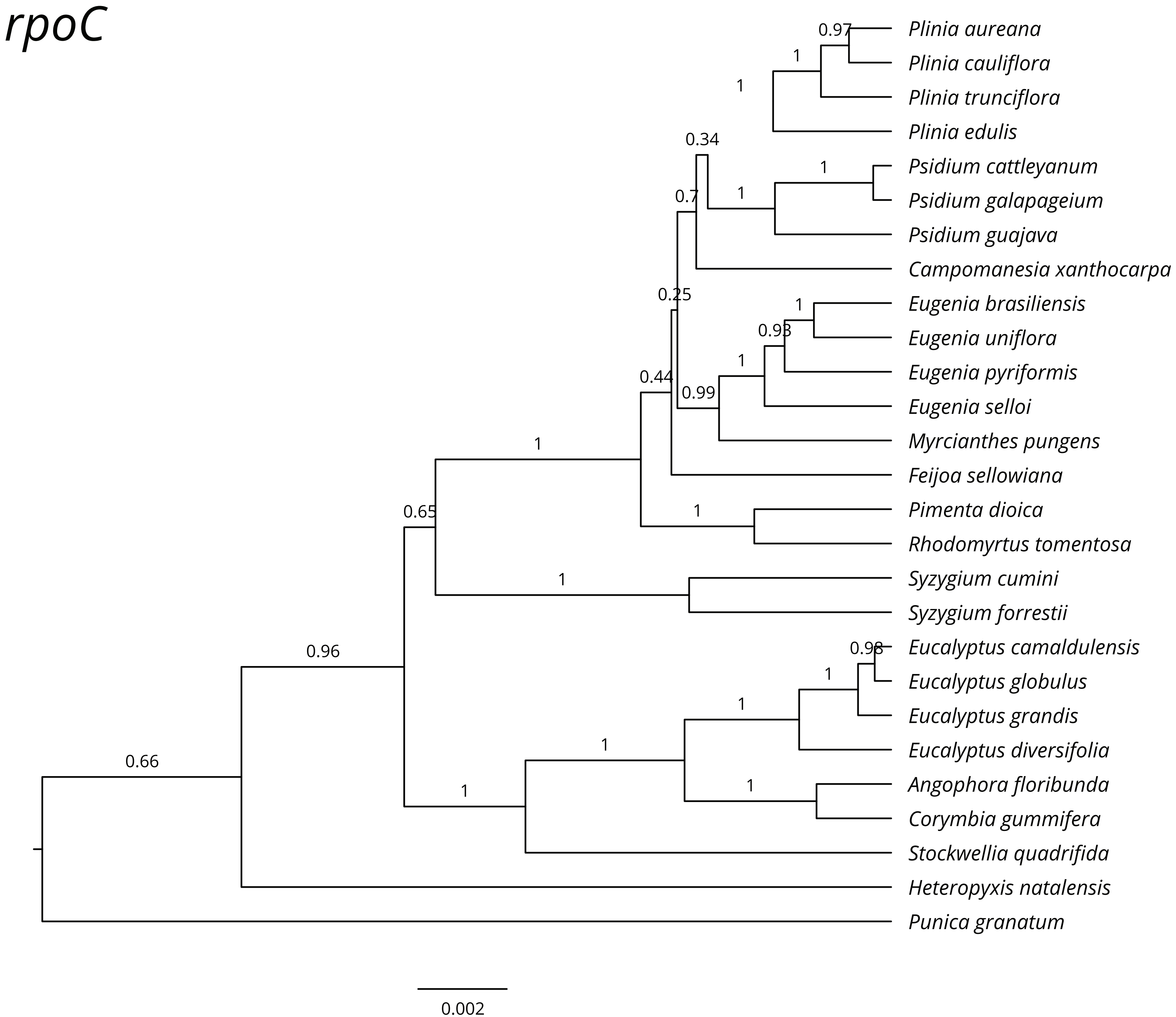
rpoC
0.97
1
1
Plinia aureana
Plinia cauliflora
Plinia trunciflora
Plinia edulis
0.34
1
1
0.7
Psidium cattleyanum
Psidium galapageium
Psidium guajava
Campomanesia xanthocarpa
0.25
1
0.93
1
0.44
0.99
Eugenia brasiliensis
Eugenia uniflora
Eugenia pyriformis
Eugenia selloi
Myrcianthes pungens
1
Feijoa sellowiana
1
Pimenta dioica
Rhodomyrtus tomentosa
0.65
1
Syzygium cumini
Syzygium forrestii
0.96
0.98
1
1
Eucalyptus camaldulensis
Eucalyptus globulus
Eucalyptus grandis
Eucalyptus diversifolia
0.66
1
1
1
Angophora floribunda
Corymbia gummifera
Stockwellia quadrifida
Heteropyxis natalensis
Punica granatum
0.002

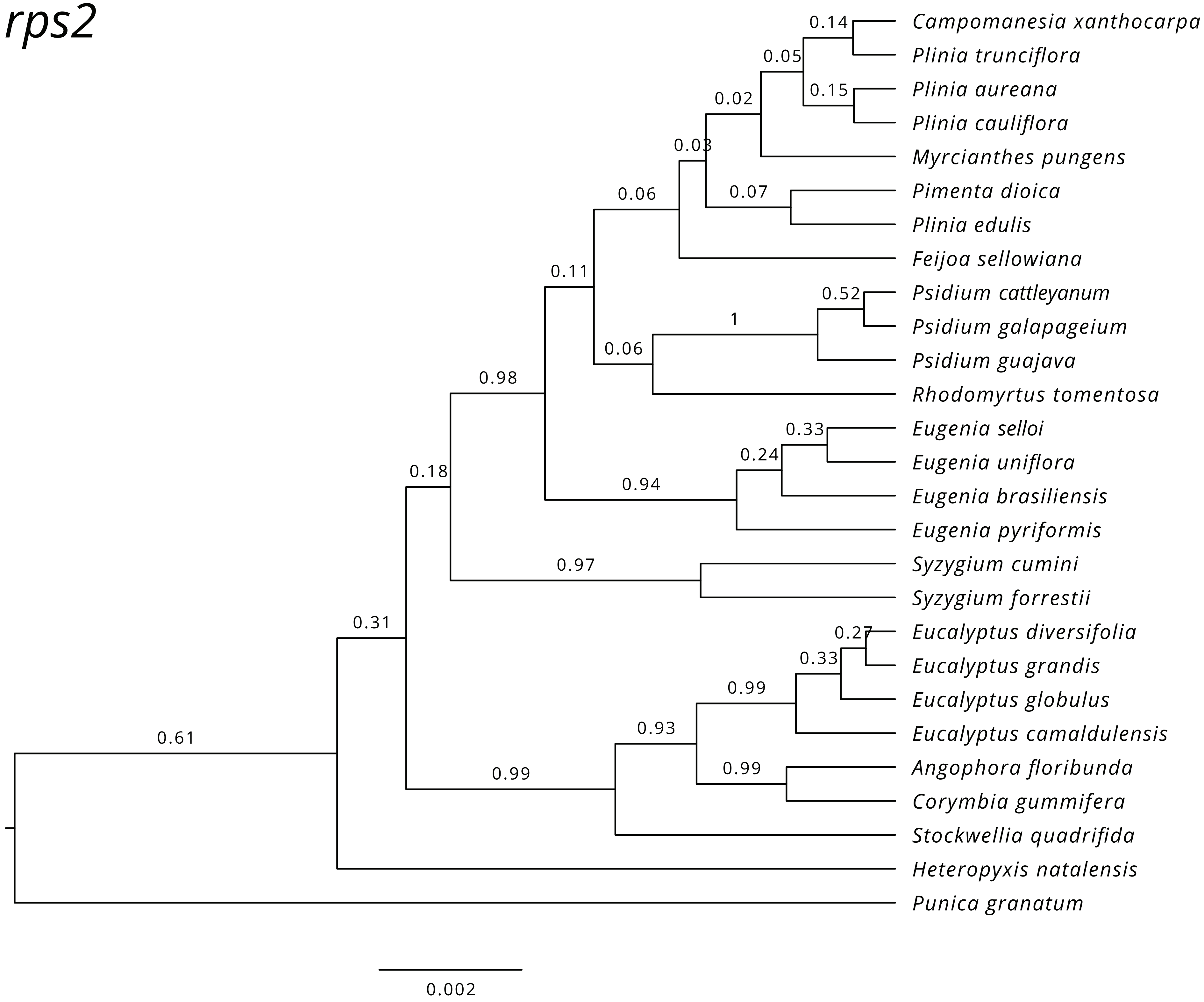

rps2
0.14
0.05
0.15
0.02
0.03
0.07
0.06
0.11
0.52
1
0.06
0.98
0.33
0.24
0.18
0.94
0.97
0.31
0.27
0.33
0.99
0.93
0.61
0.99
0.99
Campomanesia xanthocarpa
Plinia trunciflora
Plinia aureana
Plinia cauliflora
Myrcianthes pungens
Pimenta dioica
Plinia edulis
Feijoa sellowiana
Psidium cattleyanum
Psidium galapageium
Psidium guajava
Rhodomyrtus tomentosa
Eugenia selloi
Eugenia uniflora
Eugenia brasiliensis
Eugenia pyriformis
Syzygium cumini
Syzygium forrestii
Eucalyptus diversifolia
Eucalyptus grandis
Eucalyptus globulus
Eucalyptus camaldulensis
Angophora floribunda
Corymbia gummifera
Stockwellia quadrifida
Heteropyxis natalensis
Punica granatum
0.002

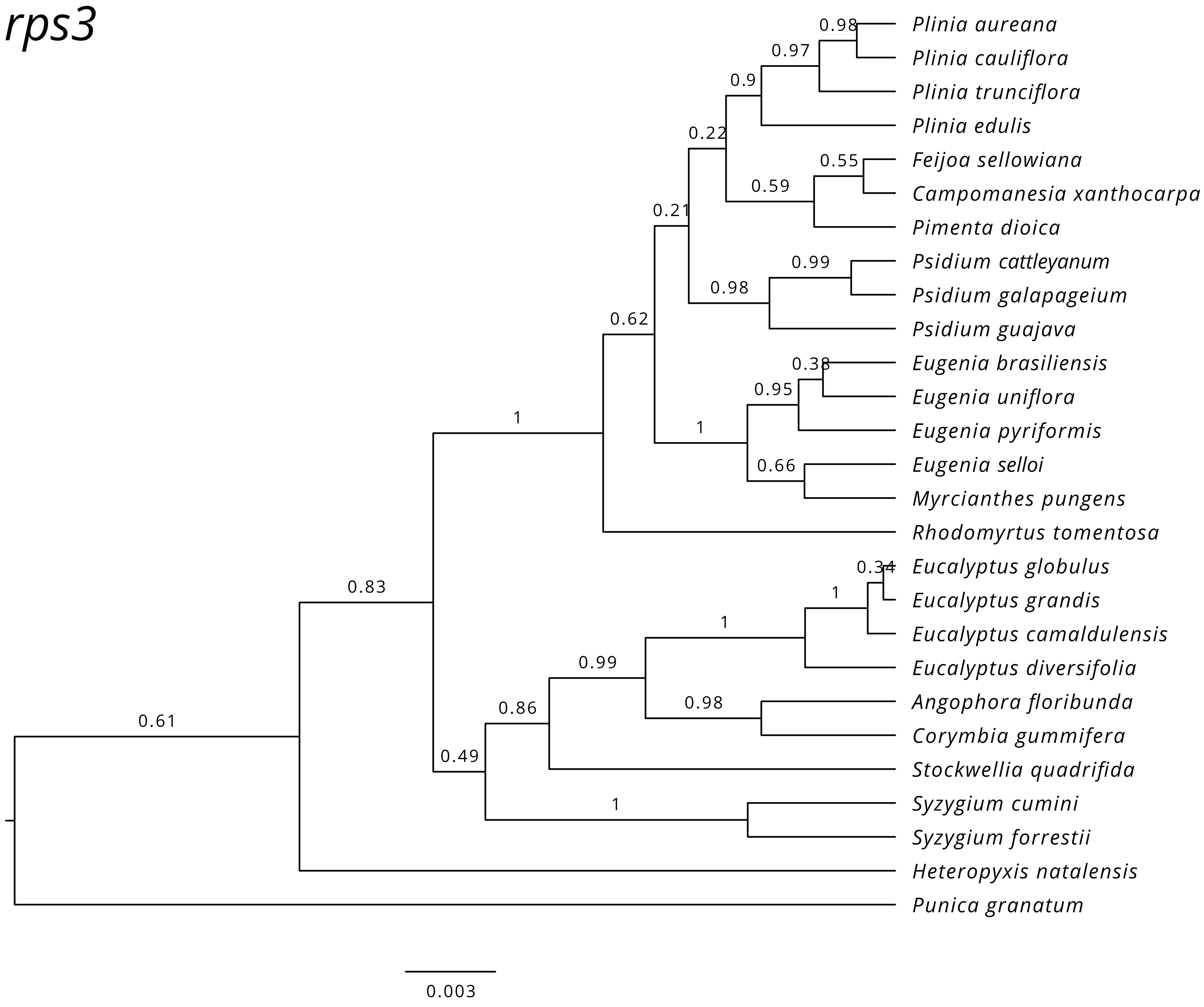
rps3
0.98
0.97
0.9
0.22
0.55
0.59
0.21
0.99
0.98
0.62
0.38
0.95
1
1
0.66
0.34
1
0.83
1
0.99
0.98
0.86
0.61
0.49
1
0.003
Plinia aureana
Plinia cauliflora
Plinia trunciflora
Plinia edulis
Feijoa sellowiana
Campomanesia xanthocarpa
Pimenta dioica
Psidium cattleyanum
Psidium galapageium
Psidium guajava
Eugenia brasiliensis
Eugenia uniflora
Eugenia pyriformis
Eugenia selloi
Myrcianthes pungens
Rhodomyrtus tomentosa
Eucalyptus globulus
Eucalyptus grandis
Eucalyptus camaldulensis
Eucalyptus diversifolia
Angophora floribunda
Corymbia gummifera
Stockwellia quadrifida
Syzygium cumini
Syzygium forrestii
Heteropyxis natalensis
Punica granatum

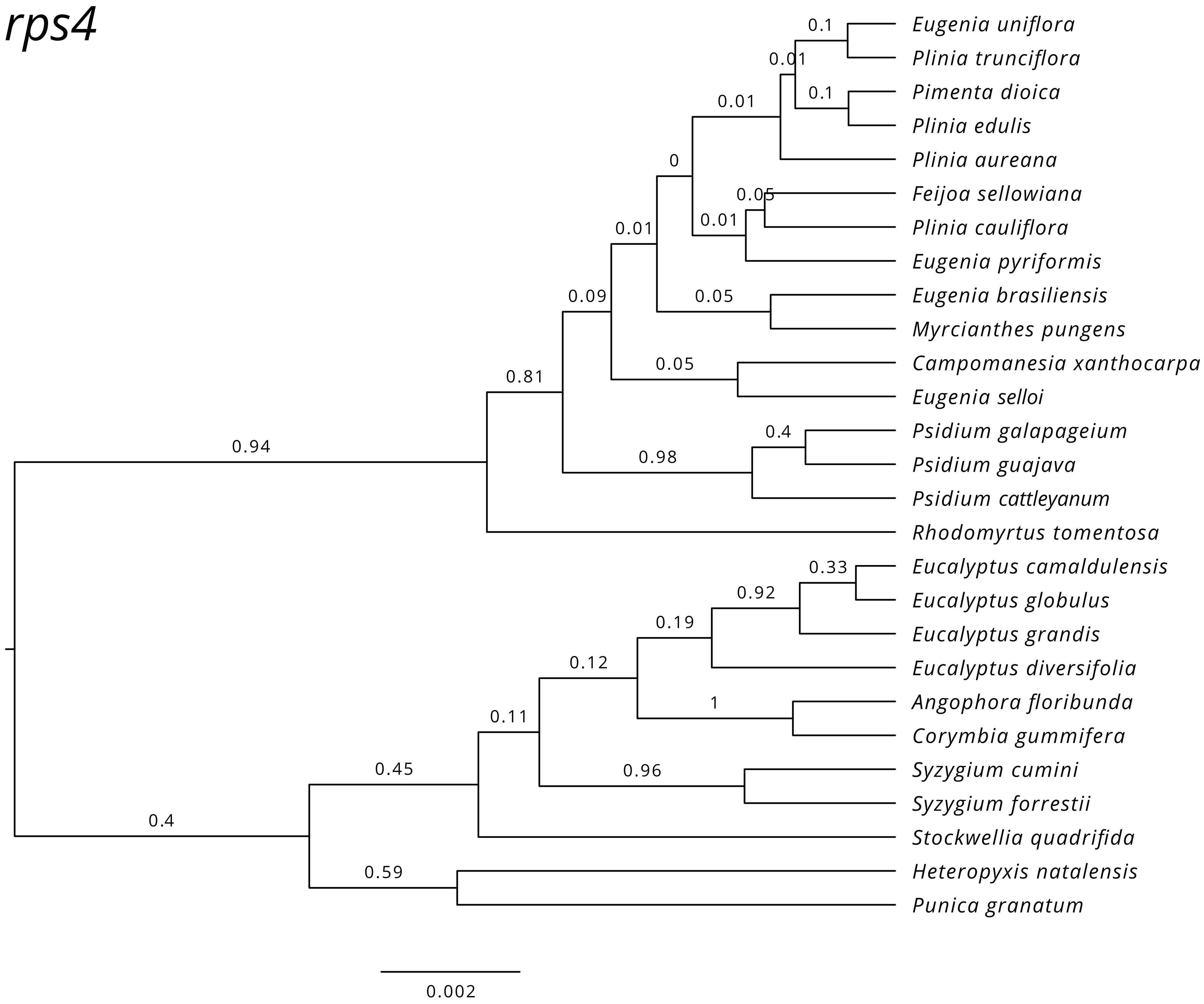
rps4
Eugenia uniflora
Plinia trunciflora
Pimenta dioica
Plinia edulis
Plinia aureana
Feijoa sellowiana
Plinia cauliflora
Eugenia pyriformis
Eugenia brasiliensis
Myrcianthes pungens
Campomanesia xanthocarpa
Eugenia selloi
Psidium galapageium
Psidium guajava
Psidium cattleyanum
Rhodomyrtus tomentosa
Eucalyptus camaldulensis
Eucalyptus globulus
Eucalyptus grandis
Eucalyptus diversifolia
Angophora floribunda
Corymbia gummifera
Syzygium cumini
Syzygium forrestii
Stockwellia quadrifida
Heteropyxis natalensis
Punica granatum
0.1
0.01
0.1
0.01
0
0.05
0.01
0.01
0.09
0.05
0.05
0.81
0.4
0.98
0.94
0.33
0.92
0.19
0.12
1
0.11
0.96
0.45
0.4
0.59
0.002

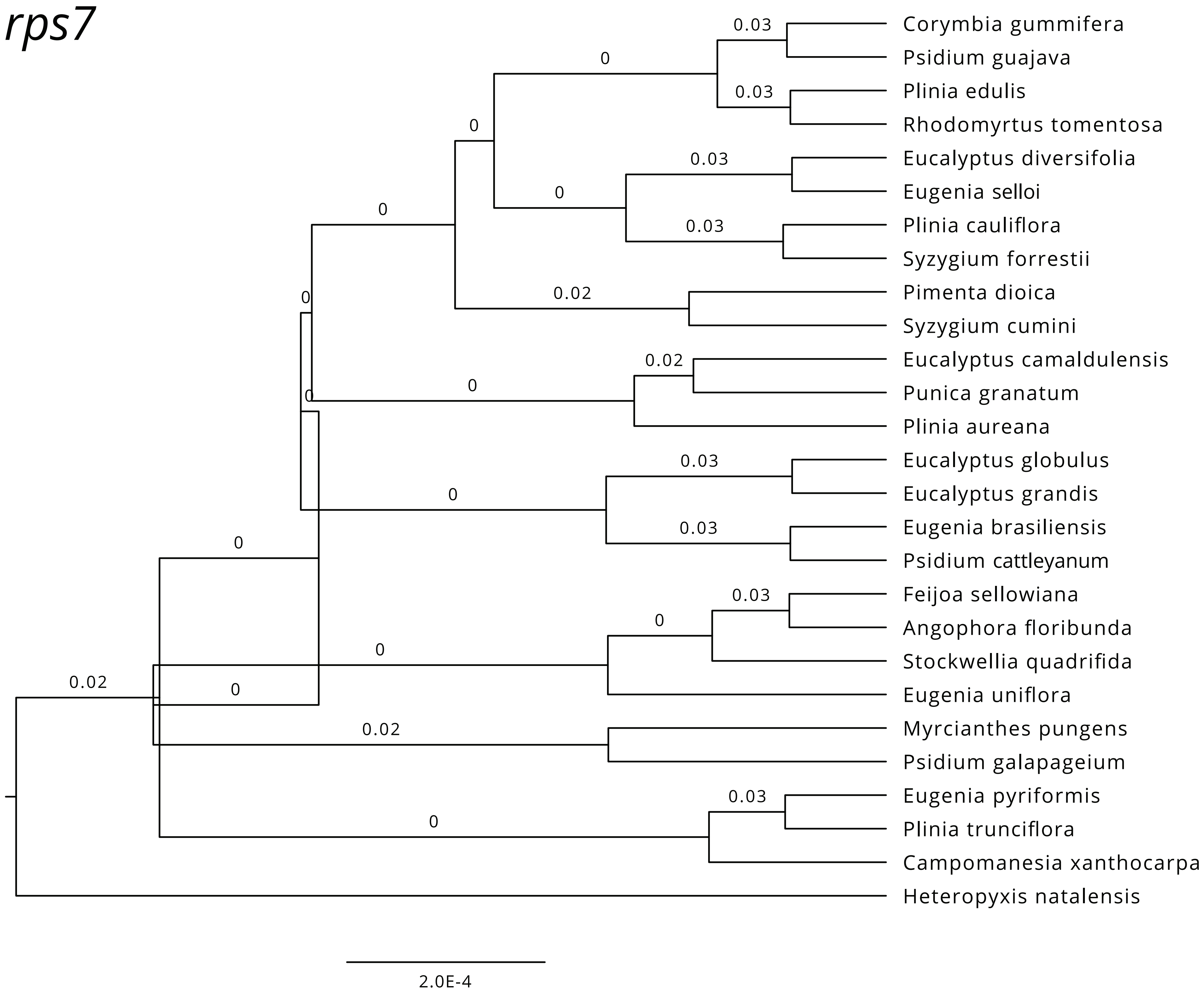
rps7
0.03
Corymbia gummifera
Psidium guajava
0
0.03
Plinia edulis
Rhodomyrtus tomentosa
0
0.03
Eucalyptus diversifolia
Eugenia selloi
0
0.03
Plinia cauliflora
Syzygium forrestii
0
0.02
Pimenta dioica
Syzygium cumini
0
0.02
Eucalyptus camaldulensis
Punica granatum
0
0
Plinia aureana
0.03
Eucalyptus globulus
Eucalyptus grandis
0
0.03
Eugenia brasiliensis
Psidium cattleyanum
0
0.03
Feijoa sellowiana
Angophora floribunda
0
Stockwellia quadrifida
0
Eugenia uniflora
0.02
0
0.02
Myrcianthes pungens
Psidium galapageium
0.03
Eugenia pyriformis
Plinia trunciflora
0
Campomanesia xanthocarpa
Heteropyxis natalensis
2.0E-4

*rps8*

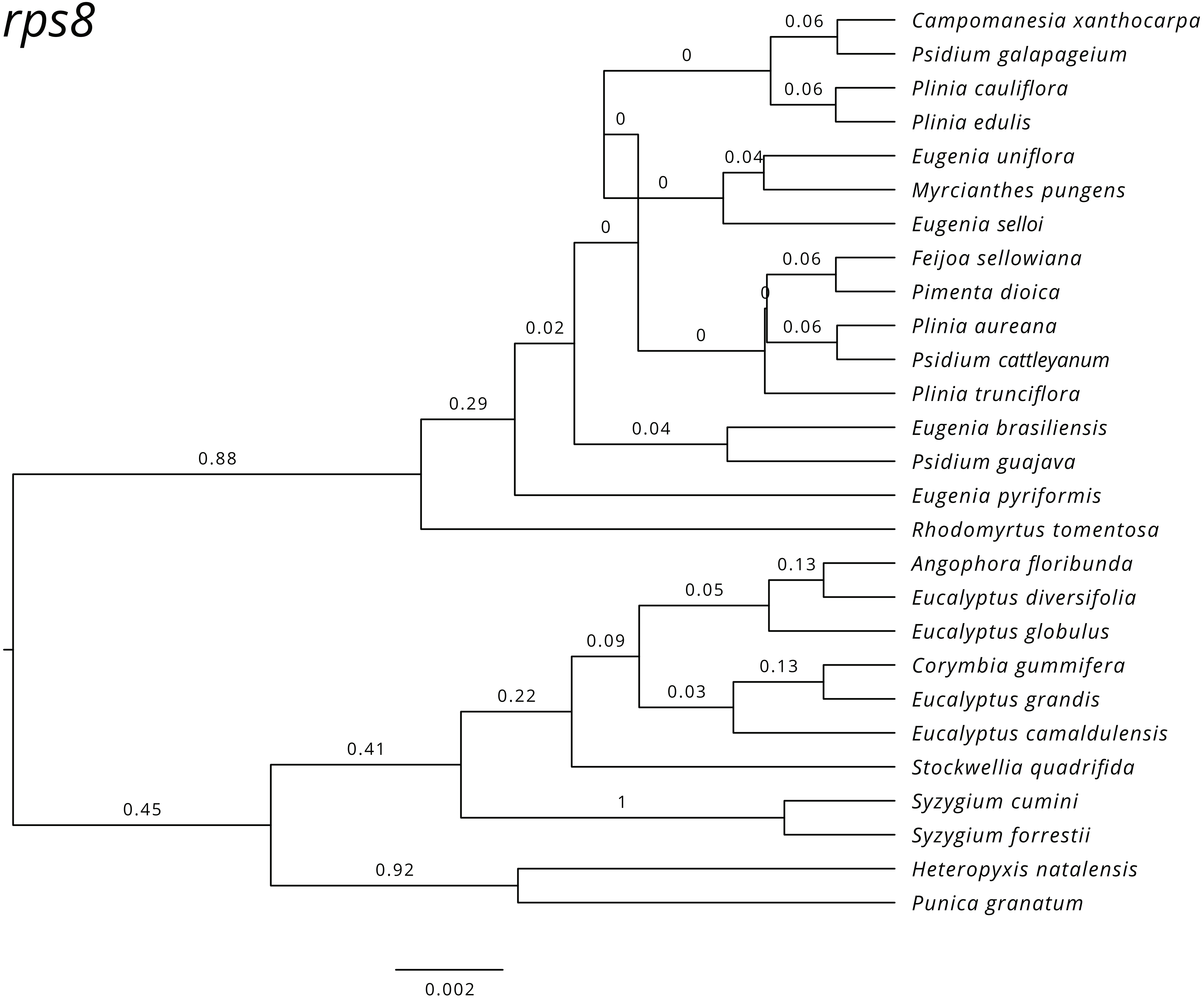
0.06
0
0.06
0
0.04
0
0
0.06
0
0.06
0
0.02
0.29
0.04
0.88
0.13
0.05
0.09
0.13
0.03
0.22
0.41
1
0.45
0.92
0.002
Campomanesia xanthocarpa
Psidium galapageium
Plinia cauliflora
Plinia edulis
Eugenia uniflora
Myrcianthes pungens
Eugenia selloi
Feijoa sellowiana
Pimenta dioica
Plinia aureana
Psidium cattleyanum
Plinia trunciflora
Eugenia brasiliensis
Psidium guajava
Eugenia pyriformis
Rhodomyrtus tomentosa
Angophora floribunda
Eucalyptus diversifolia
Eucalyptus globulus
Corymbia gummifera
Eucalyptus grandis
Eucalyptus camaldulensis
Stockwellia quadrifida
Syzygium cumini
Syzygium forrestii
Heteropyxis natalensis
Punica granatum

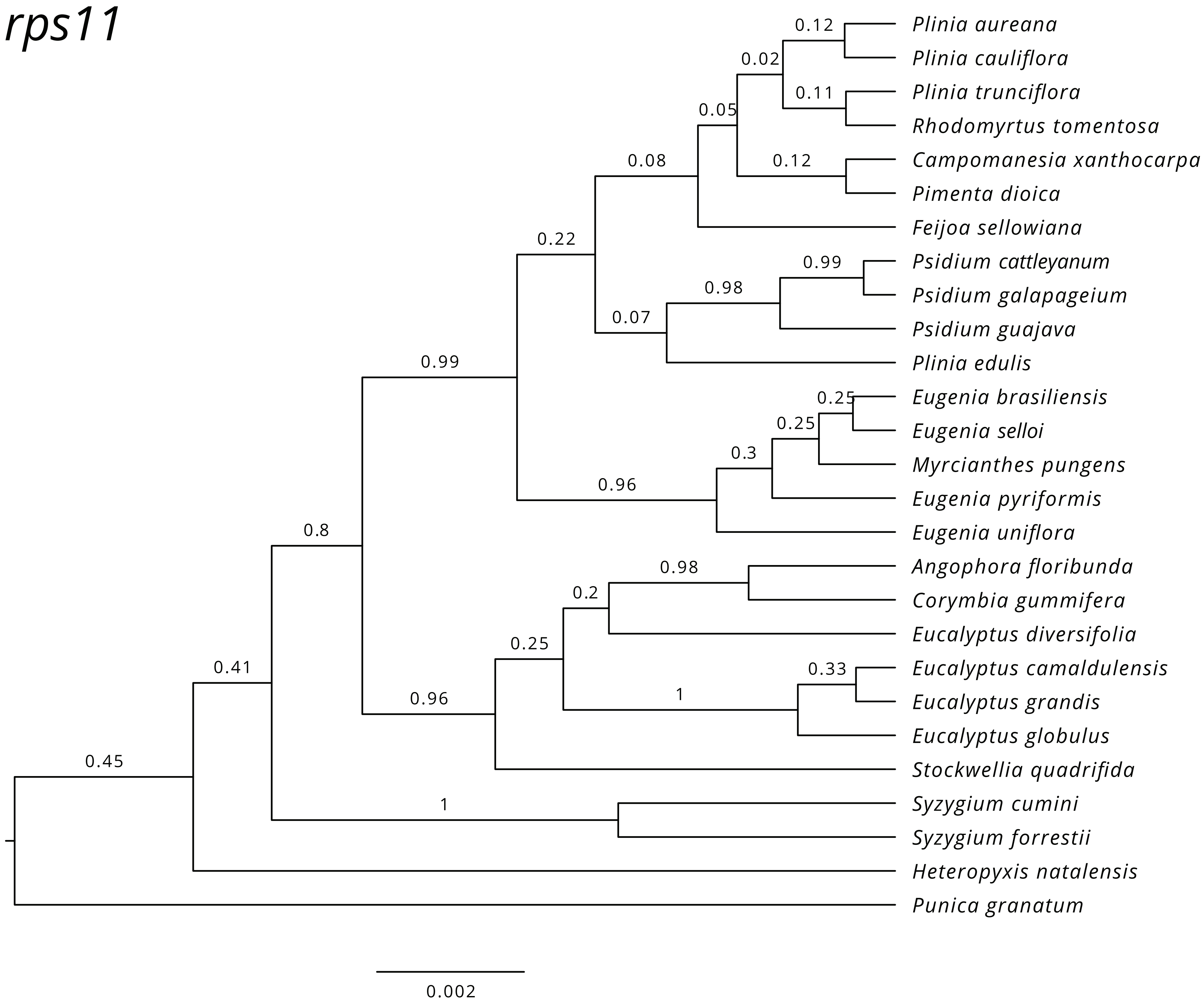
rps11
0.12
Plinia aureana
Plinia cauliflora
0.02
0.11
Plinia trunciflora
0.05
Rhodomyrtus tomentosa
0.12
Campomanesia xanthocarpa
0.08
Pimenta dioica
Feijoa sellowiana
0.22
0.99
Psidium cattleyanum
Psidium galapageium
0.98
Psidium guajava
0.07
Plinia edulis
0.99
0.25
Eugenia brasiliensis
Eugenia selloi
0.25
0.3
Myrcianthes pungens
Eugenia pyriformis
0.96
Eugenia uniflora
0.8
0.98
Angophora floribunda
0.2
Corymbia gummifera
Eucalyptus diversifolia
0.25
0.33
Eucalyptus camaldulensis
0.41
1
Eucalyptus grandis
0.96
Eucalyptus globulus
Stockwellia quadrifida
0.45
1
Syzygium cumini
Syzygium forrestii
Heteropyxis natalensis
Punica granatum
0.002

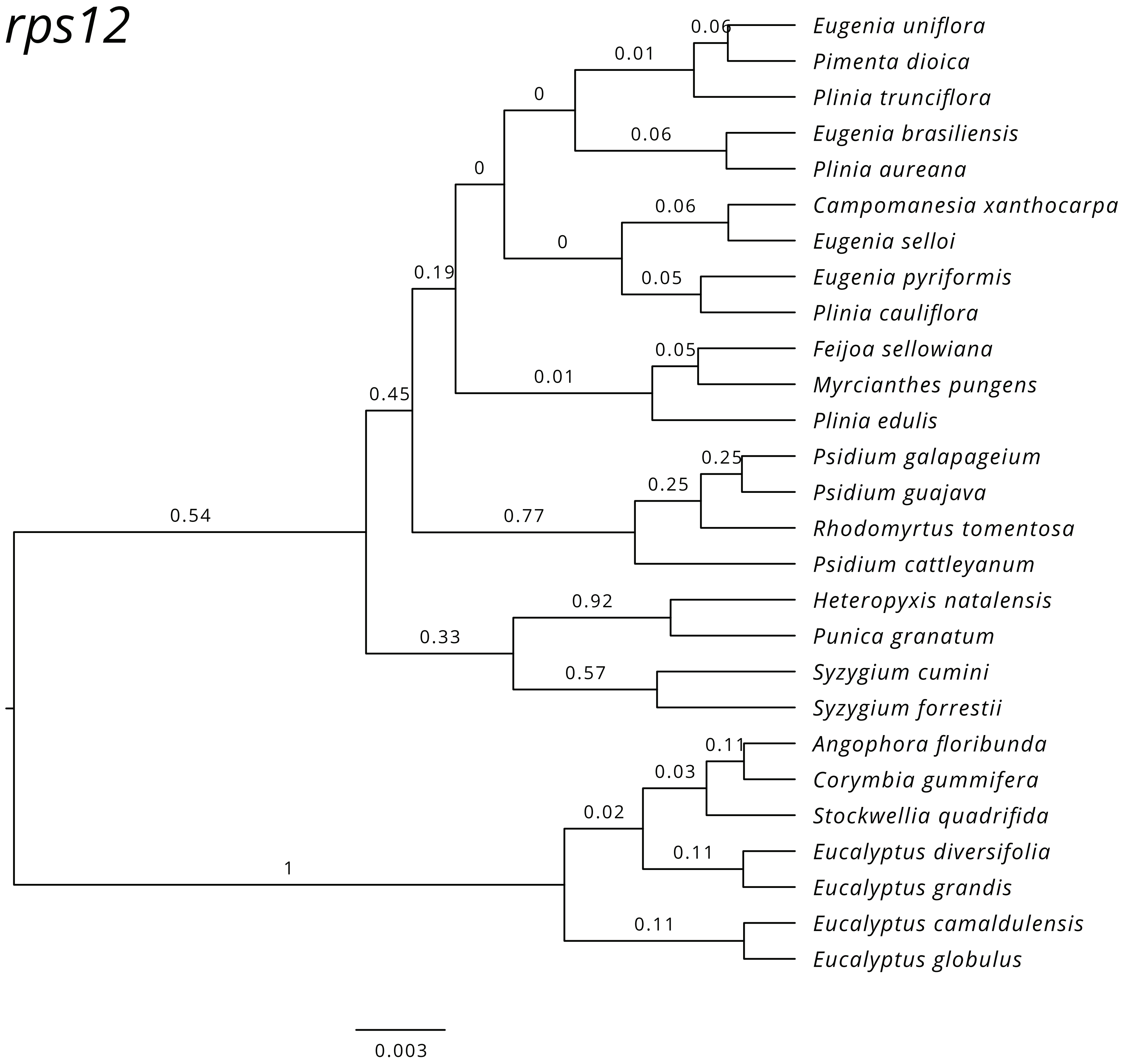
rps12
0.06
0.01
0
0.06
0
0.06
0
0.05
0.19
0.05
0.01
0.45
0.25
0.25
0.54
0.77
0.92
0.33
0.57
0.11
0.03
0.02
0.11
1
0.11
0.003
Eugenia uniflora
Pimenta dioica
Plinia trunciflora
Eugenia brasiliensis
Plinia aureana
Campomanesia xanthocarpa
Eugenia selloi
Eugenia pyriformis
Plinia cauliflora
Feijoa sellowiana
Myrcianthes pungens
Plinia edulis
Psidium galapageium
Psidium guajava
Rhodomyrtus tomentosa
Psidium cattleyanum
Heteropyxis natalensis
Punica granatum
Syzygium cumini
Syzygium forrestii
Angophora floribunda
Corymbia gummifera
Stockwellia quadrifida
Eucalyptus diversifolia
Eucalyptus grandis
Eucalyptus camaldulensis
Eucalyptus globulus

# *rps14*

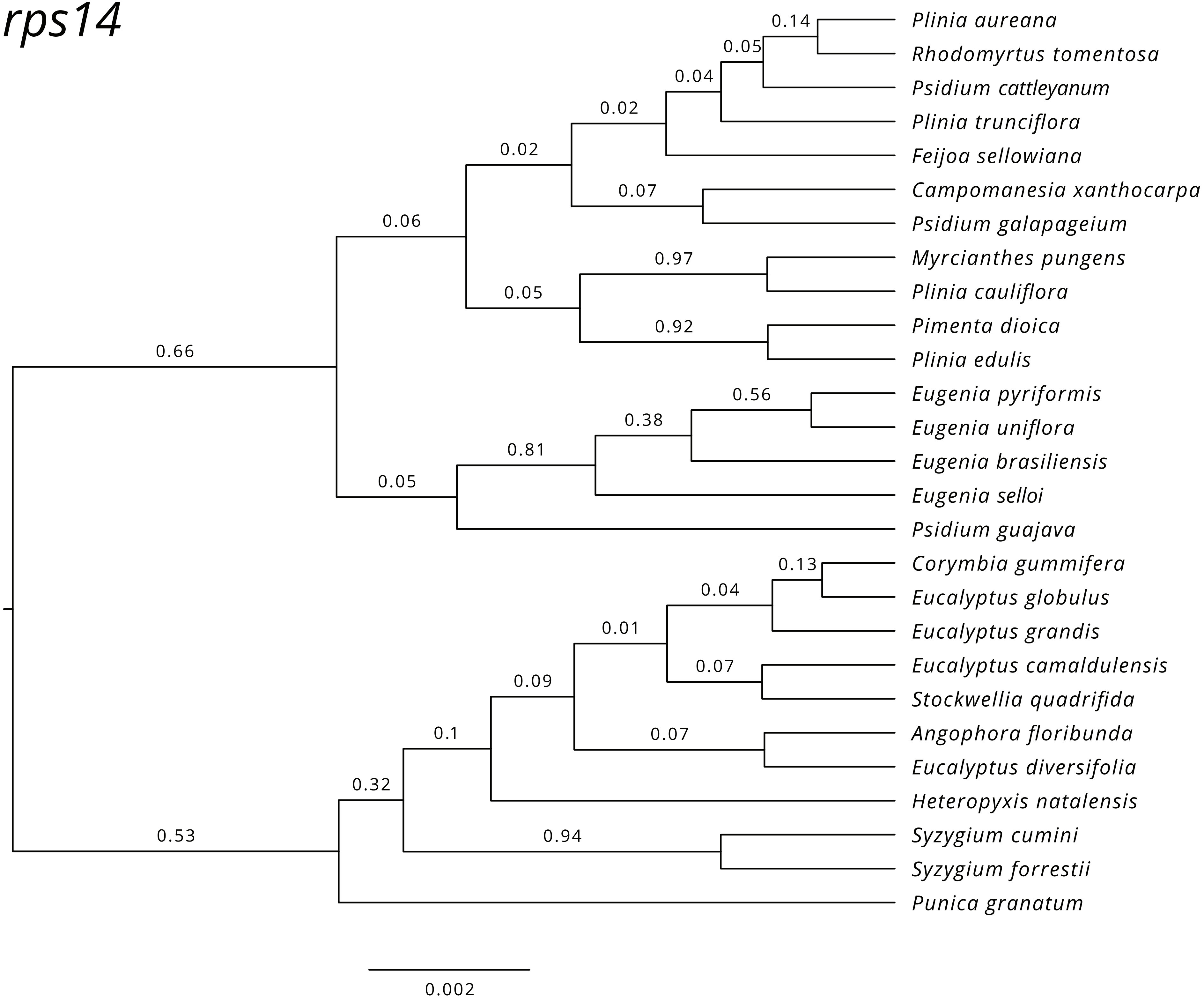
0.14
0.05
0.04
0.02
0.02
0.07
0.06
0.97
0.05
0.92
0.66
0.56
0.38
0.81
0.05
0.13
0.04
0.01
0.07
0.09
0.07
0.1
0.32
0.94
0.53
*Plinia aureana*
*Rhodomyrtus tomentosa*
*Psidium cattleyanum*
*Plinia trunciflora*
*Feijoa sellowiana*
*Campomanesia xanthocarpa*
*Psidium galapageium*
*Myrcianthes pungens*
*Plinia cauliflora*
*Pimenta dioica*
*Plinia edulis*
*Eugenia pyriformis*
*Eugenia uniflora*
*Eugenia brasiliensis*
*Eugenia selloi*
*Psidium guajava*
*Corymbia gummifera*
*Eucalyptus globulus*
*Eucalyptus grandis*
*Eucalyptus camaldulensis*
*Stockwellia quadrifida*
*Angophora floribunda*
*Eucalyptus diversifolia*
*Heteropyxis natalensis*
*Syzygium cumini*
*Syzygium forrestii*
*Punica granatum*
0.002

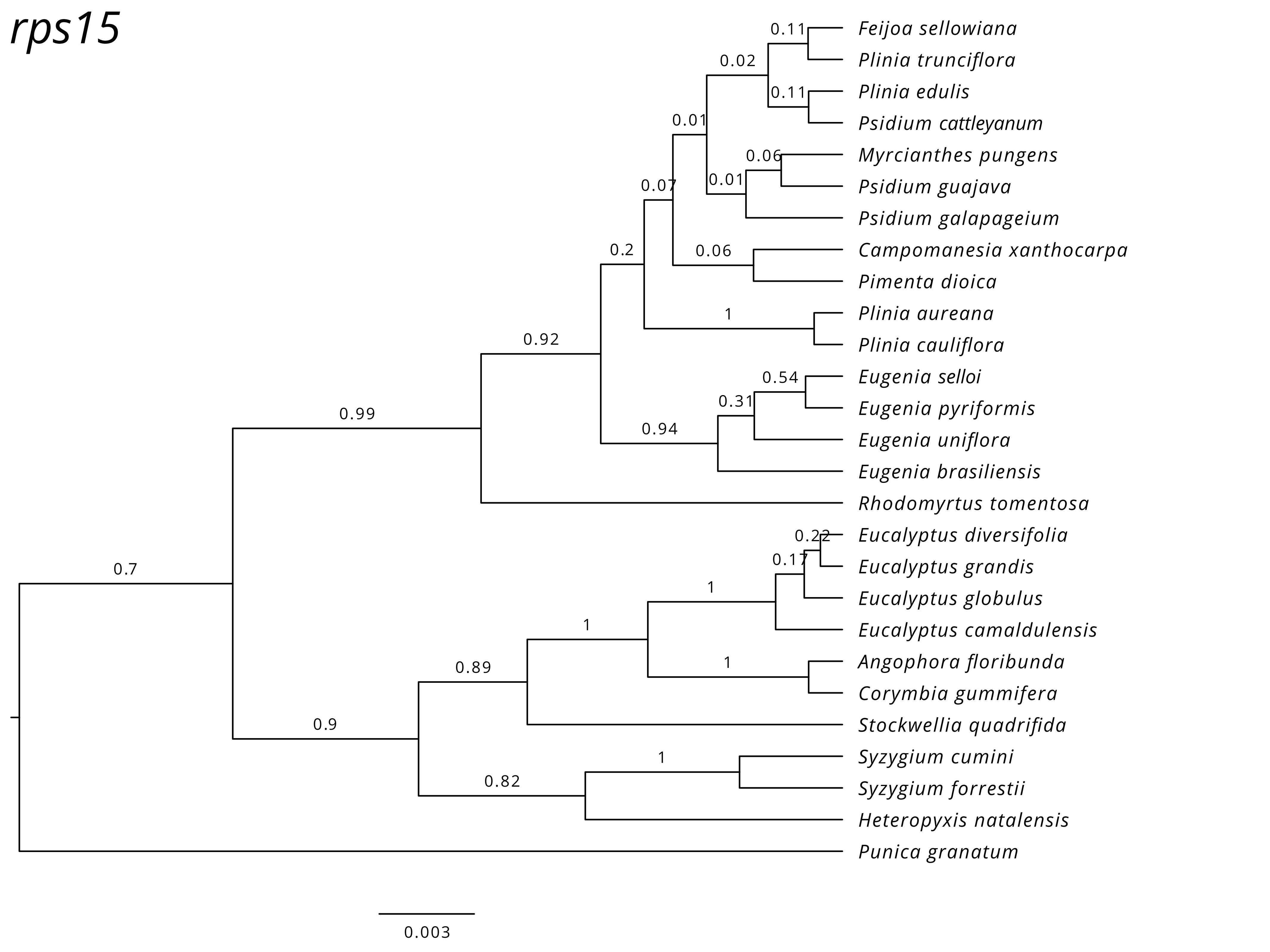
rps15
0.11
0.02
0.11
0.01
0.06
0.01
0.07
0.2
0.06
1
0.92
0.54
0.31
0.99
0.94
0.22
0.17
0.7
1
1
1
0.89
0.9
1
0.82
0.003
Feijoa sellowiana
Plinia trunciflora
Plinia edulis
Psidium cattleyanum
Myrcianthes pungens
Psidium guajava
Psidium galapageium
Campomanesia xanthocarpa
Pimenta dioica
Plinia aureana
Plinia cauliflora
Eugenia selloi
Eugenia pyriformis
Eugenia uniflora
Eugenia brasiliensis
Rhodomyrtus tomentosa
Eucalyptus diversifolia
Eucalyptus grandis
Eucalyptus globulus
Eucalyptus camaldulensis
Angophora floribunda
Corymbia gummifera
Stockwellia quadrifida
Syzygium cumini
Syzygium forrestii
Heteropyxis natalensis
Punica granatum

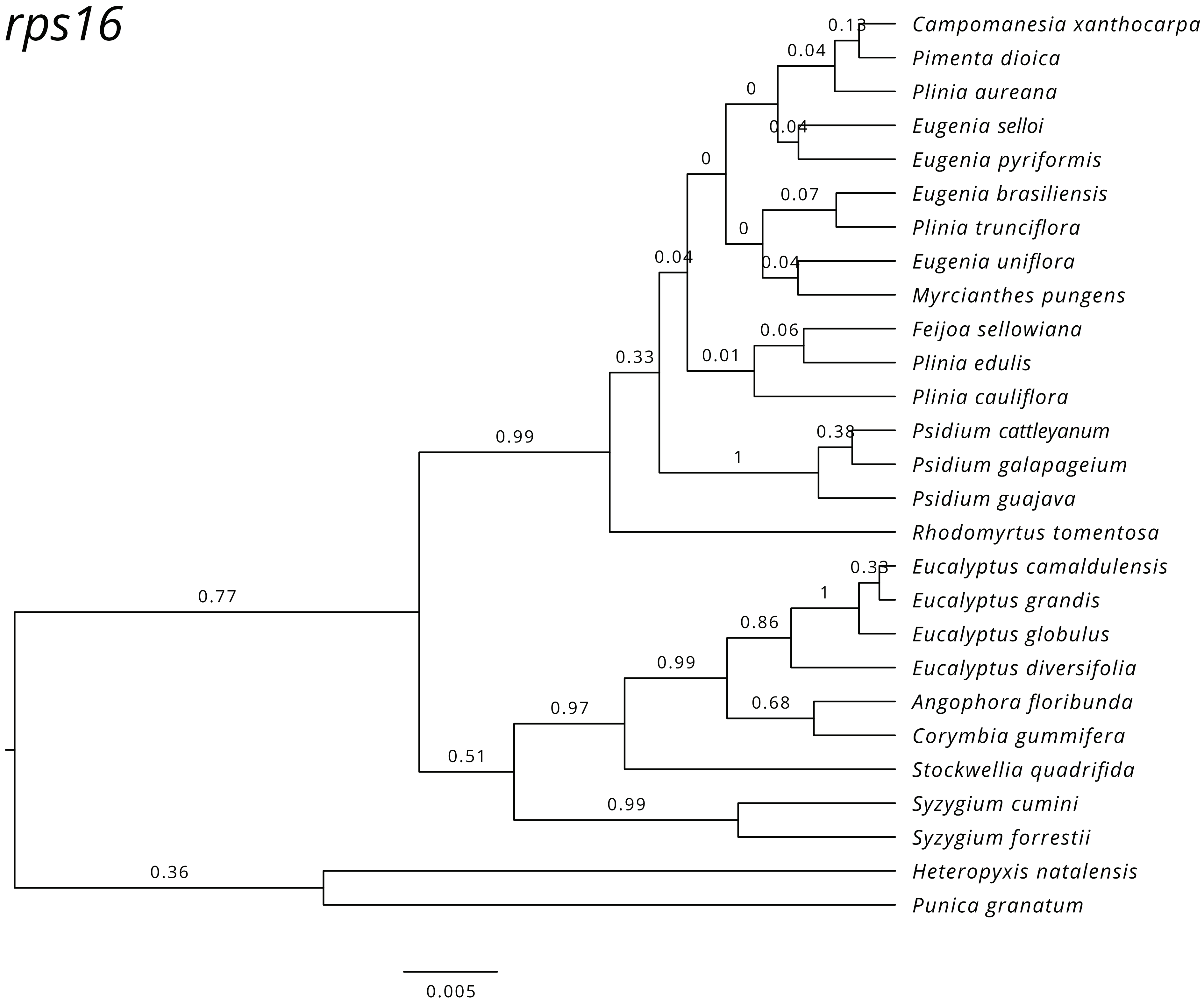
rps16
0.13
0.04
0
0.04
0
0.07
0
0.04
0.04
0.06
0.01
0.33
0.99
0.38
1
0.77
0.33
1
0.86
0.99
0.68
0.97
0.51
0.99
0.36
0.005
Campomanesia xanthocarpa
Pimenta dioica
Plinia aureana
Eugenia selloi
Eugenia pyriformis
Eugenia brasiliensis
Plinia trunciflora
Eugenia uniflora
Myrcianthes pungens
Feijoa sellowiana
Plinia edulis
Plinia cauliflora
Psidium cattleyanum
Psidium galapageium
Psidium guajava
Rhodomyrtus tomentosa
Eucalyptus camaldulensis
Eucalyptus grandis
Eucalyptus globulus
Eucalyptus diversifolia
Angophora floribunda
Corymbia gummifera
Stockwellia quadrifida
Syzygium cumini
Syzygium forrestii
Heteropyxis natalensis
Punica granatum

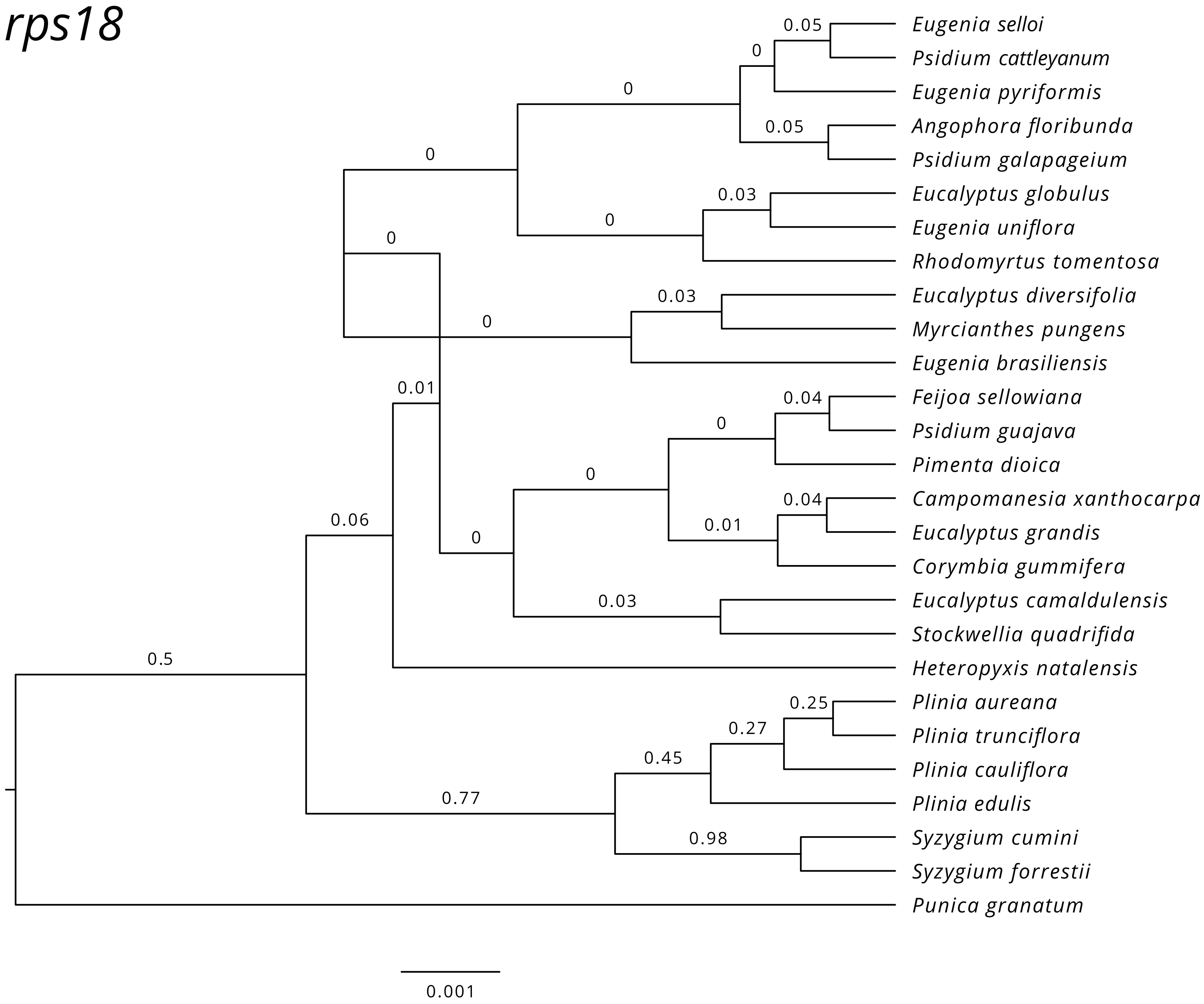
rps18
0.05
0
0
0.05
0
0.03
0
0
0.03
0
0.01
0.04
0
0
0.04
0.01
0.06
0
0.03
0.5
0.25
0.27
0.45
0.77
0.98
Eugenia selloi
Psidium cattleyanum
Eugenia pyriformis
Angophora floribunda
Psidium galapageium
Eucalyptus globulus
Eugenia uniflora
Rhodomyrtus tomentosa
Eucalyptus diversifolia
Myrcianthes pungens
Eugenia brasiliensis
Feijoa sellowiana
Psidium guajava
Pimenta dioica
Campomanesia xanthocarpa
Eucalyptus grandis
Corymbia gummifera
Eucalyptus camaldulensis
Stockwellia quadrifida
Heteropyxis natalensis
Plinia aureana
Plinia trunciflora
Plinia cauliflora
Plinia edulis
Syzygium cumini
Syzygium forrestii
Punica granatum
0.001

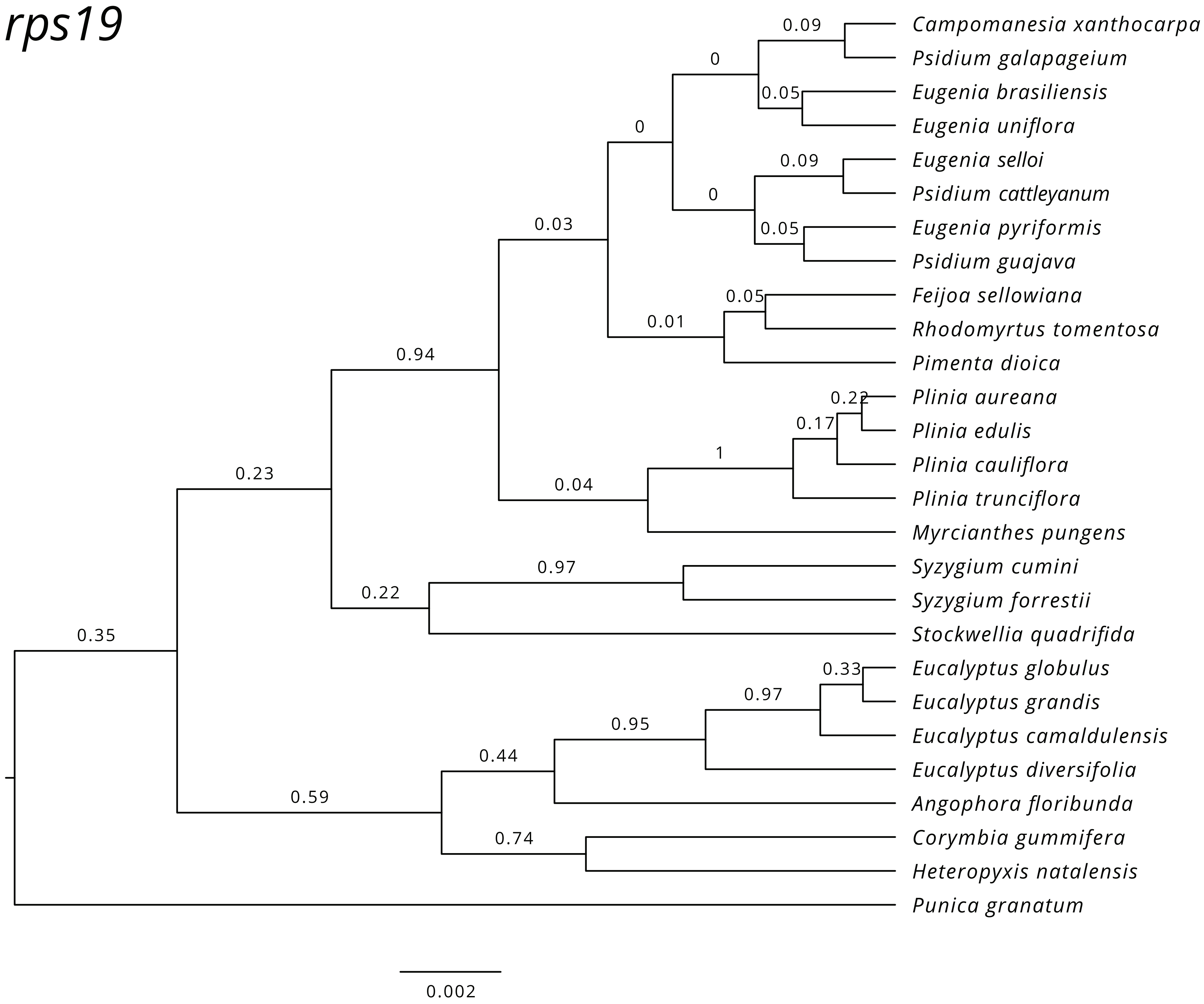
rps19
0.09
0
0.05
0
0.09
0
0.05
0.03
0.05
0.01
0.94
0.22
0.17
1
0.04
0.23
0.97
0.22
0.35
0.33
0.97
0.95
0.44
0.59
0.74
0.002
Campomanesia xanthocarpa
Psidium galapageium
Eugenia brasiliensis
Eugenia uniflora
Eugenia selloi
Psidium cattleyanum
Eugenia pyriformis
Psidium guajava
Feijoa sellowiana
Rhodomyrtus tomentosa
Pimenta dioica
Plinia aureana
Plinia edulis
Plinia cauliflora
Plinia trunciflora
Myrcianthes pungens
Syzygium cumini
Syzygium forrestii
Stockwellia quadrifida
Eucalyptus globulus
Eucalyptus grandis
Eucalyptus camaldulensis
Eucalyptus diversifolia
Angophora floribunda
Corymbia gummifera
Heteropyxis natalensis
Punica granatum

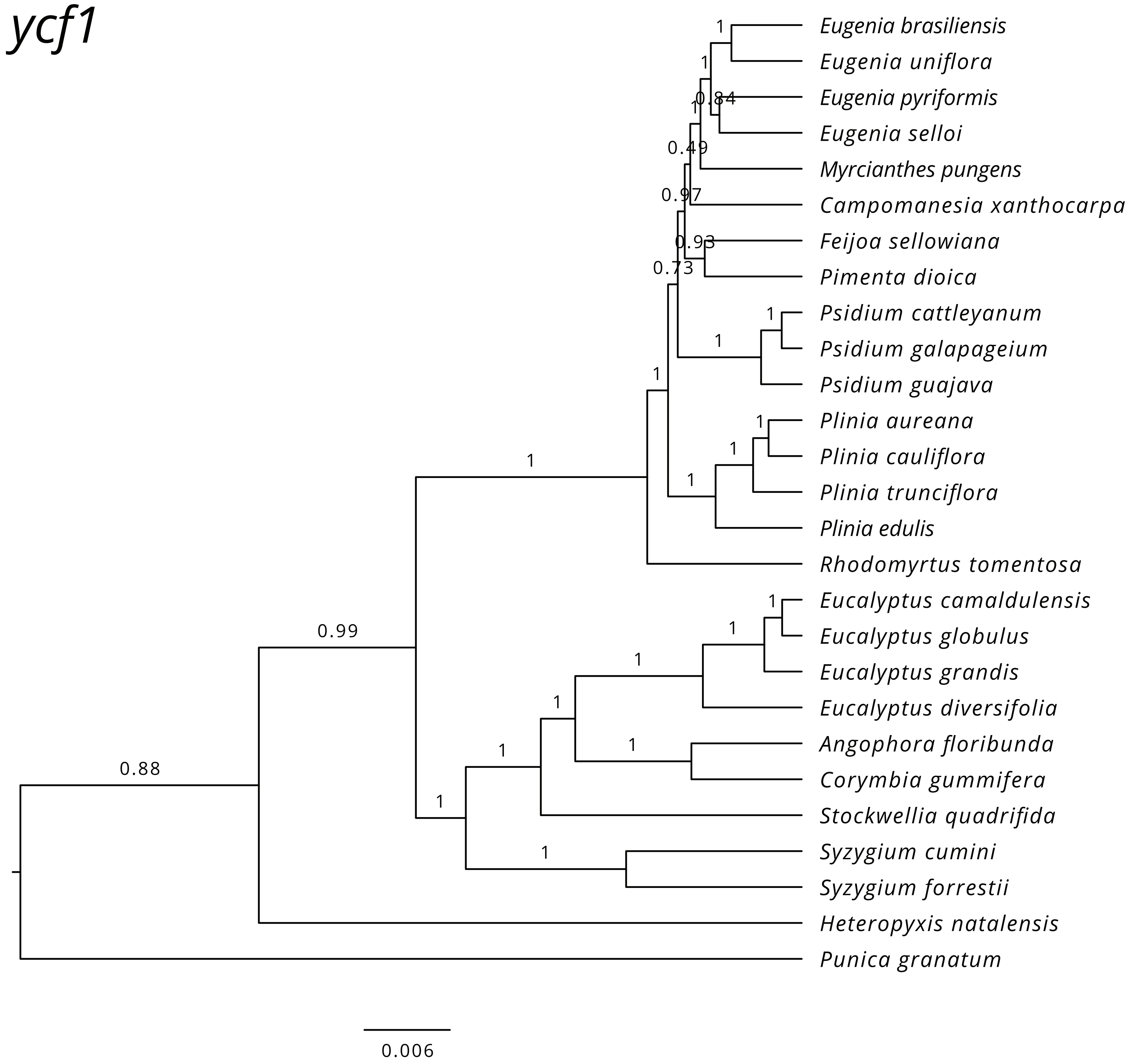
ycf1
Eugenia brasiliensis
Eugenia uniflora
Eugenia pyriformis
Eugenia selloi
Myrcianthes pungens
Campomanesia xanthocarpa
Feijoa sellowiana
Pimenta dioica
Psidium cattleyanum
Psidium galapageium
Psidium guajava
Plinia aureana
Plinia cauliflora
Plinia trunciflora
Plinia edulis
Rhodomyrtus tomentosa
Eucalyptus camaldulensis
Eucalyptus globulus
Eucalyptus grandis
Eucalyptus diversifolia
Angophora floribunda
Corymbia gummifera
Stockwellia quadrifida
Syzygium cumini
Syzygium forrestii
Heteropyxis natalensis
Punica granatum
1
1
0.84
1
0.49
0.97
0.93
0.73
1
1
1
1
1
1
1
1
0.99
1
1
1
1
1
1
1
0.88
0.006

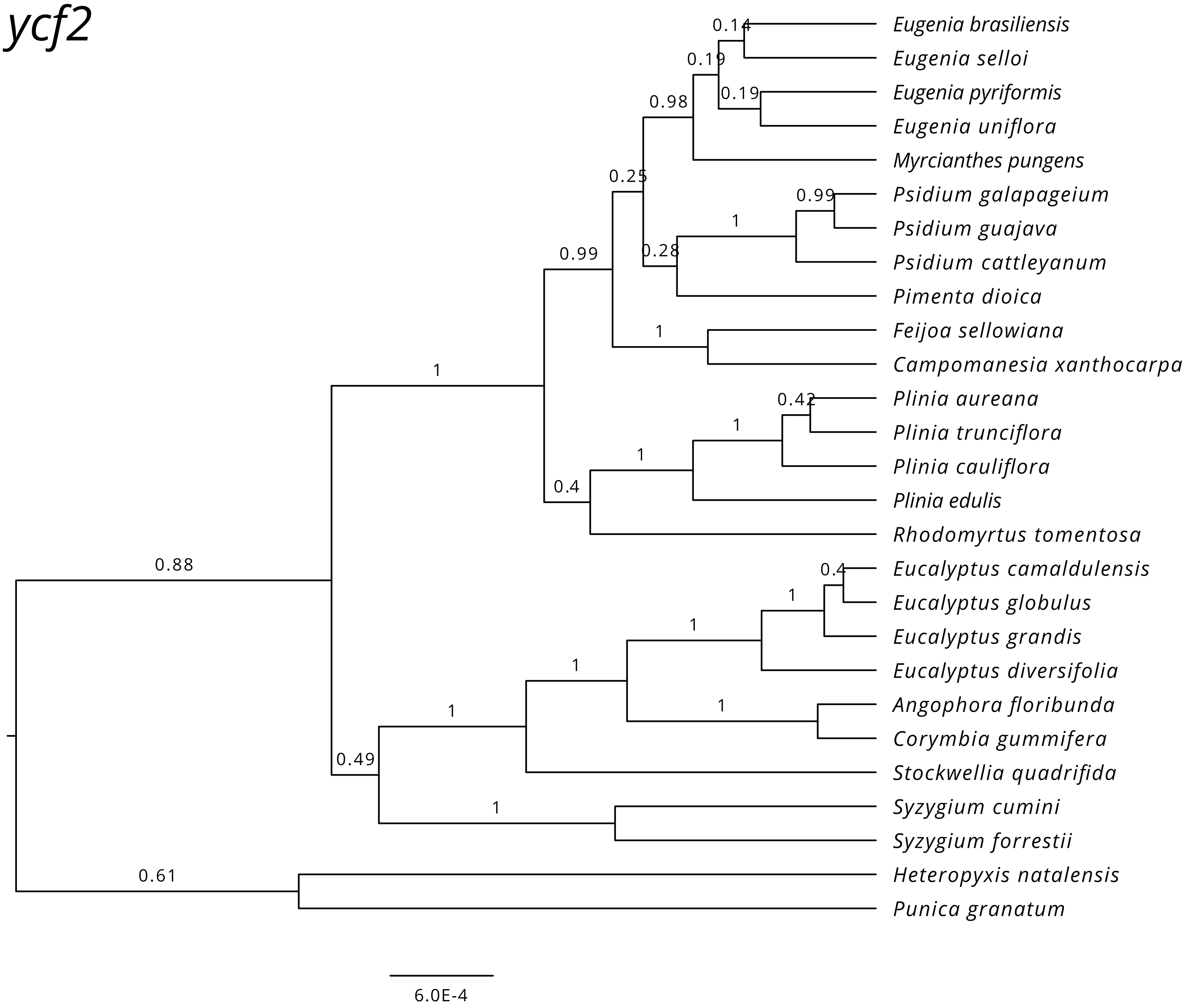
ycf2
Eugenia brasiliensis
Eugenia selloi
Eugenia pyriformis
Eugenia uniflora
Myrcianthes pungens
Psidium galapageium
Psidium guajava
Psidium cattleyanum
Pimenta dioica
Feijoa sellowiana
Campomanesia xanthocarpa
Plinia aureana
Plinia trunciflora
Plinia cauliflora
Plinia edulis
Rhodomyrtus tomentosa
Eucalyptus camaldulensis
Eucalyptus globulus
Eucalyptus grandis
Eucalyptus diversifolia
Angophora floribunda
Corymbia gummifera
Stockwellia quadrifida
Syzygium cumini
Syzygium forrestii
Heteropyxis natalensis
Punica granatum
0.14
0.19
0.19
0.98
0.25
0.99
1
0.28
0.99
1
1
0.42
1
1
0.4
0.88
0.4
1
1
1
1
1
0.49
1
0.61
6.0E-4

# *ycf3*

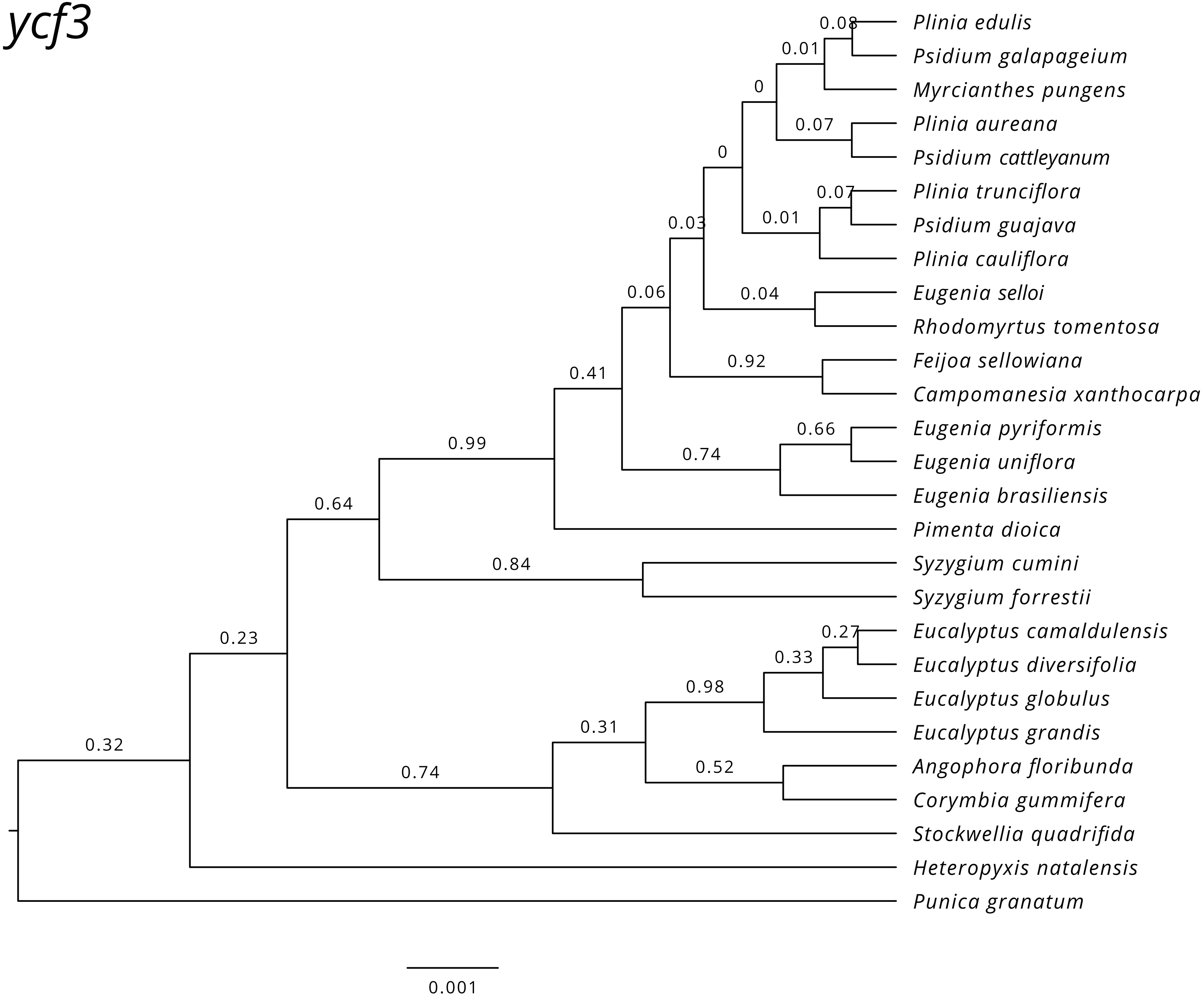
0.08
0.01
0
0.07
0
0.07
0.01
0.03
0.04
0.06
0.92
0.41
0.66
0.74
0.99
0.64
0.84
0.27
0.33
0.98
0.23
0.31
0.52
0.32
0.74
Plinia edulis
Psidium galapageium
Myrcianthes pungens
Plinia aureana
Psidium cattleyanum
Plinia trunciflora
Psidium guajava
Plinia cauliflora
Eugenia selloi
Rhodomyrtus tomentosa
Feijoa sellowiana
Campomanesia xanthocarpa
Eugenia pyriformis
Eugenia uniflora
Eugenia brasiliensis
Pimenta dioica
Syzygium cumini
Syzygium forrestii
Eucalyptus camaldulensis
Eucalyptus diversifolia
Eucalyptus globulus
Eucalyptus grandis
Angophora floribunda
Corymbia gummifera
Stockwellia quadrifida
Heteropyxis natalensis
Punica granatum
0.001

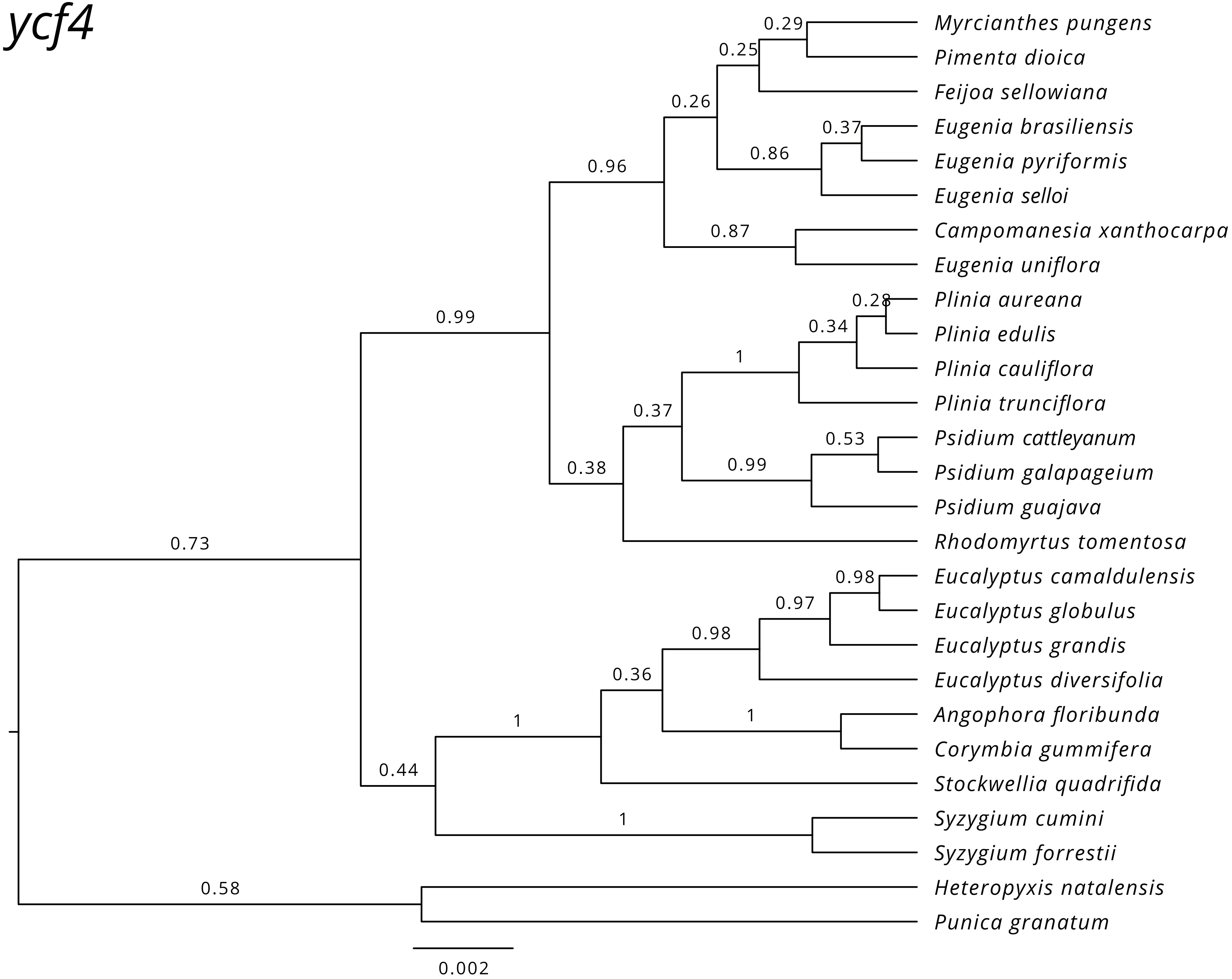


**Figure S4** - Individual maximum clade-credibility phylogenies of Myrtaceae inferred from the 78 plastid protein-coding sequences. Posterior probabilities for each node are indicated and branch lengths are scaled according to number of substitutions per site.
