# Supplementary material for: Perspectives in Myrtaceae evolution from plastomes and nuclear phylogenies
Source: Genet Mol Biol. 2022 Jan 21;45(1):e20210191. doi: 10.1590/1678-4685-GMB-2021-0191 (PMC8796035; doi:10.1590/1678-4685-GMB-2021-0191)
Supplement: Figure S7 - [file 1415-4757-GMB-45-1-e20210191-s13.pdf]

Genetics and Molecular Biology
https://doi.org/10.1590/1678-4685-GMB-2021-0191

Supplementary Material to "Perspectives in Myrtaceae evolution from plastomes and nuclear phylogenies"

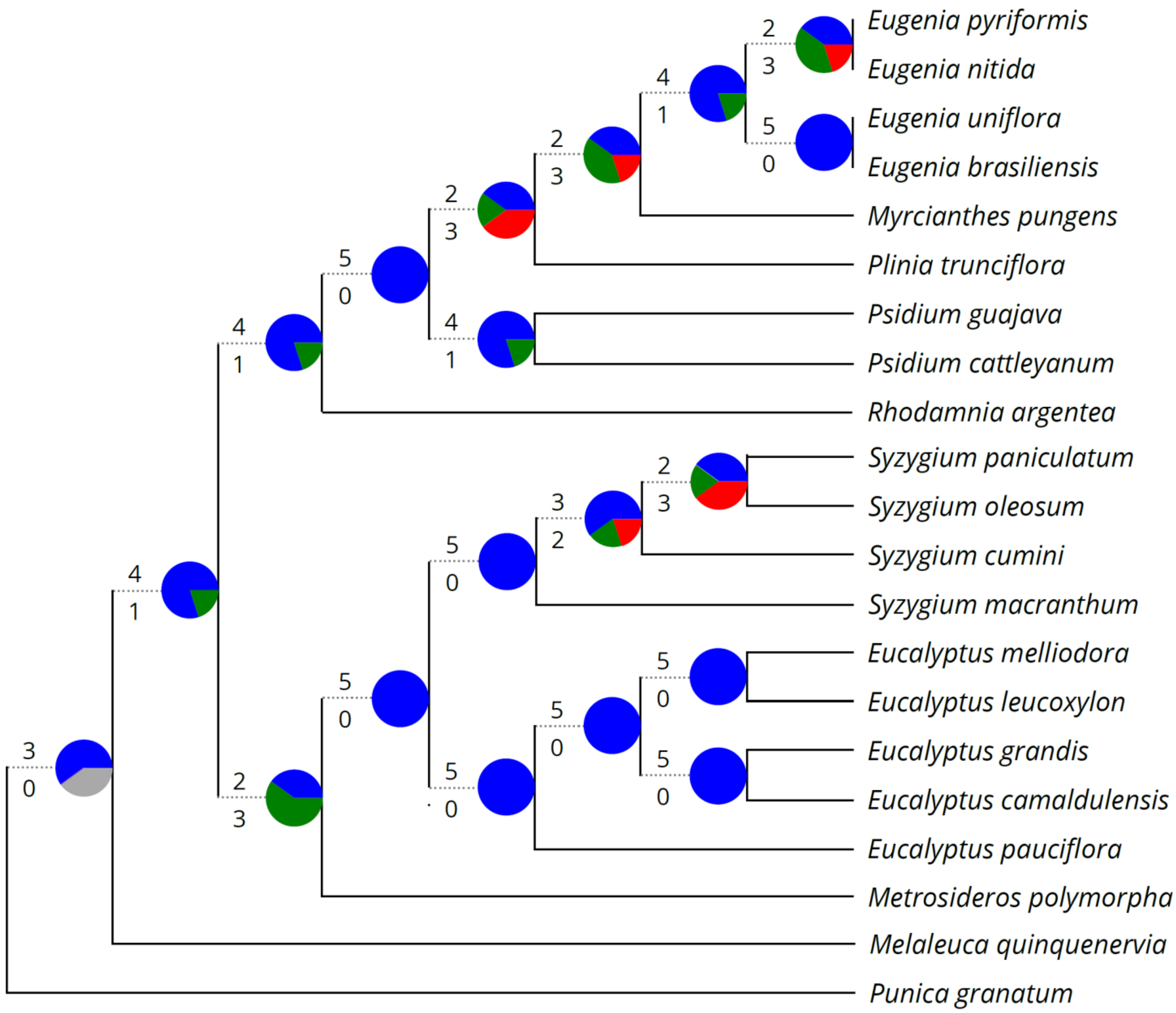

Figure S7 - Bipartition analysis of Myrtaceae phylogeny using the five single-copy nuclear markers *MCM5*, *MLH1*, *MSH1*, *SMC1* and *SMC2*. Pie charts and the numbers in the nodes represent the concordant (blue), discordant (red) and discordant prevalent alternative (green) bipartitions. Numbers above the branch correspond to the concordant bipartitions, while numbers under the branch represent the number of genes supporting a discordant prevalent bipartition + other discordant bipartitions.
